# Fragments of rDNA Genes Scattered over the Human Genome Are Targets of Small RNAs

**DOI:** 10.3390/ijms23063014

**Published:** 2022-03-10

**Authors:** Nickolai A. Tchurikov, Elena S. Klushevskaya, Ildar R. Alembekov, Anastasiia S. Bukreeva, Antonina N. Kretova, Vladimir R. Chechetkin, Galina I. Kravatskaya, Yuri V. Kravatsky

**Affiliations:** 1 Department of Epigenetic Mechanisms of Gene Expression Regulation, Engelhardt Institute of Molecular Biology Russian Academy of Sciences, 119334 Moscow, Russia; giedre@inbox.ru (E.S.K.); alembeki@gmail.com (I.R.A.); asa192bukreeva@yandex.ru (A.S.B.); tonya_kretova@mail.ru (A.N.K.); vladimir_chechet@mail.ru (V.R.C.); galina.kravatskaya@gmail.com (G.I.K.); jiri@eimb.ru (Y.V.K.); 2 Center for Precision Genome Editing and Genetic Technologies for Biomedicine, Engelhardt Institute of Molecular Biology Russian Academy of Sciences, 119334 Moscow, Russia

**Keywords:** small ribosomal RNAs (srRNAs), rDNA fragments, HEK293T, epigenetics, transcriptional silencing, transcriptional activation, differentiation

## Abstract

Small noncoding RNAs of different origins and classes play several roles in the regulation of gene expression. Here, we show that diverged and rearranged fragments of rDNA units are scattered throughout the human genome and that endogenous small noncoding RNAs are processed by the Microprocessor complex from specific regions of ribosomal RNAs shaping hairpins. These small RNAs correspond to particular sites inside the fragments of rDNA that mostly reside in intergenic regions or the introns of about 1500 genes. The targets of these small ribosomal RNAs (srRNAs) are characterized by a set of epigenetic marks, binding sites of Pol II, RAD21, CBP, and P300, DNase I hypersensitive sites, and by enrichment or depletion of active histone marks. In HEK293T cells, genes that are targeted by srRNAs (srRNA target genes) are involved in differentiation and development. srRNA target genes are enriched with more actively transcribed genes. Our data suggest that remnants of rDNA sequences and srRNAs may be involved in the upregulation or downregulation of a specific set of genes in human cells. These results have implications for diverse fields, including epigenetics and gene therapy.

## 1. Introduction

RNA molecules are capable of recognizing complementary genomic regions [1]. The pervasive transcription of RNA likely gives rise to RNA copies of the entire genome [2]. Small RNAs of different classes (miRNA, siRNA, piRNA, tsRNA, srRNA, and others) and long noncoding RNAs (lncRNAs) play important roles in the regulation of gene expression in metazoan organisms [3,4,5,6]. Small rDNA-derived RNAs (srRNAs) bind to the AGO protein complex and may be involved in various signaling pathways and can affect the levels of ribosomal proteins [7,8,9]. Recently, it was shown that rDNA clusters shape inter-chromosomal contacts within different genomic regions in HEK293T cells and that the contact sites are enriched with small noncoding RNAs, suggesting the RNA-mediated nature of the contacts [10,11]. These data prompted us to study whether srRNAs are involved in these contacts, and thus we investigated the origin and the target sites of srRNAs. For this study, we used the 20–50-nt long RNAs associated with DGCR8—a Microprocessor-complex subunit—that was isolated by crosslinking immunoprecipitation in HEK293T cells [12]. DGCR8 (also known as Pasha) directly recognizes the RNA substrates and is involved not only in the initial step of miRNA biogenesis but also in the fate of different classes of RNAs, including ribosomal RNAs and several hundred mRNAs, as well as snoRNAs and lncRNAs [12]. srRNAs mostly correspond to the 28S gene [6] and are not random products formed during rRNA degradation, but correspond to a new class of small RNAs that deserves further investigation [13].

Here, we report that srRNAs in HEK293T cells correspond to short sequences (targets) from the transcribed portion of rDNA units in about 1500 srRNA target genes that are enriched in genes involved in differentiation. About one-third of srRNA target genes are involved in shaping the contacts with rDNA clusters. We observed that the level of expression of srRNA target genes varies widely. Surprisingly, some abundant srRNAs correspond to the targets in a single gene, suggesting high specificity toward the target gene. srRNA-target sites are enriched either with active or repressive epigenetic marks. Taken together, our data suggest that srRNAs may be involved in the transcriptional regulation of multiple genes.

## 2. Results

### 2.1. There Are Thousands of Unique srRNAs

**Figure 1:**
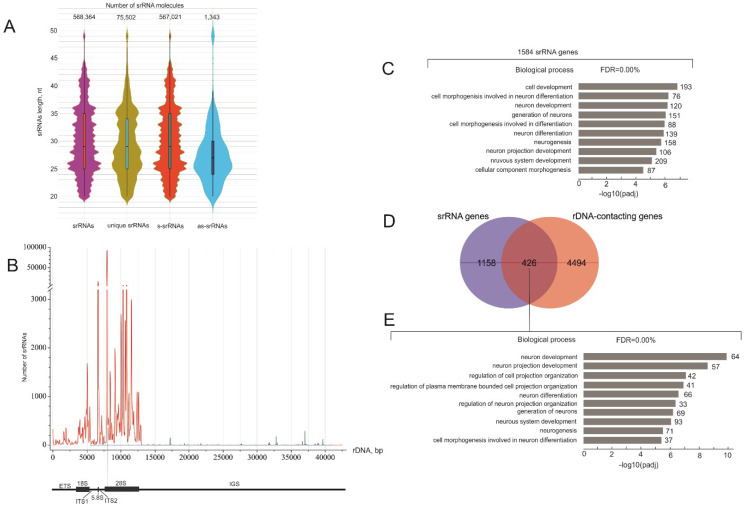
Characterization of srRNAs and their target genes. (**A**) Violin presentation of srRNA lengths and abundance, including all isolated molecules, unique molecules (without exact copies), and sense and antisense molecules. The complete data are presented in Table S1. (**B**) The distribution of sense (red curve) and antisense (blue curve) srRNAs along the length of a 43 kb rDNA unit. The vertical line rising from the 28S gene indicates the highest peak of srRNAs. (**C**) The top ten Gene Ontology (GO) biological process associations of srRNA target genes. The values to the right of the bars show the number of srRNA target genes associated with a process. The complete list of srRNA target genes is shown in Table S1. Table S2 shows the results of the GO search. (**D**) A Venn diagram showing the intersections between srRNA target genes and rDNA-contacting genes [9]. Table S3 shows the list of overlapping genes. (**E**) The top ten GO biological process associations of 426 genes are shown in (**D**). The values to the right of the bars show the number of srRNA target genes associated with a process. Table S4 shows the results of the corresponding GO search.

srRNAs were selected from the sequenced library of RNAs that were isolated by crosslinking immunoprecipitation using antibodies to DGCR8 (sample GSM955512) [12]. About 20% of the reads correspond to rDNA sequences [12]. The isolation of total small-RNA reads and the selection of srRNAs and the corresponding genes were performed as described in Section 4. Figure 1A shows that more than 99% of srRNAs correspond to the sense strand of the 43-kb rDNA unit. There are about 75,000 unique srRNA molecules, which comprise overlapping 19–50-nt rRNA molecules that align with 2047 regions of rDNA (Table S1). Most srRNAs have numerous exact copies. The violin plot presenting the length distribution of all srRNAs shows that the number of nucleotides ranges from 19 to 50 with a mean value of 29 nt. There are also major peaks at 25 and 26 nt. Interestingly, the scarce antisense srRNAs are shorter and have a mean value of 27 nt, with a major peak at 25 nt.

Figure 1B shows the distribution of the relative amounts of srRNAs along the length of the rDNA unit. Small noncoding sense RNAs are distributed almost exclusively within the transcribed portion of the rDNA gene. Small peaks of antisense srRNAs were observed in intergenic spacer (IGS) regions (Figure 1B). srRNAs are non-randomly distributed throughout the rDNA, and we detected ten main peaks in the transcribed rDNA region. These include a peak from the 18S gene, a prominent peak from the 5.8S gene, and several large peaks from the 28S gene. The strongest peak corresponds to the 5′ region of the 28S gene.

### 2.2. Detection of srRNA Target Genes

Next, we searched for non-rDNA genes that may correspond to these srRNAs and share homologous nucleotide stretches. We found 1584 genes possessing sequences corresponding to the selected srRNAs (srRNA target genes). The list of these genes is shown in Table S1. In order to study whether srRNA target genes share biological properties, we used the Gene Ontology (GO) search and revealed that these genes in HEK293T cells were most frequently associated with a number of GO Biological Process items relating to cell development and neuron differentiation (Figure 1C, Table S2), which could probably be explained by the neuronal origin of HEK293T cells [14]. About 25% of srRNA genes overlapped with the list of rDNA-contacting genes [9] (Figure 1D, Table S3). These overlapping genes are highly associated with neuron development (Figure 1E, Table S4). The data indicate that srRNAs may be involved in RNA-mediated interchromosomal contacts of rDNA units with some genes; however, other RNAs or other mechanisms are responsible for a major part of these contacts.

### 2.3. Abundant srRNAs in the UNC45B Gene

**Figure 2:**
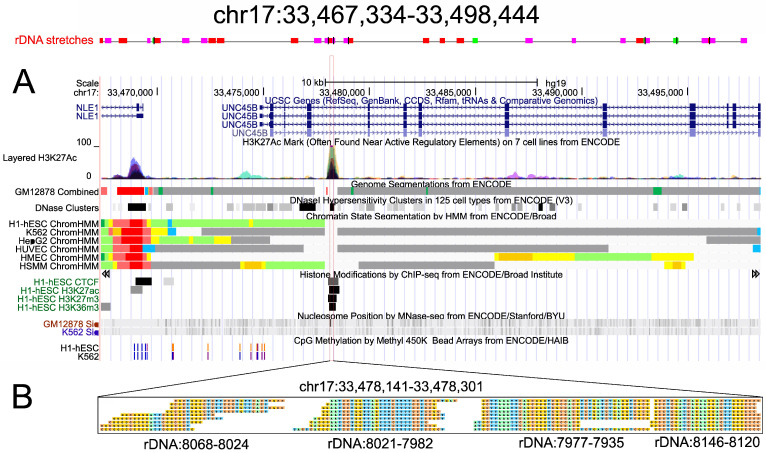
Characterization of srRNA targets inside the intron of the *UNC45B* gene. (**A**) Divergent rDNA stretches in the region are shown at the top. The colors indicate the alignment score of NCBI BLAST. The dot plot in Figure S1 shows the position of a short rearranged 5′ fragment of the 28S gene inside a segment of chr17. The distribution of layered H3K27ac marks, genome segmentation from ENCODE, histone modifications, nucleosome position, and CpG methylation inside a region of chr17 are shown as in the UCSC Browser. (**B**) Four groups of overlapping sequences of srRNAs of length 23–43 nt correspond to the main peak of sense srRNAs from the 28S gene, as shown in Figure 1B.

We selected several genes that are targeted by multiple srRNAs for detailed analysis and observed that srRNA target sites often correspond to genomic regions containing fragments of diverged and heavily rearranged rDNA sequences. Figure 2 shows one example in the *UNC45B* gene, which specifies a co-chaperone required for folding and accumulation of type II myosins.

There are many small fragments similar to rDNA sequences in this region (Figure 2A), which mostly correspond to rearranged sequences of IGS (Figure S1). Only one 161-nt region corresponds to four blocks of 23–43-nt overlapping srRNAs from the main peak in the 28S gene (Figure 2B). The region demonstrates 96.32% identity with the 28S gene and is located in the antisense orientation inside the fourth intron of the *UNC45B* gene. This narrow region is characterized by the prominent H3K27ac mark in seven cell lines and by DNase I hypersensitive sites in 125 cell lines (Figure 2A). In H1-hESCs cells, there are active H3K27ac and H3K36me3 marks and the repressive chromatin mark H3K27me3 in this region. The reciprocal changes of H3K27ac and H3K27me3 marks were previously described in the promoter regions of endometrial cells [15]. Chromatin-state segmentation data indicate that the *UNC45B* gene is repressed in six cell lines. About 2.7 kb upstream from the region corresponding to srRNAs, there are CpG methylation marks. Similar results were obtained for several different srRNA target genes, but some genes were transcriptionally active and possessed CpG-methylated regions corresponding to srRNAs, e.g., *PMF1* (Figure S2). We observed that the targets of srRNAs are often methylated (Figure S2, Supplementary Text). Many of the same epigenetic marks (CpG methylation marks, H3K27ac, H3K27me3, H3K36me3, and CTCF marks, as well as DNase I hypersensitive sites) were observed in different combinations at srRNA targets in different genes, including *ANKRD30BL*, *RYR2*, *RELN*, *PID1*, and *HFM1* (Figures S4–S8).

Remarkably, the most abundant srRNAs targeting the *UNC45B* gene (Figure 2B) have no other gene targets in HEK293T cells (Table S1). There are further examples demonstrating unique targeting (Table S1). Figures S2 and S3 show the actively transcribed *PMF1* gene, which possesses stretches from the external transcribed spacer (ETS) regions that are targeted by srRNAs.

### 2.4. Epigenetic Features at srRNA Targets

**Figure 3:**
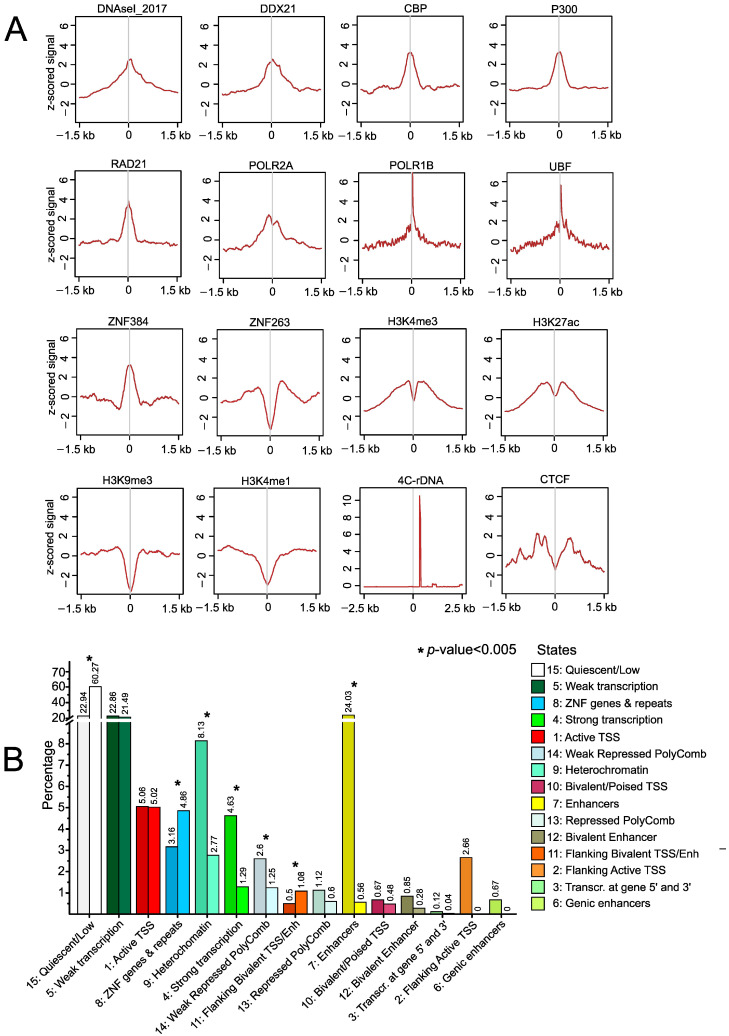
Properties of srRNA targets in HEK293T cells. (**A**) Profiles of DNase I sites, binding sites of different factors, histone marks, and rDNA-contacting sites around srRNA targets. The z-scored signals ±1.5 kb around srRNA targets are indicated. (**B**) The percentage of chromatin states (15-state model) in H1-derived neuronal progenitor-cultured cells in the whole genome (left dark bars) and at srRNA targets (right light bars) is shown. The color codes of the epigenetic states are shown in the order they appear at the srRNA sites. The labels present a state number and the percentage of the corresponding state. The statistical significance of the difference between epigenome states is tested with the independent-samples unequal-variances t-test. All cases with statistically significant differences (*p* < 0.005) are marked by an asterisk.

These results suggest a putative regulatory role of srRNAs, which prompted us to perform a genome-wide study of epigenetic marks and transcription factor-binding sites ±1.5 kb around the regions corresponding to srRNAs in HEK293T cells. We expected that there would be enrichment with active and repressive marks at these sites and we observed enrichment or depletion of several factors at the srRNA targets or immediately around them. srRNA target sites are enriched with DNase I hypersensitive sites, binding sites of DDX21 RNA helicase (a sensor of the transcriptional status of Pol I and Pol II RNA polymerases), and binding sites of the subunits of RNA Polymerase I and II (Figure 3A).

The data on the enrichment of srRNA targets by binding sites for CBP (the transcriptional coactivator of many transcription factors) and P300 histone acetyltransferase (acetylates core histones in nucleosomes and provides epigenetic tags for transcriptional activation) suggest that there are srRNA target genes that are activated by RNA-mediated mechanisms. ZNF384 and ZNF263—a modifier of the transcription of specific gene sets transcribed by RNA polymerase II and a transcriptional repressor, respectively—have contrasting profiles (Figure 3A). These data may indicate that srRNA target genes are not repressed by ZNF263 and may be regulated by ZNF384. There is enrichment of UBF and POLR1B at srRNA targets in the whole hg38 genome, which does not currently include rDNA genes (see Section 4). However, their role in these regions outside of rDNA clusters is not known.

Among the core histone modifications at targets of srRNAs, we observed depletion of the H3K9me3 mark, suggesting that srRNAs escape the constitutive heterochromatin regions. The target sites are also depleted of the H3K4me1 mark, which is characteristic of enhancers. Nevertheless, we observed some enrichment with active marks (H3K4me3 and H3K27ac, which are characteristic of promoters and super-enhancers) but only around ±300 bp of srRNA targets. These data suggest a connection between the activation of transcription and srRNA sites. Figure 1D demonstrates that about 10% of rDNA-contacting genes overlap with srRNA target genes. In our 4C-rDNA experiments [10], we used *EcoR*I and *Fae*I enzymes, which is why we expected that the 4C-rDNA profile would reflect the distance between the midpoints of srRNA targets and the midpoints of *EcoR*I–*Fae*I restriction fragments of about 200–500 bp in length. We observed that the peak of the 4C-rDNA profile was at some distance from the zero points in the srRNA targets in HEK293T cells (Figure 3A). The profile is shown in more detail in Figure S9. The data support the view that rDNA contacts might be RNA-mediated [10,11].

We also found a high enrichment of RAD21-binding sites directly at srRNA targets, as well as the presence of CTCF at about ±300 bp from the targets. RAD21 is a key component of the multiprotein cohesin complex. As cohesin and CTCF are involved in the formation of TADs and loop boundaries [16], we suggest that the observed genomic distribution of srRNA sites suggests the involvement of srRNAs in the organization of looped chromatin structures.

To further elucidate the epigenetic states that are characteristic of srRNA sites, we performed a search of the available human epigenome data based on the analysis of the core set of five chromatin marks (H3K4me3, H3K4me1, H3K36me3, H3K27me3, and H3K9me3) [17]. There are no corresponding data for the HEK293T cells, which originate from a human embryonic kidney yet have an unexpected relationship with neurons but not typical kidney epithelial cells. Therefore, we selected the available data for the H1-der (H1-derived neuronal progenitor cultured cells) human embryonic stem-cell line. Figure 3B shows the comparison of the epigenetic states at the srRNA sites and in the whole genome in this cell line. The srRNA target sites are enriched for ZNF genes and flanking bivalent TSS/Enh, as well as for genomic regions with a quiescent/low state, which are characterized by a chromatin structure largely devoid of the histone modifications included in the segmentation analysis [18]. In contrast, srRNA sites are depleted for heterochromatin, strongly transcribed and weakly repressed PolyComb regions, enhancers, flanking active TSS, and genic enhancers. The data on the depletion of H3K9me3 marks at srRNA targets (Figure 3A) are supported by the data on the reduced representation of heterochromatin states (Figure 3B).

### 2.5. Expression of srRNA Target Genes

**Figure 4:**
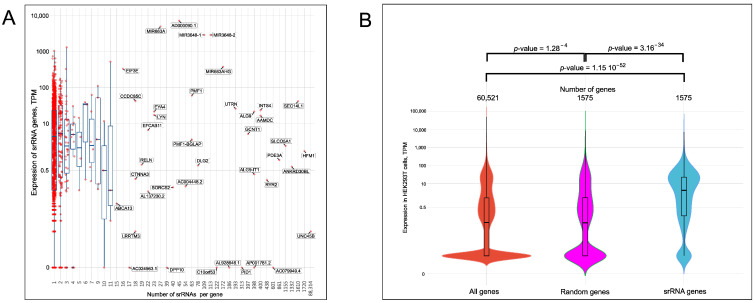
Analysis of expression of srRNA target genes. (**A**) The scatter plot presents the expression levels of 1584 srRNA target genes and the numbers of corresponding srRNAs. The red dots indicate srRNA target genes. The median position and whiskers are shown in blue. The *X*-axis is not to scale. The list of all srRNAs is shown in Table S1. The names of genes are indicated if a single gene is targeted by a particular set of isolated srRNAs (see Section 4). (**B**) Violin plots showing the distribution of genes with respect to their expression levels for all HEK293T genes (red), random genes (violet), and srRNA target genes (blue). The numbers of corresponding genes are shown at the top.

Taken together, the epigenetic profiling data suggest that srRNAs could be involved in both activation and repression of gene expression. Therefore, we next investigated the possible link between the numbers of srRNA per gene and the expression rate of the target genes in HEK293T cells. Although there are many factors controlling transcriptional and post-transcriptional gene expression in nuclei and cytoplasm, we attempted to find the putative link between expression levels of srRNA target genes and the abundance of the corresponding srRNAs. The plot of the RNA-Seq data and the numbers of srRNAs per gene (Figure 4A, Table S5) demonstrate that most srRNA target genes are targeted by one srRNA. Interestingly, the region in the plot with more than 11 srRNAs corresponds to single genes (the genes shown by dots without whiskers in Figure 4A), suggesting the high specificity of srRNAs. In this part of the plot, there are silenced and actively transcribed genes. The result suggests that srRNAs may be associated with both activation and repression of transcription.

For a better visualization of the relative proportions of active and repressed srRNA target genes, we used a violin plot to show the distribution of genes in relation to their expression levels. We observed that srRNA target genes have a larger proportion of actively transcribed genes than the bulk genes in HEK293T cells (Figure 4B). A set of 1575 randomly selected genes was also used for the comparison. The transcription pattern of the random genes shows a similar distribution to that of the full HEK293T gene set and differs from the transcription pattern of the srRNA target genes. Together with the data on the enrichment of srRNA sites with Pol II, CBP, P300, and active histone marks, as well as the depletion within heterochromatin regions (Figure 3), these findings demonstrate that srRNA targets mainly occur in the more actively transcribed genes.

## 3. Discussion

Almost exclusively, srRNAs originate from sense rRNA transcripts, including coding sequences and spacers (ETS, ITS1, ITS2, and 3′ ETS), which are present only in pre-rRNA molecules (Figure 1B). This suggests that the processing of pre-rRNA by the Microprocessor complex occurs in the nucleus. We assume that in the complex, DGCR8 recognizes the secondary structures in the pre-rRNA and Drosha cleaves in these regions, similar to the biogenesis of miRNAs. Figure S10 shows that the most abundant srRNA targeting the *UNC45B* gene corresponds to the stem-loop structure inside the 5′ end region of the 28S gene. We observed that IGS-homologous sequences often occur in various non-rRNA genes (Figures S1 and S3), but only a small number of srRNAs originate from the IGS (Figure 1B). The nature of antisense srRNAs derived from the IGS is still to be determined and we are currently studying the srRNAs detected in the enhancer sequences inside the IGS.

About 90% of srRNA targets are located in intergenic regions, inside introns, and at promoters, including unidirectional and bidirectional TSS, in all human chromosomes (Figures S11–S13). The origin of the rDNA sequences that are scattered across the human genome is not clear. One possible mechanism is translocation, because rDNA genes are the most fragile sites in the human genome and they shape frequent contacts with different genomic regions also possessing DSBs [19,20,21]. In the course of evolution, highly divergent and rearranged rDNA remnants could be selected as the mechanism of regulation of a set of genes associated with the activity of rDNA clusters. srRNAs coimmunoprecipitate with AGO proteins [6], which supports our conclusion that srRNAs may participate in the regulation of a particular set of genes. Further studies using different cell types are required to support our conclusion regarding the regulatory function of srRNAs. HEK293T is an aneuploid transformed cell line that possesses multiple chromosomal translocations, and the spectra of srRNAs in normal human cells are yet to be determined. Although transcripts from rDNA genes are the most abundant transcripts in various cell types, their processing into srRNAs could differ between tissues. It is known that cancer cells boost rDNA expression [22], which potentially could result in changes in the spectra of srRNAs. It will be of interest, therefore, to study srRNAs in other human cell types.

Changes in rRNA transcription are associated with differentiations in human, mouse, and *Drosophila* cells [11,19,23]. Our data support these observations and suggest one possible mechanism of gene regulation by rDNA-related small RNAs.

Recent studies of expression signatures suggest that the origin of HEK293 cells is from the adrenal gland adjacent to the kidney [24] and is associated with the sympathetic nervous system. The data on rDNA-contacting genes in HEK293T cells strongly indicate that this cell line is associated with the development of neurons [10]. The GO associations of srRNA target genes shown in Figure 1C–E support this conclusion. The role of small RNAs in transcriptional regulation has been discussed for a long time (for a review, see [3]). Small RNAs can target protein complexes to the complementary nascent transcripts, leading to the deposition of H3K9me2/3 repressive marks in the corresponding chromatin regions, or could directly recognize genomic regions subjected to methylation [25]. Our data argue in favor of a regulatory role for srRNAs in the expression of numerous genes throughout the human genome. However, direct experiments are required to test the capacity of srRNAs to induce the active or repressive epigenetic marks and to change expression levels of the srRNA target genes. In further studies, we will test the effects of transfected srRNAs and srRNA targets on activation or repression of particular genes.

## 4. Materials and Methods

### 4.1. Isolation of srRNAs

The HEK293T small-RNA NGS dataset was obtained from GEO accession GSM955512/SRR518497 (37,065,975 reads). The dataset was processed by Trimmomatic [26] 0.36 to remove reads shorter than 20 bp, to remove low-quality ends, and to sustain acceptable read quality throughout all read lengths (options: LEADING:18 TRAILING:18 SLIDINGWINDOW:4:22 MINLEN:20, 36,625,832 reads left). The next step was to obtain a deduplicated dataset in which all complete copies of reads were removed so only unique reads remained. This was achieved by dedupe.sh from BBtools [27] 38.62 with options to remove only exact copies and to remove low-quality ends (ac = f qtrim = rl trimq = 18, 3, 931,671 unique reads left). All further processing was completed in parallel for both datasets with all reads (36,625,832) and with unique reads (3,931,671) only.

To separate srRNA reads from all small RNA data, we aligned the dataset to the rDNA sequence (Genbank accession U13369) by bowtie2 [28] 2.3.4.1 with preset --end-to-end --very-sensitive to find the maximum possible amount of rDNA-aligned sequences. All unaligned sequences were removed from the alignment file (--no-unal option), and the file was sorted by coordinate (samtools sort [29]) and converted to the BAM format (568 364 aligned reads). The initial reads that aligned to rDNA were recovered from this BAM file by the bedtools [30] 2.29.1 bamToFastq tool. Then, srRNA-associated reads were aligned to the GRCh38/hg38 p.12 human genome by bowtie2 [28] with preset --end-to-end --sensitive, all unaligned reads were removed from the alignment file (--no-unal), and the alignment file was sorted by coordinate (samtools sort [29]) and then converted simultaneously both to the resulting table (with genome coordinates, number of reads, coverage, and sequence per mapping) by ad hoc in-house bash and Perl scripts and to the genome-wide srRNA profile by genomeCoverageBed [30] and bedGraphToBigWig [31] tools.

The resulting table was converted to GFF format for further processing by the Perl script. The mapping areas from the resulting table were assigned to genes by the following procedure. Ensembl genome annotation GRCh38/hg38 p.12 v.97 was used to obtain the list of *H. sapiens* genes. The gene names, IDs, and chromosome coordinates were extracted from the GTF file by the R script with the help of refGenome and dplyr libraries. The intersectBed [30] tool was applied to find intersections between the srRNA mappings file and the *H. sapiens* genes list. Thus, the list of srRNA target genes was generated. The complete bioinformatic flowchart is shown in Figure S15.

The srRNA profile along the rDNA was generated from the aligned srRNAs to rDNA BAM file by the genomeCoverageBed [30] tool. Alignment BAM files are available at the following link: http://epigen.eimb.ru/IJMS2022/ (accessed on 8 March 2022).

### 4.2. Genome-Wide Profiles

The following genome-wide HEK293 profiles were downloaded from the ENCODE project (see Table S6): CTCF (ENCSR000DTW/ENCFF924LOC), DNAseI (HEK293T) (ENCSR000EJR/ENCFF716SFD), H3K4me1 (ENCSR000FCG/ENCFF717JWL), H3K4me3 (ENCSR000DTU/ENCFF756EHF), H3K36me3 (ENCSR910LIE/ENCFF704SBO), H3K27ac (ENCSR000FCH/ENCFF631VZK), ZNF263 (ENCSR000EVD/ENCFF367HGG), H3K9me3 (ENCSR000FCJ/ENCFF902RQI), and ZNF384 (HEK293T) (ENCSR882ICT/ENCFF128ERM).

The following HEK293 data were downloaded from NCBI GEO/SRA database: DDX21 (SRR1910478/SRR1910479), CBP (SRR1001897, SRR1001898/SRR1001900), p300 (SRR1001893, SRR1001894/SRR1001900), RAD21 (HEK293T) (SRR710096/SRR710097), POLR2A (GSM935534/GSM935533), RPA116/POLR1B (HEK293T) (SRR087747/SRR087753), and UBF (HEK293T) (SRR087746/SRR087753).

The data were processed uniformly by the following pipeline. In the first step, all short and/or low-quality reads were removed, and low-quality ends were trimmed by Trimmomatic [26] (options: LEADING:18 TRAILING:18 SLIDINGWINDOW:4:22 MINLEN:20). In the second step, all data were aligned to the hg38 genome by bowtie [32] 1.2.3 with options --best --strata -m 1. The resulting SAM files were converted to BAM files and sorted, and unaligned reads were removed. Samtools [29] fixmate/markdup tools were used to find and mark complete duplicates that were ignored in further processing. MACS2 [33] 2.1.2 was applied as a peak caller (options --bdg --gsize hs --call-summits) and later to build fold-enrichment profiles (options bdgcmp -m FE). Resulting fold-enrichment profiles were converted to bigWig format by the bedGraphToBigWig tool [31].

An HEK293T 4C-rDNA-contacting regions genome-wide profile was created in the following way. The HEK293T line was provided by Dr. V. S. Prassolov (Engelhardt Institute of Molecular Biology). Raw data for HEK293T cells were downloaded from GEO GSM3434713 and GSM3434714, and adapters were removed according to the description in the GEO. The filtered replicas were aligned to the GRCh38/hg38 p.12 human genome that did not include rDNA clusters by the bwa [34] 0.7.17-r1188 mem algorithm. Unaligned reads were removed and alignment files were sorted and converted to BAM format by samtools [29]. BAM files were converted to bedGraph profiles by the genomeCoverageBed [30] tool. The subtractBed [30] tool was used to subtract mappings that were mapped completely to low complexity and/or repeat regions that were present in the DFAM [35] database from the profiles. A mean profile was created by WiggleTools [36] and converted to bigWig format by the bedGraphToBigWig [31] tool. All the epigenetic plots were created interactively by the SeqPlots [37] package.

Profile plots were created at 10 bp binning size with mean values from the z-score-normalized (in the plot range) data and the midpoints of the srRNA genome-wide mappings applied as the plot center. Z-score normalization was performed by SeqPlot’s built-in function. srRNA input data for profiles were processed according to the procedure described in Methods for each genome strand separately (options --norc and --nofw for bowtie2) and then united into a single GFF file with strand information.

### 4.3. Epigenome Statistics

Epigenome chromatin-state statistics were calculated for the core 15-state model (five marks) for the data that were downloaded from the “NIH Roadmap Epigenomics” [38] for the H1-derived neuronal progenitor cells (E007 epigenome) as the closest cell type to HEK293T cells from the Epigenome atlas. Intersections between srRNA mappings and chromatin states were found by intersectBed [30] tools and statistics were calculated by an in-house Perl script. Donut charts were created by the R script with the help of ggplot2 and ggrepel R libraries.

### 4.4. RNA-Seq Analysis

We performed HEK293T expression analysis using both iTPM values for each experiment and as a raw-value matrix for the differential RNA-Seq (as required by DESeq2). HEK293T RNA-Seq data (two replicates, GSE130262) were used. Figure S14 shows the consistency between the RNA-Seq replicates. All RNA-Seq data were processed uniformly. Trimmomatic [26] was applied to remove low-quality reads with the following options: LEADING:18 TRAILING:18 SLIDINGWINDOW:4:22 MINLEN:20. The filtered reads were aligned to the GRCh38/hg38 genome with Ensembl v.97 annotation using the STAR RNA-Seq aligner 2.6.1c [39]. The package featureCounts [40] 1.5.1 was applied to quantify alignments to the GRCh38/hg38 Ensemble v.97 list of genes with the options: -a hg38.97.gtf -t exon -g gene_id *.bam. Next, the list of quantified genes was filtered using the list of srRNA target genes. For further analysis, we excluded 13 genes corresponding to the rDNA gene family (RNA5-8SN1, RNA5-8SN3, RNA5-8SN2, FP671120.4, FP671120.2, FP236383.3, FP236383.1, FP236383.2, RNA5-8SP6, FP671120.1, RF00002, RNA5-8SP2, AC010970.1).

To obtain accurate transcript quantification from the RNA-Seq data, the RSEM [41] software package was applied. The resulting gene tables were combined and 13 genes corresponding to the rDNA family of genes were also excluded. Gene expression values (in TPM) were assigned to the previously obtained srRNA mappings and were used to create violin plots and scatterplots with box-and-whiskers plots. All charts were created by R scripts with the help of the ggplot2 library.

### 4.5. Transcription Start-Site Analysis

Transcription start sites (TSSs) were obtained from the NCBI RefSeq Curated [42] database as follows. All genes from the ncbiRefSeqCurated table for the hg38 genome were downloaded from the genome-mysql.cse.ucsc.edu server by SQL request. In the next step, all complete gene duplicates were removed, and then the list of genes was converted to the TSS list. We selected only those TSSs that are expressed in HEK293T cells by filtering the TSS list according to CAGE/Phantom5 [43] genome-wide expression data (downloaded from EPD [43] server ftp://ccg.epfl.ch/mga/hg38/fantom5/) (accessed on 8 March 2022). In the case of multiple gene TSSs, the minor TSSs that had an expression level of less than 1% of the major TSS were excluded from further consideration. The list was then divided into the list of bidirectional promoter TSSs (i.e., the distance between TSSs should be less than 1000 bp, the TSSs should be located on the opposite strands, and transcription from these TSSs should not intersect) and the list of unidirectional promoter TSSs. The lists were converted to GFF format and srRNA profiles around bidirectional and unidirectional promoter TSSs were created by the SeqPlots [37] package.

### 4.6. Permutation Analysis

To ensure the specificity of the srRNA mapping, we performed a permutation test in the following way. A Perl *in hoc* program was developed to shuffle FastQ records in the source SRR518497 file. This program employs BioPerl’s Seq::Quality module for input/output and the Mersenne Twister pseudorandom generator [44] Math::Random::MT::Auto, and implements Durstenfeld’s version of the Fisher–Yates shuffling algorithm [45] for sequence letter/quality value pairs (so sequence letters are shuffled together with their quality values). This approach ensures that the GC content and amounts of all letters in the sequence remain the same while their order becomes random. Coupling quality values with letters ensures that all sequence letters keep their quality values. The permuted dataset was processed according to the section “Isolation of srRNAs” in Section 4. The intersections between the resulting mapping tables of nonpermuted and permuted datasets were found by the intersectBed tool. The intersections of the gene lists were calculated by a Unix shell one-liner: intersectBed-a srRNA_table.txt-b permuted_table.txt|cut-f 12|sort|uniq|wc–l Permutation and mapping procedures were performed ten times. The mean number of mapped reads was 96 ± 3.8 (in the non-permuted dataset the number of mapped reads equals 555,385). The mean number of mapped regions was equal to 96 ± 3.8 (in the nonpermuted dataset, this number is equal to 2961). The number of intersected nonpermuted and permuted mapped regions did not exceed eight. We performed Jaccard intersection tests using the bedtools jaccard tool. The mean value of Jaccard statistics was 0.00067 ± 0.00035, while the maximum observed value 2qs 0.00115. These data mean that the intersection by the length of permuted and non-permuted dataset mappings was statistically negligible (<0.115%). We performed correlation tests between nonpermuted and permuted mapped regions by AnCorr [46]. The mean value of |z| = 1.032 ± 0.397, *p* = 0.3345 ± 0.171, and the maximum value z = 1.762, *p* = 0.078, which means that there was no statistically significant correlation observed in any test between permuted and nonpermuted dataset mappings. The mapping tables obtained from nonpermuted and permuted datasets and associated with genes intersected in 2.70 ± 1.57 genes (the median number of overlapping genes was two and the maximum number of overlapping genes was six). We tested the probability of obtaining these numbers of overlapping genes by chance using the hypergeometric test *p* = phyper (2, 1575, 60522-1575, 67, lower.tail = FALSE) = 0.2534 (*p* > 0.05). Therefore, intersections in gene lists that were obtained from the permuted datasets with the srRNA-associated gene list could be obtained by chance and should be ignored.

Thus, we can conclude that the srRNA mappings (SRR518497 accession) to the human rDNA and genome-wide to the human hg38.p12 genome build are robust to the shuffling permutations. Therefore, the results based upon these mappings could not be obtained by chance and are nonrandom.

### 4.7. Analysis of the Distribution of Genes versus Their Expression Levels by Violin Plots

Gene expression datasets do not follow a normal distribution [47] and, therefore, nonparametric statistical criteria should be used for their analysis. We tested the nonparametric independent two-group Mann–Whitney U-test applicability for this task. We performed the following tests.

1. Test with subsets of the same size—Two gene subsets of equal size were created, and the gene expression values were shuffled randomly using Durstenfeld’s version of the Fisher–Yates shuffling algorithm [45]. Appropriate amounts of values were selected from the shuffled lists. The Mann–Whitney U-test was applied to test if these two randomly selected subsets originated from the same distribution. The procedure was performed 10,000 times. FDR = 0.0483 for subsets containing 1575 genes.
2. Test with the full gene set and a smaller subset—A subset was created by randomly shuffling all gene expression values by Durstenfeld’s version of the Fisher–Yates shuffling algorithm and then selecting 1575 appropriate values from the shuffled list. The Mann–Whitney U-test was applied to test if the full expression set and created subset originate from the same distribution. The procedure was performed 100,000 times. FDR = 0.0453 for the subset with 1575 genes.

In all cases, the FDR values corresponded to the theoretical value 0.05 ± 0.005 and so the Mann–Whitney U-test can be applied to the gene expression datasets.

We applied the Mann–Whitney U-test to detect whether the srRNA-associated gene expression subset and the full expression dataset originate from the same distribution. The independent two-group Mann–Whitney U-test between the complete gene expression set and the srRNA-associated gene expression subset yielded a *p*-value of 1.152 × 10^−52^.

We also tested whether the difference in expression distributions could be obtained by chance in the case of equally sized datasets by Monte Carlo (MC) simulations. The test was performed using the same design as the tests in test 1, above, except that the first gene set was the srRNA-associated gene expression dataset (1575 unique genes) and the second expression dataset was the expression set of the same number (1575) of randomly selected genes. The test was repeated 10,000 times. In all cases, the results were negative, i.e., the srRNA-associated gene expression dataset and the randomly picked gene datasets do not originate from the same distribution and are independent. Maximum observed *p*-value = 1.493 × 10^−11^, i.e., <<0.01. We can conclude that at the level of *p* = 0.0001, the expression of the srRNA-associated genes dataset cannot be obtained from the gene expression dataset by chance.

Thus, we can conclude that the srRNA-associated gene expression dataset is significantly different from the full expression set.

The middle violin plot in Figure 4B represents the dataset that was obtained by averaging 10,000 randomly selected datasets from the full dataset by shuffling 1575 gene dataset expression values, which were sorted and then summed up to the storage array. After 10,000 MC trials, all members of the storage array were divided by 10,000. The averaged random dataset was created for presentation purposes only and the main conclusion about the independence of the full expression dataset and the srRNA-associated genes dataset is made on the basis of the statistical tests.

### 4.8. Availability of Data Sources and Applied Scripts

The sources and accession numbers of all data mentioned in the paper are collected in Table S6. Figure S15 illustrates the bioinformatics pipeline flowcharts. All scripts that were developed and applied for this article are deposited in the public Github repository: https://github.com/lokapal/IJMS2022.srRNA (accessed on 8 March 2022).

## Data Availability

Not applicable.

## Supplementary Materials

The following supporting information can be downloaded at: https://www.mdpi.com/article/10.3390/ijms23063014/s1. Reference [48] is cited in the supplementary materials.
