# Supplementary material for: Fragments of rDNA Genes Scattered over the Human Genome Are Targets of Small RNAs
Source: Int J Mol Sci. 2022 Mar 10;23(6):3014. doi: 10.3390/ijms23063014 (PMC8954558; doi:10.3390/ijms23063014)
Supplement: Supplementary file 1 [file ijms-23-03014-s001.zip › ijms-1604534-supplementary/ijms-1604534-supplementary.pdf]

International Journal of *Molecular Sciences*

MDPI

Supplemental Information for:

Fragments of rDNA genes scattered over the human genome are targets of small RNAs

Nickolai A. Tchurikov[1], Elena S. Klushevskaya[1], Ildar R. Alembekov[1], Anastasiia S. Bukreeva[1], Yulia A. Polunina[1], Valeriia D. Khazieva[1], Vladimir R. Chechetkin[1], Galina I. Kravatskaya[1] and Yuri V. Kravatsky[1]

[1] Department of Epigenetic Mechanisms of Gene Expression Regulation, Engelhardt Institute of Molecular Biology Russian Academy of Sciences, Moscow, 119334, Russia

Figures S1–S15

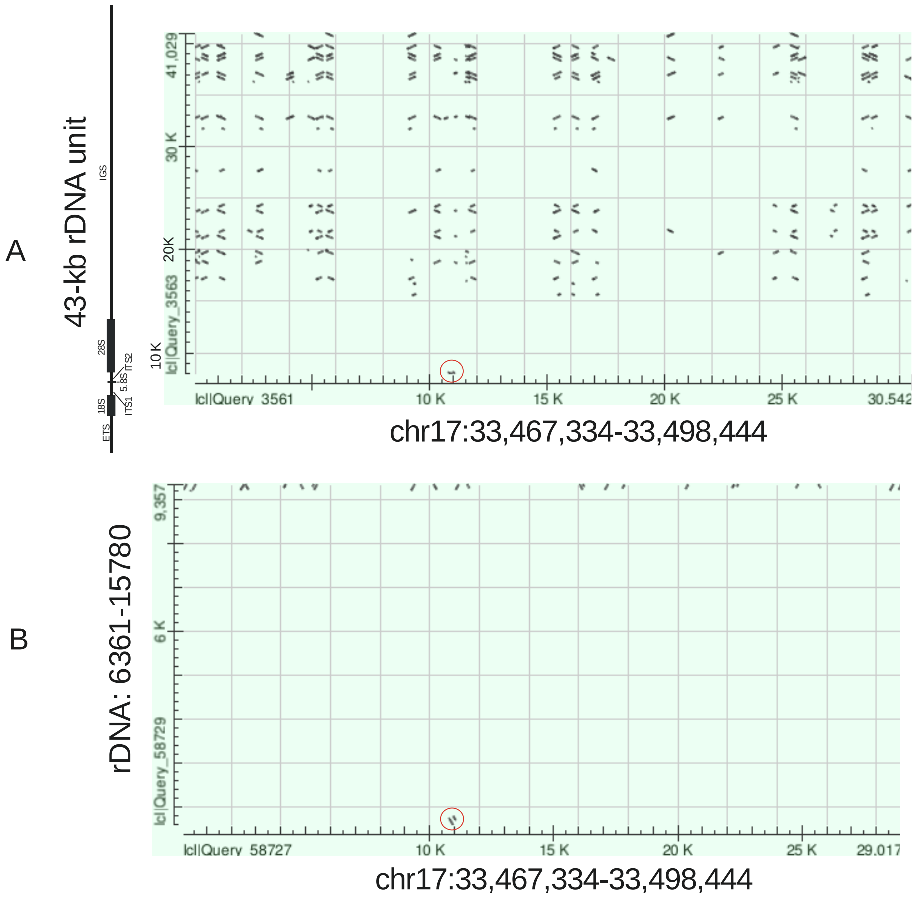

Figure S1. Dot plot showing homologous regions between the rDNA unit and a region of chr17 containing the *UNC45B* gene. (**A**) The complete 43-kb rDNA was used. The numbering in rDNA is according to the human ribosomal DNA complete repeating unit sequence (accession number: U13369). The red circle indicates the only homologous region from the transcribed portion of rDNA (7935–8146) corresponding to the 5′ end of the 28S gene. (**B**) A fragment of rDNA sequence (6361–15780) and the same region of chr17 were used for dot plot to show the homology regions inside 28S RNA gene in more detail.

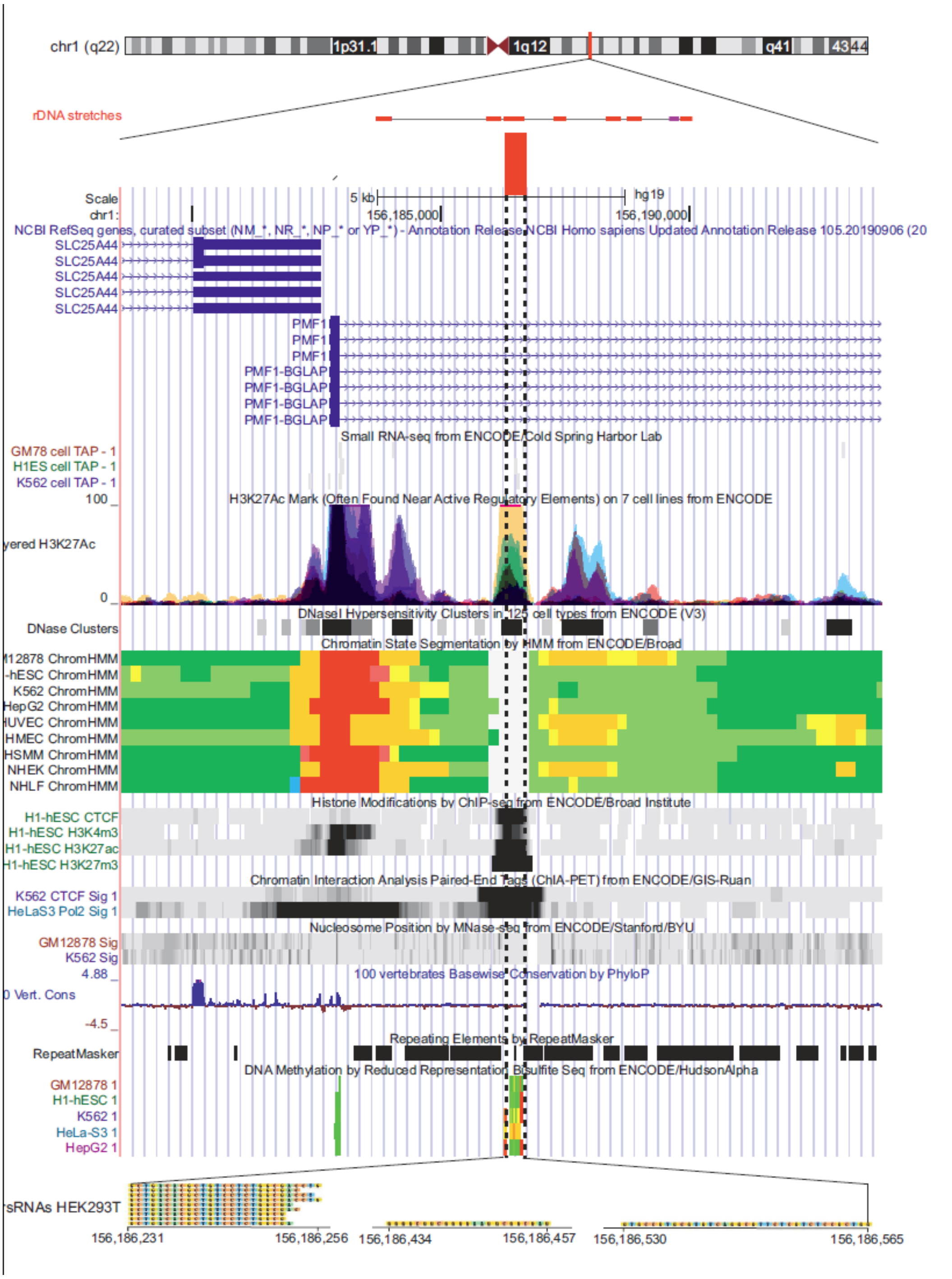

Figure S2. Characterization of the srRNA targets inside the intron of the *PMF1* gene. The distribution of layered H3K27ac marks, genome segmentation from ENCODE, histone modifications, nucleosome position, and CpG methylation inside a region of chr1 are shown as in the UCSC Browser. Sequences of 23–35-nt-long srRNAs from the ETS portion of the rDNA unit (see Fig. S3) are shown at the bottom.

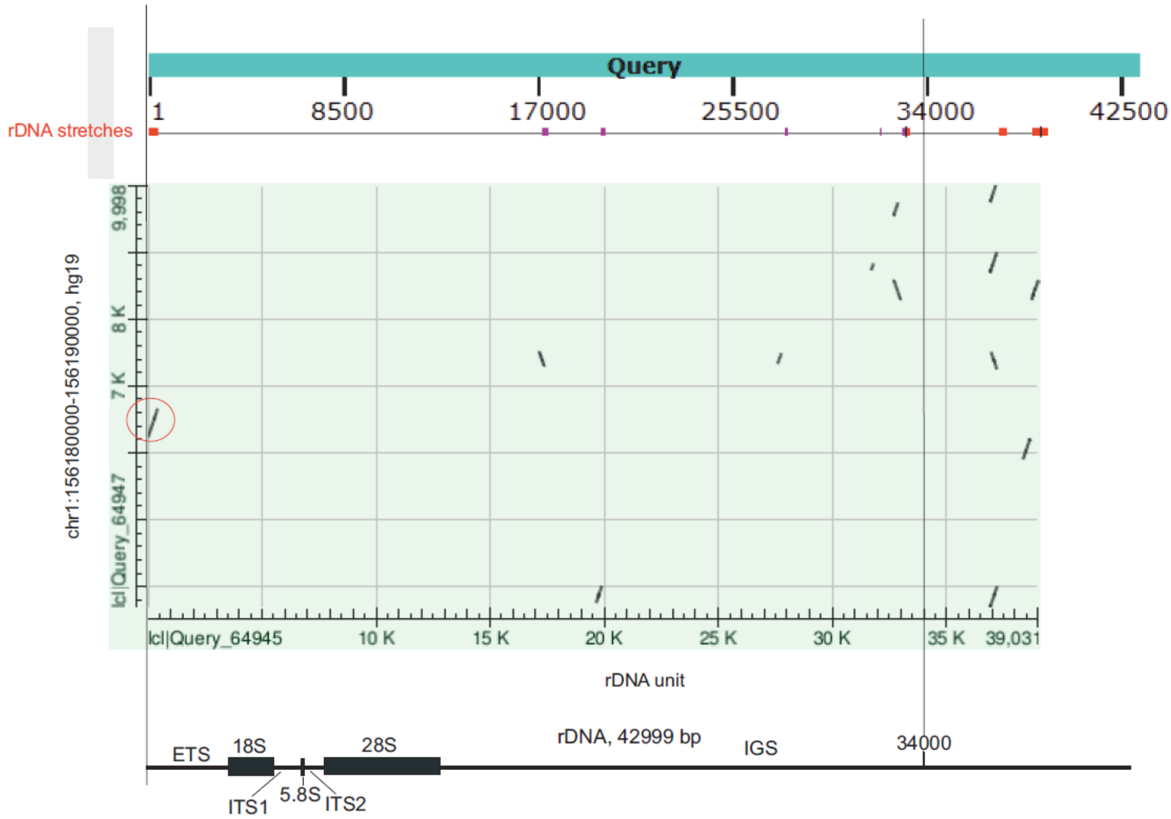

Figure S3. Dot plot showing homologous regions between the rDNA unit and the region of chr1 containing the *PMF1* gene. The numbering in rDNA is according to the human ribosomal DNA complete repeating unit sequence (accession number: U13369). The red circle on the Y-axis indicates the only homologous region from the transcribed portion of rDNA (1–331) corresponding to the very beginning of the ETS sequence. rDNA stretches are shown at the top.

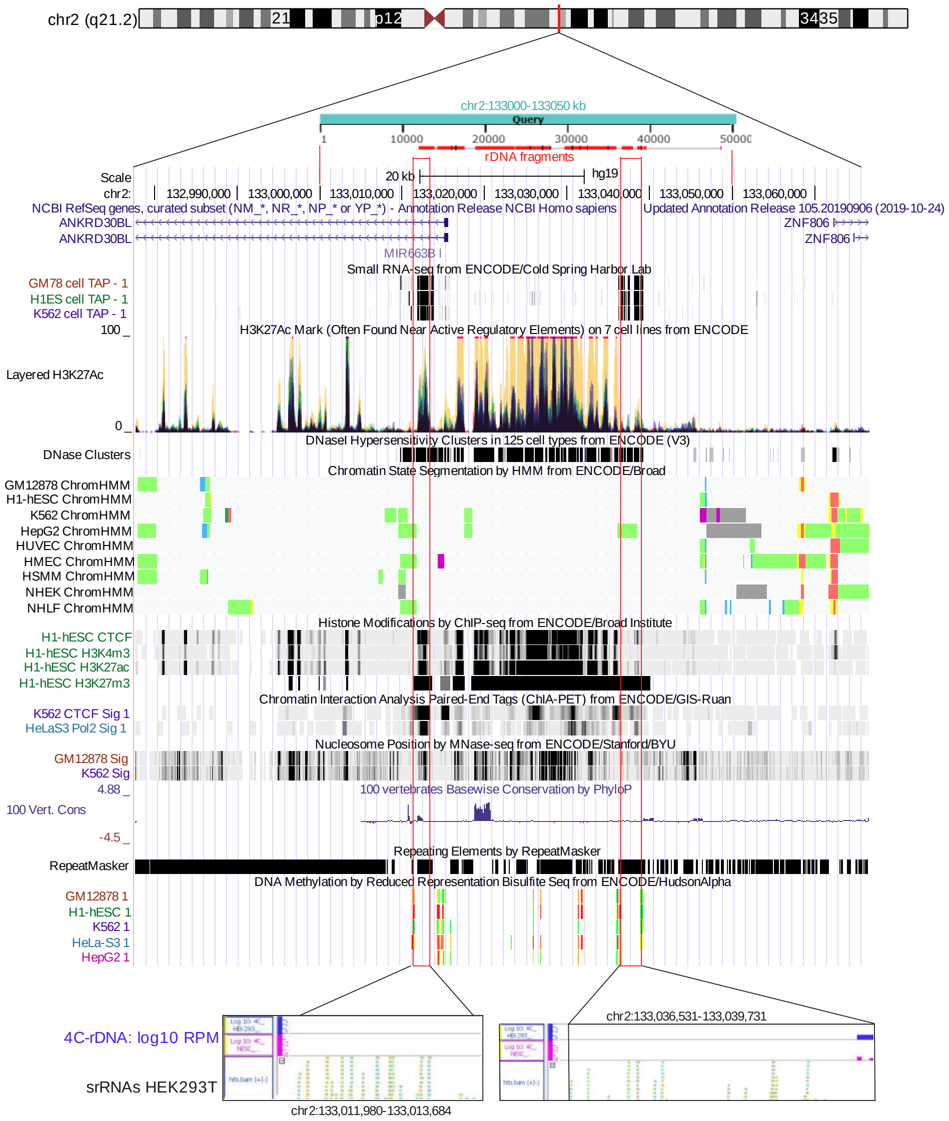

Figure S4. Characterization of the srRNA targets inside the intron of the *ANKRD30BL* gene and in the upstream region. The alignment of the genomic region with the rDNA unit is shown at the top. The distribution of layered H3K27ac marks, genome segmentation from ENCODE, histone modifications, nucleosome position, and CpG methylation inside a region of chr1 are shown as in the UCSC Browser. The distribution of 23–35-nt-long srRNAs is shown schematically at the bottom.

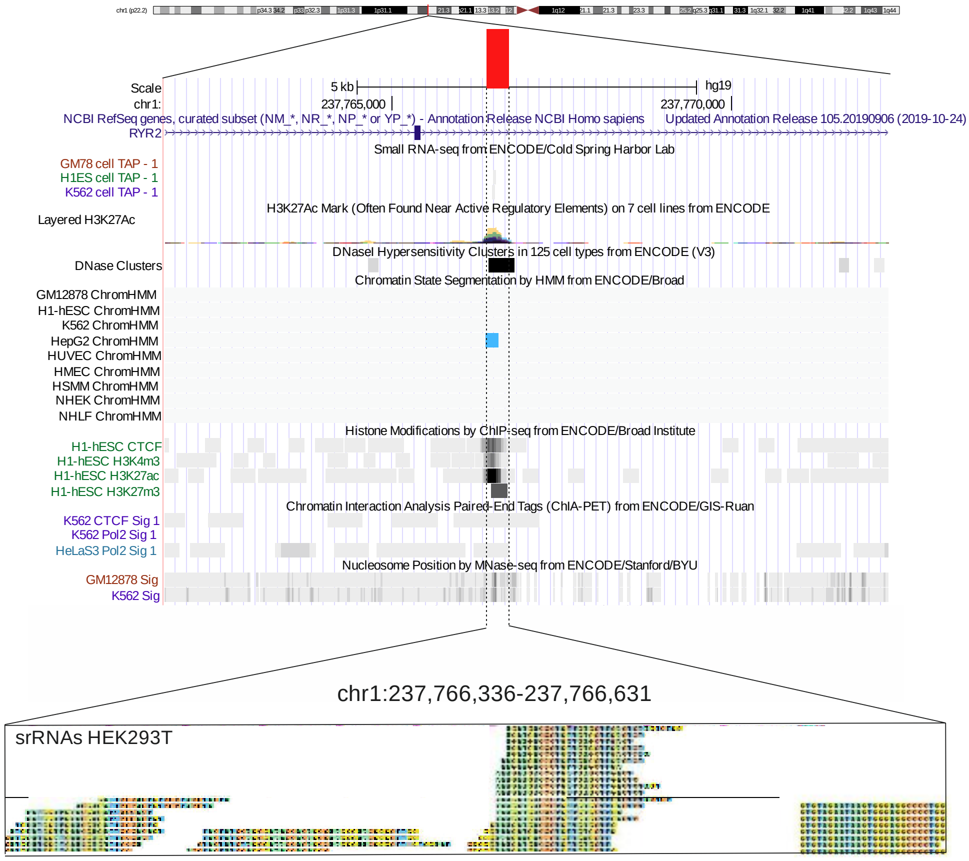

Figure S5. Characterization of the srRNA targets inside the intron of the *RYR2* gene. The red bar at the top shows a region corresponding to srRNAs. The distribution of layered H3K27ac marks, genome segmentation from ENCODE, histone modifications, nucleosome position, and CpG methylation inside a region of chr1 are shown as in the UCSC Browser. Sequences of srRNAs are shown at the bottom.

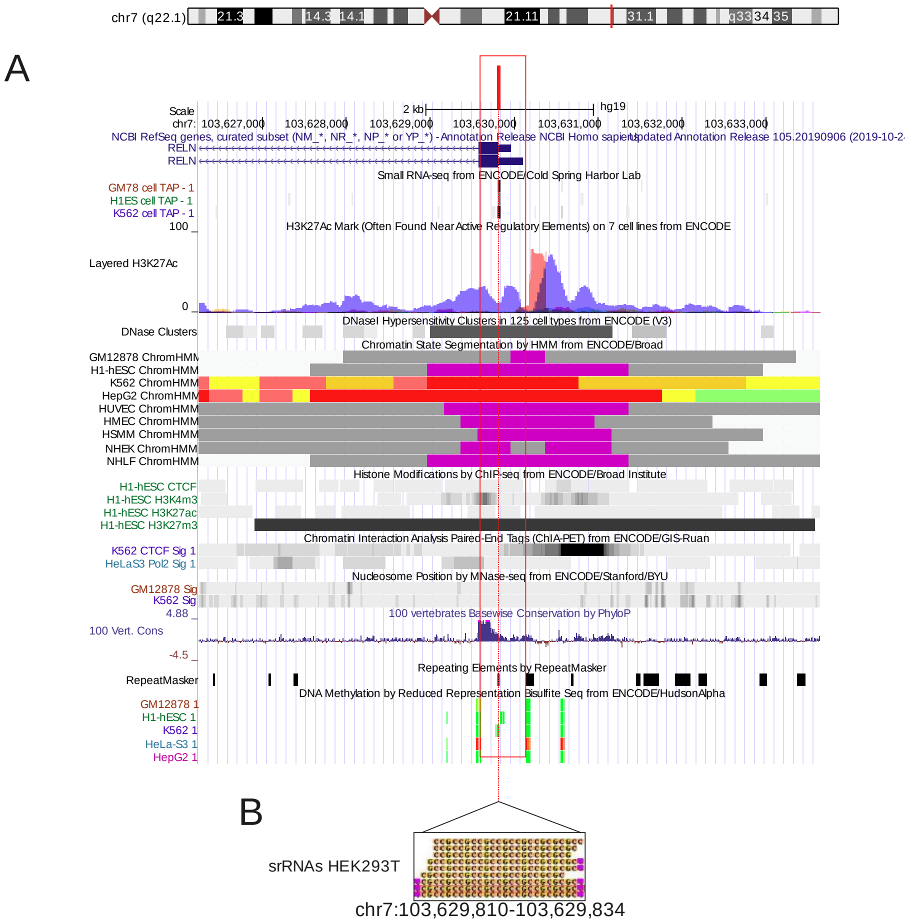

Figure S6. Characterization of the srRNA targets inside of the *RELN* gene. The red bar at the top shows the region corresponding to srRNAs. (**A**) The distribution of layered H3K27ac marks, genome segmentation from ENCODE, histone modifications, nucleosome position, and CpG methylation inside a region of chr1 are shown as in the UCSC Browser. (**B**) Sequences of srRNAs are shown at the bottom.

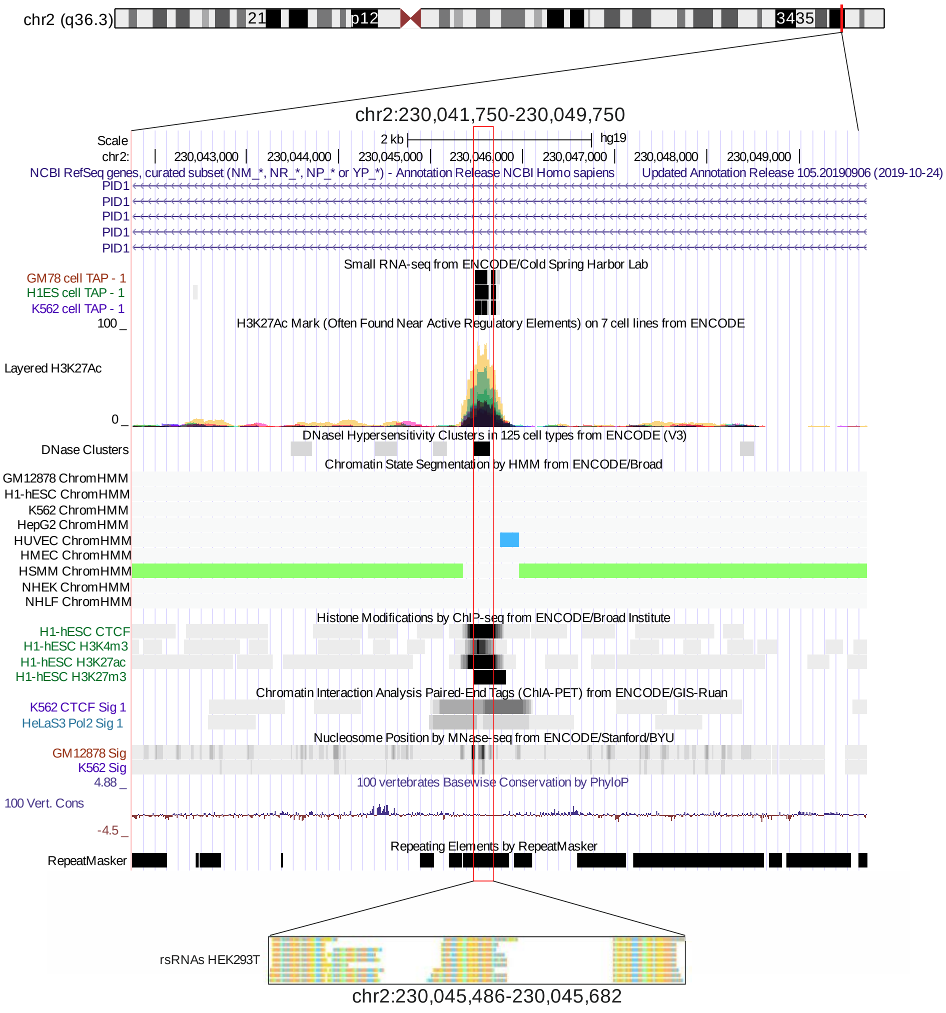

Figure S7. Characterization of the srRNA targets inside the intron of the *PID1* gene. The red frame shows the region corresponding to srRNAs. The distribution of layered H3K27ac marks, genome segmentation from ENCODE, histone modifications, nucleosome position, and CpG methylation inside a region of chr1 are shown as in the UCSC Browser. Sequences of srRNAs are shown at the bottom.

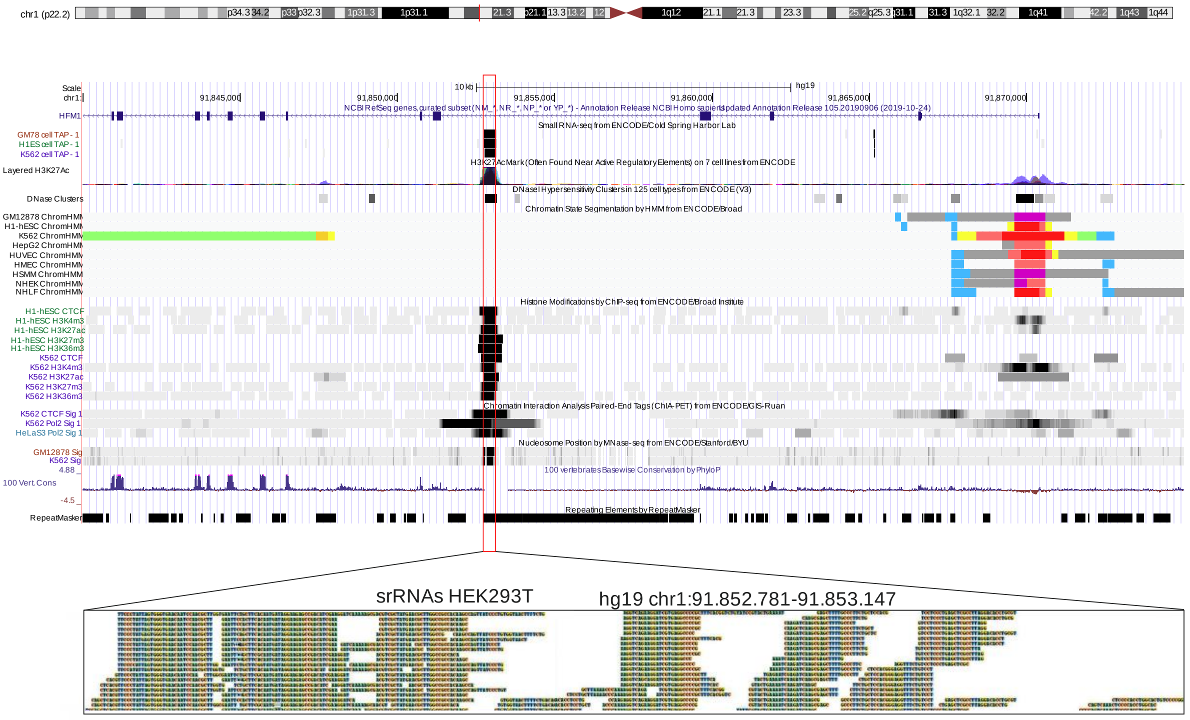

Figure S8. Characterization of the srRNA targets inside the intron of the *HFM1* gene. The red frame shows the region corresponding to srRNAs. The distribution of layered H3K27ac marks, genome segmentation from ENCODE, histone modifications, nucleosome position, and CpG methylation inside a region of chr1 are shown as in the UCSC Browser. Sequences of srRNAs are shown at the bottom.

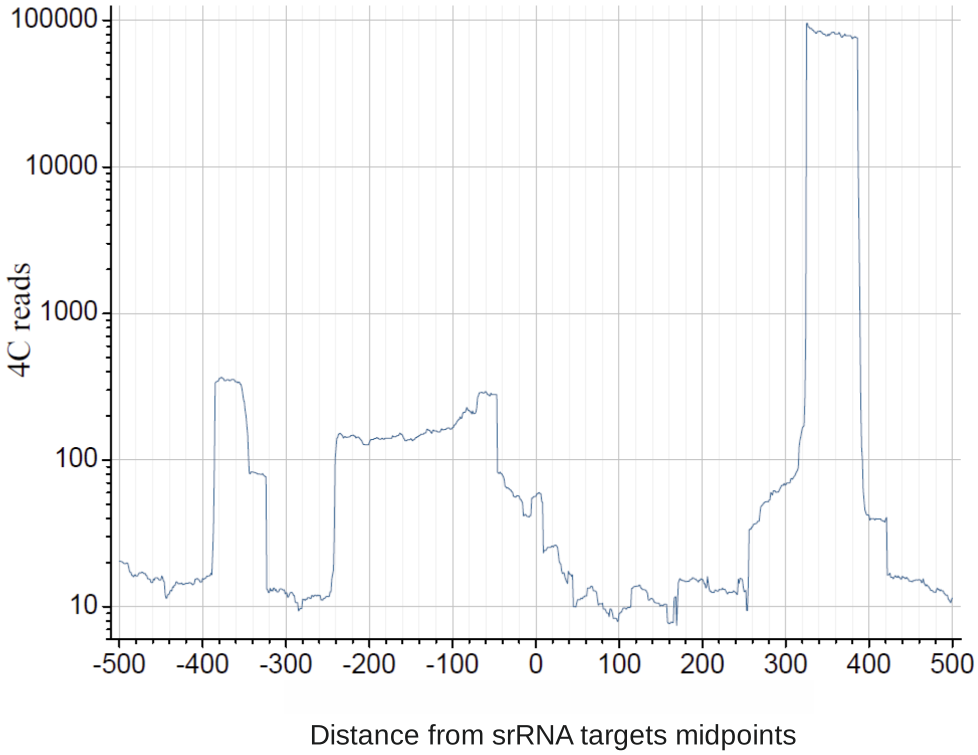

Figure S9. Profile of 4C-rDNAcontacts around srRNA targets in the hg38 genome. The number of mapped rDNA contacts (log10 scale) are shown around srRNA target midpoints.

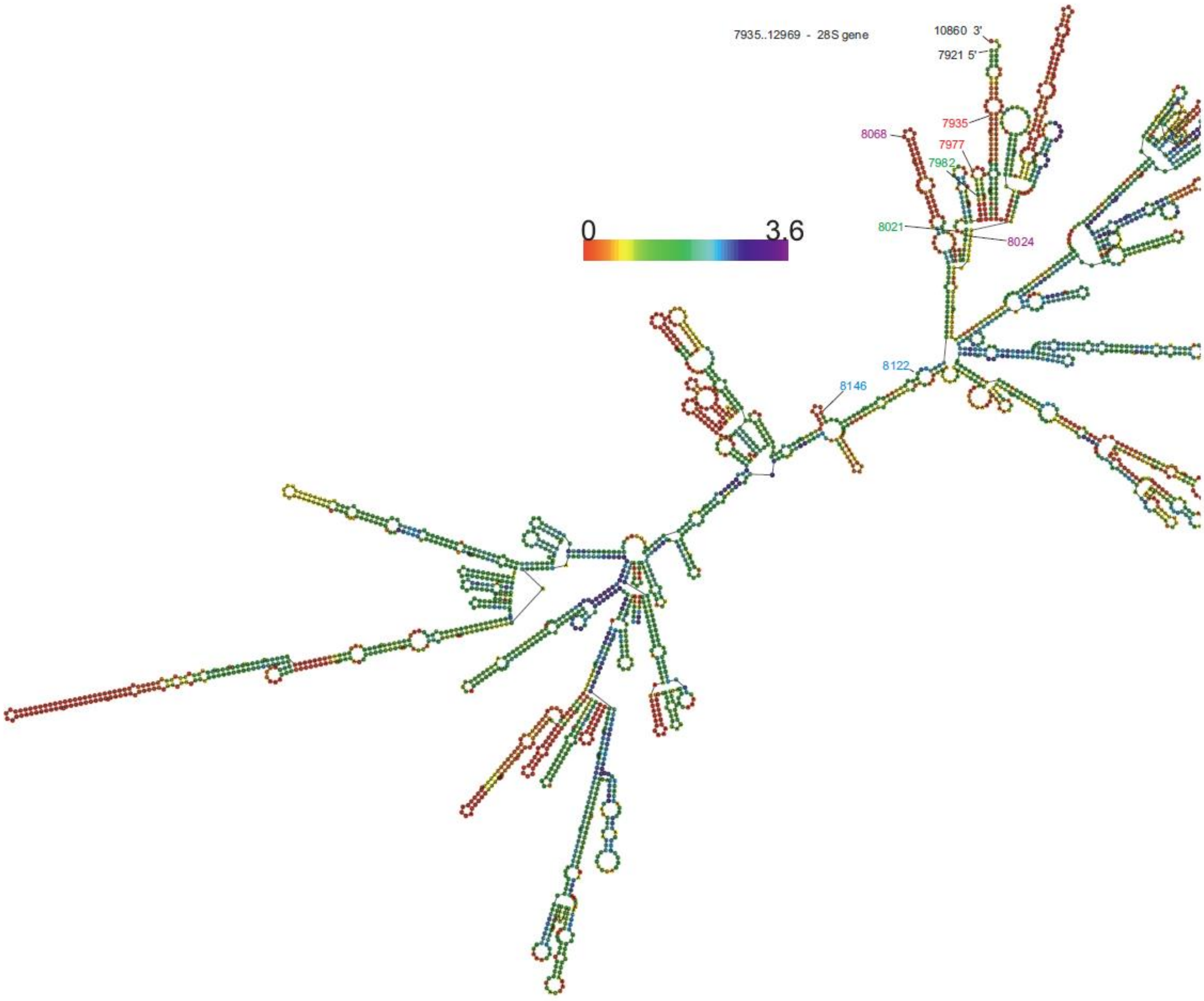

Figure S10. Example of the formation of stem-loop structures in the 28S rRNA fragment. The numbering in rDNA is according to the human ribosomal DNA complete repeating unit sequence (accession number: U13369). The most prominent srRNA is formed from the 5′ end of the 28S gene with coordinates 7935–7977 (shown in red; see Fig. 2B). Other stem-loop structures shown in Fig. 2B with coordinates 8024–8068, 7982–8021, and 8122–8146 are shown by the values in violet, green, and blue, respectively. The search of RNA secondary structures was performed using the RNAfold Web server (http://rna.tbi.univie.ac.at).

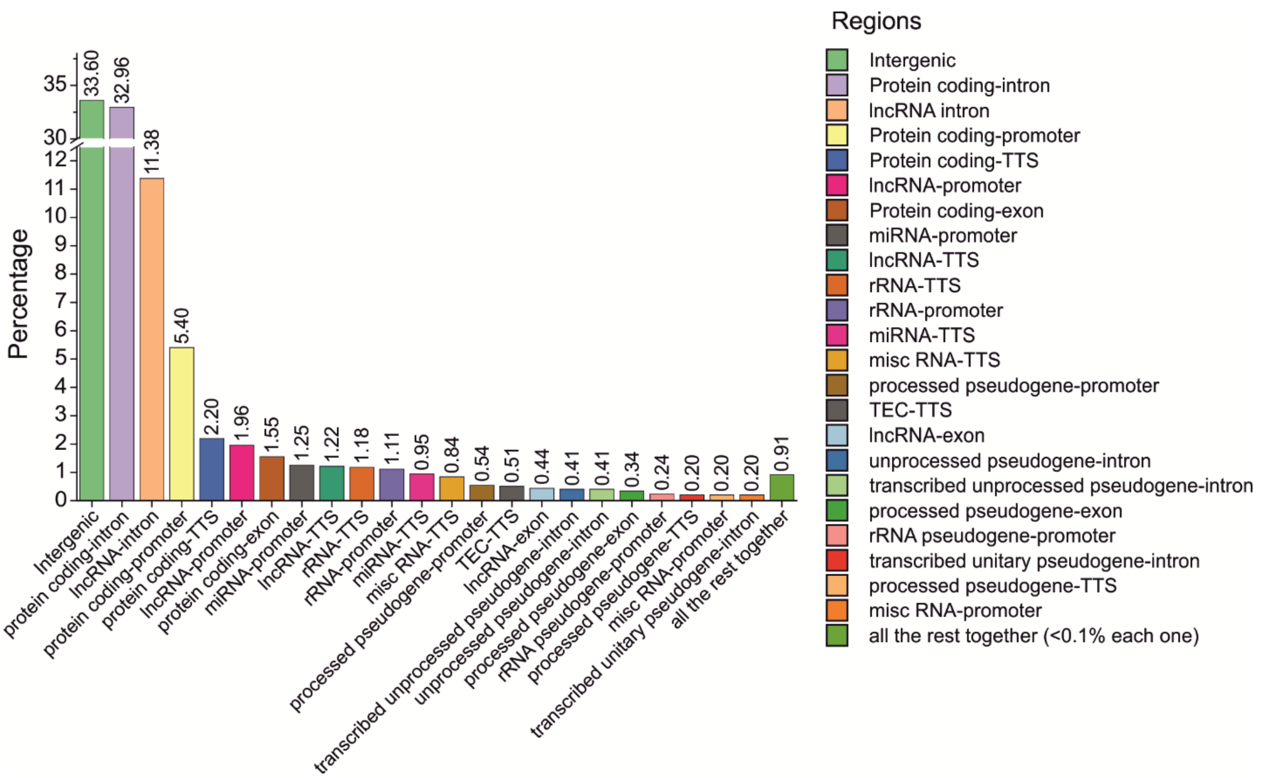

Figure S11. Distribution of srRNA target sites in different portions of the human genome. The values in the labels present a percentage of the corresponding portion. srRNA mappings were annotated to the genome by annotatePeaks.pl tool from Homer software package. EMBL annotation hg38 v.97 was obtained from Ensembl-EBI FTP server (ftp://ftp.ensembl.org/pub/release-97/gtf/homo_sapiens/Homo_sapiens.GRCh38.97.chr.gtf.gz).

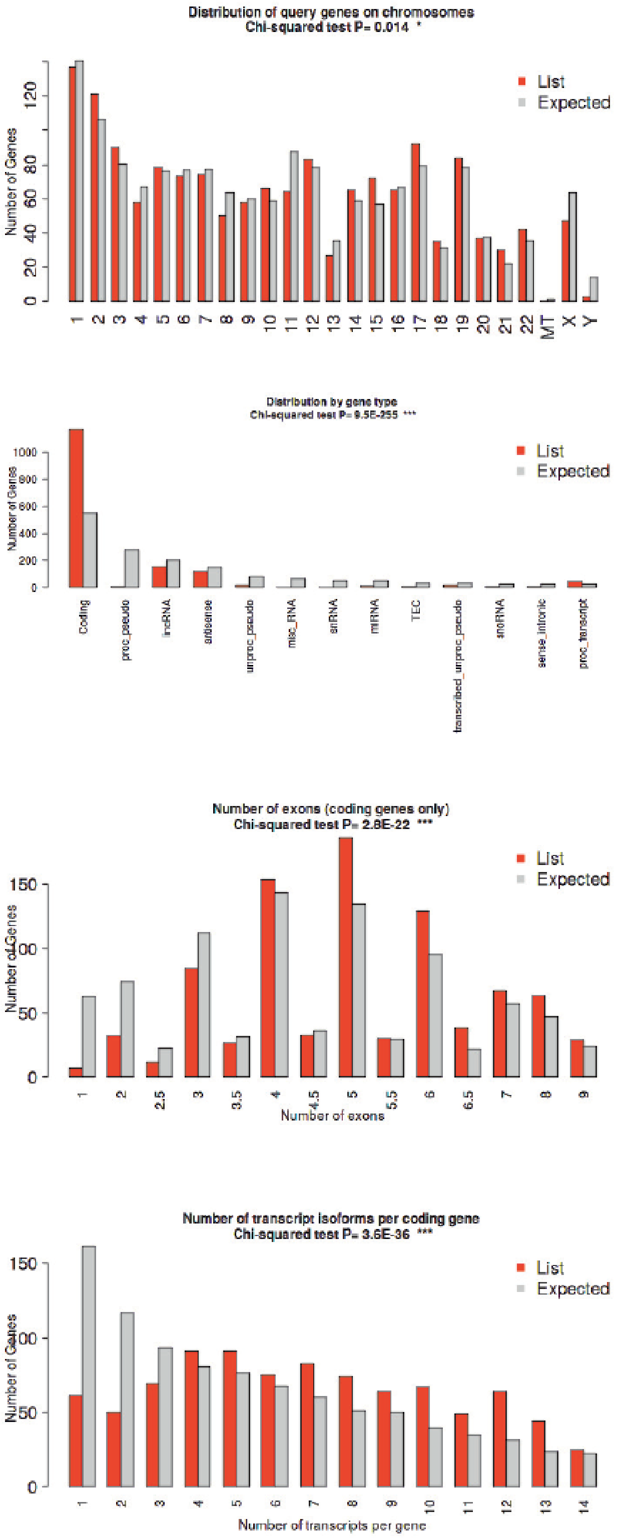

Figure S12. The distribution of srRNA targets in human chromosomes. The search was performed using the http://bioinformatics.sdstate.edu/go/ resource. All chromosomes possess the targets.

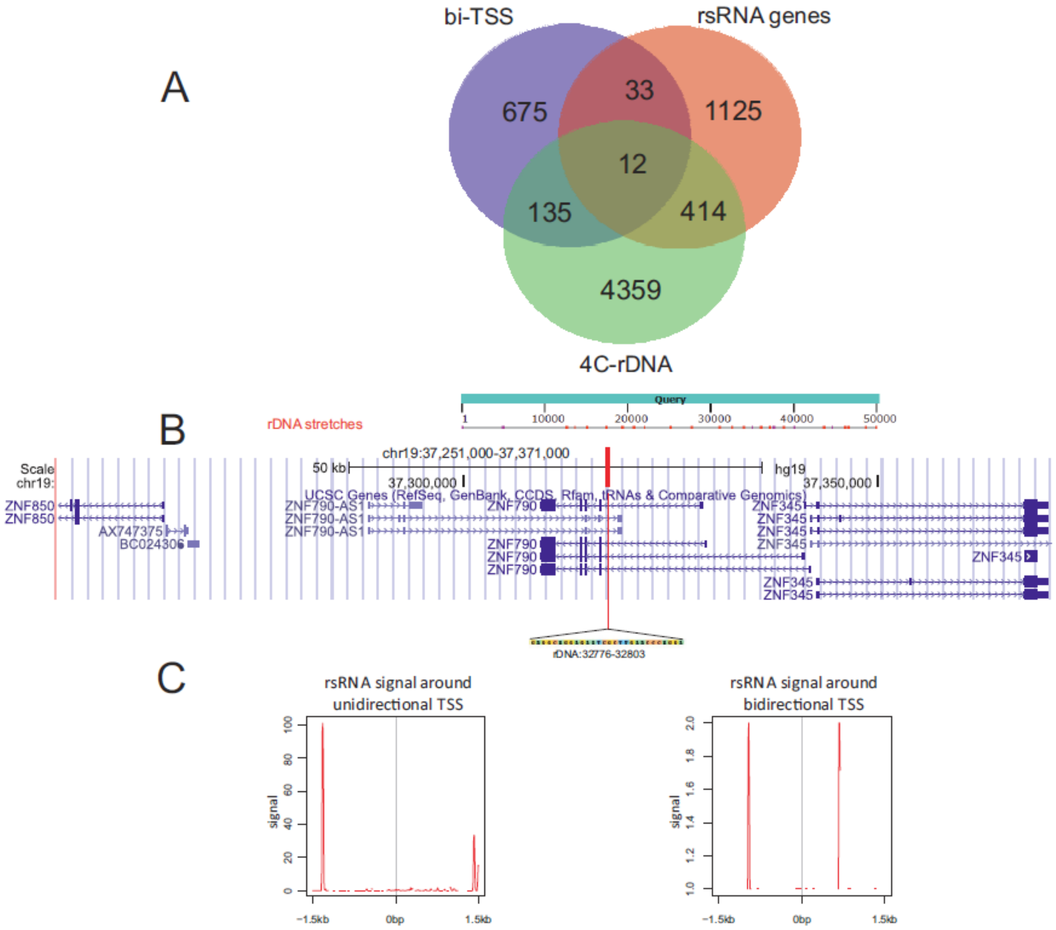

Figure S13. The srRNA targets at bidirectional TSS. (A) A Venn diagram shows the intersections between srRNA genes, rDNA-contacting genes, and bidirectional TSS. (**B**) A region from chr19 containing a bidirectional promoter that is shaped by *ZNF790* and *ZNF790-AS* genes is shown. At the top, the diverged and rearranged rDNA stretches are presented. At the bottom, srRNA from the IGS region of the rDNA gene is indicated. (**C**) Whole-genome profiles of unique srRNAs around unidirectional or bidirectional promoters in HEK293T cells. The RefSeq database at NCBI was used for the analysis.

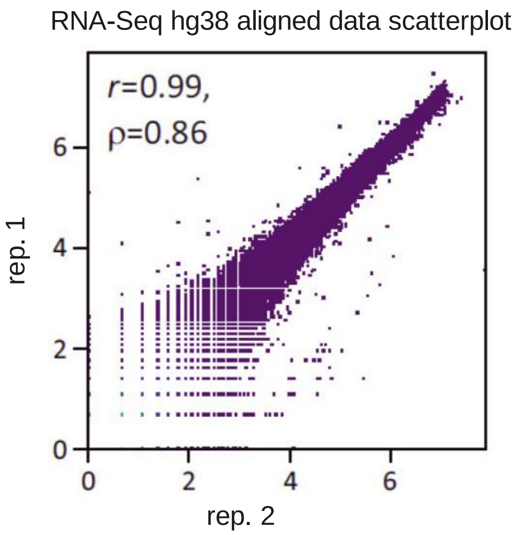

Figure S14. The consistency of RNA-Seq data. Scatterplot shows the consistency between the RNA-Seq replicates for the HEK293T cells.

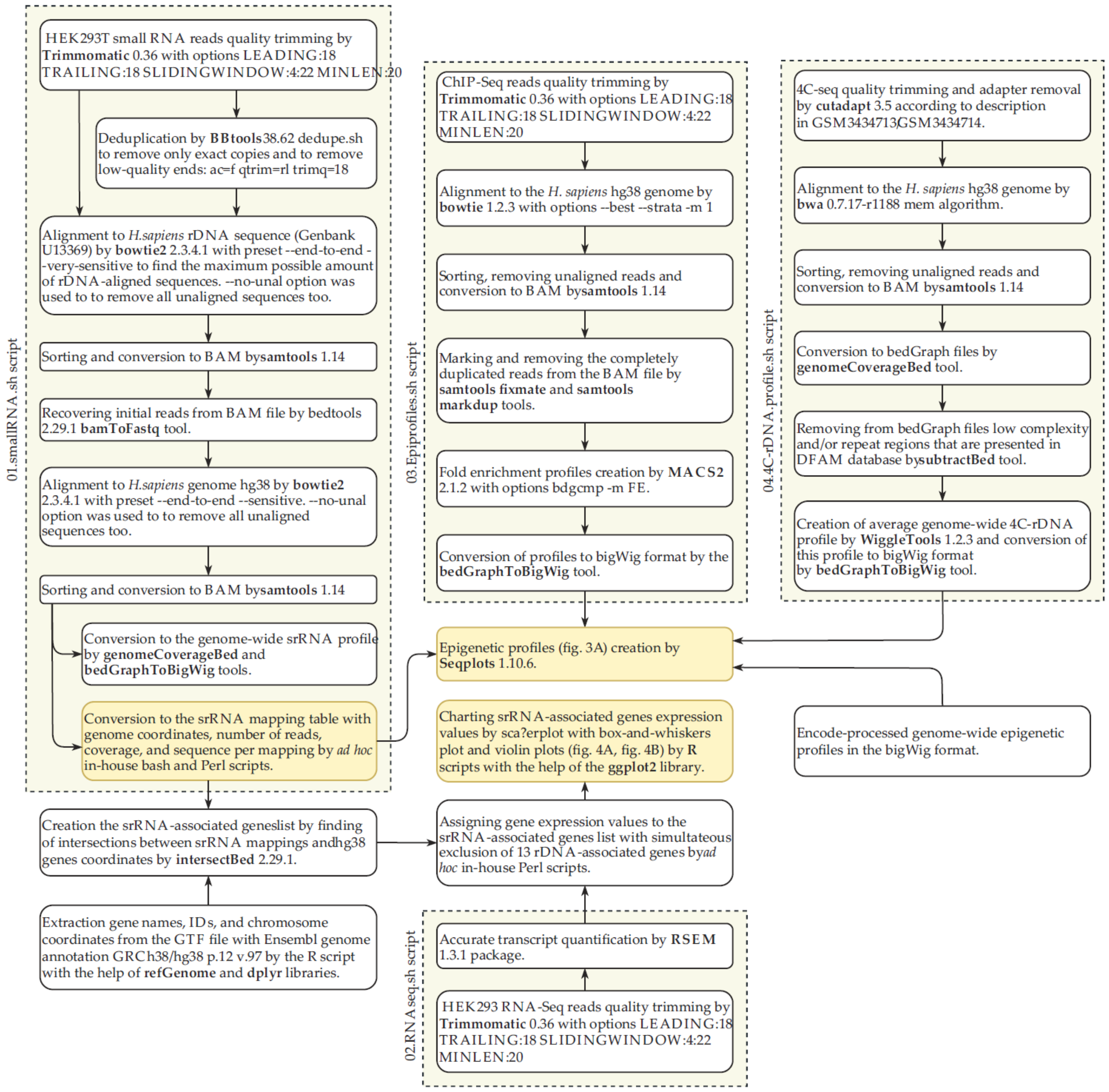

Figure S15. The bioinformatics pipeline illustrating the isolation of srRNAs and integration with downstream analysis. The details are described in the Materials and Methods. All scripts from this figure are deposited to the public Github repository: https://github.com/lokapal/IJMS2022.srRNA.

Tables S1–S6

Table S1. List of srRNA genes and the corresponding srRNA sequences. Excel file attached separately. Related to Fig. 1. The sequences corresponding to the interspersed repeats are shown in lower case. Column F shows the sequences of 19–50-nt srRNAs or the longer stretches where individual 19–50-nt srRNAs are aligned without gaps.

Table S2. GO associations with Biological Process (GO Profiler) of srRNA genes. Related to Fig. 1C.

GO.ID	Description	padj	Genes
			Biological Process
GO:0048468	cell development	1.316301E-07	*PDE3A LYN RELN HDGFL3 MAP1S MEX3C DMD RERE PAK1 LRP8 NKX2-1 RUNX1 RASGRF1 RAB11A APP CTNNA1 FOXC1 MAPK1 ACTN1 VCL HOXB3 RAP1A NOTCH2 BMP7 PPP3CA AKAP13 KMT2B PLS1 TIAM1 PTPRG GLI3 CEBPA FAM172A HDAC4 NEBL EPHA3 WDPCP ROBO2 ZSWIM4 RYR1 NUP210L CACNA1A OPCML LAMA2 FEZF2 HSP90AA1 CCNB1 ID2 GDI1 YWHAH HIF1A YY1 ADD1 MDM2 PTPN11 PDCD4 AUTS2 SPAG9 ROBO1 RBFOX2 PCM1 CCDC88A DENND5A GOLGA4 FGFR1 DLX2 ARID1B TBCD STK11 EIF2B4 UBE4B NKX2-5 ARHGEF2 SOS1 ALDH1A2 PDLIM5 OGDH PDPK1 ROCK2 MEF2A SIRT2 BRCA2 RANBP9 SHTN1 DTNBP1 ZMYND8 TRAK1 BRAF KNL1 STRN PTPN9 BCL11A MYO9A ALKBH1 HS6ST1 ITSN2 RAPGEF1 MAP2K4 ANKS1A RREB1 HERC1 TBC1D20 TPRN RCAN1 EPB41L5 RNF157 IFT74 ARHGEF26 HES6 RFX2 NEDD4L LPAR3 PLCB1 DIAPH2 UST PLXND1 PRKCA RPS6KA2 KANK1 EFNB1 IQCG DOCK5 COBL SHROOM3 PRKD1 ADGRV1 BRSK1 INPP5F EPHA6 NLGN1 OPHN1 FLI1 OSBP2 ACTN2 BMPR1B WNT5B NEDD4 CAMK1D EDNRA PPFIA2 MAGI2 WNT3 RILPL2 RNF165 ZP3 NFASC RFX3 GJC2 DCLK1 TNC AKAP6 ERBB4 FRY POU2F2 TMEFF2 PDE2A NEDD9 UNC5A NTRK2 CTNND2 PROC MEI1 NEURL1 CLDN3 ATP8A2 CCDC36 NOS1 CHODL GPR68 HRH2 KIAA0319 ALK DOK6 MOBP NTM TOX TTPA SLC4A10 PCDH15 NME5 PTPRQ TRPM1 HCN1 TENM2 OPRM1 SYCP1 RHEX CNTN4 SEMA5A SH3TC2 DCC DSCAM IL1RAPL1*
GO:0048667	cell morphogenesis involved in neuron differentiation	3.739676E-07	*RELN MAP1S RERE PAK1 LRP8 NKX2-1 RAB11A APP MAPK1 VCL NOTCH2 BMP7 PPP3CA PLS1 TIAM1 GLI3 EPHA3 WDPCP ROBO2 ZSWIM4 CACNA1A LAMA2 FEZF2 HSP90AA1 ID2 GDI1 YWHAH PTPN11 AUTS2 ROBO1 RBFOX2 GOLGA4 TBCD STK11 SOS1 PDLIM5 MEF2A RANBP9 SHTN1 DTNBP1 TRAK1 BRAF BCL11A TPRN NEDD4L LPAR3 UST PLXND1 PRKCA EFNB1 COBL BRSK1 EPHA6 NLGN1 OPHN1 BMPR1B NEDD4 PPFIA2 WNT3 RNF165 NFASC DCLK1 UNC5A NTRK2 CTNND2 ATP8A2 CHODL KIAA0319 DOK6 PCDH15 PTPRQ CNTN4 SEMA5A DCC DSCAM IL1RAPL1*
GO:0048666	neuron development	4.594074E-07	*LYN RELN HDGFL3 MAP1S DMD RERE PAK1 LRP8 NKX2-1 RUNX1 RASGRF1 RAB11A APP CTNNA1 MAPK1 VCL RAP1A NOTCH2 BMP7 PPP3CA PLS1 TIAM1 PTPRG GLI3 EPHA3 WDPCP ROBO2 ZSWIM4 CACNA1A OPCML LAMA2 FEZF2 HSP90AA1 ID2 GDI1 YWHAH MDM2 PTPN11 AUTS2 SPAG9 ROBO1 RBFOX2 CCDC88A DENND5A GOLGA4 FGFR1 ARID1B TBCD STK11 UBE4B SOS1 PDLIM5 OGDH MEF2A RANBP9 SHTN1 DTNBP1 ZMYND8 TRAK1 BRAF STRN PTPN9 BCL11A MYO9A ALKBH1 HS6ST1 ITSN2 RAPGEF1 ANKS1A HERC1 TPRN RNF157 NEDD4L LPAR3 UST PLXND1 PRKCA KANK1 EFNB1 COBL PRKD1 ADGRV1 BRSK1 INPP5F EPHA6 NLGN1 OPHN1 BMPR1B NEDD4 CAMK1D PPFIA2 MAGI2*

			WNT3 RNF165 NFASC DCLK1 TNC FRY UNC5A NTRK2 CTNND2 NEURL1 ATP8A2 CHODL KIAA0319 ALK DOK6 NTM TOX SLC4A10 PCDH15 PTPRQ TRPM1 HCN1 TENM2 CNTN4 SEMA5A DCC DSCAM IL1RAPL1
GO:0048699	generation of neurons	5.312798E-07	*LYN RELN HDGFL3 MAP1S DMD GIGYF2 RERE PAK1 LRP8 NKX2-1 RUNX1 RASGRF1 RAB11A APP CTNNA1 MAPK1 VCL HOXB3 RAP1A NOTCH2 BMP7 PPP3CA CDK5RAP2 PLS1 TIAM1 PTPRG GLI3 ZFHX3 EPHA3 WDPCP ROBO2 ZSWIM4 CACNA1A OPCML LAMA2 FEZF2 HSP90AA1 ID2 GDI1 YWHAH HIF1A DDX6 MDM2 PTPN11 AUTS2 SPAG9 ROBO1 RBFOX2 PCM1 CCDC88A DENND5A GOLGA4 FGFR1 DLX2 ARID1B TBCD STK11 UBE4B NKX2-5 NDE1 ARHGEF2 SOS1 ALDH1A2 PDLIM5 OGDH MEF2A STAT3 MYEF2 SIRT2 CIT RANBP9 SHTN1 TCF12 DTNBP1 ZMYND8 TRAK1 BRAF STRN PTPN9 BCL11A MYO9A ALKBH1 HS6ST1 ITSN2 RAPGEF1 ANKS1A IFT172 HERC1 TPRN RNF157 HES6 NEDD4L LPAR3 UST PLXND1 PRKCA CELSR1 KANK1 EFNB1 FZD8 COBL PRKD1 ADGRV1 BRSK1 INPP5F EPHA6 NLGN1 OPHN1 BMPR1B WNT5B NEDD4 CAMK1D PPFIA2 MAGI2 WNT3 RNF165 NFASC GJC2 DCLK1 TNC ERBB4 FRY IGSF10 UNC5A NTRK2 CTNND2 NEURL1 ATP8A2 NOS1 CHODL DTX1 KIAA0319 ALK DOK6 TAFA1 NTM TOX WNT9B SLC4A10 PCDH15 PTPRQ TRPM1 HCN1 TENM2 OPRM1 BHLHE22 CNTN4 SEMA5A DCC DSCAM IL1RAPL1*
GO:0000904	cell morphogenesis involved in differentiation	6.042380E-07	*RELN MAP1S RERE PAK1 LRP8 NKX2-1 RAB11A APP MAPK1 ACTN1 VCL NOTCH2 BMP7 PPP3CA PLS1 TIAM1 GLI3 EPHA3 WDPCP ROBO2 ZSWIM4 CACNA1A LAMA2 FEZF2 HSP90AA1 ID2 GDI1 YWHAH PTPN11 AUTS2 ROBO1 RBFOX2 GOLGA4 TBCD STK11 SOS1 PDLIM5 MEF2A RANBP9 SHTN1 DTNBP1 TRAK1 BRAF BCL11A RREB1 TBC1D20 TPRN EPB41L5 ARHGEF26 NEDD4L LPAR3 UST PLXND1 PRKCA KANK1 EFNB1 DOCK5 COBL BRSK1 EPHA6 NLGN1 OPHN1 BMPR1B NEDD4 PPFIA2 WNT3 RILPL2 RNF165 NFASC DCLK1 TMEFF2 NEDD9 UNC5A NTRK2 CTNND2 CLDN3 ATP8A2 CHODL HRH2 KIAA0319 DOK6 PCDH15 PTPRQ CNTN4 SEMA5A DCC DSCAM IL1RAPL1*
GO:0030182	neuron differentiation	9.102134E-07	*LYN RELN HDGFL3 MAP1S DMD GIGYF2 RERE PAK1 LRP8 NKX2-1 RUNX1 RASGRF1 RAB11A APP CTNNA1 MAPK1 VCL RAP1A NOTCH2 BMP7 PPP3CA CDK5RAP2 PLS1 TIAM1 PTPRG GLI3 ZFHX3 EPHA3 WDPCP ROBO2 ZSWIM4 CACNA1A OPCML LAMA2 FEZF2 HSP90AA1 ID2 GDI1 YWHAH HIF1A DDX6 MDM2 PTPN11 AUTS2 SPAG9 ROBO1 RBFOX2 CCDC88A DENND5A GOLGA4 FGFR1 DLX2 ARID1B TBCD STK11 UBE4B NKX2-5 ARHGEF2 SOS1 ALDH1A2 PDLIM5 OGDH MEF2A STAT3 MYEF2 RANBP9 SHTN1 TCF12 DTNBP1 ZMYND8 TRAK1 BRAF STRN PTPN9 BCL11A MYO9A ALKBH1 HS6ST1 ITSN2 RAPGEF1 ANKS1A IFT172 HERC1 TPRN RNF157 NEDD4L LPAR3 UST PLXND1 PRKCA KANK1 EFNB1 FZD8 COBL PRKD1 ADGRV1 BRSK1 INPP5F EPHA6 NLGN1 OPHN1 BMPR1B WNT5B NEDD4 CAMK1D PPFIA2 MAGI2 WNT3 RNF165 NFASC DCLK1 TNC ERBB4 FRY UNC5A NTRK2 CTNND2 NEURL1 ATP8A2 CHODL DTX1 KIAA0319 ALK DOK6 NTM TOX WNT9B SLC4A10 PCDH15 PTPRQ TRPM1 HCN1 TENM2 BHLHE22 CNTN4 SEMA5A DCC DSCAM IL1RAPL1*
GO:0022008	neurogenesis	1.584311E-06	*LYN RELN HDGFL3 MAP1S DMD GIGYF2 RERE PAK1 LRP8 NKX2-1 RUNX1 RASGRF1 RAB11A APP CTNNA1 MAPK1 VCL HOXB3 RAP1A NOTCH2 BMP7 PPP3CA CDK5RAP2 PLS1 TIAM1 PTPRG GLI3 ZFHX3 EPHA3 WDPCP ROBO2 ZSWIM4 CACNA1A OPCML LAMA2 FEZF2 HSP90AA1 ID2 GDI1 YWHAH HIF1A DDX6 MDM2 PTPN11 AUTS2 SPAG9 ROBO1 RBFOX2 NUP133 PCM1 CCDC88A DENND5A GOLGA4 FGFR1 DLX2 ARID1B TBCD STK11 EIF2B4 UBE4B NKX2-5 NDE1 ARHGEF2 SOS1 ALDH1A2 PDLIM5 OGDH MEF2A STAT3 MYEF2 SIRT2 CIT RANBP9 SHTN1 TCF12 DTNBP1 ZMYND8 TRAK1 BRAF STRN PTPN9 BCL11A MYO9A ALKBH1 HS6ST1 ITSN2 RAPGEF1 ANKS1A IFT172 HERC1 TPRN RNF157 HES6 NEDD4L LPAR3 UST PLXND1 PRKCA CELSR1 KANK1 EFNB1 FZD8 P2RX4 COBL PRKD1 ADGRV1 LEPR BRSK1 INPP5F EPHA6 NLGN1 OPHN1 BMPR1B*

			WNT5B NEDD4 CAMK1D ETV6 PPFIA2 MAGI2 WNT3 RNF165 NFASC GJC2 DCLK1 TNC ERBB4 FRY IGSF10 UNC5A NTRK2 CTNND2 NEURL1 ATP8A2 NOS1 CHODL DTX1 KIAA0319 ALK DOK6 MOBP TAFA1 NTM TOX WNT9B SLC4A10 PCDH15 PTPRQ TRPM1 HCN1 TENM2 OPRM1 BHLHE22 CNTN4 SEMA5A SH3TC2 DCC DSCAM IL1RAPL1
GO:0031175	neuron projection development	4.804454E-06	*LYN RELN HDGFL3 MAP1S DMD RERE PAK1 LRP8 NKX2-1 RASGRF1 RAB11A APP CTNNA1 MAPK1 VCL RAP1A NOTCH2 BMP7 PPP3CA PLS1 TIAM1 PTPRG GLI3 EPHA3 ROBO2 ZSWIM4 CACNA1A LAMA2 FEZF2 HSP90AA1 GDI1 YWHAH MDM2 PTPN11 AUTS2 SPAG9 ROBO1 RBFOX2 CCDC88A DENND5A GOLGA4 FGFR1 ARID1B STK11 UBE4B SOS1 PDLIM5 MEF2A RANBP9 SHTN1 DTNBP1 ZMYND8 TRAK1 BRAF STRN PTPN9 BCL11A MYO9A ALKBH1 ITSN2 RAPGEF1 HERC1 TPRN RNF157 NEDD4L LPAR3 UST PLXND1 PRKCA KANK1 EFNB1 COBL PRKD1 ADGRV1 BRSK1 INPP5F EPHA6 NLGN1 OPHN1 BMPR1B NEDD4 CAMK1D PPFIA2 MAGI2 WNT3 RNF165 NFASC DCLK1 TNC FRY UNC5A NTRK2 CTNND2 NEURL1 ATP8A2 CHODL KIAA0319 ALK DOK6 TOX PCDH15 CNTN4 SEMA5A DCC DSCAM IL1RAPL1*
GO:0007399	nervous system development	7.593441E-06	*LYN RELN CCDC85C LRRTM3 HDGFL3 MYO1D MAP1S DMD GIGYF2 RERE PAK1 LRP8 NOTCH2NLA POU3F3 NKX2-1 RUNX1 BSN RASGRF1 RAB11A APP CTNNA1 FOXC1 MAPK1 BCR VCL HOXB3 RAP1A ABR NOTCH2 BMP7 PPP3CA CDK5RAP2 PLS1 TIAM1 PTPRG GLI3 FZD6 ZFHX3 HDAC4 FBXL17 EPHA3 IMMP2L WDPCP ROBO2 ZSWIM4 CACNA1A OPCML LAMA2 FEZF2 HSP90AA1 ID2 GDI1 YWHAH BPTF HIF1A DDX6 MDM2 PTPN11 WLS AUTS2 SPAG9 ROBO1 RBFOX2 NUP133 PCM1 CHD8 CLSTN1 B4GALT2 CCDC88A SETD1A DENND5A GOLGA4 FGFR1 DLX2 ARID1B TBCD STK11 EIF2B4 UBE4B NKX2-5 NDE1 ARHGEF2 SOS1 ALDH1A2 PDLIM5 OGDH ESS2 MEF2A STAT3 KDM2B MYEF2 SIRT2 CIT BRCA2 RANBP9 SHTN1 DLC1 TCF12 DTNBP1 ZMYND8 TRAK1 BRAF PCDH9 STRN PTPN9 BCL11A MYO9A WWP1 ALKBH1 HS6ST1 ITSN2 RAPGEF1 ANKS1A IFT172 HERC1 TPRN RNF157 PPARD HES6 NEDD4L LPAR3 PLCB1 UST PLXND1 PRKCA LIAS CELSR1 NPAS2 KANK1 EFNB1 FZD8 P2RX4 ABAT COBL SHROOM3 PRKD1 ADGRV1 LEPR BRSK1 INPP5F EPHA6 NLGN1 OPHN1 GRIN2D SALL4 BMPR1B CHST8 WNT5B NEDD4 CAMK1D ETV6 EDNRA PPFIA2 MAGI2 KALRN WNT3 RNF165 CHRM3 NFASC GJC2 DCLK1 TNC ERBB4 FRY SNTG2 IGSF10 SCIN UNC5A NTRK2 CTNND2 NEURL1 CLDN3 ATP8A2 NOS1 CHODL DTX1 LRRN3 KIAA0319 ALK DOK6 MOBP TAFA1 NTM TOX TTPA WNT9B BCAS1 SLC4A10 DNAH5 PCDH15 NME5 PTPRQ TRPM1 MALL HCN1 TENM2 OPRM1 BHLHE22 KCNC2 VIT CNTN4 SEMA5A SH3TC2 TFAP2D LINGO2 CA10 DCC DSCAM IL1RAPL1*
GO:0032989	cellular component morphogenesis	2.062463E-05	*PID1 RELN MAP1S DMD RERE PAK1 LRP8 NKX2-1 RAB11A APP MAPK1 VCL NOTCH2 BMP7 PPP3CA AKAP13 TIAM1 GLI3 NEBL EPHA3 ROBO2 ZSWIM4 CACNA1A LAMA2 FEZF2 HSP90AA1 GDI1 YWHAH PTPN11 AUTS2 SPAG9 ROBO1 RBFOX2 GOLGA4 STK11 NKX2-5 SOS1 PDLIM5 MEF2A RANBP9 SHTN1 DTNBP1 TRAK1 BRAF KNL1 BCL11A MYO9A ITSN2 CLASP1 RREB1 TBC1D20 RNF157 RFX2 NEDD4L LPAR3 MTM1 UST PLXND1 PRKCA KANK1 EFNB1 COBL BRSK1 EPHA6 NLGN1 OPHN1 ACTN2 BMPR1B NEDD4 PPFIA2 WNT3 RNF165 NFASC DCLK1 UNC5A NTRK2 CTNND2 ATP8A2 CHODL KIAA0319 DOK6 MOBP CNTN4 SEMA5A DCC DSCAM IL1RAPL1*
GO:0043412	macromolecule modification	3.037811E-05	*ALG9 AP001781.2 GCNT1 PID1 EYA4 LYN RELN PPEF2 GALNT1 BMP8B USP53 MEX3C DMD RGS3 PAK1 LRP8 ZNRF3 RNF150 TEX14 CD80 RAB11A APP MAPK1 NUP98 BCR OTUD5 NAA25 KDM2A PIM3 PUS7 RAP1A ABR BMP7 OGG1 PPP3CA MAP3K3 ATXN7 THADA AKAP13 KMT2B USP13 PHKB TIAM1 PTPRG HERC4 PHLPP1 CEBPA HDAC4 SASH1 FBXL17 EPHA3 CD74 FLT3 FEZF2 PRMT8 HSP90AA1 ILF3 DNMT1 UBE2M CCNB1 ARIH1 PSMB2 SUMO1 UBE2D2 IPO7 IPO5 SSU72 CDC27 NPEPPS HIF1A CAMTA1*

			YY1 FXR1 KTN1 UBE2V2 CUL1 MDM2 PTPN11 HELLS BAZ1A UHRF1 PDCD4 AUTS2 SPAG9 RNF220 ROBO1 NAA15 NSD1 PPP1R12A FAF1 NUP133 SEPHS1 ELP6 TSG101 B4GALT2 DPH1 WNK2 CCDC88A CDC23 PPP1R7 BRCA1 RNF130 HDAC8 SETD1A PIAS1 EHMT1 GALNT2 TRMT1 FGFR1 LRRC41 RC3H2 NXN PPME1 AIDA SENP6 SELENOS MBD2 STK11 UBE4B NAA60 CHEK1 RNF4 ARHGEF2 UNKL TSPYL2 TELO2 POR FBXO28 PTPN1 GCLC MTA3 KCTD10 AP3B1 MAP2K7 SRPK2 BLM RBX1 METTL6 RPAP2 EIF2AK2 OGDH ATF7IP PRMT3 PDPK1 CCNE1 NEK4 PIGU ITCH MYSM1 ROCK2 CSNK1G3 CASK STAT3 KDM2B OTUD4 USP4 SIRT2 CIT FOXF2 UBR3 MAP3K2 BRCA2 SLC39A10 CCNY KANSL2 ASCC3 RNF149 RNF121 RPTOR DLC1 USP32 RNF111 KLHL22 DTNBP1 PPP2R3C PEX14 TRAK1 GFPT1 BRAF PIGX HECTD4 BRAP TULP4 ASB3 PTPN9 BCL11A SIRT1 TNFRSF10B PKN3 KMT5B WWP1 ALKBH1 PTPN4 KDM4B RAPGEF1 MAP2K4 MAP2K5 TRIM14 FUT8 BTRC DCAF10 HERC1 USP18 CHFR PHKA1 OTUD7B PIGN KAT14 RCAN1 CDK17 TYW1 OXR1 RNF157 ERRFI1 XXYLT1 STK39 ACSL1 FBXW4 TET1 MOB1B ABHD17C RPS6KC1 LARGE1 RHOT1 NEDD4L FAM161A LPAR3 SNTA1 TBCK PALD1 JADE3 MTM1 UST DSTYK PRKCA EEF1AKMT2 LIAS NEDD8-MDP1 RPS6KA2 SPRED2 FZD8 KANSL1L FAM220A PRKD1 USP44 LEPR PLAUR BRSK1 FAXDC2 INPP5F EPHA6 WDFY2 FBXO4 PPM1N SHC2 EYA2 BMPR1B RNF43 HUNK MDP1 NEDD4 CAMK1D PPP1R3B RBM47 RAB3B MYO3A INKA2 MAGI2 KALRN LDLRAD4 ACER2 RNF165 AGAP2 CHRM3 OTUD7A GJC2 DCLK1 AQP11 USP43 TNC ERBB4 FRY PLCL1 AL133500.1 GALNT14 NEDD9 NTRK2 TNXB FBXL13 PROC AC068896.1 TRAF1 NEURL1 CLDN3 HS3ST5 NOS1 AC004687.2 PTPN22 LEFTY1 DTX1 SLCO3A1 PPP1R36 IL7 ALK SLC11A1 BANK1 TOX WNT9B LRRC2 AC013717.1 PTPRQ PTPRR AC058822.1 PADI4 MGAT4C TDRD9 ARRDC5 PTPRT
GO:0036211	protein modification process	5.290544E-05	*ALG9 AP001781.2 GCNT1 PID1 EYA4 LYN RELN PPEF2 GALNT1 BMP8B USP53 MEX3C DMD RGS3 PAK1 LRP8 ZNRF3 RNF150 TEX14 CD80 RAB11A APP MAPK1 NUP98 BCR OTUD5 NAA25 KDM2A PIM3 RAP1A ABR BMP7 PPP3CA MAP3K3 ATXN7 AKAP13 KMT2B USP13 PHKB TIAM1 PTPRG HERC4 PHLPP1 CEBPA HDAC4 SASH1 FBXL17 EPHA3 CD74 FLT3 FEZF2 PRMT8 HSP90AA1 ILF3 DNMT1 UBE2M CCNB1 ARIH1 PSMB2 SUMO1 UBE2D2 IPO7 IPO5 SSU72 CDC27 NPEPPS HIF1A CAMTA1 YY1 FXR1 KTN1 UBE2V2 CUL1 MDM2 PTPN11 BAZ1A UHRF1 PDCD4 AUTS2 SPAG9 RNF220 ROBO1 NAA15 NSD1 PPP1R12A FAF1 NUP133 SEPHS1 TSG101 B4GALT2 DPH1 WNK2 CCDC88A CDC23 PPP1R7 BRCA1 RNF130 HDAC8 SETD1A PIAS1 EHMT1 GALNT2 FGFR1 LRRC41 RC3H2 NXN PPME1 AIDA SENP6 SELENOS STK11 UBE4B NAA60 CHEK1 RNF4 ARHGEF2 UNKL TSPYL2 TELO2 POR FBXO28 PTPN1 GCLC MTA3 KCTD10 AP3B1 MAP2K7 SRPK2 BLM RBX1 RPAP2 EIF2AK2 OGDH PRMT3 PDPK1 CCNE1 NEK4 PIGU ITCH MYSM1 ROCK2 CSNK1G3 CASK STAT3 KDM2B OTUD4 USP4 SIRT2 CIT FOXF2 UBR3 MAP3K2 BRCA2 SLC39A10 CCNY KANSL2 RNF149 RNF121 RPTOR DLC1 USP32 RNF111 KLHL22 DTNBP1 PPP2R3C PEX14 TRAK1 GFPT1 BRAF PIGX HECTD4 BRAP TULP4 ASB3 PTPN9 BCL11A SIRT1 TNFRSF10B PKN3 KMT5B WWP1 PTPN4 KDM4B RAPGEF1 MAP2K4 MAP2K5 TRIM14 FUT8 BTRC DCAF10 HERC1 USP18 CHFR PHKA1 OTUD7B PIGN KAT14 RCAN1 CDK17 OXR1 RNF157 ERRFI1 XXYLT1 STK39 ACSL1 FBXW4 TET1 MOB1B ABHD17C RPS6KC1 LARGE1 RHOT1 NEDD4L FAM161A LPAR3 SNTA1 TBCK PALD1 JADE3 MTM1 UST DSTYK PRKCA EEF1AKMT2 LIAS NEDD8-MDP1 RPS6KA2 SPRED2 FZD8 KANSL1L FAM220A PRKD1 USP44 LEPR PLAUR BRSK1 FAXDC2 INPP5F EPHA6 WDFY2 FBXO4 PPM1N SHC2 EYA2 BMPR1B RNF43 HUNK MDP1 NEDD4 CAMK1D PPP1R3B RAB3B MYO3A INKA2 MAGI2 KALRN LDLRAD4 ACER2 RNF165 AGAP2 CHRM3 OTUD7A GJC2 DCLK1 AQP11 USP43 TNC ERBB4 FRY PLCL1 AL133500.1 GALNT14 NEDD9 NTRK2 TNXB FBXL13 PROC AC068896.1 TRAF1 NEURL1 CLDN3 HS3ST5 NOS1 AC004687.2 PTPN22*

			LEFTY1 DTX1 SLCO3A1 PPP1R36 IL7 ALK SLC11A1 BANK1 WNT9B LRRC2 AC013717.1 PTPRQ PTPRR AC058822.1 PADI4 MGAT4C ARRDC5 PTPRT
GO:0006464	cellular protein modification process	5.290544E-05	*ALG9 AP001781.2 GCNT1 PID1 EYA4 LYN RELN PPEF2 GALNT1 BMP8B USP53 MEX3C DMD RGS3 PAK1 LRP8 ZNRF3 RNF150 TEX14 CD80 RAB11A APP MAPK1 NUP98 BCR OTUD5 NAA25 KDM2A PIM3 RAP1A ABR BMP7 PPP3CA MAP3K3 ATXN7 AKAP13 KMT2B USP13 PHKB TIAM1 PTPRG HERC4 PHLPP1 CEBPA HDAC4 SASH1 FBXL17 EPHA3 CD74 FLT3 FEZF2 PRMT8 HSP90AA1 ILF3 DNMT1 UBE2M CCNB1 ARIH1 PSMB2 SUMO1 UBE2D2 IPO7 IPO5 SSU72 CDC27 NPEPPS HIF1A CAMTA1 YY1 FXR1 KTN1 UBE2V2 CUL1 MDM2 PTPN11 BAZ1A UHRF1 PDCD4 AUTS2 SPAG9 RNF220 ROBO1 NAA15 NSD1 PPP1R12A FAF1 NUP133 SEPHS1 TSG101 B4GALT2 DPH1 WNK2 CCDC88A CDC23 PPP1R7 BRCA1 RNF130 HDAC8 SETD1A PIAS1 EHMT1 GALNT2 FGFR1 LRRC41 RC3H2 NXN PPME1 AIDA SENP6 SELENOS STK11 UBE4B NAA60 CHEK1 RNF4 ARHGEF2 UNKL TSPYL2 TELO2 POR FBXO28 PTPN1 GCLC MTA3 KCTD10 AP3B1 MAP2K7 SRPK2 BLM RBX1 RPAP2 EIF2AK2 OGDH PRMT3 PDPK1 CCNE1 NEK4 PIGU ITCH MYSM1 ROCK2 CSNK1G3 CASK STAT3 KDM2B OTUD4 USP4 SIRT2 CIT FOXF2 UBR3 MAP3K2 BRCA2 SLC39A10 CCNY KANSL2 RNF149 RNF121 RPTOR DLC1 USP32 RNF111 KLHL22 DTNBP1 PPP2R3C PEX14 TRAK1 GFPT1 BRAF PIGX HECTD4 BRAP TULP4 ASB3 PTPN9 BCL11A SIRT1 TNFRSF10B PKN3 KMT5B WWP1 PTPN4 KDM4B RAPGEF1 MAP2K4 MAP2K5 TRIM14 FUT8 BTRC DCAF10 HERC1 USP18 CHFR PHKA1 OTUD7B PIGN KAT14 RCAN1 CDK17 OXR1 RNF157 ERRFI1 XXYLT1 STK39 ACSL1 FBXW4 TET1 MOB1B ABHD17C RPS6KC1 LARGE1 RHOT1 NEDD4L FAM161A LPAR3 SNTA1 TBCK PALD1 JADE3 MTM1 UST DSTYK PRKCA EEF1AKMT2 LIAS NEDD8-MDP1 RPS6KA2 SPRED2 FZD8 KANSL1L FAM220A PRKD1 USP44 LEPR PLAUR BRSK1 FAXDC2 INPP5F EPHA6 WDFY2 FBXO4 PPM1N SHC2 EYA2 BMPR1B RNF43 HUNK MDP1 NEDD4 CAMK1D PPP1R3B RAB3B MYO3A INKA2 MAGI2 KALRN LDLRAD4 ACER2 RNF165 AGAP2 CHRM3 OTUD7A GJC2 DCLK1 AQP11 USP43 TNC ERBB4 FRY PLCL1 AL133500.1 GALNT14 NEDD9 NTRK2 TNXB FBXL13 PROC AC068896.1 TRAF1 NEURL1 CLDN3 HS3ST5 NOS1 AC004687.2 PTPN22 LEFTY1 DTX1 SLCO3A1 PPP1R36 IL7 ALK SLC11A1 BANK1 WNT9B LRRC2 AC013717.1 PTPRQ PTPRR AC058822.1 PADI4 MGAT4C ARRDC5 PTPRT*
GO:0032990	cell part morphogenesis	6.275903E-05	*PID1 RELN MAP1S DMD RERE PAK1 LRP8 NKX2-1 RAB11A APP MAPK1 VCL NOTCH2 BMP7 PPP3CA TIAM1 GLI3 EPHA3 ROBO2 ZSWIM4 CACNA1A LAMA2 FEZF2 HSP90AA1 GDI1 YWHAH PTPN11 AUTS2 SPAG9 ROBO1 RBFOX2 GOLGA4 STK11 SOS1 PDLIM5 MEF2A RANBP9 SHTN1 DTNBP1 TRAK1 BRAF BCL11A MYO9A ITSN2 RREB1 RNF157 NEDD4L LPAR3 MTM1 UST PLXND1 PRKCA KANK1 EFNB1 COBL BRSK1 EPHA6 NLGN1 OPHN1 BMPR1B NEDD4 PPFIA2 WNT3 RNF165 NFASC DCLK1 UNC5A NTRK2 CTNND2 ATP8A2 CHODL KIAA0319 DOK6 CNTN4 SEMA5A DCC DSCAM IL1RAPL1*
GO:0120039	plasma membrane bounded cell projection morphogenesis	7.222962E-05	*RELN MAP1S DMD RERE PAK1 LRP8 NKX2-1 RAB11A APP MAPK1 VCL NOTCH2 BMP7 PPP3CA TIAM1 GLI3 EPHA3 ROBO2 ZSWIM4 CACNA1A LAMA2 FEZF2 HSP90AA1 GDI1 YWHAH PTPN11 AUTS2 SPAG9 ROBO1 RBFOX2 GOLGA4 STK11 SOS1 PDLIM5 MEF2A RANBP9 SHTN1 DTNBP1 TRAK1 BRAF BCL11A MYO9A ITSN2 RREB1 RNF157 NEDD4L LPAR3 UST PLXND1 PRKCA KANK1 EFNB1 COBL BRSK1 EPHA6 NLGN1 OPHN1 BMPR1B NEDD4 PPFIA2 WNT3 RNF165 NFASC DCLK1 UNC5A NTRK2 CTNND2 ATP8A2 CHODL KIAA0319 DOK6 CNTN4 SEMA5A DCC DSCAM IL1RAPL1*
GO:0048858	cell projection morphogenesis	9.202244E-05	*RELN MAP1S DMD RERE PAK1 LRP8 NKX2-1 RAB11A APP MAPK1 VCL NOTCH2 BMP7 PPP3CA TIAM1 GLI3 EPHA3 ROBO2 ZSWIM4 CACNA1A LAMA2 FEZF2 HSP90AA1 GDI1 YWHAH PTPN11 AUTS2 SPAG9 ROBO1 RBFOX2 GOLGA4 STK11 SOS1 PDLIM5 MEF2A RANBP9 SHTN1 DTNBP1*

			TRAK1 BRAF BCL11A MYO9A ITSN2 RREB1 RNF157 NEDD4L LPAR3 UST PLXND1 PRKCA KANK1 EFNB1 COBL BRSK1 EPHA6 NLGN1 OPHN1 BMPR1B NEDD4 PPFIA2 WNT3 RNF165 NFASC DCLK1 UNC5A NTRK2 CTNND2 ATP8A2 CHODL KIAA0319 DOK6 CNTN4 SEMA5A DCC DSCAM IL1RAPL1
GO:0120036	plasma membrane bounded cell projection organization	1.135557E-04	*LYN RELN HDGFL3 MAP1S DMD RERE PAK1 LRP8 NKX2-1 RASGRF1 RAB11A APP CTNNA1 MAPK1 VCL LGMN RAP1A NOTCH2 BMP7 PPP3CA CDK5RAP2 PLS1 TIAM1 PTPRG GLI3 ARHGEF4 HDAC4 EPHA3 WDPCP ROBO2 ZSWIM4 CACNA1A LAMA2 FEZF2 HSP90AA1 GDI1 YWHAH FXR1 SEPT9 MDM2 PTPN11 AUTS2 SPAG9 ROBO1 RBFOX2 PCM1 CCDC88A SEPT6 DENND5A GOLGA4 FGFR1 ARID1B RHOQ STK11 UBE4B NDE1 SOS1 PDLIM5 MEF2A RANBP9 SHTN1 MYO10 OFD1 DTNBP1 ZMYND8 TRAK1 BRAF IFT81 STRN PTPN9 BCL11A MYO9A ALKBH1 ITSN2 CLASP1 RAPGEF1 RREB1 IFT172 HERC1 CROCC TPRN RNF157 IFT74 ARHGEF26 FBF1 DOCK11 RFX2 NEDD4L FAM161A LPAR3 UST PLXND1 CEP76 PRKCA KANK1 EFNB1 IQCG COBL PRKD1 ADGRV1 SNX10 BRSK1 INPP5F EPHA6 NLGN1 OPHN1 ACTN2 BMPR1B NEDD4 MTSS1 CAMK1D PPFIA2 MAGI2 WNT3 RILPL2 RNF165 NFASC RFX3 DCLK1 TNC FRY UNC5A NTRK2 CTNND2 NEURL1 CFAP61 ATP8A2 CHODL KIAA0319 ALK DOK6 TOX DNAH5 PCDH15 NME5 TENM2 CNTN4 SEMA5A DCC DSCAM IL1RAPL1*
GO:0031346	positive regulation of cell projection organization	1.208944E-04	*LYN RELN DMD PAK1 LRP8 RAB11A RAP1A BMP7 TIAM1 HDAC4 EPHA3 ROBO2 GDI1 SEPT9 AUTS2 ROBO1 CCDC88A GOLGA4 FGFR1 RHOQ STK11 SHTN1 ZMYND8 TRAK1 BRAF BCL11A RAPGEF1 RREB1 CROCC DOCK11 LPAR3 PLXND1 COBL PRKD1 NLGN1 CAMK1D MAGI2 WNT3 NTRK2 NEURL1 ATP8A2 CHODL ALK TOX TENM2 SEMA5A DSCAM IL1RAPL1*
GO:0048812	neuron projection morphogenesis	1.431853E-04	*RELN MAP1S DMD RERE PAK1 LRP8 NKX2-1 RAB11A APP MAPK1 VCL NOTCH2 BMP7 PPP3CA TIAM1 GLI3 EPHA3 ROBO2 ZSWIM4 CACNA1A LAMA2 FEZF2 HSP90AA1 GDI1 YWHAH PTPN11 AUTS2 SPAG9 ROBO1 RBFOX2 GOLGA4 STK11 SOS1 PDLIM5 MEF2A RANBP9 SHTN1 DTNBP1 TRAK1 BRAF BCL11A MYO9A ITSN2 RNF157 NEDD4L LPAR3 UST PLXND1 PRKCA EFNB1 COBL BRSK1 EPHA6 NLGN1 OPHN1 BMPR1B NEDD4 PPFIA2 WNT3 RNF165 NFASC DCLK1 UNC5A NTRK2 CTNND2 ATP8A2 CHODL KIAA0319 DOK6 CNTN4 SEMA5A DCC DSCAM IL1RAPL1*
GO:0000902	cell morphogenesis	1.548785E-04	*RELN MAP1S DMD RERE PAK1 LRP8 NKX2-1 RAB11A APP EPB41 MAPK1 ACTN1 VCL NOTCH2 BMP7 PPP3CA PLS1 TIAM1 GLI3 EPHA3 WDPCP ROBO2 ZSWIM4 CACNA1A LAMA2 FEZF2 HSP90AA1 ID2 GDI1 YWHAH ADD1 PTPN11 AUTS2 SPAG9 ROBO1 RBFOX2 GOLGA4 RHOQ TBCD STK11 ARHGEF2 SOS1 AP3B1 PDLIM5 MEF2A RANBP9 SHTN1 MYO10 DLC1 DTNBP1 TRAK1 BRAF BCL11A MYO9A ITSN2 RREB1 TBC1D20 TPRN EPB41L5 RNF157 ARHGEF26 NEDD4L LPAR3 UST PLXND1 PRKCA KANK1 EFNB1 DOCK5 COBL SHROOM3 BRSK1 EPHA6 NLGN1 OPHN1 BMPR1B NEDD4 PPFIA2 WNT3 RILPL2 RNF165 NFASC DCLK1 CDH18 FRY TMEFF2 NEDD9 UNC5A NTRK2 CTNND2 CLDN3 ATP8A2 CHODL HRH2 KIAA0319 DOK6 PCDH15 PTPRQ CNTN4 SEMA5A DCC DSCAM IL1RAPL1*
GO:0030030	cell projection organization	1.817047E-04	*LYN RELN HDGFL3 MAP1S DMD RERE PAK1 LRP8 NKX2-1 RASGRF1 RAB11A APP CTNNA1 MAPK1 VCL LGMN RAP1A NOTCH2 BMP7 PPP3CA CDK5RAP2 PLS1 TIAM1 PTPRG GLI3 ARHGEF4 HDAC4 EPHA3 WDPCP ROBO2 ZSWIM4 CACNA1A LAMA2 FEZF2 HSP90AA1 GDI1 YWHAH FXR1 SEPT9 MDM2 PTPN11 AUTS2 SPAG9 ROBO1 RBFOX2 PCM1 CCDC88A SEPT6 DENND5A GOLGA4 FGFR1 ARID1B RHOQ STK11 UBE4B NDE1 SOS1 PDLIM5 MEF2A RANBP9 SHTN1 MYO10 OFD1 DTNBP1 ZMYND8 TRAK1 BRAF IFT81 STRN PTPN9 BCL11A MYO9A ALKBH1 ITSN2 CLASP1 RAPGEF1 RREB1 IFT172 HERC1 CROCC TPRN RNF157 IFT74 ARHGEF26 FBF1 DOCK11 RFX2 NEDD4L FAM161A LPAR3 UST PLXND1 CEP76 PRKCA KANK1 EFNB1 IQCG COBL PRKD1 ADGRV1 SNX10 BRSK1 INPP5F EPHA6 NLGN1 OPHN1 ACTN2 BMPR1B NEDD4 MTSS1 CAMK1D PPFIA2 MAGI2 WNT3 RILPL2*

			RNF165 NFASC RFX3 DCLK1 TNC FRY SCIN UNC5A NTRK2 CTNND2 NEURL1 CFAP61 ATP8A2 CHODL KIAA0319 ALK DOK6 TOX DNAH5 PCDH15 NME5 TENM2 DNAH9 CNTN4 SEMA5A DCC DSCAM IL1RAPL1
GO:0048523	negative regulation of cellular process	2.355613E-04	SEC14L1 PDE3A RYR2 PID1 DLG2 SORCS2 EYA4 LYN EIF3E ADAMTS8 PPEF2 HDGFL3 MYO1D DMD PLCG2 RGS3 NCL GIGYF2 RERE PAK1 ZNRF3 POU3F3 SLC8B1 NKX2-1 RUNX1 TEX14 CD80 DKK2 APP CTNNA1 FOXC1 MAPK1 BCR NAMPT OAZ2 ACTN1 ZNF282 VCL LGMN KDM2A PIM3 PUS7 HOXB3 RAP1A ABR NOTCH2 BMP7 OGG1 PPP3CA CDK5RAP2 ZNF131 MAP3K3 ATXN7 THADA GTF2IRD1 PTPRG HERC4 GLI3 PHLPP1 FZD6 ZFHX3 CEBPA FAM172A HDAC4 AHR FOXF1 EPHA3 ROBO2 RYR3 CD74 CPAMD8 RYR1 CACNA1A FEZF2 NONO ST13 ILF3 TMBIM6 SNRNP70 DNMT1 HNRNPR ACIN1 PRCC CCNB1 ID2 PSMB2 SUMO1 IPO7 CDC123 IPO5 GDI1 ELAVL1 YWHAH BPTF FOXM1 HIF1A NAA38 NCOR1 PUM1 YY1 FXR1 DDX6 EXOSC10 CUL1 ADD1 MDM2 PTPN11 HELLS NPLOC4 DNAJC2 UHRF1 PDCD4 SPAG9 SARNP ROBO1 RBFOX2 NAA15 NSD1 PCM1 GIPC1 CHD8 TFDP1 TSG101 WNK2 BRCA1 TERF2 CNOT6 PDS5A HDAC8 DENND5A TNRC6A PIAS1 EHMT1 GTSE1 TFDP2 CHD3 FGFR1 DLX2 RC3H2 NXN PPME1 RHOQ AIDA PDS5B SELENOS RHBDD3 MBD2 TBCD STK11 CHEK1 NKX2-5 RNF4 ARHGEF2 TSPYL2 POR ALDH1A2 PTPN1 GCLC IDE MTA3 KCTD10 BLM RBX1 EIF2AK2 WAPL ATF7IP PRMT3 PDPK1 CCNE1 TCF7L2 ITCH SCMH1 TP53BP1 PSMG2 ROCK2 MEF2A CASK STAT3 EPN2 KDM2B OTUD4 USP4 SIRT2 FOXF2 RAB11FIP3 TNRC6B BRCA2 RBL2 SLC39A10 RANBP9 RNF149 EPS15 RPTOR DLC1 KLHL22 DTNBP1 PEX14 WDR76 ZMYND8 CHMP4A BRAF BRAP KNL1 STRN PTPN9 BCL11A SIRT1 CARD8 TNFRSF10B WWP1 ALKBH1 KDM4B CLASP1 RAPGEF1 ZNF263 WWOX MAP2K4 RREB1 MAP2K5 BTRC SMG6 IFT172 HERC1 FOXN3 CHFR RHBDF2 OTUD7B CLEC16A ZNF620 RCAN1 EPB41L5 CNOT6L DEPDC5 TMEM131L OXR1 RNF157 ERRFI1 IFT74 CTNNBIP1 STK39 TET1 NFIX PPARD ABHD17C HES6 MCC NEDD4L TNRC6C CREM SNTA1 PLCB1 MTM1 HIVEP1 TRPC1 PLXND1 DSTYK PRKCA ZNF875 RPS6KA2 TLE2 SPRED2 NPAS2 KANK1 ABAT ZNFX1 FAM220A PROS1 JAZF1 PRKD1 ADGRV1 USP44 LEPR TNFAIP8 PLAUR BRSK1 INPP5F CCDC3 FBXO4 SOGA1 NLGN1 OPHN1 PPM1N ACTN2 SALL4 EYA2 BMPR1B RNF43 SVIL WNT5B CPEB2 NEDD4 MTSS1 CAMK1D ETV6 CDA ADAMTS9 ARFGEF3 INKA2 MAGI2 WNT3 ESRRB LDLRAD4 ACER2 C1QL4 LAMP3 ZBTB20 AGAP2 ZP3 OTUD7A RFX3 GJC2 DCLK1 AQP11 GLIS3 TNC AKAP6 DOCK8 ERBB4 FRY KANK3 TMEFF2 CRYM SCIN CORO2B PDE2A NTRK2 PROC SULT2B1 NEURL1 GRM7 CLDN3 WFDC3 ATP8A2 NOS1 SP100 AC019117.3 PTPN22 ZNF141 LEFTY1 DTX1 PPP1R36 GPR68 IL7 KIAA0319 SLC11A1 COL28A1 DNAJC15 BANK1 SH2D1A TTPA LAPTM5 NME5 NPSR1 PTPRR TENM2 OPRM1 CYP7B1 SORCS3 IGSF1 KCNK2 CNTN4 SEMA5A TFAP2D SPINK4 TMEM132D TDRD9 APOH DCC SERPINB11 PTPRT DSCAM IL1RAPL1
GO:0010975	regulation of neuron projection development	4.321156E-04	LYN RELN DMD PAK1 LRP8 RAB11A RAP1A BMP7 PPP3CA TIAM1 PTPRG EPHA3 ROBO2 CACNA1A GDI1 YWHAH MDM2 ROBO1 CCDC88A DENND5A GOLGA4 FGFR1 STK11 PDLIM5 SHTN1 TRAK1 BRAF PTPN9 BCL11A RAPGEF1 NEDD4L LPAR3 UST PLXND1 KANK1 COBL PRKD1 BRSK1 INPP5F NLGN1 NEDD4 CAMK1D PPFIA2 MAGI2 WNT3 NTRK2 ATP8A2 CHODL KIAA0319 ALK TOX SEMA5A DCC DSCAM IL1RAPL1
GO:0050772	positive regulation of axonogenesis	8.291958E-04	PAK1 RAB11A TIAM1 ROBO2 GDI1 ROBO1 GOLGA4 STK11 SHTN1 TRAK1 BRAF BCL11A LPAR3 PLXND1 WNT3 NTRK2 CHODL SEMA5A DSCAM
GO:0048731	system development	8.434058E-04	UNC45B RYR2 GCNT1 UTRN EYA4 LYN RELN CCDC85C LRRTM3 BMP8B HDGFL3 MAMLD1 PKP4 MYO1D MAP1S MEX3C DMD PLCG2 NCL CAPNS1 GIGYF2 RERE PAK1 LRP8 NOTCH2NLA KAZN ZNRF3 POU3F3 NKX2-1 RUNX1 BSN PDE1B RASGRF1 CD80 RAB11A APP CTNNA1 FOXC1 MAPK1 BCR ACTN1 VCL PUS7 HOXB3 RAP1A ABR NOTCH2 BMP7 PPP3CA CDK5RAP2 MAP3K3 AMOTL1 AKAP13 KMT2B PLS1 TIAM1 PTPRG GLI3

			PHLPP1 FZD6 ZFHX3 CEBPA FAM172A HDAC4 SASH1 AHR NEBL FBXL17 FOXF1 EPHA3 IMMP2L WDPCP ROBO2 ZSWIM4 NKX2-3 SLC24A4 CD74 CPAMD8 RYR1 NUP210L CACNA1A OPCML FLT3 LAMA2 FEZF2 HSP90AA1 DNMT1 ACIN1 GNB1 CCNB1 ID2 PSMB2 GDI1 YWHAH BPTF FOXM1 HIF1A UBP1 YY1 FXR1 DDX6 GLRX5 CUL1 ADD1 MDM2 PTPN11 WLS HELLS PDCD4 AUTS2 SPAG9 ROBO1 RBFOX2 NAA15 ACADM NUP133 PCM1 CHD8 CLSTN1 TSG101 B4GALT2 CCDC88A BRCA1 ACO2 SETD1A DENND5A SELENOM TNRC6A GOLGA4 TFDP2 FGFR1 DLX2 ARID1B RC3H2 NXN PDS5B RHBDD3 MBD2 TBCD STK11 EIF2B4 UBE4B NKX2-5 NDE1 ARHGEF2 POR SOS1 ALDH1A2 AP3B1 SRPK2 PDLIM5 EIF2AK2 OGDH ESS2 PDPK1 TCF7L2 ITCH MYSM1 TP53BP1 ROCK2 MEF2A STAT3 EPN2 KDM2B MYEF2 SIRT2 CIT FOXF2 TNRC6B BRCA2 RANBP9 PAPSS1 SH3PXD2A GREB1L SHTN1 DLC1 SMAP1 TCF12 DTNBP1 PPP2R3C ZMYND8 TRAK1 BRAF PCDH9 STRN PTPN9 BCL11A SIRT1 KMT5B MYO9A WWP1 ALKBH1 HS6ST1 ITSN2 CLASP1 RAPGEF1 WWOX MAP2K4 ANKS1A RREB1 MAP2K5 CASP7 BTRC IFT172 HERC1 FOXN3 TBC1D20 TPRN RCAN1 EPB41L5 TMEM131L RNF157 ERRFI1 IFT74 CTNNBIP1 FBXW4 PPARD DOCK11 HES6 LARGE1 NEDD4L TNRC6C LPAR3 PLCB1 MTM1 UST PLXND1 PRKCA LIAS RPS6KA2 TLE2 CELSR1 SPRED2 NPAS2 FOXN2 KANK1 EFNB1 FZD8 P2RX4 ABAT PLAC1 COBL SHROOM3 PRKD1 ADGRV1 SNX10 LEPR BRSK1 IL17D FAXDC2 INPP5F EPHA6 NLGN1 RIN2 OPHN1 FLI1 GRIN2D ACTN2 SALL4 BMPR1B ASXL3 CHST8 MEGF11 SVIL WNT5B NEDD4 MTSS1 CAMK1D ETV6 EDNRA ADAMTS9 PPFIA2 EDA RBM47 MYO3A MAGI2 KALRN WNT3 ESRRB LDLRAD4 ADAM19 KIF26B RNF165 HIVEP3 NHS AGAP2 CHRM3 ZP3 NFASC RFX3 GJC2 DCLK1 AQP11 TNC AKAP6 ERBB4 FRY APLF POU2F2 SNTG2 IGSF10 TMEFF2 SCIN PRICKLE2 PDE2A UNC5A NTRK2 CTNND2 WDR72 NEURL1 ADAMTS6 CLDN3 ATP8A2 NOS1 SP100 PTPN22 LEFTY1 CHODL DTX1 LRRN3 GPR68 HRH2 IL7 KIAA0319 ALK DOK6 MOBP TAFA1 NTM TOX TTPA WNT9B BCAS1 SLC4A10 DNAH5 PCDH15 NME5 ADAM12 PTPRQ TRPM1 MALL HCN1 TENM2 OPRM1 BHLHE22 KCNC2 CYP7B1 RHEX KCNK2 VIT CNTN4 RXFP1 ZFP42 SEMA5A SH3TC2 TFAP2D LINGO2 HSD17B3 KRTAP5-11 CA10 APOH DCC DSCAM NFAM1 IL1RAPL1
GO:0023051	regulation of signaling	1.021111E-03	*SEC14L1 PDE3A RYR2 PID1 DLG2 SORCS2 EYA4 LYN RELN PPEF2 BMP8B DMD RGS3 PAK1 LRP8 NOTCH2NLA ZNRF3 SLC8B1 NKX2-1 RUNX1 GSG1L RASGRF1 CD80 DKK2 RAB11A APP CTNNA1 MAPK1 BCR LGMN PIM3 ARHGAP21 RAP1A ABR NOTCH2 BMP7 PPP3CA MAP3K3 ATXN7 AKAP13 TIAM1 HERC4 GLI3 PHLPP1 FZD6 ARHGEF4 SASH1 FBXL17 ROBO2 RYR3 GPC5 CD74 GRIK4 RYR1 CACNA1A FLT3 LAMA2 NONO HSP90AA1 TMBIM6 PSMB2 SUMO1 GDI1 YWHAH FOXM1 HIF1A CAMTA1 NCOR1 PUM1 MDM2 PTPN11 WLS HELLS NPLOC4 PDCD4 AUTS2 SPAG9 RNF220 ROBO1 FAF1 WDR59 GIPC1 CHD8 CLSTN1 TSG101 WNK2 CCDC88A PREPL BRCA1 TMED4 PIAS1 EHMT1 CHD3 FGFR1 DLX2 RC3H2 NXN RHOQ AIDA GBF1 SELENOS MBD2 STK11 CHEK1 NKX2-5 ARHGEF2 TSPYL2 TELO2 POR SOS1 DENND4B PTPN1 GCLC KCTD10 MAP2K7 BLM RBX1 EIF2AK2 PDPK1 TCF7L2 PIGU ITCH ROCK2 CSNK1G3 CASK STAT3 EPN2 OTUD4 RAB11FIP3 MAP3K2 SLC39A10 CCNY NFAT5 RANBP9 RNF149 EPS15 RPTOR ARHGAP8 DLC1 RNF111 KLHL22 DTNBP1 ZMYND8 BRAF IFT81 BRAP WDFY1 SIRT1 CARD8 TNFRSF10B MYO9A DENND4C RAPGEF1 WWOX MAP2K4 ANKS1A CYTH1 MAP2K5 TRIM14 PIP4K2B BTRC IFT172 USP18 DENND4A RHBDF2 OTUD7B CLEC16A RCAN1 DEPDC5 TMEM131L RNF157 ERRFI1 ARHGEF26 CTNNBIP1 STK39 RALGAPA2 PPARD RHOT1 GAREM1 MCC LPAR3 TBCK PLCB1 MTM1 TRPC1 DSTYK PRKCA TLE2 SPRED2 KANK1 FZD8 P2RX4 ABAT PRKD1 ADGRV1 PLAUR BRSK1 KCNMB4 INPP5F CCDC3 NLGN1 OPHN1 BTBD9 GRIN2D PPM1N ACTN2 SHC2 EYA2 BMPR1B RNF43 SORBS1 WNT5B NEDD4 PPFIA2 EDA ARFGEF3 MAGI2 KALRN WNT3 SYN3 LDLRAD4 C1QL4 RNF165 AGAP2 OTUD7A RFX3 AKAP6 ERBB4 PLCL1 SCG5*

			C1QTNF4 MGLL PDE2A NTRK2 CTNND2 TRAF1 NEURL1 GRM7 NOS1 SP100 PTPN22 LEFTY1 DTX1 DLGAP1 GPR68 HRH2 IL7 KIAA0319 ALK TAFA1 BANK1 SH2D1A LAPTM5 SLC4A10 NME5 ADRA1D PCDH11Y PTPRR OPRM1 KCNC2 UNC13C CYP7B1 SORCS3 IGSF1 CNTN4 SEMA5A SH3TC2 DCC PTPRT CACNG2 NFAM1
GO:0007267	cell-cell signaling	1.024538E-03	*RYR2 DLG2 SORCS2 LYN RELN PKP4 PLCG2 LRP8 ZNRF3 SLC8B1 RUNX1 BSN RASGRF1 DKK2 GABRA6 RAB11A APP MAPK1 BCR NAMPT LGMN PIM3 RAP1A ABR PPP3CA DTNB AMOTL1 TIAM1 GLI3 FZD6 GPC5 GABBR2 GRIK4 CACNA1A LAMA2 KCNN2 GNB1 PSMB2 YWHAH HIF1A CUL1 PTPN11 WLS RNF220 GIPC1 CHD8 CLSTN1 WNK2 PREPL SELENOM TNRC6A FGFR1 ARID1B NXN MBD2 STK11 NKX2-5 LRRFIP2 RBX1 CCNE1 TCF7L2 CSNK1G3 CASK STAT3 RAB11FIP3 TNRC6B CCNY DTNBP1 ZMYND8 BRAF STRN RAPGEF1 WWOX BTRC GABBR1 SLC16A10 FCHSD2 TMEM131L CTNNBIP1 FBXW4 PPARD RHOT1 MCC TNRC6C LPAR3 PLCB1 PRKCA TLE2 CELSR1 KANK1 EFNB1 FZD8 HCN2 P2RX4 ABAT ERC2 BRSK1 KCNMB4 NLGN1 GPR176 OPHN1 BTBD9 GRIN2D PPM1N RNF43 WNT5B PPFIA2 EDA RAB3B MAGI2 WNT3 SYN3 CHRM3 RFX3 GJC2 TNC TRHDE PLCL1 SCG5 PRICKLE2 NTRK2 CTNND2 NEURL1 GRM7 NOS1 DLGAP1 GPR68 HRH2 IL7 SH2D1A WNT9B SLC4A10 ADRA1D GLRA3 PCDH11Y HCRTR2 OPRM1 KCNC2 UNC13C KCNQ3 SORCS3 GABRG3 CNTN4 HTR3D SEMA5A DCC CACNG2 IL1RAPL1*
GO:0060284	regulation of cell development	1.151724E-03	*PDE3A LYN RELN PAK1 LRP8 RAB11A CTNNA1 VCL HOXB3 NOTCH2 BMP7 PPP3CA TIAM1 GLI3 HDAC4 ROBO2 FEZF2 ID2 GDI1 YWHAH HIF1A ADD1 ROBO1 PCM1 GOLGA4 DLX2 STK11 ARHGEF2 ROCK2 SIRT2 SHTN1 TRAK1 BRAF BCL11A RREB1 HES6 LPAR3 PLCB1 PLXND1 KANK1 DOCK5 WNT3 RFX3 GJC2 NEDD9 NTRK2 PROC NEURL1 NOS1 CHODL GPR68 KIAA0319 TTPA OPRM1 SEMA5A DCC DSCAM IL1RAPL1*
GO:0120035	regulation of plasma membrane bounded cell projection organization	1.160142E-03	*LYN RELN DMD PAK1 LRP8 RAB11A RAP1A BMP7 PPP3CA PLS1 TIAM1 PTPRG HDAC4 EPHA3 WDPCP ROBO2 CACNA1A GDI1 YWHAH FXR1 SEPT9 MDM2 AUTS2 ROBO1 CCDC88A DENND5A GOLGA4 FGFR1 RHOQ STK11 PDLIM5 SHTN1 MYO10 ZMYND8 TRAK1 BRAF PTPN9 BCL11A RAPGEF1 RREB1 CROCC DOCK11 NEDD4L LPAR3 UST PLXND1 KANK1 COBL PRKD1 BRSK1 INPP5F NLGN1 NEDD4 CAMK1D PPFIA2 MAGI2 WNT3 NTRK2 NEURL1 ATP8A2 CHODL KIAA0319 ALK TOX TENM2 SEMA5A DCC DSCAM IL1RAPL1*
GO:0007275	multicellular organism development	1.262741E-03	*UNC45B RYR2 GCNT1 UTRN EYA4 LYN RELN CCDC85C LRRTM3 BMP8B HDGFL3 MAMLD1 PKP4 MYO1D MAP1S MEX3C DMD PLCG2 NCL CAPNS1 GIGYF2 RERE PAK1 LRP8 NOTCH2NLA KAZN ZNRF3 POU3F3 NKX2-1 RUNX1 BSN PDE1B RASGRF1 CD80 DKK2 RAB11A APP CTNNA1 ZFR FOXC1 MAPK1 BCR ACTN1 VCL PUS7 HOXB3 RAP1A ABR NOTCH2 BMP7 PPP3CA CDK5RAP2 MAP3K3 AMOTL1 AKAP13 KMT2B PLS1 TIAM1 GTF2IRD1 PTPRG GLI3 PHLPP1 FZD6 ZFHX3 CEBPA FAM172A HDAC4 SASH1 AHR NEBL FBXL17 FOXF1 EPHA3 IMMP2L WDPCP ROBO2 ZSWIM4 NKX2-3 SLC24A4 CD74 CPAMD8 RYR1 NUP210L CACNA1A OPCML FLT3 LAMA2 FEZF2 HSP90AA1 DNMT1 ACIN1 GNB1 CCNB1 ID2 PSMB2 GDI1 YWHAH BPTF FOXM1 HIF1A UBP1 YY1 FXR1 DDX6 GLRX5 CUL1 ADD1 MDM2 PTPN11 WLS HELLS PDCD4 AUTS2 SPAG9 ROBO1 RBFOX2 NAA15 ACADM NUP133 PCM1 CHD8 ASF1B CLSTN1 TSG101 B4GALT2 CCDC88A BRCA1 ACO2 SETD1A DENND5A SELENOM TNRC6A EHMT1 GOLGA4 TFDP2 FGFR1 DLX2 ARID1B RC3H2 NXN AIDA PDS5B RHBDD3 MBD2 TBCD STK11 EIF2B4 UBE4B CHEK1 NKX2-5 NDE1 ARHGEF2 PHF3 POR SOS1 ALDH1A2 IDE AP3B1 SRPK2 PDLIM5 EIF2AK2 OGDH ESS2 PDPK1 TCF7L2 ITCH MYSM1 SCMH1 TP53BP1 ROCK2 MEF2A STAT3 EPN2 KDM2B MYEF2 SIRT2 CIT FOXF2 UBR3 TNRC6B BRCA2 RANBP9 PAPSS1 SH3PXD2A GREB1L SHTN1 DLC1 OFD1 RNF111 TAF8 SMAP1 TCF12 DTNBP1 PPP2R3C ZMYND8 TRAK1 BRAF PCDH9 STRN PTPN9 BCL11A*

			SIRT1 KMT5B MYO9A WWP1 ALKBH1 HS6ST1 ITSN2 CLASP1 RAPGEF1 WWOX MAP2K4 ANKS1A RREB1 MAP2K5 CASP7 FUT8 BTRC IFT172 HERC1 FOXN3 TBC1D20 TPRN OTUD7B RCAN1 EPB41L5 TMEM131L RNF157 ERRFI1 IFT74 CTNNBIP1 FBXW4 TET1 PPARD DOCK11 HES6 LARGE1 NEDD4L TNRC6C CREM LPAR3 PLCB1 DIAPH2 MTM1 UST PLXND1 PRKCA ZNF875 LIAS RPS6KA2 TLE2 CELSR1 SPRED2 NPAS2 FOXN2 KANK1 EFNB1 FZD8 P2RX4 ABAT SP8 PLAC1 COBL SHROOM3 PRKD1 ADGRV1 SNX10 LEPR BRSK1 IL17D FAXDC2 INPP5F EPHA6 NLGN1 RIN2 OPHN1 FLI1 GRIN2D ACTN2 SALL4 EYA2 BMPR1B RNF43 HUNK ASXL3 CHST8 MEGF11 SVIL WNT5B NEDD4 MTSS1 CAMK1D ETV6 EDNRA ADAMTS9 PPFIA2 EDA RBM47 MYO3A MAGI2 KALRN WNT3 ESRRB LDLRAD4 ADAM19 KIF26B RNF165 HIVEP3 NHS AGAP2 CHRM3 ZP3 NFASC RFX3 GJC2 DCLK1 AQP11 TNC AKAP6 CDH18 ERBB4 FRY APLF POU2F2 SNTG2 IGSF10 TMEFF2 SCIN PRICKLE2 PDE2A UNC5A NTRK2 CTNND2 PROC WDR72 SULT2B1 NEURL1 ADAMTS6 CLDN3 ATP8A2 NOS1 SP100 PTPN22 ZNF141 LEFTY1 CHODL DTX1 LRRN3 C6ORF58 GPR68 HRH2 IL7 KIAA0319 ALK DOK6 MOBP TAFA1 NTM SLC2A14 TOX TTPA WNT9B BCAS1 SLC4A10 DNAH5 PCDH15 NME5 ADAM12 PTPRQ TRPM1 EVX2 PTPRR MALL HCN1 TENM2 OPRM1 BHLHE22 KCNC2 CYP7B1 RHEX KCNK2 VIT CNTN4 RXFP1 ZFP42 SEMA5A SH3TC2 TFAP2D LINGO2 HSD17B3 KRTAP5-11 TDRD9 CA10 APOH DCC DSCAM NFAM1 IL1RAPL1
GO:0010646	regulation of cell communication	1.354868E-03	*SEC14L1 PDE3A RYR2 PID1 DLG2 SORCS2 EYA4 LYN RELN PPEF2 BMP8B DMD RGS3 PAK1 LRP8 NOTCH2NLA ZNRF3 SLC8B1 NKX2-1 RUNX1 GSG1L RASGRF1 CD80 DKK2 RAB11A APP CTNNA1 MAPK1 BCR LGMN PIM3 ARHGAP21 RAP1A ABR NOTCH2 BMP7 PPP3CA MAP3K3 ATXN7 AKAP13 TIAM1 HERC4 GLI3 PHLPP1 FZD6 ARHGEF4 SASH1 FBXL17 ROBO2 GPC5 CD74 GRIK4 CACNA1A FLT3 LAMA2 NONO HSP90AA1 TMBIM6 PSMB2 SUMO1 GDI1 YWHAH FOXM1 HIF1A CAMTA1 NCOR1 PUM1 MDM2 PTPN11 WLS HELLS NPLOC4 PDCD4 AUTS2 SPAG9 RNF220 ROBO1 FAF1 WDR59 GIPC1 CHD8 CLSTN1 TSG101 WNK2 CCDC88A PREPL BRCA1 TMED4 PIAS1 EHMT1 CHD3 FGFR1 DLX2 RC3H2 NXN RHOQ AIDA GBF1 SELENOS MBD2 STK11 CHEK1 NKX2-5 ARHGEF2 TSPYL2 TELO2 POR SOS1 DENND4B PTPN1 GCLC KCTD10 MAP2K7 BLM RBX1 EIF2AK2 PDPK1 TCF7L2 PIGU ITCH ROCK2 CSNK1G3 CASK STAT3 EPN2 OTUD4 RAB11FIP3 MAP3K2 SLC39A10 CCNY NFAT5 RANBP9 RNF149 EPS15 RPTOR ARHGAP8 DLC1 RNF111 KLHL22 DTNBP1 ZMYND8 BRAF IFT81 BRAP WDFY1 SIRT1 CARD8 TNFRSF10B MYO9A DENND4C RAPGEF1 WWOX MAP2K4 ANKS1A CYTH1 MAP2K5 TRIM14 PIP4K2B BTRC IFT172 USP18 DENND4A RHBDF2 OTUD7B CLEC16A RCAN1 DEPDC5 TMEM131L RNF157 ERRFI1 ARHGEF26 CTNNBIP1 STK39 RALGAPA2 PPARD RHOT1 GAREM1 MCC LPAR3 TBCK PLCB1 MTM1 DSTYK PRKCA TLE2 SPRED2 KANK1 FZD8 P2RX4 ABAT PRKD1 ADGRV1 PLAUR BRSK1 KCNMB4 INPP5F CCDC3 NLGN1 OPHN1 BTBD9 GRIN2D PPM1N ACTN2 SHC2 EYA2 BMPR1B RNF43 SORBS1 WNT5B NEDD4 PPFIA2 EDA ARFGEF3 MAGI2 KALRN WNT3 SYN3 LDLRAD4 C1QL4 RNF165 AGAP2 OTUD7A RFX3 AKAP6 ERBB4 PLCL1 SCG5 C1QTNF4 MGLL PDE2A NTRK2 CTNND2 TRAF1 NEURL1 GRM7 NOS1 SP100 PTPN22 LEFTY1 DTX1 DLGAP1 GPR68 HRH2 IL7 KIAA0319 ALK TAFA1 BANK1 SH2D1A LAPTM5 SLC4A10 NME5 ADRA1D PCDH11Y PTPRR OPRM1 KCNC2 UNC13C CYP7B1 SORCS3 IGSF1 CNTN4 SEMA5A SH3TC2 DCC PTPRT CACNG2 NFAM1*
GO:0048589	developmental growth	1.385676E-03	*MEX3C DMD GIGYF2 PAK1 RAB11A APP FOXC1 MAPK1 VCL LGMN NOTCH2 PPP3CA AKAP13 PLS1 TIAM1 GLI3 HSP90AA1 CCNB1 GDI1 YY1 ADD1 PTPN11 AUTS2 SPAG9 SELENOM GOLGA4 FGFR1 RC3H2 STK11 CHEK1 NKX2-5 POR SOS1 PDLIM5 STAT3 BRCA2 SHTN1 TAF8 BCL11A ALKBH1 ITSN2 MAP2K4 EPB41L5 RNF157 PPARD LARGE1 NEDD4L LPAR3 PLCB1 MTM1 COBL LEPR SALL4 BMPR1B MAGI2 WNT3 KIF26B ZP3 DCLK1*

			TNC AKAP6 ERBB4 EYS ATP8A2 IL7 KIAA0319 SLC4A10 PCDH15 KCNK2 SEMA5A DCC DSCAM
GO:0031344	regulation of cell projection organization	1.459608E-03	*LYN RELN DMD PAK1 LRP8 RAB11A RAP1A BMP7 PPP3CA PLS1 TIAM1 PTPRG HDAC4 EPHA3 WDPCP ROBO2 CACNA1A GDI1 YWHAH FXR1 SEPT9 MDM2 AUTS2 ROBO1 CCDC88A DENND5A GOLGA4 FGFR1 RHOQ STK11 PDLIM5 SHTN1 MYO10 ZMYND8 TRAK1 BRAF PTPN9 BCL11A MYO9A RAPGEF1 RREB1 CROCC DOCK11 NEDD4L LPAR3 UST PLXND1 KANK1 COBL PRKD1 BRSK1 INPP5F NLGN1 NEDD4 CAMK1D PPFIA2 MAGI2 WNT3 NTRK2 NEURL1 ATP8A2 CHODL KIAA0319 ALK TOX TENM2 SEMA5A DCC DSCAM IL1RAPL1*
GO:0070727	cellular macromolecule localization	1.961560E-03	*RYR2 PID1 DLG2 SORCS2 DPP10 RELN DMD PAK1 GSG1L RAB11A CTNNA1 EPB41 MAPK1 NUP98 BCR OAZ2 VCL RAP1A BMP7 PPP3CA SNX27 STX8 PLS1 TIAM1 GLI3 SYNE1 RFTN1 EPHA3 IMMP2L GPC5 SGSM1 CD74 CACNA1A HSP90AA1 RPL35A SUMO1 UBE2D2 IPO7 IPO5 GDI1 ELAVL1 YWHAH NPEPPS MTCH2 GGA2 SLBP SRP68 EXOSC10 SEPT9 MDM2 PTPN11 WLS NPLOC4 SARNP CDCA5 NUP133 PCM1 GIPC1 POLDIP3 TFDP1 COPB1 CLSTN1 TSG101 CCDC88A PREPL AP1G1 SEPT6 TERF2 HDAC8 HK1 TMED4 TM9SF3 SCP2 GTSE1 GOLGA4 TFDP2 RHOQ GBF1 SELENOS STK11 ARHGEF2 PTPN1 IDE AP3B1 WAPL ANKRD13C PDPK1 CCNE1 TCF7L2 PIGU ARFGAP3 ROCK2 STAT3 SNUPN USP4 CENPA RAB11FIP3 BRCA2 VPS37B EPS15 WRAP53 CDC40 RABGAP1L TAF8 PEX14 ZMYND8 TRAK1 CHMP4A BRAF IFT81 ASB3 KNL1 PTPN9 SIL1 DENND4C KIAA0753 SMG6 IFT172 CROCC OTUD7B RTN2 XPO4 IFT74 ABHD17C NEDD4L TBCK TBC1D22A CLTCL1 TBC1D5 CELSR1 AP4S1 PRKD1 SNX10 FBXO4 NLGN1 OPHN1 ACTN2 SORBS1 NEDD4 KCNB2 EFR3B RAB3B MAGI2 RILPL2 FAM53A LAMP3 NFASC DCLK1 AQP11 AKAP6 ANK1 ERBB4 SCG5 SCIN WDR72 SP100 PTPN22 KCNIP4 RGPD3 DNAJC15 CD247 LAPTM5 TRPM1 SH3TC2 DAO CACNG2*
GO:0034613	cellular protein localization	2.216764E-03	*RYR2 PID1 DLG2 SORCS2 DPP10 RELN DMD PAK1 GSG1L RAB11A CTNNA1 EPB41 MAPK1 NUP98 BCR OAZ2 VCL RAP1A BMP7 PPP3CA SNX27 STX8 PLS1 TIAM1 GLI3 SYNE1 RFTN1 EPHA3 IMMP2L GPC5 SGSM1 CD74 CACNA1A HSP90AA1 RPL35A SUMO1 UBE2D2 IPO7 IPO5 GDI1 ELAVL1 YWHAH NPEPPS MTCH2 GGA2 SLBP SRP68 SEPT9 MDM2 PTPN11 WLS NPLOC4 SARNP CDCA5 NUP133 PCM1 GIPC1 POLDIP3 TFDP1 COPB1 CLSTN1 TSG101 CCDC88A PREPL AP1G1 SEPT6 TERF2 HDAC8 HK1 TMED4 TM9SF3 SCP2 GTSE1 GOLGA4 TFDP2 RHOQ GBF1 SELENOS STK11 ARHGEF2 PTPN1 IDE AP3B1 WAPL ANKRD13C PDPK1 CCNE1 TCF7L2 PIGU ARFGAP3 ROCK2 STAT3 SNUPN USP4 CENPA RAB11FIP3 BRCA2 VPS37B EPS15 WRAP53 CDC40 RABGAP1L TAF8 PEX14 ZMYND8 TRAK1 CHMP4A BRAF IFT81 ASB3 KNL1 PTPN9 SIL1 DENND4C KIAA0753 SMG6 IFT172 CROCC OTUD7B RTN2 XPO4 IFT74 ABHD17C NEDD4L TBCK TBC1D22A CLTCL1 TBC1D5 CELSR1 AP4S1 PRKD1 SNX10 FBXO4 NLGN1 OPHN1 ACTN2 SORBS1 NEDD4 KCNB2 EFR3B RAB3B MAGI2 RILPL2 FAM53A LAMP3 NFASC DCLK1 AQP11 AKAP6 ANK1 ERBB4 SCG5 SCIN WDR72 SP100 PTPN22 KCNIP4 RGPD3 DNAJC15 CD247 LAPTM5 TRPM1 SH3TC2 DAO CACNG2*
GO:0016358	dendrite development	2.329500E-03	*RELN MAP1S RERE LRP8 APP BMP7 PPP3CA TIAM1 CACNA1A FEZF2 YWHAH RBFOX2 ARID1B STK11 PDLIM5 MEF2A DTNBP1 ZMYND8 TRAK1 STRN BCL11A NEDD4L COBL NLGN1 NEDD4 CAMK1D PPFIA2 DCLK1 CTNND2 NEURL1 KIAA0319 ALK DCC DSCAM IL1RAPL1*
GO:0007409	axonogenesis	2.688656E-03	*RELN MAP1S PAK1 NKX2-1 RAB11A APP MAPK1 VCL NOTCH2 BMP7 TIAM1 GLI3 EPHA3 ROBO2 ZSWIM4 CACNA1A LAMA2 FEZF2 HSP90AA1 GDI1 PTPN11 AUTS2 ROBO1 GOLGA4 STK11 SOS1 RANBP9 SHTN1 TRAK1 BRAF BCL11A LPAR3 UST PLXND1 PRKCA EFNB1 COBL BRSK1 EPHA6 OPHN1 BMPR1B WNT3 RNF165 NFASC DCLK1 UNC5A NTRK2 ATP8A2 CHODL KIAA0319 DOK6 CNTN4 SEMA5A DCC DSCAM*

GO:0045597	positive regulation of cell differentiation	2.750355E-03	*PDE3A AAMDC LYN RELN DMD PAK1 LRP8 RUNX1 CD80 RAB11A CTNNA1 FOXC1 RAP1A NOTCH2 BMP7 TIAM1 GLI3 ZFHX3 CEBPA ROBO2 CD74 FEZF2 ACIN1 ID2 GDI1 HIF1A ADD1 MDM2 SPAG9 ROBO1 PIAS1 GOLGA4 FGFR1 DLX2 STK11 NKX2-5 ARHGEF2 POR AP3B1 TCF7L2 MEF2A STAT3 SIRT2 SHTN1 SMAP1 TCF12 PPP2R3C TRAK1 BRAF BCL11A SIRT1 RREB1 CTNNBIP1 PPARD LPAR3 PLCB1 PLXND1 PRKCA DOCK5 PRKD1 FAXDC2 CCDC3 WDFY2 RIN2 BMPR1B WNT5B ADAMTS9 WNT3 RFX3 GJC2 AKAP6 SCIN NEDD9 NTRK2 PROC SULT2B1 NEURL1 CHODL GPR68 IL7 TOX OPRM1 RHEX SEMA5A DSCAM IL1RAPL1*
GO:0016043	cellular component organization	3.331861E-03	*HFM1 PDE3A PID1 UTRN DLG2 EYA4 LYN RELN LRRTM3 EIF3E ADAMTS8 TXNL4A HDGFL3 PKP4 BCORL1 MYO1D MAP1S DMD PLCG2 CAPNS1 RERE PAK1 LRP8 EPB41L4B NKX2-1 RUNX1 BSN TEX14 GSG1L RASGRF1 RAB11A APP CTNNA1 STAG2 EPB41 FOXC1 MAPK1 NUP98 MICOS10 BCR ACTN1 VCL LGMN KDM2A ARHGAP21 RAP1A ABR NOTCH2 BMP7 PPP3CA CDK5RAP2 AMOTL1 ATXN7 ABCC1 AKAP13 KMT2B STX8 TRAPPC11 PLS1 TIAM1 SLC12A9 PTPRG GLI3 ARHGEF4 CEBPA FAM172A HDAC4 SYNE1 RFTN1 NEBL FOXF1 EPHA3 IMMP2L WDPCP ROBO2 RYR3 ZSWIM4 CD74 RYR1 CACNA1A LAMA2 FEZF2 PRMT8 HSP90AA1 TUBA1C ST13 ATP5MC2 DNMT1 SMC1A SNRPD1 ACIN1 CCNB1 CLNS1A SUMO1 UBE2D2 CDC123 MRPL12 GDI1 ELAVL1 CDC27 YWHAH NPEPPS NDUFA12 BPTF FOXM1 FAM50A EIF3J HIF1A NCOR1 YY1 FXR1 DDX6 GLRX5 EXOSC10 CUL1 SEPT9 ADD1 MDM2 PTPN11 MRPL15 HELLS NPLOC4 BAZ1A DNAJC2 UHRF1 CHAF1A TRAPPC5 AUTS2 SPAG9 ROBO1 RBFOX2 NSD1 CDCA5 PPP1R12A FAF1 NUP133 PCM1 CHD8 TFDP1 ASF1B CLSTN1 TSG101 ATAD3B CCDC88A CDC23 AP1G1 BRCA1 SEPT6 TERF2 CNOT6 PDS5A NFU1 HDAC8 SETD1A DENND5A ESYT1 TUBB6 TMED4 SCP2 PIAS1 EHMT1 GOLGA4 TFDP2 CHD3 FGFR1 ARID1B GAPVD1 RHOQ AIDA SENP6 GBF1 PDS5B MTFR1 MBD2 TBCD STK11 UBE4B NAA60 ATP8B2 CHEK1 NKX2-5 RNF4 NDE1 PDE4DIP ARHGEF2 TSPYL2 TELO2 SOS1 ALDH1A2 PTPN1 GCLC IDE MTA3 KCTD10 AP3B1 MDN1 MAP2K7 SRPK2 BLM RBX1 PDLIM5 EIF2AK2 WAPL ATF7IP PDPK1 CCNE1 TCF7L2 ITGAE ITCH PLEKHM2 MYSM1 SCMH1 ARFGAP3 TP53BP1 DMAC2 PSMG2 LSM3 STMP1 ROCK2 MEF2A STAT3 EPN2 KDM2B SNUPN USP4 CENPA SIRT2 CIT FOXF2 RAP1GDS1 BRCA2 RBL2 VPS37B KANSL2 ASCC3 POLE2 RANBP9 EPS15 WRAP53 STAG1 SH3PXD2A RPTOR SHTN1 MYO10 DLC1 OFD1 KLHL22 TAF8 SMAP1 DTNBP1 REEP3 PPP2R3C PEX14 ZMYND8 TRAK1 CHMP4A BRAF IFT81 KNL1 STRN PTPN9 BCL11A SIRT1 KMT5B MYO9A ALKBH1 ITSN2 KDM4B CLASP1 RAPGEF1 RREB1 MAP2K5 PRIM2 PIP4K2B GULP1 KIAA0753 SMG6 NDUFAF6 IFT172 HERC1 CROCC FCHSD2 TBC1D20 TPRN CLEC16A ATL3 KAT14 EPB41L5 CNOT6L RNF157 SPIRE2 IFT74 ARHGEF26 CTNNBIP1 TET1 FBF1 PPARD DOCK11 DMAC2L ABHD17C MTRF1 RHOT1 RFX2 NEDD4L FAM161A LPAR3 TBCK PLCB1 DIAPH2 JADE3 MTM1 UST PLXND1 PLSCR3 CEP76 TMCC1 CLTCL1 PRKCA ATP9A TBC1D5 RPS6KA2 TLE2 CELSR1 KANK1 EFNB1 ZNFX1 IQCG KANSL1L COBL SHROOM3 ERC2 LRRC8C PRKD1 ADGRV1 SNX10 USP44 PLAUR BRSK1 INPP5F EPHA6 FBXO4 NLGN1 PLEKHA7 OPHN1 EPB41L4A BTBD9 ACTN2 EYA2 BMPR1B SORBS1 SVIL CADM2 NEDD4 MTSS1 STON1 CAMK1D ETV6 CDA KCNB2 ADAMTS9 PPFIA2 ARFGEF3 RAB3B MAGI2 WNT3 LDLRAD4 LRRTM4 ADAM19 RILPL2 RNF165 AGAP2 NFASC RFX3 DCLK1 AQP11 TNC AKAP6 ANK1 CDH18 CNTD1 ERBB4 FRY KANK3 AL133500.1 TMEFF2 NLRC4 SCIN REPS2 FRMD3 CORO2B PDE2A NEDD9 UNC5A NTRK2 CTNND2 TNXB WDR72 TRAF1 MEI1 NEURL1 CFAP61 ADAMTS6 CLDN3 ATP8A2 CCDC36 NOS1 SP100 CHODL LRRN3 KIAA0319 ALK COL28A1 DOK6 DNAJC15 TERB2 MOBP TOX TTPA LAPTM5 DNAH5 PCDH15 NME5 ADAM12 GLRA3 TRPM1 HCN1 TENM2 KCNC2 UNC13C DNAH9 PADI4 SYCP1 ATP6V1G3 VIT CNTN4 RXFP1 SEMA5A SH3TC2 LINGO2 DAO TDRD9 DCC PTPRT DSCAM CACNG2 IL1RAPL1*

GO:0061564	axon development	4.252790E-03	*RELN MAP1S PAK1 NKX2-1 RAB11A APP CTNNA1 MAPK1 VCL NOTCH2 BMP7 TIAM1 GLI3 EPHA3 ROBO2 ZSWIM4 CACNA1A LAMA2 FEZF2 HSP90AA1 GDI1 PTPN11 AUTS2 ROBO1 GOLGA4 STK11 SOS1 RANBP9 SHTN1 TRAK1 BRAF BCL11A LPAR3 UST PLXND1 PRKCA EFNB1 COBL BRSK1 INPP5F EPHA6 OPHN1 BMPR1B WNT3 RNF165 NFASC DCLK1 TNC UNC5A NTRK2 ATP8A2 CHODL KIAA0319 DOK6 CNTN4 SEMA5A DCC DSCAM*
GO:0060341	regulation of cellular localization	5.409873E-03	*RYR2 PID1 DPP10 LYN DMD PAK1 GSG1L RAB11A CTNNA1 EPB41 MAPK1 OAZ2 PIM3 RAP1A STX8 PLS1 TIAM1 GLI3 FOXF1 EPHA3 GPC5 HSP90AA1 TMBIM6 IPO5 GDI1 YWHAH NPEPPS HIF1A MDM2 PTPN11 CDCA5 PPP1R12A PCM1 TFDP1 TSG101 CCDC88A HDAC8 SCP2 GTSE1 TFDP2 RHOQ GBF1 STK11 PTPN1 WAPL PDPK1 CCNE1 TCF7L2 ROCK2 WRAP53 ZMYND8 PTPN9 CROCC TBC1D20 OTUD7B XPO4 PPARD ABHD17C RHOT1 NEDD4L TRPC1 P2RX4 PRKD1 BRSK1 INPP5F FBXO4 NLGN1 OPHN1 ACTN2 SORBS1 NEDD4 MAGI2 DCLK1 AKAP6 ERBB4 NOS1 SP100 PTPN22 GPR68 CD247 NPSR1 SH3TC2 CACNG2*
GO:0044260	cellular macromolecule metabolic process	6.900222E-03	*HFM1 INTS4 ALG9 AP001781.2 GCNT1 PID1 PMF1 EYA4 LYN RELN EIF3E PPEF2 GALNT1 BMP8B HDGFL3 USP53 MAMLD1 MAP1S MEX3C DMD PLCG2 ZNF30 RGS3 NCL GIGYF2 RERE PAK1 LRP8 ZNRF3 POU3F3 RNF150 NKX2-1 GTF2IRD2 RUNX1 TEX14 GSG1L CD80 RAB11A APP FOXC1 MAPK1 NUP98 BCR OTUD5 NAMPT ZNF282 NAA25 LGMN KDM2A PIM3 PUS7 HOXB3 RAP1A ABR NOTCH2 BMP7 OGG1 PPP3CA CDK5RAP2 ZNF131 MAP3K3 ATXN7 THADA AKAP13 KMT2B USP13 PHKB TIAM1 GTF2IRD1 PTPRG HERC4 GLI3 PHLPP1 FZD6 ZFHX3 CEBPA FAM172A HDAC4 SASH1 AHR FBXL17 FOXF1 EPHA3 IMMP2L ESRRG NKX2-3 CD74 CPAMD8 STON1-GTF2A1L FLT3 FEZF2 HDGFL1 PRMT8 NONO HSP90AA1 RPL35A ILF3 TMBIM6 DNMT1 HNRNPR SMC1A PPA1 UBE2M CCNB1 ID2 ARIH1 PSMB2 SUMO1 UBE2D2 TAF1D IPO7 CDC123 CBS IPO5 MRPL12 SSU72 ELAVL1 CDC27 YWHAH NPEPPS GGA2 SND1 SLBP BPTF FOXM1 EIF3J HIF1A UBP1 CAMTA1 INTS8 NCOR1 DARS2 PUM1 YY1 FXR1 DDX6 GTF2F1 EXOSC10 KTN1 UBE2V2 CUL1 ADD1 MDM2 PTPN11 MRPL15 HELLS NPLOC4 BAZ1A DNAJC2 UHRF1 PDCD4 CHAF1A AUTS2 SPAG9 SARNP RNF220 ROBO1 RBFOX2 NAA15 ACADM NSD1 CDCA5 PPP1R12A FAF1 NUP133 SEPHS1 GIPC1 CHD8 POLDIP3 TFDP1 TSG101 B4GALT2 DPH1 WNK2 CCDC88A CDC23 PPP1R7 BRCA1 TERF2 CNOT6 RNF130 PDS5A HDAC8 SETD1A TNRC6A MLXIP SSBP4 PIAS1 EHMT1 GALNT2 TFDP2 CHD3 TRMT1 FGFR1 LRRC41 DLX2 ARID1B RC3H2 NXN PPME1 RHOQ AIDA SENP6 PDS5B SELENOS MBD2 STK11 EIF2B4 UBE4B NAA60 CHEK1 ICE2 NKX2-5 RNF4 ARHGEF2 UNKL PHF3 TSPYL2 TELO2 LRRFIP2 POR FBXO28 PTPN1 GCLC IDE MTA3 KCTD10 AP3B1 MAP2K7 SRPK2 BLM RBX1 METTL6 RPAP2 EIF2AK2 WAPL OGDH ATF7IP PRMT3 PDPK1 CCNE1 NEK4 TCF7L2 TFCP2 PIGU ITCH MYSM1 SCMH1 TP53BP1 DMAC2 LSM3 ROCK2 MEF2A CSNK1G3 CASK STAT3 POLR3G KDM2B OTUD4 MYEF2 USP4 SIRT2 CIT ZNF410 FOXF2 UBR3 TNRC6B MAP3K2 BRCA2 HS6ST2 RBL2 SLC39A10 VPS37B CCNY NFAT5 KANSL2 ASCC3 POLE2 RNF149 EPS15 WRAP53 ZXDC RNF121 RPTOR DLC1 USP32 WDHD1 RNF111 KLHL22 TAF8 TCF12 DTNBP1 PPP2R3C PEX14 ZMYND8 TRAK1 GFPT1 BRAF PIGX HECTD4 GTF2E2 BRAP TULP4 ASB3 PTPN9 BCL11A SIRT1 CARD8 TNFRSF10B PKN3 KMT5B WWP1 ALKBH1 DSE PTPN4 HS6ST1 ZNF143 KDM4B RAPGEF1 ZNF263 WWOX MAP2K4 RREB1 MAP2K5 TRIM14 PRIM2 FUT8 EEFSEC RFX1 BTRC DCAF10 SMG6 LIN52 HERC1 USP18 FOXN3 DENND4A CHFR PHKA1 OTUD7B CLEC16A PIGN ZNF620 KAT14 RCAN1 CDK17 CNOT6L OXR1 QRSL1 RNF157 ERRFI1 XXYLT1 SPIRE2 IFT74 SRBD1 CTNNBIP1 DNASE1 STK39 ACSL1 FBXW4 TET1 MED12L NFIX MOB1B PPARD ABHD17C HES6 RPS6KC1 MTRF1 LARGE1 RHOT1 RFX2 NEDD4L TNRC6C FAM161A CREM LPAR3 SNTA1 TBCK PALD1 PLCB1 JADE3 MTM1 HIVEP1 UST PLXND1 POLH DSTYK GMEB2 PRKCA EEF1AKMT2 ZNF875 TBC1D5 LIAS NEDD8-*

			MDP1 PRDM10 RPS6KA2 TLE2 SPRED2 NPAS2 FOXN2 PKNOX2 FZD8 SP8 ZNF283 ZNFX1 KANSL1L FAM220A PROS1 JAZF1 PRKD1 USP44 LEPR TNFAIP8 PLAUR ZNF616 BRSK1 FAXDC2 INPP5F EPHA6 WDFY2 FBXO4 OPHN1 FLI1 PPM1N SHC2 SALL4 EYA2 BMPR1B ZNF713 RNF43 HUNK ASXL3 CHST8 SORBS1 MDP1 CPEB2 NEDD4 CAMK1D PPP1R3B ETV6 ADAMTS9 AC092647.5 EDA RAB3B MYO3A INKA2 MAGI2 KALRN AC092835.1 SEPSECS ESRRB LDLRAD4 ACER2 RNF165 HIVEP3 ZNF790 LAMP3 ZBTB20 AGAP2 CHRM3 ZP3 OTUD7A KHDC1 RFX3 GJC2 DCLK1 AQP11 USP43 GLIS3 TNC CNTD1 ERBB4 FRY PLCL1 APLF POU2F2 AL133500.1 CRYM NLRC4 ZNF331 GALNT14 PDE2A NEDD9 NTRK2 TNXB FBXL13 PROC AC068896.1 TRAF1 AP001273.2 NEURL1 CLDN3 HS3ST5 WFDC3 CCDC36 NOS1 AC004687.2 SP100 AC019117.3 PTPN22 ZNF141 LEFTY1 DTX1 SLCO3A1 PPP1R36 IL7 ALK ZNF43 SLC11A1 COL28A1 NDST3 BANK1 TOX HS3ST4 PPP1R3A WNT9B LAPTM5 LRRC2 AC013717.1 DIO2 ASMT LYZ PTPRQ SLFN13 EVX2 PTPRR TENM2 BHLHE22 HSF5 AC058822.1 IGSF1 PADI4 SYCP1 KCNK2 ZFP42 TFAP2D SPINK4 MGAT4C TDRD9 SERPINB11 ARRDC5 AC008758.5 NLRP2 PTPRT CACNG2 NFAM1
GO:0048856	anatomical structure development	7.098369E-03	*UNC45B PDE3A RYR2 GCNT1 PID1 UTRN EYA4 LYN RELN CCDC85C LRRTM3 BMP8B HDGFL3 MAMLD1 PKP4 MYO1D MAP1S MEX3C DMD PLCG2 NCL CAPNS1 GIGYF2 RERE PAK1 LRP8 NOTCH2NLA KAZN ZNRF3 POU3F3 NKX2-1 RUNX1 BSN PDE1B RASGRF1 CD80 DKK2 RAB11A APP CTNNA1 ZFR EPB41 FOXC1 MAPK1 BCR ACTN1 VCL PUS7 HOXB3 RAP1A ABR NOTCH2 BMP7 PPP3CA CDK5RAP2 MAP3K3 AMOTL1 AKAP13 KMT2B PLS1 TIAM1 GTF2IRD1 PTPRG GLI3 PHLPP1 FZD6 ZFHX3 CEBPA FAM172A HDAC4 SYNE1 SASH1 AHR NEBL FBXL17 FOXF1 EPHA3 IMMP2L WDPCP ROBO2 ZSWIM4 STS NKX2-3 SLC24A4 CD74 CPAMD8 RYR1 NUP210L CACNA1A OPCML FLT3 LAMA2 FEZF2 HSP90AA1 DNMT1 ACIN1 GNB1 CCNB1 ID2 PSMB2 SUMO1 GDI1 YWHAH BPTF FOXM1 HIF1A UBP1 YY1 FXR1 DDX6 GLRX5 CUL1 ADD1 MDM2 PTPN11 WLS HELLS PDCD4 AUTS2 SPAG9 ROBO1 RBFOX2 NAA15 ACADM NUP133 PCM1 CHD8 TFDP1 ASF1B CLSTN1 TSG101 B4GALT2 CCDC88A BRCA1 ACO2 SETD1A DENND5A SELENOM TNRC6A PIAS1 EHMT1 GOLGA4 TFDP2 FGFR1 DLX2 ARID1B RC3H2 NXN RHOQ AIDA PDS5B RHBDD3 MBD2 TBCD STK11 EIF2B4 UBE4B CHEK1 NKX2-5 NDE1 ARHGEF2 PHF3 POR SOS1 ALDH1A2 IDE AP3B1 SRPK2 PDLIM5 EIF2AK2 OGDH ESS2 PDPK1 TCF7L2 ITCH MYSM1 SCMH1 TP53BP1 ROCK2 MEF2A STAT3 EPN2 KDM2B MYEF2 SIRT2 CIT FOXF2 UBR3 TNRC6B BRCA2 RANBP9 PAPSS1 SH3PXD2A GREB1L SHTN1 MYO10 DLC1 OFD1 RNF111 TAF8 SMAP1 TCF12 DTNBP1 PPP2R3C ZMYND8 TRAK1 BRAF PCDH9 KNL1 STRN PTPN9 BCL11A SIRT1 KMT5B MYO9A WWP1 ALKBH1 HS6ST1 ITSN2 CLASP1 RAPGEF1 WWOX MAP2K4 ANKS1A RREB1 MAP2K5 CASP7 FUT8 BTRC IFT172 HERC1 FOXN3 TBC1D20 TPRN OTUD7B RCAN1 EPB41L5 TMEM131L RNF157 ERRFI1 IFT74 ARHGEF26 CTNNBIP1 FBXW4 TET1 PPARD DOCK11 HES6 LARGE1 RFX2 NEDD4L TNRC6C CREM LPAR3 PLCB1 DIAPH2 MTM1 UST PLXND1 CLTCL1 PRKCA ZNF875 LIAS RPS6KA2 TLE2 CELSR1 SPRED2 NPAS2 FOXN2 KANK1 EFNB1 FZD8 P2RX4 ABAT SP8 IQCG PLAC1 DOCK5 COBL SHROOM3 PRKD1 ADGRV1 SNX10 LEPR BRSK1 IL17D FAXDC2 INPP5F EPHA6 NLGN1 RIN2 OPHN1 FLI1 OSBP2 GRIN2D ACTN2 SALL4 EYA2 BMPR1B RNF43 HUNK ASXL3 CHST8 MEGF11 SVIL WNT5B NEDD4 MTSS1 CAMK1D ETV6 EDNRA ADAMTS9 PPFIA2 EDA RBM47 MYO3A MAGI2 KALRN WNT3 ESRRB LDLRAD4 ADAM19 RILPL2 KIF26B RNF165 HIVEP3 NHS AGAP2 CHRM3 ZP3 NFASC RFX3 GJC2 DCLK1 AQP11 TNC AKAP6 CDH18 ERBB4 FRY APLF POU2F2 SNTG2 IGSF10 TMEFF2 SCIN PRICKLE2 PDE2A NEDD9 UNC5A NTRK2 CTNND2 PROC WDR72 MEI1 SULT2B1 NEURL1 EYS ADAMTS6 CLDN3 UPK1B ATP8A2 CCDC36 NOS1 SP100 PTPN22 ZNF141 LEFTY1 CHODL DTX1 LRRN3 C6ORF58 GPR68 HRH2 IL7 KIAA0319 ALK DOK6 MOBP TAFA1 NTM SLC2A14 TOX TTPA WNT9B*

			BCAS1 SLC4A10 DNAH5 PCDH15 NME5 ADAM12 PTPRQ TRPM1 EVX2 PTPRR MALL HCN1 TENM2 OPRM1 BHLHE22 KCNC2 CYP7B1 SYCP1 RHEX KCNK2 VIT CNTN4 RXFP1 ZFP42 SEMA5A SH3TC2 TFAP2D LINGO2 HSD17B3 KRTAP5-11 TDRD9 CA10 APOH DCC DSCAM NFAM1 IL1RAPL1
GO:0019538	protein metabolic process	7.101336E-03	*HFM1 ALG9 AP001781.2 GCNT1 PID1 DPP10 EYA4 LYN RELN LRRTM3 EIF3E ADAMTS8 PPEF2 GALNT1 BMP8B USP53 MEX3C DMD RGS3 NCL CAPNS1 GIGYF2 PAK1 LRP8 ZNRF3 RNF150 TEX14 CD80 RAB11A APP MAPK1 NUP98 BCR OTUD5 OAZ2 NAA25 LGMN KDM2A PIM3 PUS7 RAP1A ABR BMP7 PPP3CA MAP3K3 ATXN7 AKAP13 KMT2B USP13 PHKB TIAM1 PTPRG HERC4 GLI3 PHLPP1 CEBPA HDAC4 SASH1 FBXL17 EPHA3 IMMP2L CD74 CPAMD8 FLT3 FEZF2 PRMT8 HSP90AA1 RPL35A SF3B3 ILF3 SNRNP70 DNMT1 HNRNPR PPA1 UBE2M CCNB1 ARIH1 PSMB2 SUMO1 UBE2D2 IPO7 CDC123 IPO5 MRPL12 SSU72 ELAVL1 CDC27 NPEPPS GGA2 EIF3J HIF1A CAMTA1 DARS2 PUM1 YY1 FXR1 DDX6 GLRX5 KTN1 UBE2V2 CUL1 ADD1 MDM2 PTPN11 MRPL15 NPLOC4 BAZ1A UHRF1 PDCD4 AUTS2 SPAG9 SARNP RNF220 ROBO1 NAA15 NSD1 PPP1R12A FAF1 NUP133 SEPHS1 GIPC1 POLDIP3 TSG101 B4GALT2 DPH1 WNK2 CCDC88A CDC23 PREPL PPP1R7 BRCA1 CNOT6 RNF130 NFU1 HDAC8 SETD1A TNRC6A PIAS1 EHMT1 GALNT2 FGFR1 LRRC41 RC3H2 NXN PPME1 AIDA SENP6 SELENOS RHBDD3 STK11 EIF2B4 UBE4B NAA60 CHEK1 RNF4 ARHGEF2 UNKL TSPYL2 TELO2 POR FBXO28 PTPN1 GCLC IDE MTA3 KCTD10 AP3B1 MAP2K7 SRPK2 BLM RBX1 RPAP2 EIF2AK2 OGDH PRMT3 PDPK1 CCNE1 NEK4 TCF7L2 PIGU ITCH MYSM1 DMAC2 ROCK2 CSNK1G3 CASK STAT3 KDM2B OTUD4 USP4 SIRT2 CIT FOXF2 UBR3 TNRC6B MAP3K2 BRCA2 HS6ST2 SLC39A10 VPS37B CCNY KANSL2 RANBP9 RNF149 RNF121 RPTOR DLC1 USP32 RNF111 KLHL22 DTNBP1 PPP2R3C PEX14 TRAK1 GFPT1 BRAF PIGX HECTD4 BRAP TULP4 ASB3 PTPN9 BCL11A SIRT1 CARD8 TNFRSF10B PKN3 KMT5B WWP1 ALKBH1 DSE PTPN4 HS6ST1 KDM4B RAPGEF1 XPNPEP3 MAP2K4 MAP2K5 CASP7 TRIM14 FUT8 EEFSEC BTRC DCAF10 IFT172 HERC1 USP18 CHFR RHBDF2 PHKA1 OTUD7B CLEC16A PIGN KAT14 RCAN1 CDK17 CNOT6L OXR1 QRSL1 RNF157 ERRFI1 XXYLT1 SRBD1 STK39 ACSL1 FBXW4 TET1 MOB1B PPARD ABHD17C RPS6KC1 MTRF1 LARGE1 RHOT1 NEDD4L TNRC6C FAM161A LPAR3 SNTA1 TBCK PALD1 PLCB1 JADE3 MTM1 UST DSTYK PRKCA EEF1AKMT2 LIAS NEDD8-MDP1 RPS6KA2 SPRED2 FZD8 KANSL1L FAM220A PROS1 PRKD1 ADGRV1 USP44 LEPR TNFAIP8 PLAUR BRSK1 IL17D FAXDC2 INPP5F EPHA6 WDFY2 FBXO4 OPHN1 PPM1N SHC2 EYA2 BMPR1B RNF43 HUNK CHST8 MDP1 CPEB2 NEDD4 CAMK1D PPP1R3B ADAMTS9 AC092647.5 RAB3B MYO3A INKA2 MAGI2 KALRN SEPSECS LDLRAD4 ADAM19 ACER2 RNF165 LAMP3 AGAP2 CHRM3 OTUD7A KHDC1 GJC2 DCLK1 AQP11 USP43 TNC ERBB4 FRY TRHDE PLCL1 SCG5 AL133500.1 NLRC4 GALNT14 NEDD9 NTRK2 TNXB FBXL13 PROC AC068896.1 TRAF1 NEURL1 ADAMTS6 CLDN3 HS3ST5 WFDC3 NOS1 AC004687.2 PTPN22 LEFTY1 DTX1 SLCO3A1 PPP1R36 IL7 ALK SLC11A1 ADGB COL28A1 NDST3 BANK1 HS3ST4 WNT9B LAPTM5 LRRC2 AC013717.1 ADAM12 DIO2 ASMT LYZ PTPRQ PTPRR AC058822.1 PADI4 SPINK4 MGAT4C APOH SERPINB11 ARRDC5 NLRP2 PTPRT*
GO:0030010	establishment of cell polarity	7.109256E-03	*PAK1 CDK5RAP2 SNX27 AMOTL1 FOXF1 WDPCP HSP90AA1 GBF1 STK11 NDE1 ARHGEF2 ROCK2 CENPA SHTN1 MYO9A CLASP1 CYTH1 FBF1 UST KANK1 BRSK1 OPHN1 KIF26B DOCK8*
GO:0051128	regulation of cellular component organization	7.450077E-03	*PDE3A PID1 LYN RELN LRRTM3 HDGFL3 MAP1S DMD PLCG2 PAK1 LRP8 RUNX1 TEX14 GSG1L RAB11A APP MAPK1 VCL RAP1A NOTCH2 BMP7 PPP3CA CDK5RAP2 ATXN7 AKAP13 KMT2B PLS1 TIAM1 PTPRG HDAC4 EPHA3 WDPCP ROBO2 CACNA1A HSP90AA1 DNMT1 CCNB1 SUMO1 GDI1 CDC27 YWHAH FOXM1 HIF1A YY1 FXR1 EXOSC10 SEPT9 ADD1 MDM2 PTPN11 AUTS2 SPAG9 ROBO1 NSD1 CDCA5 FAF1 TFDP1 CLSTN1 TSG101 CCDC88A CDC23 BRCA1 TERF2 CNOT6 HDAC8 SETD1A DENND5A*

			GOLGA4 TFDP2 FGFR1 RHOQ AIDA SENP6 TBCD STK11 CHEK1 RNF4 PDE4DIP ARHGEF2 TSPYL2 PTPN1 GCLC MAP2K7 RBX1 PDLIM5 EIF2AK2 WAPL ATF7IP ITCH PLEKHM2 PSMG2 STMP1 ROCK2 KDM2B SIRT2 RPTOR SHTN1 MYO10 DLC1 KLHL22 SMAP1 ZMYND8 TRAK1 CHMP4A BRAF PTPN9 BCL11A SIRT1 MYO9A ITSN2 KDM4B CLASP1 RAPGEF1 RREB1 MAP2K5 PIP4K2B SMG6 CROCC FCHSD2 CLEC16A ATL3 EPB41L5 CNOT6L RNF157 SPIRE2 CTNNBIP1 TET1 PPARD DOCK11 ABHD17C MTRF1 RHOT1 NEDD4L LPAR3 PLCB1 MTM1 UST PLXND1 PLSCR3 CEP76 TBC1D5 RPS6KA2 CELSR1 KANK1 COBL PRKD1 USP44 PLAUR BRSK1 INPP5F FBXO4 NLGN1 OPHN1 BTBD9 ACTN2 SVIL NEDD4 STON1 CAMK1D CDA PPFIA2 MAGI2 WNT3 LDLRAD4 AKAP6 ANK1 KANK3 TMEFF2 SCIN CORO2B PDE2A NTRK2 NEURL1 CLDN3 ATP8A2 NOS1 CHODL LRRN3 KIAA0319 ALK DNAJC15 TOX TENM2 SEMA5A LINGO2 DCC PTPRT DSCAM IL1RAPL1
GO:1901564	organonitrogen compound metabolic process	8.485680E-03	HFM1 ALG9 AP001781.2 GCNT1 PID1 DLG2 DPP10 EYA4 LYN RELN LRRTM3 EIF3E ADAMTS8 PPEF2 GALNT1 BMP8B USP53 MEX3C DMD RGS3 NCL CAPNS1 GIGYF2 PAK1 LRP8 ZNRF3 RNF150 PDE1B TEX14 CD80 RAB11A APP FOXC1 MAPK1 NUP98 BCR OTUD5 NAMPT OAZ2 NAA25 LGMN KDM2A PIM3 PUS7 SLC25A13 RAP1A ABR BMP7 OGG1 PPP3CA MAP3K3 ATXN7 ABCC1 AKAP13 KMT2B USP13 PHKB TIAM1 PTPRG HERC4 GLI3 PHLPP1 CEBPA HDAC4 SASH1 FBXL17 EPHA3 IMMP2L STS GPC5 CD74 CPAMD8 CACNA1A SLC52A1 FLT3 FEZF2 PRMT8 HSP90AA1 RPL35A SF3B3 ILF3 SNRNP70 ATP5MC2 DNMT1 HNRNPR PPA1 UBE2M CCNB1 ARIH1 PSMB2 SUMO1 UBE2D2 IPO7 CDC123 CBS IPO5 MRPL12 SSU72 ELAVL1 CDC27 NPEPPS GGA2 EIF3J HIF1A CAMTA1 NCOR1 DARS2 PUM1 YY1 FXR1 DDX6 GLRX5 KTN1 UBE2V2 CUL1 ADD1 MDM2 PTPN11 MRPL15 NPLOC4 BAZ1A UHRF1 PDCD4 AUTS2 SPAG9 SARNP RNF220 ROBO1 NAA15 ACADM NSD1 PDXK PPP1R12A FAF1 NUP133 SEPHS1 GIPC1 POLDIP3 GSR TSG101 B4GALT2 ALDH7A1 DPH1 WNK2 CCDC88A CDC23 PREPL PPP1R7 BRCA1 CNOT6 RNF130 NFU1 HDAC8 HK1 SETD1A ESYT1 TNRC6A PIAS1 EHMT1 GALNT2 AKR1A1 FGFR1 LRRC41 RC3H2 NXN PPME1 RHOQ AIDA SENP6 SELENOS RHBDD3 STK11 EIF2B4 UBE4B NAA60 CHEK1 RNF4 ARHGEF2 UNKL TSPYL2 TELO2 POR FBXO28 PCCB PTPN1 GCLC IDE MTA3 KCTD10 AP3B1 MAP2K7 SRPK2 BLM RBX1 RPAP2 EIF2AK2 OGDH PRMT3 PDPK1 CCNE1 NEK4 TCF7L2 PIGU ITCH MYSM1 DMAC2 SUCLG2 ROCK2 CSNK1G3 FAR2 ADK CASK STAT3 KDM2B HIBADH OTUD4 USP4 SIRT2 CIT FOXF2 UBR3 TNRC6B MAP3K2 BRCA2 HS6ST2 SLC39A10 VPS37B CCNY KANSL2 RANBP9 RNF149 PAPSS1 RNF121 RPTOR DLC1 USP32 RNF111 KLHL22 DTNBP1 PPP2R3C PEX14 TRAK1 GFPT1 BRAF PIGX HECTD4 BRAP TULP4 ASB3 PTPN9 BCL11A SIRT1 CARD8 TNFRSF10B PKN3 KMT5B WWP1 ALKBH1 DSE PTPN4 HS6ST1 KDM4B RAPGEF1 XPNPEP3 MAP2K4 MAP2K5 PDE7A CASP7 TRIM14 FUT8 EEFSEC BTRC DCAF10 IFT172 HERC1 SLC16A10 USP18 CHFR RHBDF2 PHKA1 OTUD7B CLEC16A PIGN PC KAT14 RCAN1 CDK17 CNOT6L TYW1 OXR1 QRSL1 SLC44A1 RNF157 ERRFI1 XXYLT1 SRBD1 STK39 ACSL1 FBXW4 TET1 MOB1B PPARD DMAC2L ABHD17C RPS6KC1 MTRF1 LARGE1 RHOT1 NEDD4L TNRC6C DCK FAM161A CREM LPAR3 SNTA1 TBCK PALD1 PLCB1 JADE3 MTM1 CERS4 UST DSTYK DGLUCY PRKCA EEF1AKMT2 LIAS NEDD8-MDP1 RPS6KA2 VPS9D1 SPRED2 PANK1 FZD8 ABAT KANSL1L FAM220A PROS1 PRKD1 ADGRV1 USP44 LEPR TNFAIP8 PLAUR BRSK1 IL17D FAXDC2 INPP5F EPHA6 WDFY2 FBXO4 OPHN1 BTBD9 PPM1N SHC2 EYA2 BMPR1B RNF43 HUNK CHST8 MDP1 CPEB2 NEDD4 CAMK1D PPP1R3B CDA ADAMTS9 AC092647.5 RAB3B MYO3A INKA2 MAGI2 KALRN SEPSECS ESRRB LDLRAD4 ADAM19 ACER2 RNF165 LAMP3 ZBTB20 NME9 AGAP2 CHRM3 SLC7A7 OTUD7A KHDC1 GJC2 DCLK1 AQP11 USP43 TNC ERBB4 FRY TRHDE PLCL1 SCG5 AL133500.1 CRYM NLRC4 GALNT14 PDE2A NEDD9 NTRK2 DMGDH TNXB FBXL13

			PROC AC068896.1 TRAF1 SULT2B1 NEURL1 NOXRED1 ADAMTS6 CLDN3 HS3ST5 WFDC3 NOS1 AC004687.2 PTPN22 LEFTY1 DTX1 SLCO3A1 PPP1R36 IL7 ALK SLC11A1 ADGB COL28A1 HPD NDST3 BANK1 HS3ST4 WNT9B LAPTM5 LRRC2 AC013717.1 NME5 ADAM12 DIO2 ASMT LYZ PTPRQ PTPRR AC058822.1 PADI4 AL117348.2 BHMT2 SPINK4 MGAT4C DAO APOH SERPINB11 ARRDC5 NLRP2 PTPRT
GO:0044093	positive regulation of molecular function	8.616618E-03	*RYR2 LYN RELN EIF3E PKP4 DMD PLCG2 RGS3 PAK1 LRP8 RASGRF1 APP EPB41 FOXC1 MAPK1 BCR LGMN ARHGAP21 RAP1A ABR PPP3CA MAP3K3 AKAP13 TIAM1 HDAC4 SASH1 EPHA3 SGSM1 CD74 FLT3 Z82190.2 HSP90AA1 CCNB1 SUMO1 GDI1 HIF1A GTF2F1 ADD1 PTPN11 UHRF1 SPAG9 ROBO1 CDCA5 PPP1R12A TFDP1 WNK2 CCDC88A TERF2 HDAC8 FGFR1 GAPVD1 MBD2 TBCD STK11 ARHGEF2 TELO2 POR SOS1 PTPN1 IDE MAP2K7 EIF2AK2 WAPL PDPK1 TCF7L2 ARFGAP3 TP53BP1 ROCK2 STAT3 SIRT2 MAP3K2 RAP1GDS1 SLC39A10 CCNY WRAP53 SH3PXD2A RPTOR ARHGAP8 RABGAP1L DLC1 SMAP1 PPP2R3C BRAF SIRT1 CARD8 TNFRSF10B MYO9A RAPGEF1 MAP2K4 MAP2K5 TRIM14 BTRC TBC1D20 EPB41L5 PPP1R12B DEPDC5 ERRFI1 ARHGEF26 STK39 ACSL1 RALGAPA2 MOB1B DOCK11 LPAR3 TBCK PLCB1 TBC1D22A PLXND1 DSTYK TBC1D5 VPS9D1 FZD8 DOCK5 PRKD1 PLAUR EPHA6 RIN2 OPHN1 ACTN2 SHC2 CAMK1D EDNRA EDA MAGI2 ACER2 AGAP2 KHDC1 AKAP6 DOCK8 ERBB4 PLCL1 NLRC4 NEDD9 NTRK2 TRAF1 NEURL1 CLDN3 NOS1 SP100 SLCO3A1 ALK RGPD3 SLC11A1 DNAJC15 WNT9B KCNC2 APOH NLRP2 CACNG2 NFAM1*
GO:0032880	regulation of protein localization	9.613589E-03	*PID1 DPP10 PAK1 SLC8B1 GSG1L RAB11A CTNNA1 EPB41 MAPK1 OAZ2 VCL PIM3 RAP1A PPP3CA STX8 PLS1 TIAM1 GLI3 EPHA3 WDPCP GPC5 CACNA1A HSP90AA1 SUMO1 IPO5 GDI1 YWHAH NPEPPS HIF1A MDM2 PTPN11 WLS CDCA5 PCM1 TFDP1 TSG101 CCDC88A HDAC8 GTSE1 TFDP2 GAPVD1 RHOQ GBF1 RHBDD3 STK11 PTPN1 WAPL PDPK1 CCNE1 TCF7L2 PLEKHM2 ROCK2 RAB11FIP3 WRAP53 RABGAP1L ZMYND8 PTPN9 TRIM14 CROCC RHBDF2 OTUD7B EPB41L5 XPO4 PPARD ABHD17C NEDD4L PRKCA ABAT PRKD1 FBXO4 NLGN1 OPHN1 ACTN2 SORBS1 RFX3 DCLK1 ERBB4 CORO2B SP100 PTPN22 GPR68 CD247 OPRM1 KCNC2 SYCP1 SH3TC2 CACNG2*
GO:0010604	positive regulation of macromolecule metabolic process	9.640596E-03	*PID1 PMF1 EYA4 LYN RELN LRRTM3 EIF3E BMP8B PLCG2 NCL GIGYF2 RERE PAK1 LRP8 POU3F3 EPB41L4B NKX2-1 RUNX1 CD80 APP FOXC1 MAPK1 NUP98 NAMPT OAZ2 ACTN1 LGMN HOXB3 RAP1A BMP7 PPP3CA CDK5RAP2 ZNF131 MAP3K3 ATXN7 AKAP13 KMT2B USP13 TIAM1 GLI3 ZFHX3 CEBPA HDAC4 SASH1 AHR FOXF1 ESRRG NKX2-3 CD74 FLT3 FEZF2 HHLA2 HSP90AA1 ILF3 SNRNP70 DNMT1 HNRNPR CCNB1 ID2 ARIH1 SUMO1 TAF1D CDC123 MRPL12 ELAVL1 YWHAH BPTF FOXM1 HIF1A UBP1 CAMTA1 NCOR1 PUM1 YY1 FXR1 GTF2F1 MDM2 PTPN11 DNAJC2 UHRF1 AUTS2 SPAG9 ROBO1 NAA15 NSD1 PPP1R12A FAF1 CHD8 POLDIP3 TFDP1 TSG101 CCDC88A PPP1R7 BRCA1 TERF2 HDAC8 HK1 TNRC6A MLXIP SSBP4 PIAS1 TFDP2 FGFR1 DLX2 ARID1B RHOQ RHBDD3 STK11 CHEK1 ICE2 NKX2-5 RNF4 ARHGEF2 TELO2 ALDH1A2 PTPN1 GCLC IDE MTA3 AP3B1 MAP2K7 SRPK2 BLM RBX1 EIF2AK2 ATF7IP PDPK1 NEK4 TCF7L2 TFCP2 ITCH MYSM1 TP53BP1 STMP1 ROCK2 MEF2A CASK STAT3 POLR3G OTUD4 SIRT2 FOXF2 TNRC6B MAP3K2 BRCA2 SLC39A10 CCNY NFAT5 RANBP9 EPS15 WRAP53 ZXDC RPTOR DLC1 RNF111 TCF12 DTNBP1 PPP2R3C BRAF BCL11A SIRT1 CARD8 TNFRSF10B KMT5B WWP1 ZNF143 RAPGEF1 WWOX MAP2K4 RREB1 MAP2K5 BTRC CHFR CNOT6L FRMD8 SPIRE2 IFT74 STK39 ACSL1 TET1 MED12L NFIX PPARD RFX2 NEDD4L TNRC6C FAM161A CREM LPAR3 PLCB1 HIVEP1 PLXND1 GMEB2 TBC1D5 PRDM10 RPS6KA2 NPAS2 FZD8 PRKD1 LEPR PLAUR IL17D FAXDC2 INPP5F WDFY2 FBXO4 FLI1 ACTN2 SHC2 SALL4 EYA2 BMPR1B ASXL3 SORBS1 NEDD4 ETV6 EDA MAGI2 WNT3 ESRRB ADAM19 ACER2 HIVEP3 LAMP3 ZBTB20 AGAP2 ZP3 KHDC1 RFX3 GJC2 GLIS3 TNC ERBB4*

			APLF POU2F2 C1QTNF4 NLRC4 PDE2A NEDD9 NTRK2 NEURL1 CLDN3 NOS1 SP100 AC019117.3 PTPN22 LEFTY1 DTX1 SLCO3A1 IL7 ALK ZNF43 SLC11A1 BANK1 TOX LAPTM5 TFAP2D NLRP2 NFAM1
GO:0032502	developmental process	1.132650E-02	*UNC45B PDE3A RYR2 AAMDC GCNT1 PID1 UTRN EYA4 LYN RELN CCDC85C LRRTM3 BMP8B HDGFL3 MAMLD1 PKP4 MYO1D MAP1S MEX3C DMD PLCG2 NCL CAPNS1 GIGYF2 RERE PAK1 LRP8 NOTCH2NLA KAZN ZNRF3 POU3F3 NKX2-1 RUNX1 BSN PDE1B RASGRF1 CD80 DKK2 RAB11A APP CTNNA1 ZFR EPB41 FOXC1 MAPK1 BCR NAMPT ACTN1 VCL LGMN PUS7 HOXB3 RAP1A ABR NOTCH2 BMP7 OGG1 PPP3CA CDK5RAP2 MAP3K3 AMOTL1 AKAP13 KMT2B USP13 PLS1 TIAM1 GTF2IRD1 PTPRG HERC4 GLI3 PHLPP1 FZD6 ZFHX3 CEBPA FAM172A HDAC4 SYNE1 SASH1 AHR NEBL FBXL17 FOXF1 EPHA3 IMMP2L WDPCP ROBO2 ZSWIM4 STS NKX2-3 SLC24A4 CD74 CPAMD8 RYR1 NUP210L CACNA1A OPCML FLT3 LAMA2 FEZF2 HSP90AA1 DNMT1 SMC1A ACIN1 GNB1 CCNB1 ID2 PSMB2 SUMO1 GDI1 ELAVL1 YWHAH SND1 BPTF FOXM1 FAM50A HIF1A UBP1 PUM1 YY1 FXR1 DDX6 GLRX5 CUL1 ADD1 MDM2 PTPN11 WLS HELLS PDCD4 AUTS2 SPAG9 ROBO1 RBFOX2 NAA15 ACADM NUP133 PCM1 CHD8 TFDP1 ASF1B CLSTN1 TSG101 B4GALT2 CCDC88A AP1G1 BRCA1 SEPT6 TERF2 ACO2 SETD1A DENND5A SELENOM TNRC6A PIAS1 EHMT1 GOLGA4 TFDP2 FGFR1 DLX2 GOLGA3 ARID1B RC3H2 NXN RHOQ AIDA PDS5B RHBDD3 MBD2 TBCD STK11 EIF2B4 UBE4B CHEK1 NKX2-5 NDE1 ARHGEF2 PHF3 POR SOS1 ALDH1A2 GCLC IDE AP3B1 SRPK2 PDLIM5 EIF2AK2 OGDH ESS2 PDPK1 NEK4 TCF7L2 ITCH MYSM1 SCMH1 TP53BP1 ROCK2 MEF2A STAT3 EPN2 KDM2B MYEF2 SIRT2 CIT FOXF2 UBR3 TNRC6B RAI14 BRCA2 RBL2 RANBP9 PAPSS1 SH3PXD2A GREB1L SHTN1 MYO10 DLC1 OFD1 RNF111 TAF8 SMAP1 TCF12 DTNBP1 PPP2R3C ZMYND8 TRAK1 BRAF PCDH9 IFT81 KNL1 STRN PTPN9 BCL11A SIRT1 KMT5B MYO9A WWP1 ALKBH1 HS6ST1 ITSN2 CLASP1 RAPGEF1 WWOX MAP2K4 ANKS1A RREB1 MAP2K5 CASP7 FUT8 BTRC IFT172 HERC1 FOXN3 TBC1D20 TPRN OTUD7B RCAN1 EPB41L5 TMEM131L RNF157 ERRFI1 IFT74 ARHGEF26 CTNNBIP1 FBXW4 TET1 PPARD DOCK11 HES6 LARGE1 RFX2 NEDD4L TNRC6C CREM LPAR3 PLCB1 DIAPH2 MTM1 UST PLXND1 CLTCL1 PRKCA ZNF875 LIAS RPS6KA2 TLE2 CELSR1 SPRED2 NPAS2 FOXN2 KANK1 EFNB1 FZD8 P2RX4 ABAT SP8 IQCG PLAC1 DOCK5 COBL SHROOM3 LRRC8C PRKD1 ADGRV1 SNX10 LEPR BRSK1 IL17D FAXDC2 INPP5F EPHA6 CCDC3 WDFY2 FBXO4 NLGN1 RIN2 OPHN1 FLI1 OSBP2 GRIN2D ACTN2 SALL4 EYA2 BMPR1B RNF43 HUNK ASXL3 CHST8 MEGF11 SVIL WNT5B NEDD4 MTSS1 CAMK1D ETV6 EDNRA ADAMTS9 PPFIA2 EDA RBM47 MYO3A MAGI2 KALRN WNT3 ESRRB LDLRAD4 ADAM19 RILPL2 KIF26B C1QL4 RNF165 HIVEP3 NHS AGAP2 CHRM3 ZP3 NFASC RFX3 GJC2 DCLK1 AQP11 TNC AKAP6 CDH18 CNTD1 ERBB4 FRY APLF POU2F2 SNTG2 IGSF10 TMEFF2 SCIN PRICKLE2 PDE2A NEDD9 UNC5A NTRK2 CTNND2 PROC WDR72 MEI1 SULT2B1 NEURL1 EYS ADAMTS6 CLDN3 UPK1B ATP8A2 CCDC36 NOS1 SP100 PTPN22 ZNF141 LEFTY1 CHODL DTX1 LRRN3 C6ORF58 GPR68 HRH2 IL7 KIAA0319 ALK DOK6 MOBP TAFA1 NTM SLC2A14 TOX TTPA WNT9B BCAS1 SLC4A10 DNAH5 PCDH15 NME5 ADAM12 DIO2 PTPRQ TRPM1 PCDH11Y EVX2 PTPRR MALL HCN1 TENM2 OPRM1 BHLHE22 KCNC2 UNC13C CYP7B1 PADI4 SYCP1 RHEX KCNK2 VIT CNTN4 RXFP1 ZFP42 SEMA5A SH3TC2 TFAP2D LINGO2 HSD17B3 KRTAP5-11 TDRD9 CA10 APOH DCC DSCAM NFAM1 IL1RAPL1*
GO:0051173	positive regulation of nitrogen compound metabolic process	1.148483E-02	*PID1 PMF1 EYA4 LYN RELN LRRTM3 EIF3E BMP8B NCL GIGYF2 RERE PAK1 LRP8 POU3F3 NKX2-1 RUNX1 CD80 APP FOXC1 MAPK1 NUP98 NAMPT OAZ2 ACTN1 LGMN HOXB3 RAP1A BMP7 PPP3CA CDK5RAP2 ZNF131 MAP3K3 ATXN7 AKAP13 KMT2B USP13 TIAM1 GLI3 ZFHX3 CEBPA HDAC4 SASH1 AHR FOXF1 ESRRG NKX2-3 CD74 FLT3 FEZF2 HSP90AA1 ILF3 SNRNP70 DNMT1 HNRNPR CCNB1 ID2 ARIH1 SUMO1 CDC123*

			MRPL12 ELAVL1 YWHAH BPTF FOXM1 HIF1A UBP1 CAMTA1 PUM1 YY1 FXR1 GTF2F1 MDM2 PTPN11 DNAJC2 UHRF1 AUTS2 SPAG9 ROBO1 NAA15 NSD1 PPP1R12A FAF1 CHD8 POLDIP3 TFDP1 TSG101 CCDC88A PPP1R7 BRCA1 HDAC8 TNRC6A MLXIP SSBP4 PIAS1 TFDP2 FGFR1 DLX2 ARID1B RHOQ RHBDD3 STK11 CHEK1 ICE2 NKX2-5 RNF4 ARHGEF2 TELO2 PTPN1 GCLC IDE MTA3 AP3B1 MAP2K7 BLM RBX1 EIF2AK2 ATF7IP PDPK1 NEK4 TCF7L2 TFCP2 ITCH MYSM1 TP53BP1 ROCK2 MEF2A CASK STAT3 OTUD4 SIRT2 FOXF2 TNRC6B MAP3K2 BRCA2 SLC39A10 CCNY NFAT5 RANBP9 WRAP53 ZXDC RPTOR DLC1 RNF111 TCF12 PPP2R3C BRAF BCL11A SIRT1 CARD8 TNFRSF10B KMT5B WWP1 ZNF143 RAPGEF1 WWOX MAP2K4 RREB1 MAP2K5 BTRC CHFR CNOT6L SPIRE2 IFT74 STK39 ACSL1 TET1 MED12L NFIX PPARD RFX2 NEDD4L TNRC6C FAM161A CREM LPAR3 PLCB1 HIVEP1 PLXND1 GMEB2 PRDM10 NPAS2 FZD8 P2RX4 ABAT PRKD1 LEPR PLAUR IL17D FAXDC2 WDFY2 FBXO4 FLI1 ACTN2 SHC2 SALL4 EYA2 BMPR1B ASXL3 NEDD4 ETV6 MAGI2 ESRRB ACER2 HIVEP3 ZBTB20 AGAP2 ZP3 KHDC1 RFX3 GLIS3 ERBB4 APLF POU2F2 NLRC4 NEDD9 NTRK2 NEURL1 CLDN3 NOS1 SP100 AC019117.3 PTPN22 LEFTY1 DTX1 SLCO3A1 ALK ZNF43 SLC11A1 BANK1 TOX LAPTM5 OPRM1 TFAP2D NLRP2
GO:0009893	positive regulation of metabolic process	1.273147E-02	*PID1 PMF1 EYA4 LYN RELN LRRTM3 EIF3E BMP8B PLCG2 NCL GIGYF2 RERE PAK1 LRP8 POU3F3 EPB41L4B NKX2-1 RUNX1 CD80 APP FOXC1 MAPK1 NUP98 NAMPT OAZ2 ACTN1 LGMN HOXB3 RAP1A BMP7 PPP3CA CDK5RAP2 ZNF131 MAP3K3 ATXN7 AKAP13 KMT2B USP13 TIAM1 GLI3 ZFHX3 CEBPA HDAC4 SASH1 AHR FOXF1 EPHA3 ESRRG NKX2-3 CD74 FLT3 FEZF2 HHLA2 HSP90AA1 ILF3 SNRNP70 DNMT1 HNRNPR CCNB1 ID2 ARIH1 SUMO1 TAF1D CDC123 MRPL12 ELAVL1 YWHAH BPTF FOXM1 HIF1A UBP1 CAMTA1 NCOR1 PUM1 YY1 FXR1 GTF2F1 MDM2 PTPN11 DNAJC2 UHRF1 AUTS2 SPAG9 ROBO1 NAA15 NSD1 PPP1R12A FAF1 GIPC1 CHD8 POLDIP3 TFDP1 TSG101 CCDC88A PPP1R7 BRCA1 TERF2 HDAC8 HK1 TNRC6A MLXIP SSBP4 SCP2 PIAS1 EHMT1 TFDP2 FGFR1 DLX2 ARID1B RHOQ RHBDD3 STK11 CHEK1 ICE2 NKX2-5 RNF4 ARHGEF2 TELO2 POR ALDH1A2 PTPN1 GCLC IDE MTA3 AP3B1 MAP2K7 SRPK2 BLM RBX1 EIF2AK2 ATF7IP PDPK1 NEK4 TCF7L2 TFCP2 ITCH MYSM1 TP53BP1 STMP1 ROCK2 MEF2A CASK STAT3 POLR3G OTUD4 SIRT2 FOXF2 TNRC6B MAP3K2 BRCA2 SLC39A10 CCNY NFAT5 RANBP9 EPS15 WRAP53 ZXDC RPTOR DLC1 RNF111 TCF12 DTNBP1 PPP2R3C BRAF BCL11A SIRT1 CARD8 TNFRSF10B KMT5B WWP1 ZNF143 RAPGEF1 WWOX MAP2K4 RREB1 MAP2K5 TRIM14 PIP4K2B BTRC CHFR CLEC16A CNOT6L FRMD8 SPIRE2 IFT74 STK39 ACSL1 TET1 MED12L NFIX MOB1B PPARD DHRSX RFX2 NEDD4L TNRC6C FAM161A CREM LPAR3 PLCB1 HIVEP1 PLXND1 DSTYK GMEB2 TBC1D5 PRDM10 RPS6KA2 NPAS2 FZD8 P2RX4 ABAT PRKD1 LEPR PLAUR IL17D FAXDC2 INPP5F EPHA6 CCDC3 WDFY2 FBXO4 FLI1 ACTN2 SHC2 SALL4 EYA2 BMPR1B ASXL3 SORBS1 NEDD4 CAMK1D ETV6 EDA MAGI2 WNT3 ESRRB ADAM19 ACER2 HIVEP3 LAMP3 ZBTB20 AGAP2 ZP3 KHDC1 RFX3 GJC2 GLIS3 TNC ERBB4 APLF POU2F2 C1QTNF4 NLRC4 PDE2A NEDD9 NTRK2 NEURL1 CLDN3 NOS1 SP100 AC019117.3 PTPN22 LEFTY1 DTX1 SLCO3A1 IL7 ALK ZNF43 SLC11A1 BANK1 TOX LAPTM5 DIO2 OPRM1 TFAP2D NLRP2 DSCAM NFAM1*
GO:0010720	positive regulation of cell development	1.385931E-02	*PDE3A LYN RELN PAK1 LRP8 RAB11A TIAM1 GLI3 ROBO2 ID2 GDI1 HIF1A ADD1 ROBO1 GOLGA4 STK11 SIRT2 SHTN1 TRAK1 BRAF BCL11A RREB1 LPAR3 PLXND1 DOCK5 WNT3 RFX3 GJC2 NEDD9 NTRK2 PROC NEURL1 CHODL GPR68 OPRM1 SEMA5A DSCAM IL1RAPL1*
GO:0044267	cellular protein metabolic process	1.424989E-02	*HFM1 ALG9 AP001781.2 GCNT1 PID1 EYA4 LYN RELN EIF3E PPEF2 GALNT1 BMP8B USP53 MEX3C DMD RGS3 NCL GIGYF2 PAK1 LRP8 ZNRF3 RNF150 TEX14 CD80 RAB11A APP MAPK1 NUP98 BCR OTUD5 NAA25 LGMN KDM2A PIM3 PUS7 RAP1A ABR BMP7 PPP3CA MAP3K3 ATXN7 AKAP13 KMT2B USP13 PHKB TIAM1 PTPRG HERC4 PHLPP1 CEBPA HDAC4*

			SASH1 FBXL17 EPHA3 IMMP2L CD74 CPAMD8 FLT3 FEZF2 PRMT8 HSP90AA1 RPL35A ILF3 DNMT1 HNRNPR PPA1 UBE2M CCNB1 ARIH1 PSMB2 SUMO1 UBE2D2 IPO7 CDC123 IPO5 MRPL12 SSU72 ELAVL1 CDC27 NPEPPS GGA2 EIF3J HIF1A CAMTA1 DARS2 PUM1 YY1 FXR1 DDX6 KTN1 UBE2V2 CUL1 MDM2 PTPN11 MRPL15 NPLOC4 BAZ1A UHRF1 PDCD4 AUTS2 SPAG9 SARNP RNF220 ROBO1 NAA15 NSD1 PPP1R12A FAF1 NUP133 SEPHS1 GIPC1 POLDIP3 TSG101 B4GALT2 DPH1 WNK2 CCDC88A CDC23 PPP1R7 BRCA1 CNOT6 RNF130 HDAC8 SETD1A TNRC6A PIAS1 EHMT1 GALNT2 FGFR1 LRRC41 RC3H2 NXN PPME1 AIDA SENP6 SELENOS STK11 EIF2B4 UBE4B NAA60 CHEK1 RNF4 ARHGEF2 UNKL TSPYL2 TELO2 POR FBXO28 PTPN1 GCLC IDE MTA3 KCTD10 AP3B1 MAP2K7 SRPK2 BLM RBX1 RPAP2 EIF2AK2 OGDH PRMT3 PDPK1 CCNE1 NEK4 PIGU ITCH MYSM1 DMAC2 ROCK2 CSNK1G3 CASK STAT3 KDM2B OTUD4 USP4 SIRT2 CIT FOXF2 UBR3 TNRC6B MAP3K2 BRCA2 SLC39A10 VPS37B CCNY KANSL2 RNF149 RNF121 RPTOR DLC1 USP32 RNF111 KLHL22 DTNBP1 PPP2R3C PEX14 TRAK1 GFPT1 BRAF PIGX HECTD4 BRAP TULP4 ASB3 PTPN9 BCL11A SIRT1 CARD8 TNFRSF10B PKN3 KMT5B WWP1 ALKBH1 PTPN4 KDM4B RAPGEF1 MAP2K4 MAP2K5 TRIM14 FUT8 EEFSEC BTRC DCAF10 HERC1 USP18 CHFR PHKA1 OTUD7B CLEC16A PIGN KAT14 RCAN1 CDK17 CNOT6L OXR1 QRSL1 RNF157 ERRFI1 XXYLT1 SRBD1 STK39 ACSL1 FBXW4 TET1 MOB1B ABHD17C RPS6KC1 MTRF1 LARGE1 RHOT1 NEDD4L TNRC6C FAM161A LPAR3 SNTA1 TBCK PALD1 JADE3 MTM1 UST DSTYK PRKCA EEF1AKMT2 LIAS NEDD8-MDP1 RPS6KA2 SPRED2 FZD8 KANSL1L FAM220A PROS1 PRKD1 USP44 LEPR TNFAIP8 PLAUR BRSK1 FAXDC2 INPP5F EPHA6 WDFY2 FBXO4 OPHN1 PPM1N SHC2 EYA2 BMPR1B RNF43 HUNK MDP1 CPEB2 NEDD4 CAMK1D PPP1R3B AC092647.5 RAB3B MYO3A INKA2 MAGI2 KALRN SEPSECS LDLRAD4 ACER2 RNF165 LAMP3 AGAP2 CHRM3 OTUD7A KHDC1 GJC2 DCLK1 AQP11 USP43 TNC ERBB4 FRY PLCL1 AL133500.1 NLRC4 GALNT14 NEDD9 NTRK2 TNXB FBXL13 PROC AC068896.1 TRAF1 NEURL1 CLDN3 HS3ST5 WFDC3 NOS1 AC004687.2 PTPN22 LEFTY1 DTX1 SLCO3A1 PPP1R36 IL7 ALK SLC11A1 COL28A1 BANK1 WNT9B LAPTM5 LRRC2 AC013717.1 DIO2 ASMT LYZ PTPRQ PTPRR AC058822.1 PADI4 SPINK4 MGAT4C SERPINB11 ARRDC5 NLRP2 PTPRT
GO:0048522	positive regulation of cellular process	1.497234E-02	*PDE3A RYR2 AAMDC PID1 UTRN PMF1 DPP10 EYA4 LYN RELN LRRTM3 EIF3E BMP8B PKP4 DMD PLCG2 NCL CAPNS1 GIGYF2 RERE PAK1 LRP8 NOTCH2NLA POU3F3 EPB41L4B NKX2-1 RUNX1 RASGRF1 CD80 DKK2 RAB11A APP CTNNA1 EPB41 FOXC1 MAPK1 NUP98 BCR NAMPT OAZ2 ACTN1 LGMN HOXB3 RAP1A ABR NOTCH2 BMP7 PPP3CA CDK5RAP2 ZNF131 MAP3K3 AMOTL1 ATXN7 AKAP13 KMT2B USP13 PLS1 TIAM1 GLI3 ZFHX3 ARHGEF4 CEBPA HDAC4 SASH1 AHR FOXF1 EPHA3 ROBO2 GPC5 ESRRG NKX2-3 CD74 FLT3 LAMA2 FEZF2 HHLA2 HSP90AA1 ILF3 SNRNP70 DNMT1 HNRNPR ACIN1 UBE2M CCNB1 ID2 ARIH1 PSMB2 SUMO1 CDC123 IPO5 MRPL12 GDI1 ELAVL1 CDC27 YWHAH NPEPPS MTCH2 BPTF FOXM1 HIF1A UBP1 CAMTA1 PUM1 YY1 FXR1 DDX6 GTF2F1 SEPT9 ADD1 MDM2 PTPN11 WLS DNAJC2 UHRF1 PDCD4 AUTS2 SPAG9 RNF220 ROBO1 NAA15 NSD1 CDCA5 PPP1R12A FAF1 PCM1 WDR59 GIPC1 CHD8 POLDIP3 ELP6 TFDP1 CLSTN1 TSG101 WNK2 CCDC88A CDC23 AP1G1 PPP1R7 BRCA1 CNOT6 HDAC8 TNRC6A MLXIP TMED4 SSBP4 SCP2 PIAS1 GTSE1 GOLGA4 TFDP2 FGFR1 DLX2 ARID1B RC3H2 RHOQ MBD2 STK11 CHEK1 ICE2 NKX2-5 RNF4 PDE4DIP ARHGEF2 TELO2 POR SOS1 ALDH1A2 PTPN1 GCLC MTA3 AP3B1 MAP2K7 SRPK2 BLM RBX1 EIF2AK2 WAPL ATF7IP PDPK1 CCNE1 NEK4 TCF7L2 TFCP2 ITCH PLEKHM2 MYSM1 TP53BP1 STMP1 ROCK2 MEF2A CSNK1G3 CASK STAT3 EPN2 KDM2B OTUD4 SIRT2 CIT FOXF2 TNRC6B MAP3K2 BRCA2 RBL2 SLC39A10 CCNY NFAT5 EPS15 WRAP53 ZXDC RPTOR ARHGAP8 SHTN1 MYO10 DLC1 RNF111 KLHL22 SMAP1 TCF12 DTNBP1 PPP2R3C ZMYND8 TRAK1 BRAF WDFY1 PTPN9 BCL11A*

			SIRT1 CARD8 TNFRSF10B KMT5B ZNF143 ITSN2 CLASP1 RAPGEF1 WWOX MAP2K4 RREB1 MAP2K5 TRIM14 PIP4K2B BTRC IFT172 CROCC FCHSD2 TBC1D20 CHFR CLEC16A ATL3 EPB41L5 CNOT6L XPO4 RNF157 SPIRE2 IFT74 ARHGEF26 CTNNBIP1 STK39 ACSL1 FBXW4 TET1 MED12L NFIX MOB1B PPARD DOCK11 ABHD17C DHRSX GAREM1 RFX2 NEDD4L TNRC6C FAM161A CREM LPAR3 PLCB1 HIVEP1 TRPC1 PLXND1 DSTYK GMEB2 CLTCL1 PRKCA TBC1D5 PRDM10 RPS6KA2 SPRED2 NPAS2 KANK1 EFNB1 FZD8 P2RX4 ABAT DOCK5 COBL PRKD1 ADGRV1 LEPR TNFAIP8 PLAUR FAXDC2 INPP5F EPHA6 CCDC3 WDFY2 FBXO4 NLGN1 RIN2 FLI1 GRIN2D PPM1N ACTN2 SHC2 SALL4 EYA2 BMPR1B ASXL3 SORBS1 SVIL WNT5B NEDD4 CAMK1D ETV6 ADAMTS9 EDA MAGI2 KALRN WNT3 ESRRB ADAM19 KIF26B ACER2 RNF165 HIVEP3 ZBTB20 AGAP2 ZP3 KHDC1 RFX3 GJC2 AQP11 GLIS3 TNC AKAP6 ANK1 DOCK8 ERBB4 APLF POU2F2 C1QTNF4 NLRC4 SCIN NEDD9 NTRK2 PROC TRAF1 SULT2B1 NEURL1 CLDN3 ATP8A2 NOS1 SP100 AC019117.3 PTPN22 LEFTY1 CHODL DTX1 SLCO3A1 LRRN3 GPR68 IL7 FCAR KIAA0319 ALK ZNF43 SLC11A1 CD247 BANK1 TOX SH2D1A LAPTM5 NPSR1 ADRA1D PCDH11Y TENM2 OPRM1 KCNC2 CYP7B1 RHEX KCNK2 VIT SEMA5A TFAP2D LINGO2 APBB1IP NLRP2 DSCAM CACNG2 NFAM1 IL1RAPL1
GO:0048583	regulation of response to stimulus	1.579639E-02	*SEC14L1 PDE3A PID1 UTRN DLG2 EYA4 LYN RELN PPEF2 BMP8B DMD PLCG2 RGS3 DNAJC7 PAK1 LRP8 NOTCH2NLA ZNRF3 SLC8B1 NKX2-1 RUNX1 GSG1L RASGRF1 CD80 DKK2 APP CTNNA1 MAPK1 NUP98 BCR NUCB2 NAMPT LGMN PIM3 ARHGAP21 RAP1A ABR NOTCH2 BMP7 OGG1 PPP3CA MAP3K3 ATXN7 ABCC1 AKAP13 USP13 TIAM1 GTF2IRD1 HERC4 GLI3 PHLPP1 FZD6 ARHGEF4 CEBPA HDAC4 SASH1 RFTN1 FBXL17 FOXF1 ROBO2 ZSWIM4 GPC5 CD74 FLT3 FEZF2 HHLA2 NONO HSP90AA1 TMBIM6 PSMB2 SUMO1 GDI1 FOXM1 HIF1A CAMTA1 NCOR1 PUM1 YY1 FXR1 UBE2V2 CUL1 MDM2 PTPN11 WLS HELLS NPLOC4 DNAJC2 PDCD4 AUTS2 SPAG9 RNF220 ROBO1 FAF1 NUP133 WDR59 GIPC1 CHD8 TSG101 WNK2 CCDC88A AP1G1 BRCA1 TERF2 TMED4 SPG21 PIAS1 EHMT1 CHD3 FGFR1 DLX2 RC3H2 NXN RHOQ AIDA GBF1 SELENOS RHBDD3 MBD2 STK11 EIF2B4 CHEK1 NKX2-5 ARHGEF2 TSPYL2 TELO2 POR SOS1 DENND4B PTPN1 GCLC KCTD10 MAP2K7 BLM RBX1 EIF2AK2 PDPK1 NEK4 TCF7L2 PIGU ITCH TP53BP1 ROCK2 MEF2A CSNK1G3 CASK STAT3 EPN2 POLR3G OTUD4 SIRT2 MAP3K2 SLC39A10 CCNY NFAT5 RANBP9 RNF149 EPS15 WRAP53 RPTOR ARHGAP8 MYO10 DLC1 RNF111 KLHL22 DTNBP1 PPP2R3C WDR76 ZMYND8 BRAF IFT81 BRAP WDFY1 SIRT1 CARD8 TNFRSF10B KMT5B MYO9A DENND4C CLASP1 RAPGEF1 WWOX MAP2K4 ANKS1A CYTH1 RREB1 MAP2K5 TRIM14 FUT8 PIP4K2B BTRC IFT172 USP18 WIPF2 DENND4A RHBDF2 OTUD7B CLEC16A RCAN1 DEPDC5 TMEM131L OXR1 RNF157 ERRFI1 SPIRE2 ARHGEF26 CTNNBIP1 DNASE1 STK39 RALGAPA2 PPARD RHOT1 GAREM1 MCC LPAR3 TBCK MTUS1 PLCB1 MTM1 POLH DSTYK PRKCA TLE2 SPRED2 NPAS2 KANK1 FZD8 P2RX4 ABAT PROS1 PRKD1 ADGRV1 PLAUR INPP5F CCDC3 FBXO4 NLGN1 OPHN1 PPM1N ACTN2 SHC2 EYA2 BMPR1B RNF43 SORBS1 WNT5B NEDD4 CAMK1D EDA ARFGEF3 MAGI2 KALRN WNT3 LDLRAD4 C1QL4 RNF165 AGAP2 ZP3 OTUD7A AQP11 AKAP6 ERBB4 APLF C1QTNF4 MGLL NLRC4 CORO2B PDE2A NTRK2 CTNND2 PROC TRAF1 NEURL1 CLDN3 NOS1 SP100 PTPN22 LEFTY1 DTX1 DLGAP1 IL7 FCAR KIAA0319 ALK SLC11A1 CD247 TAFA1 BANK1 SH2D1A LAPTM5 NME5 ADRA1D PCDH11Y PTPRR OPRM1 CYP7B1 IGSF1 KCNK2 SEMA5A SH3TC2 CLEC4C APOH DCC PTPRT DSCAM CACNG2 NFAM1*
GO:0048519	negative regulation of biological process	1.599274E-02	*SEC14L1 PDE3A RYR2 PID1 DLG2 SORCS2 MIR663A EYA4 LYN EIF3E ADAMTS8 PPEF2 HDGFL3 MYO1D DMD PLCG2 RGS3 NCL GIGYF2 RERE PAK1 ZNRF3 POU3F3 SLC8B1 NKX2-1 RUNX1 TEX14 CD80 DKK2 APP CTNNA1 FOXC1 MAPK1 NUP98 BCR NUCB2 OTUD5 NAMPT OAZ2 ACTN1 ZNF282 VCL LGMN KDM2A PIM3 PUS7 HOXB3 RAP1A ABR NOTCH2 BMP7*

			OGG1 PPP3CA CDK5RAP2 ZNF131 MAP3K3 ATXN7 THADA KMT2B GTF2IRD1 PTPRG HERC4 GLI3 PHLPP1 FZD6 ZFHX3 CEBPA FAM172A HDAC4 AHR FOXF1 EPHA3 ROBO2 RYR3 CD74 CPAMD8 RYR1 CACNA1A FEZF2 NONO RPL35A SF3B3 ST13 ILF3 TMBIM6 SNRNP70 DNMT1 HNRNPR ACIN1 PRCC CCNB1 ID2 PSMB2 SUMO1 IPO7 CDC123 IPO5 GDI1 ELAVL1 YWHAH SND1 BPTF FOXM1 HIF1A UBP1 NAA38 NCOR1 PUM1 YY1 FXR1 DDX6 EXOSC10 CUL1 ADD1 MDM2 PTPN11 HELLS NPLOC4 DNAJC2 UHRF1 PDCD4 SPAG9 SARNP ROBO1 RBFOX2 NAA15 NSD1 NUP133 PCM1 GIPC1 CHD8 TFDP1 TSG101 WNK2 BRCA1 TERF2 CNOT6 PDS5A HDAC8 DENND5A TNRC6A PIAS1 EHMT1 GTSE1 TFDP2 CHD3 FGFR1 DLX2 RC3H2 NXN PPME1 RHOQ AIDA PDS5B SELENOS RHBDD3 MBD2 TBCD STK11 CHEK1 NKX2-5 RNF4 ARHGEF2 TSPYL2 POR ALDH1A2 PTPN1 GCLC IDE MTA3 KCTD10 SRPK2 BLM RBX1 EIF2AK2 WAPL ATF7IP PRMT3 PDPK1 CCNE1 TCF7L2 ITCH SCMH1 TP53BP1 PSMG2 LSM3 ROCK2 MEF2A CASK STAT3 EPN2 KDM2B OTUD4 MYEF2 USP4 SIRT2 FOXF2 RAB11FIP3 TNRC6B BRCA2 RBL2 SLC39A10 RANBP9 RNF149 EPS15 RPTOR DLC1 KLHL22 DTNBP1 PEX14 WDR76 ZMYND8 CHMP4A BRAF BRAP KNL1 STRN PTPN9 BCL11A SIRT1 CARD8 TNFRSF10B WWP1 ALKBH1 KDM4B CLASP1 RAPGEF1 ZNF263 WWOX MAP2K4 RREB1 MAP2K5 TRIM14 BTRC SMG6 IFT172 HERC1 FOXN3 CHFR RHBDF2 OTUD7B CLEC16A ZNF620 PC RCAN1 EPB41L5 CNOT6L DEPDC5 TMEM131L OXR1 RNF157 ERRFI1 IFT74 CTNNBIP1 STK39 TET1 NFIX PPARD ABHD17C HES6 MCC NEDD4L TNRC6C CREM SNTA1 PLCB1 MTM1 HIVEP1 TRPC1 PLXND1 DSTYK PRKCA ZNF875 RPS6KA2 TLE2 SPRED2 NPAS2 KANK1 P2RX4 ABAT ZNFX1 FAM220A DOCK5 PROS1 JAZF1 PRKD1 ADGRV1 USP44 LEPR TNFAIP8 PLAUR BRSK1 IL17D INPP5F CCDC3 FBXO4 SOGA1 NLGN1 OPHN1 PPM1N ACTN2 SALL4 EYA2 BMPR1B RNF43 ASXL3 SVIL WNT5B CPEB2 NEDD4 MTSS1 CAMK1D ETV6 CDA ADAMTS9 ARFGEF3 INKA2 MAGI2 WNT3 ESRRB LDLRAD4 ACER2 C1QL4 LAMP3 ZBTB20 AGAP2 CHRM3 ZP3 OTUD7A RFX3 GJC2 DCLK1 AQP11 GLIS3 TNC AKAP6 DOCK8 ERBB4 FRY PLCL1 KANK3 TMEFF2 CRYM SCIN CORO2B PDE2A NTRK2 PROC SULT2B1 NEURL1 GRM7 CLDN3 HS3ST5 WFDC3 ATP8A2 NOS1 SP100 AC019117.3 PTPN22 ZNF141 LEFTY1 DTX1 PPP1R36 GPR68 IL7 KIAA0319 ALK SLC11A1 COL28A1 DNAJC15 BANK1 SH2D1A TTPA EXD1 WNT9B LAPTM5 NME5 NPSR1 PTPRR TENM2 OPRM1 CYP7B1 SORCS3 IGSF1 KCNK2 CNTN4 SEMA5A TFAP2D SPINK4 TMEM132D TDRD9 APOH DCC SERPINB11 PTPRT DSCAM IL1RAPL1
GO:0048518	positive regulation of biological process	1.836412E-02	*PDE3A RYR2 AAMDC PID1 UTRN PMF1 DPP10 EYA4 LYN RELN LRRTM3 EIF3E BMP8B PKP4 DMD PLCG2 NCL CAPNS1 GIGYF2 RERE PAK1 LRP8 NOTCH2NLA POU3F3 EPB41L4B NKX2-1 RUNX1 RASGRF1 CD80 DKK2 RAB11A APP CTNNA1 EPB41 FOXC1 MAPK1 NUP98 BCR NAMPT OAZ2 ACTN1 LGMN HOXB3 RAP1A ABR NOTCH2 BMP7 PPP3CA CDK5RAP2 ZNF131 MAP3K3 AMOTL1 ATXN7 ABCC1 AKAP13 KMT2B USP13 PLS1 TIAM1 GLI3 ZFHX3 ARHGEF4 CEBPA HDAC4 SASH1 AHR RFTN1 FOXF1 EPHA3 ROBO2 GPC5 ESRRG NKX2-3 CD74 FLT3 LAMA2 FEZF2 HHLA2 NONO HSP90AA1 ILF3 SNRNP70 DNMT1 HNRNPR ACIN1 UBE2M CCNB1 ID2 ARIH1 PSMB2 SUMO1 TAF1D CDC123 IPO5 MRPL12 GDI1 ELAVL1 CDC27 YWHAH NPEPPS MTCH2 BPTF FOXM1 HIF1A UBP1 CAMTA1 NCOR1 PUM1 YY1 FXR1 DDX6 GTF2F1 CUL1 SEPT9 ADD1 MDM2 PTPN11 WLS DNAJC2 UHRF1 PDCD4 AUTS2 SPAG9 RNF220 ROBO1 NAA15 NSD1 CDCA5 PPP1R12A FAF1 PCM1 WDR59 GIPC1 CHD8 POLDIP3 ELP6 TFDP1 CLSTN1 TSG101 WNK2 CCDC88A CDC23 AP1G1 PPP1R7 BRCA1 TERF2 CNOT6 HDAC8 HK1 TNRC6A MLXIP TMED4 SSBP4 SCP2 SPG21 PIAS1 EHMT1 GTSE1 GOLGA4 TFDP2 FGFR1 DLX2 ARID1B RC3H2 RHOQ RHBDD3 MBD2 STK11 EIF2B4 CHEK1 ICE2 NKX2-5 RNF4 PDE4DIP ARHGEF2 TELO2 POR SOS1 ALDH1A2 PTPN1 GCLC IDE MTA3 AP3B1 MAP2K7 SRPK2 BLM RBX1 EIF2AK2 WAPL ATF7IP PDPK1 CCNE1 NEK4*

			TCF7L2 TFCP2 ITCH PLEKHM2 MYSM1 TP53BP1 STMP1 ROCK2 MEF2A CSNK1G3 CASK STAT3 EPN2 POLR3G KDM2B OTUD4 SIRT2 CIT FOXF2 TNRC6B MAP3K2 BRCA2 RBL2 SLC39A10 VPS37B CCNY NFAT5 RANBP9 EPS15 WRAP53 ZXDC RPTOR ARHGAP8 SHTN1 MYO10 DLC1 RNF111 KLHL22 SMAP1 TCF12 DTNBP1 PPP2R3C ZMYND8 TRAK1 BRAF WDFY1 PTPN9 BCL11A SIRT1 CARD8 TNFRSF10B KMT5B WWP1 ZNF143 ITSN2 CLASP1 RAPGEF1 WWOX MAP2K4 RREB1 MAP2K5 TRIM14 PIP4K2B BTRC IFT172 CROCC WIPF2 FCHSD2 TBC1D20 CHFR CLEC16A ATL3 PC EPB41L5 CNOT6L XPO4 RNF157 FRMD8 SPIRE2 IFT74 ARHGEF26 CTNNBIP1 STK39 DENND5B ACSL1 FBXW4 TET1 MED12L NFIX MOB1B PPARD DOCK11 ABHD17C DHRSX GAREM1 RFX2 NEDD4L TNRC6C FAM161A CREM LPAR3 PLCB1 MTM1 HIVEP1 TRPC1 PLXND1 DSTYK GMEB2 CLTCL1 PRKCA TBC1D5 PRDM10 RPS6KA2 SPRED2 NPAS2 KANK1 EFNB1 FZD8 P2RX4 ABAT DOCK5 PROS1 COBL PRKD1 ADGRV1 LEPR TNFAIP8 PLAUR IL17D FAXDC2 INPP5F EPHA6 CCDC3 WDFY2 FBXO4 NLGN1 RIN2 FLI1 GRIN2D PPM1N ACTN2 SHC2 SALL4 EYA2 BMPR1B ASXL3 SORBS1 SVIL WNT5B NEDD4 CAMK1D ETV6 ADAMTS9 EDA RAB3B MAGI2 KALRN WNT3 ESRRB ADAM19 KIF26B ACER2 RNF165 HIVEP3 LAMP3 ZBTB20 AGAP2 CHRM3 ZP3 KHDC1 RFX3 GJC2 AQP11 GLIS3 TNC AKAP6 ANK1 DOCK8 ERBB4 APLF POU2F2 C1QTNF4 NLRC4 SCIN CORO2B PDE2A NEDD9 NTRK2 PROC TRAF1 SULT2B1 NEURL1 CLDN3 ATP8A2 NOS1 SP100 AC019117.3 PTPN22 LEFTY1 CHODL DTX1 SLCO3A1 LRRN3 GPR68 HRH2 IL7 FCAR KIAA0319 ALK ZNF43 SLC11A1 CD247 BANK1 TOX SH2D1A TTPA LAPTM5 NPSR1 ADAM12 DIO2 ADRA1D PCDH11Y TENM2 OPRM1 KCNC2 CYP7B1 RHEX KCNK2 VIT SEMA5A TFAP2D LINGO2 APBB1IP CLEC4C APOH NLRP2 DSCAM CACNG2 NFAM1 IL1RAPL1
GO:0198738	cell-cell signaling by wnt	1.854263E-02	*PLCG2 ZNRF3 RUNX1 DKK2 APP PPP3CA AMOTL1 TIAM1 GLI3 FZD6 GPC5 GNB1 PSMB2 CUL1 WLS RNF220 CHD8 WNK2 TNRC6A NXN MBD2 STK11 NKX2-5 LRRFIP2 RBX1 CCNE1 TCF7L2 CSNK1G3 TNRC6B CCNY STRN RAPGEF1 WWOX BTRC TMEM131L CTNNBIP1 FBXW4 MCC TNRC6C PLCB1 TLE2 CELSR1 KANK1 FZD8 PPM1N RNF43 WNT5B EDA MAGI2 WNT3 PRICKLE2 CTNND2 WNT9B PCDH11Y OPRM1 SEMA5A*
GO:0009966	regulation of signal transduction	2.003031E-02	*SEC14L1 PDE3A PID1 DLG2 EYA4 LYN RELN PPEF2 BMP8B DMD RGS3 PAK1 NOTCH2NLA ZNRF3 NKX2-1 RUNX1 GSG1L RASGRF1 CD80 DKK2 APP CTNNA1 MAPK1 BCR LGMN ARHGAP21 RAP1A ABR NOTCH2 BMP7 MAP3K3 ATXN7 AKAP13 TIAM1 HERC4 GLI3 PHLPP1 FZD6 ARHGEF4 SASH1 FBXL17 ROBO2 GPC5 CD74 FLT3 NONO HSP90AA1 TMBIM6 PSMB2 SUMO1 GDI1 FOXM1 HIF1A CAMTA1 NCOR1 PUM1 MDM2 PTPN11 WLS HELLS NPLOC4 PDCD4 AUTS2 SPAG9 RNF220 ROBO1 FAF1 WDR59 GIPC1 CHD8 TSG101 WNK2 CCDC88A BRCA1 TMED4 PIAS1 EHMT1 CHD3 FGFR1 DLX2 RC3H2 NXN RHOQ AIDA GBF1 SELENOS MBD2 STK11 CHEK1 NKX2-5 ARHGEF2 TSPYL2 TELO2 POR SOS1 DENND4B PTPN1 GCLC KCTD10 MAP2K7 BLM RBX1 EIF2AK2 PDPK1 TCF7L2 PIGU ITCH ROCK2 CSNK1G3 STAT3 EPN2 OTUD4 MAP3K2 SLC39A10 CCNY NFAT5 RANBP9 RNF149 EPS15 RPTOR ARHGAP8 DLC1 RNF111 KLHL22 DTNBP1 ZMYND8 BRAF IFT81 BRAP WDFY1 SIRT1 CARD8 TNFRSF10B MYO9A DENND4C RAPGEF1 WWOX MAP2K4 ANKS1A CYTH1 MAP2K5 TRIM14 PIP4K2B BTRC IFT172 USP18 DENND4A RHBDF2 OTUD7B CLEC16A RCAN1 DEPDC5 TMEM131L RNF157 ERRFI1 ARHGEF26 CTNNBIP1 STK39 RALGAPA2 PPARD RHOT1 GAREM1 MCC LPAR3 TBCK PLCB1 MTM1 DSTYK PRKCA TLE2 SPRED2 KANK1 FZD8 P2RX4 ABAT PRKD1 ADGRV1 PLAUR INPP5F CCDC3 NLGN1 OPHN1 PPM1N ACTN2 SHC2 EYA2 BMPR1B RNF43 SORBS1 WNT5B NEDD4 EDA ARFGEF3 MAGI2 KALRN WNT3 LDLRAD4 C1QL4 RNF165 AGAP2 OTUD7A AKAP6 ERBB4 C1QTNF4 MGLL PDE2A NTRK2 CTNND2 TRAF1 NEURL1 NOS1 SP100 PTPN22 LEFTY1 DTX1 DLGAP1 IL7 KIAA0319 ALK TAFA1 BANK1 SH2D1A LAPTM5 NME5 ADRA1D PCDH11Y PTPRR OPRM1 CYP7B1 IGSF1 SEMA5A SH3TC2 DCC PTPRT CACNG2 NFAM1*

GO:0048869	cellular developmental process	2.083791E-02	*UNC45B PDE3A AAMDC PID1 EYA4 LYN RELN BMP8B HDGFL3 PKP4 MAP1S MEX3C DMD PLCG2 CAPNS1 GIGYF2 RERE PAK1 LRP8 NOTCH2NLA KAZN POU3F3 NKX2-1 RUNX1 PDE1B RASGRF1 CD80 RAB11A APP CTNNA1 FOXC1 MAPK1 BCR NAMPT ACTN1 VCL PUS7 HOXB3 RAP1A NOTCH2 BMP7 PPP3CA CDK5RAP2 MAP3K3 AKAP13 KMT2B USP13 PLS1 TIAM1 PTPRG HERC4 GLI3 ZFHX3 CEBPA FAM172A HDAC4 SYNE1 NEBL FBXL17 FOXF1 EPHA3 WDPCP ROBO2 ZSWIM4 NKX2-3 CD74 RYR1 NUP210L CACNA1A OPCML FLT3 LAMA2 FEZF2 HSP90AA1 DNMT1 ACIN1 CCNB1 ID2 PSMB2 GDI1 YWHAH SND1 FOXM1 HIF1A PUM1 YY1 FXR1 DDX6 ADD1 MDM2 PTPN11 PDCD4 AUTS2 SPAG9 ROBO1 RBFOX2 NAA15 ACADM NUP133 PCM1 ASF1B TSG101 CCDC88A AP1G1 SEPT6 TERF2 SETD1A DENND5A TNRC6A PIAS1 GOLGA4 FGFR1 DLX2 ARID1B RC3H2 NXN TBCD STK11 EIF2B4 UBE4B CHEK1 NKX2-5 NDE1 ARHGEF2 POR SOS1 ALDH1A2 AP3B1 SRPK2 PDLIM5 EIF2AK2 OGDH PDPK1 NEK4 TCF7L2 ITCH ROCK2 MEF2A STAT3 MYEF2 SIRT2 CIT FOXF2 TNRC6B RAI14 BRCA2 RBL2 RANBP9 SH3PXD2A SHTN1 TAF8 SMAP1 TCF12 DTNBP1 PPP2R3C ZMYND8 TRAK1 BRAF KNL1 STRN PTPN9 BCL11A SIRT1 MYO9A ALKBH1 HS6ST1 ITSN2 CLASP1 RAPGEF1 WWOX MAP2K4 ANKS1A RREB1 CASP7 IFT172 HERC1 TBC1D20 TPRN RCAN1 EPB41L5 TMEM131L RNF157 ERRFI1 IFT74 ARHGEF26 CTNNBIP1 TET1 PPARD DOCK11 HES6 RFX2 NEDD4L TNRC6C CREM LPAR3 PLCB1 DIAPH2 MTM1 UST PLXND1 PRKCA RPS6KA2 CELSR1 SPRED2 FOXN2 KANK1 EFNB1 FZD8 P2RX4 IQCG DOCK5 COBL SHROOM3 LRRC8C PRKD1 ADGRV1 SNX10 LEPR BRSK1 FAXDC2 INPP5F EPHA6 CCDC3 WDFY2 NLGN1 RIN2 OPHN1 FLI1 OSBP2 ACTN2 EYA2 BMPR1B WNT5B NEDD4 MTSS1 CAMK1D ETV6 EDNRA ADAMTS9 PPFIA2 EDA RBM47 MAGI2 WNT3 ESRRB LDLRAD4 RILPL2 C1QL4 RNF165 HIVEP3 NHS ZP3 NFASC RFX3 GJC2 DCLK1 TNC AKAP6 ERBB4 FRY POU2F2 IGSF10 TMEFF2 SCIN PDE2A NEDD9 UNC5A NTRK2 CTNND2 PROC MEI1 SULT2B1 NEURL1 CLDN3 UPK1B ATP8A2 CCDC36 NOS1 PTPN22 CHODL DTX1 GPR68 HRH2 IL7 KIAA0319 ALK DOK6 MOBP TAFA1 NTM SLC2A14 TOX TTPA WNT9B SLC4A10 PCDH15 NME5 ADAM12 DIO2 PTPRQ TRPM1 HCN1 TENM2 OPRM1 BHLHE22 SYCP1 RHEX CNTN4 RXFP1 SEMA5A SH3TC2 KRTAP5-11 TDRD9 DCC DSCAM NFAM1 IL1RAPL1*
GO:0045595	regulation of cell differentiation	2.151862E-02	*PDE3A AAMDC LYN RELN MEX3C DMD PAK1 LRP8 NKX2-1 RUNX1 CD80 RAB11A APP CTNNA1 FOXC1 MAPK1 VCL PUS7 HOXB3 RAP1A NOTCH2 BMP7 PPP3CA CDK5RAP2 KMT2B TIAM1 GLI3 ZFHX3 CEBPA HDAC4 EPHA3 ROBO2 CD74 FEZF2 DNMT1 ACIN1 ID2 PSMB2 GDI1 YWHAH HIF1A YY1 DDX6 ADD1 MDM2 PTPN11 PDCD4 SPAG9 ROBO1 RBFOX2 PCM1 SETD1A TNRC6A PIAS1 GOLGA4 FGFR1 DLX2 RC3H2 STK11 NKX2-5 ARHGEF2 POR SOS1 AP3B1 EIF2AK2 TCF7L2 ITCH ROCK2 MEF2A STAT3 SIRT2 TNRC6B SHTN1 TAF8 SMAP1 TCF12 PPP2R3C TRAK1 BRAF BCL11A SIRT1 CLASP1 RREB1 RCAN1 TMEM131L ERRFI1 CTNNBIP1 PPARD HES6 TNRC6C LPAR3 PLCB1 PLXND1 PRKCA SPRED2 KANK1 DOCK5 PRKD1 ADGRV1 FAXDC2 CCDC3 WDFY2 NLGN1 RIN2 BMPR1B WNT5B ADAMTS9 WNT3 ESRRB LDLRAD4 C1QL4 RFX3 GJC2 AKAP6 SCIN NEDD9 NTRK2 PROC SULT2B1 NEURL1 NOS1 CHODL DTX1 GPR68 IL7 KIAA0319 ALK TOX TTPA WNT9B OPRM1 RHEX CNTN4 SEMA5A DCC DSCAM NFAM1 IL1RAPL1*
GO:0050793	regulation of developmental process	2.430020E-02	*PDE3A AAMDC PID1 LYN RELN LRRTM3 MEX3C DMD PAK1 LRP8 ZNRF3 NKX2-1 RUNX1 CD80 RAB11A APP CTNNA1 EPB41 FOXC1 MAPK1 BCR NAMPT VCL LGMN PUS7 HOXB3 RAP1A ABR NOTCH2 BMP7 PPP3CA CDK5RAP2 MAP3K3 KMT2B PLS1 TIAM1 GLI3 FZD6 ZFHX3 CEBPA HDAC4 SASH1 EPHA3 WDPCP ROBO2 CD74 CACNA1A LAMA2 FEZF2 DNMT1 ACIN1 CCNB1 ID2 PSMB2 GDI1 ELAVL1 YWHAH FOXM1 HIF1A YY1 DDX6 ADD1 MDM2 PTPN11 PDCD4 SPAG9 ROBO1 RBFOX2 PCM1 CLSTN1 BRCA1 TERF2 SETD1A TNRC6A PIAS1 EHMT1 GOLGA4 FGFR1 DLX2*

			RC3H2 RHOQ STK11 NKX2-5 ARHGEF2 POR SOS1 AP3B1 PDLIM5 EIF2AK2 PDPK1 NEK4 TCF7L2 ITCH MYSM1 TP53BP1 ROCK2 MEF2A STAT3 EPN2 KDM2B SIRT2 TNRC6B SHTN1 MYO10 DLC1 TAF8 SMAP1 TCF12 PPP2R3C ZMYND8 TRAK1 BRAF BCL11A SIRT1 KMT5B MYO9A ITSN2 CLASP1 RREB1 RCAN1 TMEM131L RNF157 ERRFI1 CTNNBIP1 PPARD HES6 NEDD4L TNRC6C LPAR3 PLCB1 MTM1 UST PLXND1 PRKCA CELSR1 SPRED2 KANK1 DOCK5 COBL SHROOM3 PRKD1 ADGRV1 BRSK1 IL17D FAXDC2 INPP5F CCDC3 WDFY2 NLGN1 RIN2 BMPR1B WNT5B NEDD4 CAMK1D ADAMTS9 PPFIA2 MAGI2 WNT3 ESRRB LDLRAD4 C1QL4 AGAP2 ZP3 RFX3 GJC2 AKAP6 ERBB4 SCIN PRICKLE2 NEDD9 NTRK2 PROC SULT2B1 NEURL1 CLDN3 ATP8A2 NOS1 SP100 CHODL DTX1 LRRN3 GPR68 IL7 KIAA0319 ALK TOX TTPA WNT9B ADAM12 OPRM1 RHEX KCNK2 CNTN4 SEMA5A LINGO2 APOH DCC DSCAM NFAM1 IL1RAPL1
GO:0050767	regulation of neurogenesis	2.631565E-02	*LYN RELN PAK1 LRP8 RAB11A CTNNA1 HOXB3 BMP7 PPP3CA TIAM1 GLI3 ROBO2 FEZF2 ID2 GDI1 YWHAH HIF1A ROBO1 PCM1 GOLGA4 DLX2 STK11 ARHGEF2 SIRT2 SHTN1 TRAK1 BRAF BCL11A HES6 LPAR3 PLXND1 WNT3 GJC2 NTRK2 NEURL1 NOS1 CHODL KIAA0319 OPRM1 SEMA5A DCC DSCAM IL1RAPL1*
GO:1903827	regulation of cellular protein localization	2.698246E-02	*PID1 DPP10 PAK1 GSG1L RAB11A CTNNA1 EPB41 MAPK1 OAZ2 RAP1A STX8 PLS1 GLI3 EPHA3 GPC5 HSP90AA1 IPO5 GDI1 YWHAH NPEPPS MDM2 PTPN11 CDCA5 PCM1 TFDP1 TSG101 CCDC88A HDAC8 GTSE1 TFDP2 RHOQ GBF1 STK11 PTPN1 WAPL PDPK1 CCNE1 TCF7L2 ROCK2 WRAP53 ZMYND8 PTPN9 CROCC OTUD7B XPO4 ABHD17C NEDD4L PRKD1 FBXO4 OPHN1 ACTN2 SORBS1 DCLK1 ERBB4 SP100 PTPN22 CD247 SH3TC2 CACNG2*
GO:0051094	positive regulation of developmental process	3.036587E-02	*PDE3A AAMDC LYN RELN LRRTM3 DMD PAK1 LRP8 RUNX1 CD80 RAB11A CTNNA1 FOXC1 MAPK1 RAP1A NOTCH2 BMP7 PLS1 TIAM1 GLI3 ZFHX3 CEBPA SASH1 ROBO2 CD74 FEZF2 ACIN1 CCNB1 ID2 GDI1 HIF1A ADD1 MDM2 SPAG9 ROBO1 CLSTN1 BRCA1 PIAS1 GOLGA4 FGFR1 DLX2 STK11 NKX2-5 ARHGEF2 POR AP3B1 PDPK1 TCF7L2 TP53BP1 MEF2A STAT3 KDM2B SIRT2 SHTN1 SMAP1 TCF12 PPP2R3C ZMYND8 TRAK1 BRAF BCL11A SIRT1 KMT5B ITSN2 CLASP1 RREB1 RNF157 CTNNBIP1 PPARD NEDD4L LPAR3 PLCB1 MTM1 PLXND1 PRKCA DOCK5 COBL PRKD1 ADGRV1 FAXDC2 CCDC3 WDFY2 NLGN1 RIN2 BMPR1B WNT5B ADAMTS9 WNT3 ESRRB AGAP2 ZP3 RFX3 GJC2 AKAP6 ERBB4 SCIN NEDD9 NTRK2 PROC SULT2B1 NEURL1 ATP8A2 CHODL LRRN3 GPR68 IL7 ALK TOX ADAM12 OPRM1 RHEX SEMA5A LINGO2 DSCAM IL1RAPL1*
GO:0051641	cellular localization	3.052171E-02	*RYR2 PID1 DLG2 SORCS2 DPP10 LYN RELN ABCA13 MYO1D MAP1S DMD PLCG2 PAK1 SLC8B1 BSN GSG1L RAB11A APP CTNNA1 EPB41 MAPK1 NUP98 BCR OAZ2 ACTN1 VCL PIM3 ARHGAP21 RAP1A ABR BMP7 PPP3CA CDK5RAP2 SNX27 AKAP13 STX8 TRAPPC11 PLS1 TIAM1 GLI3 SYNE1 RFTN1 FOXF1 EPHA3 IMMP2L RYR3 GPC5 SGSM1 CD74 RYR1 CACNA1A KCNN2 HSP90AA1 RPL35A TUBA1C TMBIM6 ATP5MC2 SNRPD1 CCNB1 SUMO1 UBE2D2 IPO7 IPO5 PDAP1 GDI1 ELAVL1 YWHAH NPEPPS MTCH2 GGA2 SLBP SRP68 HIF1A EXOSC10 SEPT9 MDM2 PTPN11 WLS NPLOC4 TRAPPC5 SPAG9 SARNP PDXK CDCA5 PPP1R12A NUP133 PCM1 GIPC1 POLDIP3 TFDP1 COPB1 CLSTN1 TSG101 ATAD3B CCDC88A CDC23 PREPL AP1G1 SEPT6 TERF2 HDAC8 HK1 DENND5A ESYT1 TMED4 TM9SF3 SCP2 GTSE1 GOLGA4 TFDP2 RHOQ GBF1 SELENOS STK11 NDE1 ARHGEF2 PTPN1 IDE AP3B1 WAPL ANKRD13C PDPK1 CCNE1 TCF7L2 PIGU PLEKHM2 ARFGAP3 ROCK2 MEF2A CASK STAT3 SNUPN USP4 CENPA TMEM167A RAB11FIP3 BRCA2 VPS37B EPS15 WRAP53 CDC40 RABGAP1L MYO10 OFD1 TAF8 DTNBP1 PEX14 ZMYND8 TRAK1 CHMP4A BRAF IFT81 ASB3 KNL1 PTPN9 SIL1 DENND4C ITSN2 CLASP1 KIAA0753 COG5 SMG6 IFT172 CROCC HEATR5B TBC1D20 OTUD7B CLEC16A RTN2 XPO4 RNF157 SPIRE2 AFTPH IFT74 FBF1 PPARD DMAC2L ABHD17C RHOT1 NEDD4L*

			SNTA1 TBCK TBC1D22A MTM1 TRPC1 CEP76 TMCC1 CLTCL1 TBC1D5 CELSR1 P2RX4 PROS1 AP4S1 ERC2 PRKD1 SNX10 PLAUR BRSK1 KCNMB4 INPP5F FBXO4 NLGN1 OPHN1 BTBD9 GRIN2D ACTN2 SORBS1 NEDD4 STON1 CDA KCNB2 PPFIA2 EFR3B RAB3B MAGI2 SYN3 RILPL2 FAM53A LAMP3 AGAP2 NFASC DCLK1 AQP11 AKAP6 ANK1 ERBB4 DYNC1I1 SCG5 SCIN REPS2 PROC WDR72 MEI1 NOS1 SP100 PTPN22 KCNIP4 GPR68 FCAR RGPD3 SLC11A1 DNAJC15 TERB2 CD247 LAPTM5 DNAH5 NPSR1 LYZ TRPM1 UNC13C FAM155A SH3TC2 CLEC4C DAO APOH CACNG2 NFAM1
GO:0016055	Wnt signaling pathway	3.375513E-02	*PLCG2 ZNRF3 RUNX1 DKK2 APP PPP3CA AMOTL1 TIAM1 GLI3 FZD6 GPC5 GNB1 PSMB2 CUL1 WLS RNF220 CHD8 WNK2 TNRC6A NXN MBD2 STK11 NKX2-5 LRRFIP2 RBX1 CCNE1 TCF7L2 CSNK1G3 TNRC6B CCNY STRN RAPGEF1 WWOX BTRC TMEM131L CTNNBIP1 FBXW4 MCC TNRC6C PLCB1 TLE2 CELSR1 KANK1 FZD8 PPM1N RNF43 WNT5B EDA MAGI2 WNT3 PRICKLE2 CTNND2 WNT9B PCDH11Y SEMA5A*
GO:0065009	regulation of molecular function	4.078103E-02	*RYR2 UTRN DLG2 LYN RELN EIF3E PPEF2 PKP4 MYO1D DMD PLCG2 RGS3 PAK1 LRP8 TEX14 GSG1L RASGRF1 DKK2 APP EPB41 FOXC1 MAPK1 BCR OAZ2 LGMN ARHGAP21 RAP1A ABR BMP7 PPP3CA MAP3K3 THADA AKAP13 TIAM1 FZD6 CEBPA HDAC4 SASH1 EPHA3 GABBR2 SGSM1 CD74 CPAMD8 FLT3 Z82190.2 HSP90AA1 TMBIM6 HNRNPR CCNB1 ID2 SUMO1 IPO7 IPO5 GDI1 YWHAH HIF1A GTF2F1 ADD1 MDM2 PTPN11 UHRF1 PDCD4 SPAG9 ROBO1 CDCA5 PPP1R12A FAF1 TFDP1 TSG101 WNK2 CCDC88A PPP1R7 TERF2 HDAC8 FGFR1 GAPVD1 PPME1 AIDA MBD2 TBCD STK11 ARHGEF2 TSPYL2 TELO2 POR SOS1 PTPN1 IDE MAP2K7 BLM EIF2AK2 WAPL PDPK1 CCNE1 TCF7L2 ITCH ARFGAP3 TP53BP1 ROCK2 STAT3 SIRT2 MAP3K2 RAP1GDS1 RBL2 SLC39A10 CCNY WRAP53 SH3PXD2A RPTOR ARHGAP8 RABGAP1L DLC1 SMAP1 DTNBP1 PPP2R3C PEX14 BRAF KNL1 SIRT1 CARD8 TNFRSF10B MYO9A RAPGEF1 MAP2K4 MAP2K5 TRIM14 BTRC SMG6 TBC1D20 RCAN1 EPB41L5 PPP1R12B DEPDC5 ERRFI1 ARHGEF26 CTNNBIP1 STK39 ACSL1 RALGAPA2 MOB1B DOCK11 HES6 NEDD4L LPAR3 TBCK PLCB1 TBC1D22A PLXND1 DSTYK TBC1D5 VPS9D1 SPRED2 FZD8 DOCK5 PROS1 PRKD1 ADGRV1 USP44 LEPR TNFAIP8 PLAUR EPHA6 NLGN1 RIN2 OPHN1 ACTN2 SHC2 CPEB2 NEDD4 CAMK1D PPP1R3B EDNRA EDA ARFGEF3 INKA2 MAGI2 ACER2 LAMP3 AGAP2 CHRM3 KHDC1 AKAP6 DOCK8 ERBB4 FRY PLCL1 SCG5 NLRC4 NEDD9 NTRK2 TNXB TRAF1 NEURL1 CLDN3 WFDC3 NOS1 SP100 PTPN22 SLCO3A1 DLGAP1 PPP1R36 IL7 ALK RGPD3 SLC11A1 COL28A1 DNAJC15 TAFA1 WNT9B OPRM1 KCNC2 SPINK4 TMEM132D APOH SERPINB11 NLRP2 PTPRT CACNG2 NFAM1*
GO:1905114	cell surface receptor signaling pathway involved in cell-cell signaling	4.229324E-02	*RELN PLCG2 ZNRF3 RUNX1 DKK2 APP PPP3CA AMOTL1 TIAM1 GLI3 FZD6 GPC5 GNB1 PSMB2 CUL1 WLS RNF220 CHD8 WNK2 TNRC6A NXN MBD2 STK11 NKX2-5 LRRFIP2 RBX1 CCNE1 TCF7L2 CSNK1G3 TNRC6B CCNY ZMYND8 STRN RAPGEF1 WWOX BTRC TMEM131L CTNNBIP1 FBXW4 MCC TNRC6C PLCB1 TLE2 CELSR1 KANK1 FZD8 P2RX4 ABAT NLGN1 GRIN2D PPM1N RNF43 WNT5B EDA MAGI2 WNT3 PRICKLE2 CTNND2 WNT9B GLRA3 PCDH11Y OPRM1 SEMA5A*
GO:0030154	cell differentiation	4.248004E-02	*UNC45B PDE3A AAMDC EYA4 LYN RELN BMP8B HDGFL3 PKP4 MAP1S MEX3C DMD PLCG2 CAPNS1 GIGYF2 RERE PAK1 LRP8 NOTCH2NLA KAZN POU3F3 NKX2-1 RUNX1 PDE1B RASGRF1 CD80 RAB11A APP CTNNA1 FOXC1 MAPK1 BCR ACTN1 VCL PUS7 HOXB3 RAP1A NOTCH2 BMP7 PPP3CA CDK5RAP2 AKAP13 KMT2B USP13 PLS1 TIAM1 PTPRG HERC4 GLI3 ZFHX3 CEBPA FAM172A HDAC4 SYNE1 NEBL FBXL17 FOXF1 EPHA3 WDPCP ROBO2 ZSWIM4 NKX2-3 CD74 RYR1 NUP210L CACNA1A OPCML FLT3 LAMA2 FEZF2 HSP90AA1 DNMT1 ACIN1 CCNB1 ID2 PSMB2 GDI1 YWHAH SND1 FOXM1 HIF1A PUM1 YY1 FXR1 DDX6 ADD1 MDM2 PTPN11 PDCD4 AUTS2 SPAG9 ROBO1 RBFOX2 NAA15 ACADM NUP133 PCM1 ASF1B TSG101 CCDC88A AP1G1 SEPT6 SETD1A DENND5A TNRC6A*

			PIAS1 GOLGA4 FGFR1 DLX2 ARID1B RC3H2 NXN TBCD STK11 EIF2B4 UBE4B NKX2-5 NDE1 ARHGEF2 POR SOS1 ALDH1A2 AP3B1 SRPK2 PDLIM5 EIF2AK2 OGDH PDPK1 TCF7L2 ITCH ROCK2 MEF2A STAT3 MYEF2 SIRT2 CIT FOXF2 TNRC6B RAI14 BRCA2 RBL2 RANBP9 SH3PXD2A SHTN1 TAF8 SMAP1 TCF12 DTNBP1 PPP2R3C ZMYND8 TRAK1 BRAF KNL1 STRN PTPN9 BCL11A SIRT1 MYO9A ALKBH1 HS6ST1 ITSN2 CLASP1 RAPGEF1 WWOX MAP2K4 ANKS1A RREB1 CASP7 IFT172 HERC1 TBC1D20 TPRN RCAN1 EPB41L5 TMEM131L RNF157 ERRFI1 IFT74 ARHGEF26 CTNNBIP1 TET1 PPARD DOCK11 HES6 RFX2 NEDD4L TNRC6C CREM LPAR3 PLCB1 DIAPH2 UST PLXND1 PRKCA RPS6KA2 CELSR1 SPRED2 FOXN2 KANK1 EFNB1 FZD8 P2RX4 IQCG DOCK5 COBL SHROOM3 LRRC8C PRKD1 ADGRV1 SNX10 LEPR BRSK1 FAXDC2 INPP5F EPHA6 CCDC3 WDFY2 NLGN1 RIN2 OPHN1 FLI1 OSBP2 ACTN2 EYA2 BMPR1B WNT5B NEDD4 MTSS1 CAMK1D ETV6 EDNRA ADAMTS9 PPFIA2 EDA RBM47 MAGI2 WNT3 ESRRB LDLRAD4 RILPL2 C1QL4 RNF165 HIVEP3 NHS ZP3 NFASC RFX3 GJC2 DCLK1 TNC AKAP6 ERBB4 FRY POU2F2 IGSF10 TMEFF2 SCIN PDE2A NEDD9 UNC5A NTRK2 CTNND2 PROC MEI1 SULT2B1 NEURL1 CLDN3 UPK1B ATP8A2 CCDC36 NOS1 PTPN22 CHODL DTX1 GPR68 HRH2 IL7 KIAA0319 ALK DOK6 MOBP TAFA1 NTM SLC2A14 TOX TTPA WNT9B SLC4A10 PCDH15 NME5 ADAM12 DIO2 PTPRQ TRPM1 HCN1 TENM2 OPRM1 BHLHE22 SYCP1 RHEX CNTN4 RXFP1 SEMA5A SH3TC2 KRTAP5-11 TDRD9 DCC DSCAM NFAM1 IL1RAPL1
GO:0051239	regulation of multicellular organismal process	4.470872E-02	*PDE3A RYR2 UTRN LYN RELN CTNNA3 LRRTM3 DMD PLCG2 PAK1 LRP8 EPB41L4B NKX2-1 RUNX1 CD80 RAB11A APP CTNNA1 EPB41 FOXC1 MAPK1 BCR OTUD5 VCL LGMN HOXB3 ABR NOTCH2 BMP7 PPP3CA MAP3K3 AMOTL1 KMT2B PLS1 TIAM1 GTF2IRD1 PTPRG GLI3 HDAC4 SASH1 WDPCP ROBO2 RYR3 ESRRG CD74 RYR1 LAMA2 KCNN2 FEZF2 HHLA2 ACIN1 CCNB1 ID2 SUMO1 GDI1 ELAVL1 YWHAH HIF1A YY1 ADD1 MDM2 PTPN11 NPLOC4 PDCD4 ROBO1 RBFOX2 PCM1 CLSTN1 BRCA1 HK1 SETD1A TNRC6A EHMT1 GOLGA4 FGFR1 DLX2 RC3H2 SELENOS STK11 NKX2-5 ARHGEF2 POR SOS1 AP3B1 EIF2AK2 PDPK1 TCF7L2 ITCH MYSM1 TP53BP1 STMP1 ROCK2 MEF2A STAT3 EPN2 POLR3G KDM2B SIRT2 RAB11FIP3 TNRC6B BRCA2 SHTN1 SMAP1 PPP2R3C ZMYND8 TRAK1 BRAF ASB3 BCL11A SIRT1 CARD8 KMT5B CLASP1 RREB1 MAP2K5 OTUD7B EPB41L5 PPP1R12B TMEM131L FRMD8 ERRFI1 CTNNBIP1 STK39 ACSL1 PPARD HES6 MCC TNRC6C LPAR3 SNTA1 PLCB1 MTM1 TRPC1 PLXND1 PRKCA SPRED2 NPAS2 P2RX4 ABAT DOCK5 PROS1 PRKD1 ADGRV1 LEPR PLAUR IL17D KCNMB4 FAXDC2 NLGN1 RIN2 GRIN2D BMPR1B KCNB2 ADAMTS9 WNT3 ESRRB LDLRAD4 ZBTB20 AGAP2 CHRM3 ZP3 RFX3 GJC2 AKAP6 ERBB4 PLCL1 POU2F2 C1QTNF4 MGLL NLRC4 SCIN CORO2B NTRK2 PROC SULT2B1 NEURL1 HS3ST5 ATP8A2 NOS1 SP100 PTPN22 CHODL DTX1 DLGAP1 LRRN3 KCNIP4 GPR68 HRH2 IL7 KIAA0319 SLC11A1 KCNIP1 BANK1 TOX TTPA WNT9B LAPTM5 ADAM12 DIO2 ADRA1D HCRTR2 PTPRR OPRM1 RHEX KCNK2 SEMA5A LINGO2 APOH DCC NLRP2 DSCAM CACNG2 NFAM1 IL1RAPL1*
GO:0071417	cellular response to organonitrogen compound	4.678963E-02	*PDE3A RYR2 PID1 LYN PAK1 APP CTNNA1 MAPK1 NAMPT LGMN RAP1A ABCC1 AHR RYR3 RYR1 CACNA1A DNMT1 GNB1 IPO5 MDM2 PTPN11 BRCA1 ARID1B RHOQ SELENOS ARHGEF2 POR SOS1 PTPN1 GCLC IDE BLM PDPK1 ROCK2 STAT3 SIRT2 RPTOR KLHL22 DTNBP1 BCL11A SIRT1 DENND4C RAPGEF1 CASP7 ERRFI1 GNAL KANK1 HCN2 P2RX4 SOGA1 ACTN2 SORBS1 CPEB2 CHRM3 AKAP6 PDE2A NTRK2 NEURL1 PTPN22 HRH2 ALK HCN1 OPRM1 ATP6V1G3 HTR3D*
GO:0009653	anatomical structure morphogenesis	4.740975E-02	*RYR2 GCNT1 PID1 EYA4 RELN MAP1S DMD NCL RERE PAK1 LRP8 ZNRF3 NKX2-1 RUNX1 RAB11A APP CTNNA1 EPB41 FOXC1 MAPK1 BCR ACTN1 VCL HOXB3 RAP1A ABR NOTCH2 BMP7 PPP3CA MAP3K3 AMOTL1 AKAP13 PLS1 TIAM1 GLI3 FZD6 SASH1 NEBL FOXF1 EPHA3 WDPCP ROBO2 ZSWIM4 NKX2-3 SLC24A4 RYR1 CACNA1A LAMA2 FEZF2*

		HSP90AA1 ID2 PSMB2 GDI1 YWHAH FOXM1 HIF1A UBP1 YY1 CUL1 ADD1 MDM2 PTPN11 WLS PDCD4 AUTS2 SPAG9 ROBO1 RBFOX2 NAA15 BRCA1 GOLGA4 FGFR1 DLX2 RHOQ TBCD STK11 UBE4B NKX2-5 ARHGEF2 POR SOS1 ALDH1A2 AP3B1 SRPK2 PDLIM5 PDPK1 ROCK2 MEF2A STAT3 EPN2 KDM2B FOXF2 RANBP9 SH3PXD2A GREB1L SHTN1 MYO10 DLC1 DTNBP1 TRAK1 BRAF KNL1 BCL11A SIRT1 MYO9A HS6ST1 ITSN2 CLASP1 WWOX RREB1 MAP2K5 BTRC IFT172 HERC1 FOXN3 TBC1D20 TPRN EPB41L5 RNF157 ERRFI1 ARHGEF26 CTNNBIP1 FBXW4 TET1 RFX2 NEDD4L LPAR3 MTM1 UST PLXND1 CLTCL1 PRKCA LIAS TLE2 CELSR1 KANK1 EFNB1 FZD8 SP8 DOCK5 COBL SHROOM3 PRKD1 SNX10 LEPR BRSK1 EPHA6 NLGN1 RIN2 OPHN1 FLI1 ACTN2 SALL4 EYA2 BMPR1B ASXL3 MEGF11 WNT5B NEDD4 MTSS1 EDNRA ADAMTS9 PPFIA2 EDA MYO3A MAGI2 WNT3 RILPL2 KIF26B RNF165 ZP3 NFASC DCLK1 TNC CDH18 ERBB4 FRY TMEFF2 PRICKLE2 NEDD9 UNC5A NTRK2 CTNND2 WDR72 CLDN3 ATP8A2 NOS1 SP100 ZNF141 LEFTY1 CHODL HRH2 IL7 KIAA0319 DOK6 MOBP WNT9B SLC4A10 PCDH15 ADAM12 PTPRQ TRPM1 EVX2 HCN1 CYP7B1 CNTN4 RXFP1 SEMA5A APOH DCC DSCAM IL1RAPL1

Table S3. The overlap between srRNA genes and rDNA-contacting genes. Related to the Venn diagram in Fig. 1D.

**Names**	**total**	***elements***
4920 rsRNA genes 1584	426	*PKNOX2 EPB41L4B EPN2 BANK1 SYN3 CNTN4-AS2 STX8 ROCK2 DNAJC15 CCNE1 VAT1L OPHN1 FAM182B CAMTA1 WDFY2 IPP SLC13A4 TSPAN7 CDK17 LRRTM3 ZBTB20 HBG2 MOB1B HS1BP3 PCAT1 DLGAP1 RCAN1 SVOPL LRP1B OTUD7A KALRN SDHD KIAA0753 SNTA1 CACNG2 BTBD9 TAF1D MEGF11 NR2F2-AS1 RNF43 TRHDE MAP2K4 HS3ST5 MALL NELL2 ARID1B IL1RAPL1 WDPCP VPS37B GCLC ADAMTS6 CDC40 MKKS STK39 RPS6KA2 ARHGAP8 HFM1 CDRT4 EXD1 CTNNA1 NCOR1P1 MYO9A NTRK2 FOXN3 CCDC3 SH3TC2 MDM2 NDST3 RASGEF1B STAG1 ZNRF3 GLIS3 HBE1 PTPRG NCOR1 PLAC1 DIO2 SLC24A4 SYNE1 FBXL17 PDZD2 DNAH10 STPG2 TBC1D22A PDE4DIP SLC7A7 NHS SLC39A10 FTCDNL1 TANGO6 DMD STAG3L2 FNDC3B GNG12-AS1 CHRM3 CALD1 EIF3E HSF5 TMEM132D SPRED2 ITGAE MYO1D CCDC178 KAZN CWF19L2 PHKB GULP1 ABCA13 RAP1GDS1 RPTOR KANK1 KIF26B LPP CHST8 LAMA2 RAP1A TFDP2 SH2D1A DIAPH2 NOS1 STAG2 RGS3 NEK4 TBC1D5 CNTN4 TMC2 SLCO3A1 SND1 PDXK TCF12 CORO2B GRIK4 NPSR1-AS1 RUNX1 ALDH1A2 ESRRG FAR2 MIR663A TMEM132C CDH18 RAPGEF1 PLS1 ROBO1 ANKRD30BL PRR5-ARHGAP8 GPR176 PKP4 SEPT9 PLD5 PWRN1 RBL2 USP53 DOK6 FCHSD2 CHODL ACTN2 PRICKLE2 RNF165 FXR1 DNAH2 ELOVL2-AS1 RGS5 CLSTN1 EPHA3 LRRTM4 LYN MYO3A MTA3 DSCAM NBPF10 GTF2IRD2 FAM27C EPHA6 CA10 WDR72 NLGN1 HELLS DNAH9 SHROOM3 SNRPN TNFRSF10B KCNIP4 SOX2-OT CAMK1D DYNC1I1 FAM210A PRMT3 PCDH9 CST13P OFCC1 CSNK1G3 SMG6 COBL MDN1 CIT CLEC16A HERC4 TCF7L2 FRMD6-AS2 PLEKHM2 LINC00702 RELN HS3ST4 LINC00871 DPP10 APP GTF2IP2 MAMLD1 CCDC88A MIR663B ADAM12 PAK1 DEC1 TMCC3 HCN1 FRY DKK2 UTRN GPC5 PPP3CA SPIRE2 WDR59 LINC00656 RASGRF1 RIN2 UBR3 OTUD4 SCP2 RNA5-8SP2 ZNF331 LINC01090 DEPDC5 KIAA0355 MEG8 NEBL NAALADL2 AP3B1 CPAMD8 STON1 TIAM1 FOCAD SORCS2 PRIM2 CHAF1A PHF21B GUSBP1 TDRD10 PIEZO2 SNTG2 NTM NSD1 PRMT8 LRBA DISC1FP1 KANK3 GALNT2 RNF150 USP18 PCDH15 GABRG3 ZNF790 FAM107B ERC2 POR WWOX PTPN9 TTC27 KCNIP1 HUNK GREB1L TNRC6B NME9 GSG1L INPP5F SHC2 SOGA1 ADGB NXN NAMPT HDAC4 CTNNBIP1 ATP9A POTEF ADORA2A-AS1 CASC15 PPFIA2 AKAP6 SORCS3 LINC00299 OPCML LINGO2 DIAPH2-AS1 MGAT4C PDE7A GALNT14 NPLOC4 LINC00466 RBFOX2 GRM7 IMMP2L SSPN ELAVL1 DLC1 ABCC1 GABBR2 ATP8A2 SCMH1 LOXHD1 AUTS2 STON1-GTF2A1L PLAUR TMEM56 TMC7 FBXL13 COX10-AS1 EFCAB11 WLS ANKS1A RYR3 ZPLD1 SOS1 CTNND2 DNAH14 FRMD3 DCK EPN2-AS1 CROCC ASCC3 ACYP2 PTPN11 PHF3 ZNF141 ALK REEP3 LDLRAD4 CADM2 DCLK1 CDKN2B-AS1 TENM2 SYCP1 PSMG2 KCNB2 RALGAPA2 FAF1 SGSM1 PSMB2 SNRPD1 HS6ST2 TFAP2D CYP7B1 DIO2-AS1 SLC44A1 FAM220A AGPAT4 PDS5B SNUPN FAM155A LINC00559 PTPRT MANEAL GLI3 ILF3 RXFP1 CTNNA3 SPATS2 ABHD17C SNTB2 LINC00922 NFAM1 ATF7IP GMEB2 LINC00632 KCNK2 ASXL3 DENND5A RNF220 TNC DLG2 PPP1R12B MGLL NFASC LINC01141 MAP2K5 PLCB1 CPEB2 TMEM56-RWDD3 BPTF NPEPPS DNMT1 ZNF283 MAGI2 NLRP2 FLT3 PLCL1 HPCAL1 EDA DNAJC2 PRKD1 TNFAIP8 ABAT ADK CELSR1 LINC00271 TVP23C-CDRT4 GTDC1 RYR2 NPSR1 ACIN1 EYS VCL ROBO2 AKAP13 UNC13C LINC00378 CCDC171 EYA4 HIVEP3 SEMA5A BCL11A DCC ETV6 CASK DNAH5 KHDC1 C3orf67*
rsRNA	1158	*AL157886.1 AC010261.2 FAM153A AP002884.3 LRRC41 AC076968.2 PTPRR AC090136.3 ESYT1 CHD8 SUMO1 AC012409.2 DTNBP1 CCNB1 CYTH1 AC008946.1 AC087482.1 MAP3K3 PTPN22 BRCA1 UNC45B AC114316.1 WDR89*

genes 1584	AC092835.1 EFNB1 FP671120.1 LMNB2 AC123768.3 PDCD4 ABR CCNY AL133456.1 SSU72 FOXM1 AC034154.1 RNF4 AL078459.1 PMF1 RFX3 AC040918.1 HCRTR2 SEC14L1 CHFR AC100830.1 THADA TNXB FAXC CARD8 RBFADN AL033397.1 AC239811.1 AL121929.3 AC126614.1 KDM2A USP32 OFD1 KDM4B AC009318.1 UBAC2-AS1 AC099518.4 MRPL15 TARID PCCB DOCK11 SLC3A1 CR392039.1 HEATR5B AHR ACO2 WAPL SH3PXD2A HID1 AL445363.2 AC022523.1 PDAP1 AC131392.1 HRH2 AC079917.1 TTC13 TNRC18 IL17D DTNB AL591519.1 SVIL CASC19 AL109615.3 R3HDM2 VWCE ARHGEF26 AC133555.1 FAM177A1 COQ10A GPATCH8 FAM161A POLR3G TMEM256-PLSCR3 FBF1 UPK1B AP000553.5 TERF2 SUSD6 XPO4 AQP11 NAA60 SLC29A3 SEPHS1 C10orf53 UBE2Q2P2 CBS NUP133 XXYLT1 FOXN2 NOTCH2 ADD1 TTC28-AS1 TRPC1 USP43 NFAT5 FLI1 ALG9 AC093423.3 KMT2B ESRRB MSTO2P LINC00882 TDRD9 BSDC1 AC019211.1 PTPN4 BX248415.1 PROS1 ZNF713 UNKL AC093459.1 GTF2IRD2P1 NKX2-1 SLC11A1 MIR3648-2 MBD2 AC123912.4 PIAS1 AC068205.2 ZSWIM4 AC074117.1 DMAC2 EFR3B AP000753.2 PROC KRTAP5-11 SERPINB11 NDE1 PPARD GSR AL590440.2 NEDD8-MDP1 EIF2AK2 AL359710.1 AC132938.1 AC113189.2 NKAIN1 AP001347.1 AC007130.1 TRMT1 LPAR3 LMNB1 TELO2 AC015845.2 BRCA2 ACADM TAFA1 AC103876.1 AL928646.1 AL160269.1 AC004687.2 IFT172 PKN3 CNTD1 RTN2 AL049649.1 ZNF790-AS1 NKX2-3 ELP6 MYSM1 PADI4 CAPNS1 CLDN3 SFTA3 AC008758.5 AL512598.2 LINC02405 PTPRQ CLASP1 METTL6 BCR AL391335.1 TRAPPC11 HOXB-AS1 GTF2IRD2B ADAMTS8 NAA38 AL139383.1 LINC01501 AL096870.3 LRP8 AKR1D1 HS6ST1 IPCEF1 ARHGEF4 AC079313.2 TMEM251 LINC01608 EIF3J TMEM167A PPP1R36 ACSL1 PRDM10 SPINK4 AL732314.6 AC010970.1 C16orf72 NCL MOBP AC087071.2 CD247 NEURL1 SOX1-OT MCC RNF111 ID2 NME5 AC104389.4 RHOQ AC241377.2 CCDC85C POLH ZNF263 TPT1-AS1 CYP27C1 MED12L TM9SF3 TAF8 LRRIQ1 RPL35A OSBP2 ZCCHC2 AL592429.2 CDC23 AC116345.1 AC010148.1 NONO QRFPR ITCH AC008676.3 ITCH-IT1 FAXDC2 TET1 AL358134.1 NDUFA12 GTF2E2 KCNQ3 KCNC2 SARNP SNORD20 SHTN1 AC079465.1 AC034114.2 RPAP2 TMEM131L KDM2B GNB1 AGAP13P RGPD3 AL133500.1 UST DMWD AL662884.2 ERICH1 AL022068.1 MIR2052HG TFCP2 PIGU AC079949.4 TYW1 HIBADH SUCLG2 FOXF2 ACTN1 NBPF14 AC011444.1 POU3F3 PDE1B KCNMB4 AC005532.1 PTCSC2 MAP1S NOXRED1 AP001781.2 AC020779.2 PPM1N NFU1 PRCC CCDC126 BRAF LINC02653 AC091167.7 KTN1 AC008629.1 AC011447.3 SNHG29 AC006581.2 AC073896.5 RFX2 AC012368.1 AC125613.1 DARS2 DPH1 CLCN3P1 ERBB4 DNASE1 C3orf85 HIVEP1 MIR6724-1 AL117348.2 AC004987.3 WNK2 EPB41L4A AL139254.1 KANSL2 AP001011.1 MTM1 AP1G1 NLRC4 STX18-AS1 AC093525.9 PCBP1-AS1 AC027290.2 AL138720.1 ALKBH1 KAZN-AS1 GPC5-AS2 G2E3-AS1 NEDD4 ZNF43 AL604028.1 AC005865.2 PEX14 CFAP61 FLYWCH1 DOCK8 MRPL12 CHEK1 AL441992.1 AC244517.11 ARFGEF3 LRRC75A JAKMIP2 HDGFL1 LARS LINC00701 CHD3 ZFAND4 GDI1 ZMYND8 ATP6V1G3 NDUFAF6 INTS8 EXOSC10 AC027045.1 KTN1-AS1 COLGALT2 AC113383.1 EMC3 SMC1A ATP13A4 MYO10 Z82190.1 SNX10 MIR3681HG AC058822.1 AC009403.2 AL358944.1 DNAJC7 CLEC4C BHLHE22 MROH3P LEPR SRP68 AC084879.1 AC092647.5 TSPYL2 SLC25A13 BHMT2 CBWD4P AKAP10 AC068633.1 AC009139.2 SENP6 SRBD1 RFX1 AL390334.1 TMEM209 AL590867.1 CLUL1 ZNF875 FP671120.5 STAT3 VPS9D1 AC136431.1 AC005480.1 DENND4A TSPAN16 TBC1D20 OXR1 HK1 AC007846.2 RBX1 RFX3-AS1 SENCR CENPA IPMK LINC02609 THEGL CCDC127 DENND5B ATP5MC2 ALDH7A1 CASP7 C8orf34 NPIPB3 JAZF1 MTUS1 AC009119.2 GABRA6 AC025279.1 MIR193BHG ATL3 AL132857.1 ALG9-IT1 AC011491.2 WDFY1 PPP1R3A AC018730.1 AC005993.1 AC119868.2 GLRA3 AC023282.1 AC087762.1 KANSL1L AC138819.1 C1QL4 PMF1-BGLAP AC068282.1 ZP3 TMEM202-AS1 CUL1 RHEX XPR1 AC007402.1 AL137230.2 AC005520.1 C1QTNF4 INSYN1-AS1 R3HCC1L PTPN1 AC090983.2 PLCG2 TERB2 AC036108.1 AL590764.1 IGSF10 PCDH11Y FRMD8 LGMN ZNF25-DT AC011472.2 NUCB2 SNX27 MICOS10 NFIX SEPSECS BCORL1 RREB1 COL28A1 TFDP1 GTF2F1 AADACL2-AS1 SRRM3 AC004224.2 PPME1 HCN2 LARGE1 AC004943.2 PUS7 TUBB6 SPEM2 AC000372.1 DDX6 CEP76 COG5 SIL1 IPO7 AC007277.1 PCM1 TSG101 PIGN ABCA17P TMCC1 FAM172A ANK1 PPP4R3A LRRC37A3 LINC01044 AC015660.2 PC UBE4B HIF1A RF00002 FEZF2 OTUD5 AC116407.1 AL355306.2 GOLGA2P10 TNRC6C AC016721.1 RHBDF2 CEBPA WDR76 BAZ1A LRRN3 ZFAND3 OTUD7B AL162727.2 DISP2 DOCK5 ABCA9-AS1 FBXO4 COPB1 CDC123 AC073167.1 AC010343.3 AC009070.1 AMOTL1 RAB11A CDV3 ZFR AL353743.1 MTRF1 ZFHX3 FZD6 AC020916.1 TBCK RNF130 ST13 AC006974.1 QRSL1 GTF2IRD1 AC093151.8 SATL1 UBE2V2 AC011632.1 AP003680.1 TBCD WNT5B UHRF1 APLF MCF2L2 WNT9B ADGRV1 PPA1 OR51B5 MEX3C ANKRD55 STAG3L4 AC068631.1 EPB41L5 AL512452.1 SCG5 USP44 STUM GOLGA4 AP001596.1 RPS6KC1 FP236383.11 TMED4 AC139530.3 SLC27A5 C11orf54 IL7 LINC01568 SLC16A10 LINC02650 RCC2P3 PUM1 CNOT6L HOXB-AS3 AC005255.1 PIGX SLC52A1 SSC4D GNAL NKX2-5 ZNF131 AC090114.3 SEC1P FP236383.10 SLC16A14 FGFR1 UNC5A MIR4435-2HG AL162253.2 OAZ2 AC007389.1 AC034268.2 RHOT1 ARHGAP21 BSN AC253572.1 GLT8D2 MCUB OGDH DLX2 GOLGA3 LINC01986 SLCO2A1 IQCM ADGRE4P DGLUCY LAMP3 SLC8B1 AC024563.1 HNRNPR AC092666.1 LINC02558 ADAM19 DAO EDNRA PDCL2 PAQR9-AS1 FNDC8 PIP4K2B HHLA2 RYR1 PPEF2 AC025287.1 ATAD3B WNT3 LIN52 MYOCOS ARIH1 POLDIP3 RGPD5 AC017074.1 CNOT6 AC007091.1 SPANXA2-OT1 AC012413.1 TLE2 FXN AAMDC MIR3648-1 STMP1 AC016822.1 FBXO36 PPP1R12A RGSL1 AC008738.5 AC013716.1 GABBR1 USP4 CLTCL1 LSM3 VIT C6orf58 C14orf178 KCTD10 YWHAH CYHR1 SIRT2 LRRC2 NEDD4L HDGFL3 DSTYK DNAJC16 BRSK1 DSE SPAG9 AC104574.2 PANK1 LINC01586 EPS15 AL138733.1 AL049548.1 BMP7 AC010867.1 TRAPPC5 ATXN7 AL451048.1 REPS2 FCAR IPO5 TOX AL138752.2 GIGYF2 LINC01339 HIF1A-AS2 PIM3 DMXL1 GPRC5D-AS1 LINC02240 FAM53A DLEU2 MIR6724-3 PDE3A DMGDH SELENOS TEX14 TXNL4A TRIM14 AC090579.1 ZXDC AC008892.1 SLC6A16 AD000090.1 AC021242.2 ADRA1D NAA15 AL132796.2 AL138740.1

	TMEFF2 TNKS2-AS1 GTSE1 LINC01205 BX571818.1 LINC00672 LINC02144 POU2F2 AL137856.1 FUT8 WWP1 KIAA0556 CDCA5 HTR3D CREM CWC25 PLXND1 SMAP1 RERE P2RY14 LINC02138 HDAC2-AS2 IFT74 PWRN4 HERC1 NPAS2 CDC27 GFPT1 NAALADL2-AS2 GAREM1 EIF2B4 DMAC2L AC073332.1 AP000553.1 SLC2A14 SASH1 AC016576.1 AP000331.1 LINC01566 TBCE LIAS MAPK1 HSD17B3 TNRC6A KAT14 AL137191.1 AC021351.1 AP001025.1 CMC2 APOH WDR87 AC068286.1 BX005040.4 IARS CD74 ZFHX2-AS1 ESS2 HDAC8 OSBPL1A AC025211.1 PID1 TUBA1C MAP2K7 TMBIM6 CAPS2 XPNPEP3 EHMT1 ZNF616 FZD8 ARSD AC009435.1 OSBPL9 ARFGAP3 AL445242.1 RC3H2 GJC2 ZNF143 STS AC109454.3 DCAF10 AC093865.1 AC114485.1 STK11 SETD1A C16orf87 AC093884.1 MCUR1 SLFNL1-AS1 PALD1 KCNN2 ICE2 ARRDC5 AC016044.1 TEX28P2 LYZ AC008403.4 MEI1 AL365232.1 C12orf56 TRAK1 AC068473.1 PREPL RABGAP1L AC083870.1 MDP1 ZNF620 AC009055.1 SIRT1 SP8 WRAP53 LINC02453 SLCO5A1 MIR663AHG AFTPH ASMT LINC02351 C16orf97 AL357873.1 IDE AL034430.1 GPR68 AL162425.1 AC105036.3 AKR1A1 ERRFI1 GALNT1 RBM25 FBXO28 RBM15-AS1 AC096588.1 SULT2B1 MTCH2 SEPT6 CDA ZFP42 AC093523.1 CRYBG1 Z82190.2 MIR6724-2 SF3B3 RANBP9 HSPA12A AL022318.1 NKIRAS2 PPP1R7 PDLIM5 BMP8B CYYR1-AS1 EYA2 PGR-AS1 CERS4 FO393400.1 FAM50A MLXIP AC087289.4 MTFR1 AC015522.1 AC006517.2 AL445433.2 KRT18P55 AL117190.1 AC083837.1 CHMP4A C2orf73 CDK5RAP2 NUP210L AC019117.3 C6orf223 AL354696.2 AC108047.1 AIDA AP003037.1 CRYM LINC00492 LINC01592 ADAM5 LRRC8C AC012363.1 SNRNP70 AC010618.4 LINC01117 AL031686.1 LINC01934 AC010173.1 AC021087.3 KIAA1958 JADE3 GTF2IRD1P1 ATP8B2 RFTN1 SSBP4 AL049765.1 SLC2A1-AS1 AC007780.1 FOXC1 BTRC SCIN IGSF1 AC013717.1 FAM72B MYEF2 MEF2A ASF1B ADAMTS9 DHRSX UFL1-AS1 RAI14 HOXB3 AC009899.1 ARHGEF2 AC011468.2 EEFSEC ITSN2 AC098828.3 DTX1 ASB3 AC068286.2 TEX13D OGG1 ZNF410 AC068025.2 AC068896.1 IL17RE CPNE2 HECTD4 LINC02657 TAMM41 LEFTY1 YY1 SLBP FAM149A USP13 AC027045.2 FBXW4 AL445584.2 AC138123.1 AC011455.6 UBE2M INKA2 FP236383.7 AC012358.3 BLM AC020718.1 PPP1R3B PHKA1 MORN3 B4GALT2 LINC01160 PDPK1 DENND4B RAB11FIP3 LINC00278 PLEKHA7 AC025283.3 IQCG TULP4 AL078590.2 SLC12A9 SELENOM PDCD2L TPRN TP53BP1 FP236383.1 AC083800.1 PKD1P6 AC114489.1 C16orf96 AC068205.1 AGAP2 RSBN1L AP000459.2 AC024230.1 AC023055.1 AC010605.1 AL032819.1 HSP90AA1 ZFR2 TRAPPC13 SLC4A10 PHLPP1 AC092666.2 CEP57L1 RILPL2 AC012158.1 GAPVD1 ID2-AS1 HPD CCDC36 EPB41 NUP98 LINC00309 BRAP AL355315.1 PDE2A AC009412.1 DENND2C AC098936.1 ZNF428 SP100 KNL1 CD80 WIPF2 AL358113.1 SORBS1 LINC01524 AC092574.2 TSEN2 AP001273.2 MIR4300HG C17orf64 MAP3K2 ANKRD13C KMT5B HES6 RAB3B POLE2 SRPK2 CACNA1A GRIN2D SPG21 LINC02488 TSPOAP1-AS1 LINC00491 AC026341.1 TRPM1 TMEM245 NOTCH2NLA AP4S1 KLHL22 RBM47 AC022784.1 CHIC2 PCDH10 FOXF1 KIF18BP1 BCAS1 WDHD1 PAPSS1 TRAF1 NBPF26 SLFN13 NIPAL3 LINC01697 AC011287.1 STX17-AS1 AC007785.3 GLRX5 AC090360.1 SALL4 KLHL29 AC063944.1 OPRM1 ACER2 AL663023.1 AF241726.2 AC016766.1 GBF1 AC012485.1 AC107373.1 AL590627.2 AL360015.1 IFT81 LRRFIP2 INTS4 FP671120.2 AC110995.1 RNF121 MTSS1 AC121161.2 WFDC3 PDS5A DENND4C KIAA0319 NEDD9 MORN4 AC018680.1 LAPTM5 P2RX4 PPP2R3C AL360169.2 FAM114A1 RNF149 MIR6724-4 AC097462.3 RHBDD3 BMPR1B EEF1AKMT2 LRRC28 UBP1 ZNF282 NAA25 GIPC1 ZNF30 AL136295.1 RNF157 LINC01811 ZNFX1 AL022721.1 C3orf67-AS1 TTPA CLNS1A AC067751.1 APBB1IP STRN UBE2D2 AC090559.1 RASSF6 LRRC37A6P AL049869.2 AC119396.1 AC005355.1 TEX101 FDPS GCNT1 NANS GGA2 PRKCA AL157702.2 EVX2 AC004448.2 LINC02208 PLSCR3 LINC01075 AC084734.1
4920 4494	*DDC8 USP17L28 CD44 SAMD4A TRAF3IP2-AS1 RNU7-66P LRRC37A5P DPY30 KCNMA1 MIR4677 PAX7 C10orf90 CMTR2 RALYL ZHX3 C15orf27 APBB2 C10orf25 BBOX1 KLHL13 REXO1L12P GLT1D1 VPS4A ZNF799 FRG2 GGT3P NCF4 CST2P1 MTHFD2L RNU6-498P LCORL MIR4461 LDB3 ALDOAP2 BSN-AS2 FGFR1OP2P1 TMC4 DLEU7 SCAF8 EP300 NIFK-AS1 RDH10 GPSM2 CPXM2 RTN1 OR51A8P KLHL14 LRRC71 KCNA6 MIR3156-3 C4orf22 BCRP6 CDK14 ERG PARN CHD9 UBE2CP4 MSRB3 XK PDE1C SEMA4D MREG AK7 INIP NINL WSCD1 RNF185 SLC12A8 TNS3 NR4A2 PIR IGSF3 RRP7A ZFYVE1 MED13L CXCL13 KIAA1199 SORL1 NFIA TEAD1 CTAGE5 SNORA46 SLC22A2 LINC00698 RPS6KA5 SUGCT ATXN10 POTED RNA5SP359 DHRS11 CCDC34 BMS1P11 KIAA1210 TAOK3 ZNF510 GADL1 PRKCB EIF4G3 ACSM3 A2M BAZ1B SLCO2B1 RNU1-77P TEK OR5D3P PHF20 FOXK2 CPNE4 GRK5 SLC8A1 DGUOK FGGY RNU6-749P SRRM4 FAM73A ARHGEF26-AS1 OR8J3 ANKRD6 PYY KCNC1 STARD5 GPR55 GBP7 SLFN12 RSU1P2 STAU2 OR9L1P PCDHA13 SIAH3 LINC01121 C2CD3 CD38 GML ZNF257 LINC00159 IGHV3-25 RPL26P9 GPR78 GSTA2 ISM1 RNU6-132P ANKRD20A3 CORO2A OR7K1P ARHGAP42P4 ANGPT4 MIR4499 FBXO31 FRS2 C12orf40 AMY1B AP4B1-AS1 CDK8 HDAC7 ABCD1P3 FAM19A4 PIWIL3 CHST15 FPGT CDK1 NLGN1-AS1 N4BP2L2 ZNF232 A2ML1-AS1 TRAPPC9 RPS15AP6 BANF2 FAM90A6P KIAA0100 C8orf44-SGK3 FNDC1 MAP3K13 CLUHP4 LCMT1 LINC00924 SSXP1 SAA2-SAA4 BCL2 MMP16 MIR654 ZNF239 LINC01047 SAMSN1 SPANXN4 OR7E25P KIAA1324 OR4M2 PLCL2 BDKRB1 C4orf29 FAM90A3P COL18A1 PXDN GXYLT1P1 RNU6-436P TOMM34 MIR1200 RAC1P5 ADAM32 RSRC1 TOX3 TF CHST11 THRB PWP2 DNAL4 FGF7P2 SLC9A9 ANHX ZSCAN5C CDC42BPA F7 CNOT1 ADAM23 B3GALT5 RAG1 SAMM50 CDC42EP3 OR10J6P MIR1185-1 RN7SL52P RHOH ATR IGLV3-25 ADAM20P1 PAQR8 FSIP1 HERC2P8 MIR4768 AKR1B15 GRIK2 SNX25 IGSF11 RNA5SP284 GNRHR SUZ12P DPYD SDC2 MAPK10 SARS QKI GAGE2B LAMB4 MAN2B1 OTX2-AS1 PRDX4 AGT DAPP1 CHRM1 HMGB3P24 FUT4 OR4A42P CD53 DBH COX10 FAM46D OR5G1P CENPU LINC00162 GPATCH2 TMEM11 MCOLN2 ZNF227 RPL7P55 KCNQ5 CDH8 PLCXD2 LINC00851 KRT222 UBE2L6 IPO13 HEPHL1 SUV420H1 GMFB ARHGAP15 LTBR CCR3 GNB4 PRR5 PCSK6 SPRR2B IGLV2-23 SALL4P7 RNY4P7 PALM2-AKAP2 AHI1 LINC00486 BDH2P1 UBE2W ACTR5 SCN11A*

HK3 LPIN3 LCA5L ACTG1P1 LRRC53 DLEC1 ATP10A FAM225B RTCB SORBS2 C7orf60 ACAD11 PCMTD1P3 NEGR1-IT1 SKAP2 SCHIP1 RNU7-177P IGLV3-7 SYTL5 PIGL GDI2P2 C1orf112 HUS1 KIF19 DYRK4 GOT2 ZNF667 CYCSP34 GRIK1-AS1 TRAV8-7 MIR4789 PLSCR4 PPARGC1A KCNJ6 STRIP1 MSNP1 KCNK10 CASP5 ZNF709 FSTL5 RNF182 ETFA AHRR BRD4 UPP2 NDUFA13 MIA2 ZDHHC11B LINC00517 MLIP-AS1 PCDHGB4 S100Z ANKRD26P3 GPX1P2 GPR158 VRK2 DNAJC9 ZNF568 ITSN1 OAS1 NDRG2 LIN28B TMEM241 GRIP1 APBA2 TTC3 DUX4L3 SIDT1-AS1 P2RX6P ELMO1-AS1 RN7SL16P C3orf22 PLK1S1 ZNF962P CMA1 MIR3118-2 PAWR TBC1D3B TLK1 UPK3B PREX2 LDLR URGCP-MRPS24 ASTN2 ZNF384 SNORD11 ELAVL2 RPN1 FAM9B TANC2 WDR16 PGM1 ZMYM5 ZNF863P DEFA1B CSMD3 DLGAP1-AS4 DUX4L15 OR11L1 FAM193A DNAJB4 TMEM189 MIR519D PECR PCDHGA10 C15orf60 GTF2IP1 EIF2S1 EDDM3B JAK2 LGR5 POLR1A TMPRSS4 TSHZ1 TM9SF4 SIRPG BICD1 LRRC16B RAB5A AKAP2 ARMC10P1 LRRC37A12P ZBTB8OS MIR17HG ABCG8 JRK H2AFZP1 PHGDH ICA1L SNX18P9 RNA5SP470 TPTE2 EMCN-IT2 MIR491 ANKRD20A5P CCDC60 OR8K3 MKLN1 DOPEY2 RAB11FIP4 SUMF1 NF1 RPL39P36 RPS17P15 PPFIBP1 USP24P1 FAM49A NEGR1 MAP4K3 PCDHGA5 IL20RA MIR4439 IGLL5 SNORA75 OR9I3P FPGT-TNNI3K SAFB2 NCK2 FMO9P DNAJC25 CD93 GEMIN8P3 CCDC144A OR52T1P FKBP5 ERCC8 OR11H12 WIF1 IGHVIII-47-1 STRN3 CMKLR1 TMOD2 PGBD4P7 LINC00575 SCNN1B SLC44A5 PLCB1-IT1 SDK2 DICER1-AS1 C18orf64 CCT6B KRT74 SPINT4 CLIC5 PCDHA12 FBXL5 RNU6-1241P SNX18P25 ZNF677 ZNF536 CSRP2BP CD72 KAT5 MAP6 SDR39U1 MAS1 OR2L13 PARVB PPP6R2 SETBP1 CDH11 ZBTB41 CDK2AP2P1 ZBTB7C ITGA2 MME FAM213A GRIN2A MAST4 RNU1-142P WDR26 NRXN1 RNU6-723P SNORD116-27 FAM230C YJEFN3 DSCAM-IT1 MBL1P SPTLC3 DTWD2 ULBP1 AGBL1 RNU4ATAC8P PARK2 LINC00226 MAPK8IP2 DEFT1P2 FLJ00273 DPY19L2P2 IGHVIII-2-1 C21orf2 MAGI1 NIPA2 LAMA3 RNU6-554P SLC14A2 BZRAP1-AS1 RNU7-87P GSK3B PDXP KIF9-AS1 KIR2DL1 PCDHGA4 FOXO1 ADAMTS17 PCDH9-AS2 C9orf135 GBP4 WDR82 MAPK1IP1L PKP2 MTND6P3 SPATA16 AMPD3 NPHP3 ADCYAP1R1 SFXN3 SNORA71 ST6GALNAC3 PDE6G LRRFIP1 TUBAP SERPINA4 MTCO3P2 RERGL TBPL2 IL18RAP RNU6-164P TRIM16 TNR-IT1 DPP6 SETD3 MIR539 PQLC3 MIR4713 PRELID2 ACOX3 CD109 GOLGA8J GRID1 CR1 SLC22A3 LINC00161 SNORD116-17 C2orf48 ZNF571-AS1 SLCO1C1 PLXNA4 PTGER4P2 GRIN3A RN7SL678P DEPDC1 GRAMD1C LINC00504 ANAPC1P1 SMOC2 TNRC18P3 ZDHHC17 KCNH1 IGHVIII-26-1 HORMAD2 HLCS MCF2L STK24 ACSS3 LINC00604 CNBD1 ABCD3 DCDC1 RHOQP1 VPS26B PARVG CACNA1C FEM1AP4 PDCD6 CCSER2 AMPH BRI3BP BACH1-AS1 LMO7 RNU6-768P NUTF2 KRTAP10-5 TPT1P2 MAN1A1 U8 FOXP2 PCED1B MIR524 NLGN4X SNORD109A RNU6-1021P IGHV4-28 FSD2 EIF4E EXOC4 LINC01162 ATP5A1 SPRR2C HEATR5A BPIFB1 HTR4 PPP1R26P3 URGCP EMB TRAF6 AMY1C CKMT1B PNPLA3 C14orf183 LINC00320 FAM19A5 ANO4 C12orf55 RNU6-1003P KCNQ4 GIPC2 PIK3C2B BBS9 C5orf64 PA2G4P3 FAM184B ERCC4 FAM83B OR4K15 ST7L CDC27P2 LINC00595 C15orf26 RNASEH2C COMMD1 SCAMP4 PFKFB2 HNRNPA1P61 ENTHD1 RNU6-917P WDR33 RN7SKP233 OR5K4 PCMTD1P2 RN7SL92P PAK7 CHD7 WDFY3 RNF126 RXFP4 C5orf51 ANKH C19orf18 MOV10L1 BIRC6 ADHFE1 SH3PXD2B PTBP3 IGKC ANKRD20A14P AK8 NLK PCAT2 BCRP7 TDRD3 NIPA2P2 MIR544A IPPK OVCH1-AS1 NBEA POTEC FAM19A2 CDHR4 OR5L2 GBE1 ANKRD30BP2 ZNF285 SNX5 AFAP1 NCAM2 OC90 DPY19L2P1 CYSLTR1 PAPD7 MIR154 SNORD115-35 GPR161 TIAM2 OR51B2 SLC20A1P3 NR4A1 ZNF10 TIGD4 SLX1B-SULT1A4 TRAK2 CEACAM5 C14orf37 MYT1L TMPRSS3 ZNF878 SRGAP2B UPB1 GIMAP4 CLDN14 KCNA3 IQCJ-SCHIP1 SCARNA15 CARM1P1 ZHX2 KIR2DP1 LRRC49 C1orf177 LINC00273 SMARCA4 SOHLH2 HERC2P3 RNF152 TAGLN3 FAHD2A CNTN1 NFE2L3 TTLL9 PALMD MEOX2 EML4 STK31 ENPP7 TTC39B KCNG3 TMC5 DUX4L12 KIAA1257 LINC00365 HIGD1AP13 PLEKHB2 OR4K6P TMEM50B KRTAP13-5P SNORA1 ANKRD36 MTATP8P1 ARHGAP29 OR4S2 GOLGA8K RTCA TFP1 NRG3 APOOP1 TMEM52B CAMSAP2 LINC00970 RNU6-141P ABL1 ZNF433 RPL7L1P12 ABCA11P PWAR6 LINC01094 DUSP27 MC2R TBX3 OR8K4P NANOGNBP2 CHRFAM7A BTBD11 MIR620 ELMO1 RPL13AP2 PAAF1 SLC5A10 CABIN1 TRIOBP A2ML1-AS2 TUBA3FP ABCD1P4 HRH1 MRPS31P5 CHEK2 FBXO47 WDR27 CYP4F33P OIT3 ZBBX PKHD1L1 SLC9B1P4 HTRA1 ZNF585A IFT88 SIGLEC30P GUSBP11 RAB22A ARHGAP5 DDX21 HSPE1P19 ZNF160 ANKRD20A9P ZNF688 KIAA1731 PWP1 ILDR1 GAS2 GRIK3 GRXCR1 CACNB2 PDE10A NUMB TNIP1 PAN3 STXBP4 FSIP2 TOX2 PSG10P MED15 ZBED4 ZNF720 NABP1 MT1HL1 LIN9 MTPN RHOJ ZER1 CLUHP5 MRPS31P4 BRE FAM9A ZNF474 SEC14L2 OR52X1P OGFOD1 ZBTB8B BACH1-IT2 PHEX SOX6 CIRH1A CCZ1 POTEM FAM182A POLE SYBU TSPEAR LINC01035 FILIP1L TTC4P1 COL4A6 CATSPERB ZNF559 ZNF559-ZNF177 TRPM3 DET1 ZMYM4 STK32B TACR3 LINC01020 VCAN ACTBP8 BRMS1L POTEB2 DNAH8 IGLC2 CNTNAP5 SLC1A3 MIR3173 RPS3P6 PCDHGA11 RGS12 C14orf119 DNAJA1P1 MIR670HG RNA5SP438 ASNSP5 SHANK2 LRCH1 CHP1 SAA4 RAPGEF5 MIR3666 TCL1B RN7SKP218 RPA2P1 UBE2E2 CDCA2 DTD2 IGHVIV-44-1 PDIA6 PTGFRN ZMYM2 RNU7-119P TAB3 SNORA76 NCK1 KCTD8 SDIM1 CHCHD6 RNU6-631P SPIN3 SH2D4B CPB2-AS1 UNC5D CD8B HS3ST2 MRPS21 ZNF830 EVA1A MAGEA11 ZNF567 SH3BP1 ERMN MIR605 SIM2 CHCHD2P9 GABRA5 TRIM48 CCNB1IP1P2 KIRREL GPC3 PDGFRA DOK5 TLK2P1 PIGUP1 PTPRJ NANOGP4 C22orf39 RNU4-56P STAMBPL1 ADCY2 ZRANB2-AS2 ZNF737 CRYL1 OTC AGMO MRPS6 NOVA1 HADHA IGHV3-64 DLG5 PER1 RNU6-46P ANKRD20A4 MIR3118-3 MYH4 WFDC10B ADAMTS9-AS2 ASTN1 MIR1324 AIFM3 SH3BGR PCDHGB3 NF1P8 CENPK ATRNL1 RN7SL714P KIR3DL3 DTX2P1 SUN3 SLC27A2 TMEM135 DUX4L2 PRDX2P3 WARS2 RPL23AP60 CPE C16orf3 RNA5SP385 TUBGCP5 LINC00587 UBE2V1 RFFL MBOAT7 IGLJ2 POMC TRDV1 RPL21P41 KIAA1462 NLRP12 MIR4681 DNM1P32 TLK2 ZNF2 SH3GL2 ABCC13 COL24A1 SNX18P8 MIR759 GAK ATP5F1P6 C10orf113 PSPC1 ARHGAP24 ALMS1 TERF1 SNORA16B COL11A1 VN1R91P

SLC13A3 N4BP2L1 C11orf65 LINC00907 IGHV1OR15-9 PITPNC1 SLIT2 TPTE2P6 MYLK3 ANKRD18A C2orf88 CACNG6 CEP128 MCM3AP-AS1 LRIT3 ZSWIM3 ZNF804A IGHVII-67-1 WDR83 COMMD6 SNORA67 MIR1254-2 NARS2 WRB OR4N3P TTC4 ZNF734P C15orf54 XKR4 OR10AK1P LPPR5 PTGER4P3 PCOLCE2 POTEB CDC20B IGLVI-68 LINC01143 LINC00347 SLC26A7 GPC5-IT1 C9orf3 KIAA1432 ROR1 SYNM RAPGEF4-AS1 MAPK4 GLP2R MAP2K3 OMG SLC4A4 ZNF486 RN7SL83P ADAMTSL1 KRR1P1 ZNF675 EZH2P1 CSNK2A1 NRG3-AS1 VN2R17P EXOSC3P1 OR4F16 RNA5SP353 MRPS18B AOX1 IGKV1-5 USP17L27 DTNA SERPINA5 PMS2P9 AKT3 CRB1 ANKRD34C ADAM2 NDEL1 RBAK KMT2C LAPTM4B LINC00472 MT2P1 DIO3OS IFNWP4 TRIM5 KCNS3 ARHGAP11B RPL23AP82 PCDHGA3 THEMIS VWA3B SLC5A8 FLVCR2 SDHA CYP4B1 COL12A1 STEAP2-AS1 MEDAG MIR4529 RNU6-810P MTND2P25 ABLIM1 ZNF280D GAGE2C FAM230B RHBDD1 OR8K2P TTC26 ZNF22 IGHV3-76 PSD3 CHRNA7 HOMER2P2 HECW1 HMGB1 MEF2C FAM174B MAST4-AS1 PSPH AFF3 COG7 IGHVII-31-1 IGHVII-22-1 KLF3 BAIAP2L1 EFTUD1P1 RN7SL130P SDAD1P4 IGKJ5 LINC01013 UGCG GPHN SLC6A2 C1orf106 PTN ORC3 VWFP1 ATRX DMRT1 TVP23C SLC9B1P3 ELP4 RYK BID IER2 SNORD115-48 LINC00839 TGFB2 CHRDL1 TRAF3IP2 HSPB8 MNAT1 PXT1 ATP6V0D1 RLBP1 CD300C NPM1P41 LZTR1 TRIO ZNF385D IQCK C8A TEX36 CAPN3 SLC15A5 CTNNBL1 CCDC12 IGLVIV-64 REXO1L10P KCNE1 PSMC1P13 ITLN1 TRMT61B RAD51B DGCR9 ZNF248 PTPRE RNA5SP487 PTK2B FAM20A ZSCAN5A LINC00645 RNU6-321P TSPAN3 FBXW8 RALGAPA1P MYO3B DUSP22 CALB1 LINC00866 SLX1B KRT223P SH3RF3 FAM95B1 C18orf8 ZNF347 TPP2 GTF2E1 STRADB ST6GALNAC5 KIAA2022 MYOM1 KCNIP3 PSG8 EXT2 RGS16 IL21-AS1 URB1 ZSCAN30 TUBAL3 RNU6-58P CRYZL1 MECP2 PSMA1 DIP2C ZNF454 MAP3K5 BCO2 CLDN11 BMPR1A TIMP3 MIR4742 RNA5SP50 RPL23AP12 ABCG1 SPAG16 ZNF299P DSCAML1 ABHD4 FAM207CP DUXAP8 PLN DUX4L8 PRDM9 FBXO27 YOD1 EML1 SMAD3 CTDSPL2 C14orf182 TBC1D12 PABPC1P12 DGKH CDK12 USP17L6P RERG SLC16A7 HTR2C RNU6-196P DCBLD2 PRKAR2A FAM90A13P CACNA1E SYT10 THYN1 KATNAL1 NF1P2 MAGI2-IT1 MYO6 EZH2 RBM22P2 MUC16 AKIRIN2 MRPL33 VCAN-AS1 HIGD1AP2 KIFAP3 GLRA2 PHKA2-AS1 PON1 BORA PI4KA SOD3 VWA8 DDAH1 ALDH4A1 SAMD13 RNF17 DHRS7C OR51B8P PCDHGA7 RNU2-5P RNU1-139P TMEM161BP1 PRAMEF26 TNFRSF11B FAM102B CD36 MIR3116-2 PIBF1 ARF4 LINC00448 SNORD115-20 MIR3667 TMPRSS11E TNFSF15 RN7SKP162 NCF2 GABRR2 PIK3C3 BNIP3P3 CAPN14 RNA5SP453 FAM90A5P USP16 SLC9C1 KRTAP20-2 SNORA40 GPCPD1 BCL9 UBE2CP2 IGHV4OR15-8 C9orf41 TRAF3 RIPPLY3 PLXNB1 C9orf171 MPPED1 ERICH1-AS1 SNAP25-AS1 MIRLET7C CHD6 HMCN1 DPYD-AS1 PWRN2 FGD4 LY86 CYCSP51 MTND1P12 CDC27P1 LRRC4 SPICE1 N4BP2 ETS2 IFFO2 IGFBP7 NDNL2 FCRL3 POTEE CTBP2P4 EPHA5 NMNAT1P1 ERMP1 FAM24B MACROD2-IT1 OVGP1 FAM27B ZNF721 VN1R7P SGIP1 B4GALT4-AS1 HIRA MERTK ARMC8 RN7SKP253 RN7SL801P SNORD115-36 SLC47A1 AEBP2 OR5BL1P RNF144A USP17L30 SLC7A14 C18orf63 RN7SL864P TP73 C7orf76 RBMS2 TMPRSS6 SERPINA1 ANTXR2 LINC00376 RN7SKP76 TMEM74 RBM19 TAPBP IL1RL1 ENAH ZNF765 KIR3DL2 GPR64 FEM1AP1 C3orf33 TAC4 GABRG1 MYO16 CYP4F31P KRTAP10-1 TSHZ2 TRAV22 MAPK9 ZNF366 MTND4P12 MIR548U ZNF540 LOH12CR1 ISPD CD58 PTGFR SNX31 GRHL2 CDR2 IGHVII-30-1 SELE IGHV1OR21-1 PSMC6 PCDHA2 FAM171A1 TRAT1 MAGT1 MIR517A PCDHA11 ITGB7 CHKB SCUBE1 ARHGAP42P5 ZSCAN10 ARHGAP22-IT1 ZNF595 CNN2P12 CDH17 NLRP13 GNAT3 OXCT1 RPL5P35 MANSC4 SV2B HECW2 FAM230A NOTCH2NL ABCA8 PDE9A LINC00894 ZNF679 SLCO1B7 PEAK1 EYA1 KCNQ1OT1 MAPK14 PRR14L DYNLL2 FBXW12 LINC00669 SATB2 RNF216P1 KIAA1217 ABTB2 MORC3 MAGOH2 MIR4477A GPR126 ANKS1B OR4F14P PPHLN1 RNU6-185P SNORD115-21 WFDC10A CHL1 VANGL2 P2RX6 KRTAP19-7 HSF2BP SPOCK3 BNIP3P1 FRMD6 UNC79 IGLVVI-22-1 INTS4L2 ANK2 SPON1 RAB23 REXO1L8P ROCK1P1 FAM8A2P ZNF516 PRLH SLC1A2 INVS KRTAP12-2 STXBP5-AS1 ANO2 CST9L PCDHA9 hsa-mir-6080 LNP1 HPS3 LINS KCNK17 ARHGEF3 SNORD115 RHBDL3 SUPT3H KIAA1244 MAML3 ZNF840 GABRB2 EDNRB SLIT3 MAN1C1 CFTRP1 BAGE2 GRIN2B RCVRN NLRP7 LIN7A CYCSP27 CYCSP17 TPCN2 CNN2P4 MPZL1 DMBT1P1 ZNF578 LRRIQ3 ADAM9 TPH1 SNORA51 MARK2P5 RNU6-1266P MATN3 IFNGR2 RNASE9 EGFLAM FAM155A-IT1 UOX LUZP4 SIPA1L1 DGKB RNU6-538P TGFBR1 GBP2 WDR4 DUX4L13 RPL19P12 TTLL2 UACA LRPPRC ACVR1C HEXB RPL34P3 DPH6 ZNF209P TNKS RPS4XP22 ABI3BP DNAJC25-GNG10 TSNAX TAS2R1 YBX1P7 IGHV1OR15-6 PRRC2C TUB KLF12 ARHGEF18 KCNK1 PCDHAC2 EREG AXDND1 DCAF7 LNX1-AS2 LINC00473 WBSCR17 CDH6 PCDHGB2 NDFIP2 RBPJP7 GABRA2 RIMKLB MIR4327 KRT126P ANKRD36BP2 TRAV8-5 TNS1 MDM1 OVOL2 SSUH2 LENG8 DUX4L9 RN7SL516P CBX3P4 IGHV3-30-2 AATF CHEK2P4 ACSL5 RNU6-352P LATS2-AS1 C1orf87 PRUNE2 RN7SL449P SGMS1 WDR64 GART hsa-mir-4528 ADCY8 LINC01122 HERC2P4 PCDHGA12 TTC7A RN7SL99P GRAMD4 PSG1 RNU6-978P GJB7 ELP3 PPIAP1 ITPR2 SUN2 SNRPGP9 BRINP1 HIAT1 SEPT7P9 RNU6-552P LINC00158 FRMPD2 NPM1P31 OMA1 BCL2L13 RPL30 CCDC30 IGSF5 SULT4A1 SHC3 DRG1 MIR1299 SRP19 SIRPD TRAPPC10 KLHL25 LEMD3 KHDRBS2 GAGE2D MGMT CPQ IGLVIVOR22-1 POLQ CNOT10 DYNLRB2 SOAT1 IGHV1-3 ABHD17AP5 MAP4K5 TCAIM FOXD4L4 OASL KCTD9 MSANTD3-TMEFF1 TM4SF2 EHBP1 OSGIN2 DARC SKA1 SLC9B2 SHMT1 CRISP3 RPL36AL NEB SRSF4 GABPA SCGB2B2 POU5F1P3 CAMK2G C6orf3 HNRNPA1P7 FGF14 BRD1 KIF3B CSRNP3 MIR1290 RNU6-1184P KLHL32 CEP164P1 BCL7C RNU6-278P TMEM100 PSTPIP2 LINC00861 SNORD115-12 MIR4490 ANP32C CASC4P1 ITGB3BP UBASH3A FRG2C CACNA2D3 ZNF429 HDHD1 CUBNP1 DRD5P1 CTBP2P5 RNF213 LYPD6 PDIA5 SUCLG1 TMEM67 OR51I1 WDR47 VDR MIR4452 CLDN12 SLC25A1P2 KRTAP13-6P SPESP1 PRKCE DTD1 BTF3L4P1 ITGAM MIR4695 CCDC149 DUX4L7 SH3RF2 SIGLEC26P CNTN3 RPS3AP46 OR4A5 MGAM DUX4L10 GLIS1 RN7SKP168 PRDM11 CACNA1D CD163 DPT BTBD3 ACTR3BP2

RBM22P1 ACSS1 BBS2 PACRG IL1RAPL2 ULK4P1 PCDHA10 LANCL3 IGHVIII-22-2 SPAG17 ZDHHC11 CHCHD3 GAPDHP69 MOV10 EDIL3 RN7SL587P RPL39P40 SLC20A1P1 ZNF337-AS1 LATS2 MS4A1 MIXL1 RPS6KA2-IT1 IGHV3-16 SKAP1 TMEM51 CC2D2A DSC3 WDR11 MIR487B RNU6-909P CNR2 KLHL33 SNX29P2 GLRX3 ST13P15 CCR1 UVRAG CACNB4 PTPRN2 LINC00674 MTND1P2 SDAD1P1 LINC00395 USP6 INPP4B ZNF732 MIR655 KLF7 AHSA1 MIR941-1 DKKL1P1 GDAP2 AKR1B1P1 MGAT2 LARGE-IT1 HIVEP2 UBE2R2 RN7SL673P PYGL FAM65B TXK GRIK1 CUL5 OR9A4 NOL12 CHTF8 SYT6 C7orf66 FOXD4L2 LINC00446 NUBPL SOX5 PRAMENP KIF6 ADH5P3 GXYLT1P2 DGKI IL12RB2 RIN3 RN7SL23P REG4 USP17L29 OR52H2P UBL3 RNASE3 NLN RNU6-49P PABPC1L TBC1D30 MTND1P23 ANKFN1 NME7 MND1 GTF2A1L PABPC1P5 MIR4519 DENND1B ANKRD30BP3 SPNS2 SKOR1 BOLL HERC2P5 RNU6ATAC33P IL1R2 RPS27P27 EFHB TRAPPC8 RUSC1 KDM4C FGF14-IT1 ACSM2A DCUN1D4 SDK1 SLC9B1P2 LINC00544 CYCSP41 C5orf66 SLC1A1 ST20 DHX57 ACSM1 RNU6-1063P MIR1267 TDRD12 RNA5SP366 SNORA8 CCL8 PPP6R2P1 SLC12A1 ENTPD4 KRTAP8-3P THAP7 MAPKAPK5P1 TMCO4 CTBP2P7 SMC1B RNF144B GRM5 ZNF841 PEX3 BMS1P12 PAMR1 ATG4C RBM11 NTN1 ABCB10 LHFPL1 RPL31P40 LINC01107 DIAPH1 KRTAP20-3 GAGE12I OS9 SLC4A1AP NGLY1 U6 IGLC1 IGHV7-34-1 ARAP2 ENTPD3-AS1 LINC00937 NR5A2 LDB2 MYRFL CYP4Z2P IGF1R TATDN2P3 PPP1R26P5 SNX30 SNORD112 PGAP3 PKD1L1 C21orf62 TSNARE1 RNU6-803P STRBP TMEM260 OR11H1 BTF3P14 ARHGAP42P3 SHISA9 OR9Q1 JAM2 ZNF355P CYCSP32 MSI2 CORIN OR4K17 SNHG17 LRRC7 IGHV4-4 RNA5SP125 TEX41 LINC00458 LINC00221 PRSS23 SRD5A3-AS1 RBPMSLP CPHL1P ANKRD29 EPM2A DEFB127 PDZRN4 SGMS2 CNTNAP2 S100A7L2 SNORD45 LINC00930 SPECC1L-ADORA2A SHFM1 MIR600HG QTRTD1 LCE6A KIF18A LARP1B RN7SL743P CBLN4 LIPE-AS1 AQP4-AS1 C21orf91 NPM1P13 CFTR OR4C15 MIR96 RNU2-27P KRT18P2 FLRT2 WRN SDF2 NALCN-AS1 KMT2A RN7SL435P ZNF90P3 CCDC144NL XBP1P1 TCEB1P32 ARHGAP44 RAD51L3-RFFL EFCAB6 ZNF402P BRINP2 RNU6-56P C2CD2 ERCC6 TEX26-AS1 EFTUD1 LINC00840 RN7SKP140 DSCR3 IGKV2OR2-2 HSPE1P25 USP17L5 RPS20P5 ZDHHC14 DPP8 NIPA1 MIR4764 ZFP64 FAM194A MSRA EMCN-IT3 KIF13B SYNPR DDX51 ZNF626 MKRN3 LINC00349 SLC30A5 ITFG1 LINC01029 IMPG1 C22orf34 ARHGEF33 RN7SKP99 DSCR4 GLB1L3 NKAIN2 CYP2C8 RANBP10 FAM90A4P CD96 CAP2 RBMS3 LINC01053 DGCR6L SNORD56 FAR2P4 MATN2 TDRP TMOD1 REXO1L1P NHSL1 SNORD115-41 SRGNP1 CIZ1 ITGA11 WWTR1 GAS7 SNORD17 NFX1 ZNF607 ATP6V0D2 HTR4-IT1 ARHGAP6 MCM3AP KCTD7 RNVU1-18 ZNF98 ST3GAL3 PLCD3 MIR4307 PCDHA1 TTC6 APCDD1L-AS1 WSB2 TYW1B SETD7 ITGA9 hsa-mir-3171 CHAF1B QSER1 RN7SKP96 KIAA1328 Y_RNA RNU6-286P OFD1P13Y ADIPOR2 PCDHA8 GBA3 LINC00298 RN7SKP238 RPGR CES5A OVCH1 PABPC1 CATSPER2 TMEFF1 GPR125 CLDN8 STARD9 RN7SL766P HSD17B12 PCSK5 CTPS2 NDUFA9 LINC00537 EFCAB6-AS1 RNU1-131P NES EGLN3 HS6ST3 TBC1D2 FKSG68 CUX1 RNU3P3 MUC5AC GPM6B CLEC4A LINC01010 RN7SL456P PRR12 KCNMB2 ANK3 SLFN5 SNORD74 TIMP2 CDH12 ZMAT4 LINC00911 TECRL GTF3C6 SNX24 MORC2 OR11A1 UPP2-IT1 TMEM132B CPSF1P1 SLC25A15P5 PTPLAD2 ENPP7P10 SAMD9 FLG-AS1 SV2C HNRNPKP3 ZNF490 AKR1C3 FGFR3P5 GMDS TPTE2P3 CACNA2D4 LHFPL3 SPARC RGPD1 C14orf144 WDR52 CNIH3 DOCK3 ANKRD62P1-PARP4P3 ASB17 RNU1-33P FEZ2 THSD4 CDC5L RPL39P33 SLC9B1 EFCAB1 RPS24P16 KPNB1 LRRC37A7P TAS2R38 FOXO3 LARS2 TRIM59 NF1P3 TTLL8 GPC6 HIP1 ACTR3BP3 MRPS31P2 LINC00284 RASGRF2 ST7 MAMDC2 TRPS1 RPS17P14 MFSD9 RNU6-1193P PCNXL2 MTUS2-AS2 TMEM101 NF2 ADAMTSL3 EVA1C FBXO32 SNHG14 PCDHGA6 NXPH1 OFD1P12Y DDX3X PHF2P2 MYO18B IGLV4-69 CDH4 ARHGEF10 RNU6-410P SERPINB12 CASP12 GABRA3 E2F3 FRG2B FGD6 TFF3 MIR603 TNR LINC00687 VDAC1P1 ADCY9 OCA2 CELF4 PPP4R4 PRKCQ-AS1 CDKAL1 VAV3 RMI2 ARHGAP22 MROH1 ULK4P2 NPFFR1 NPC1 PCDHGB1 KCTD9P1 INPP5A RPL12L3 C17orf51 SMARCE1 SLC18B1 SUSD1 VRK1 MIR649 ZNF678 CNTN6 MLLT10P1 CXorf30 TMEM106B CLIC6 TRAP1 ACSM2B SMEK2 SCN9A ZNF420 CDK2AP2P2 SCARNA17 ATP2B1 ATF7IP2 MS4A14 BNIP3P2 C6 KCTD9P2 IGF2BP1 ZNRF1 LINC00470 SERHL ATRN FBLN5 HAPLN1 MIR3152 RANGAP1 RAB7A MIR551B CHKB-CPT1B C10orf11 ARNT2 KCTD1 C5orf17 RNLS SPOCK1 SPEF2 NF1P6 MIR3118-1 CXorf22 H2AFY APBA1 ANKRD20A7P RAB2A SNORD115-11 HPSE2 PLCE1 CEACAMP6 KRTAP4-12 IGHV1OR15-4 GRAP2 MUM1L1 TACC2 ATP5BP1 HNRNPA1P71 WASF1 ANKRD36B CUBN RNU6-1280P ITPK1 FAM214A DNAJA1P5 NT5DC1 CYP39A1 CUBNP3 RNU4-82P AMZ2 FRAS1 SIDT1 ATP9B CHCHD2P4 MITF ARL2BPP8 TPRG1L PEBP4 BTN2A1 ASNSP1 IGF2BP3 STK32C CACNA2D1 DHX35 LINC00520 PON3 ADCK1 RPS15AP34 G2E3 CCDC88B RN7SL143P TCL6 SAMSN1-AS1 CD2BP2 PAPOLA TOP3A RNU6-10P RAB3IP CEACAM6 CHRM5 SPECC1L TET3 ZNF208 CASP6 MAGEB3 LINC00693 KIAA1009 GCNT1P1 ZNF582-AS1 NOC4L PIGFP2 MNS1 MSANTD2P1 ZNF718 CXADR PTPN20B POLR3K FAM209A PCDHAC1 TANGO2 CCDC64 HERC2 GGT2 LRFN5 NASP PIK3R5 CTBP2P8 CDC14A PALLD TRAV24 ENOX2 AGBL4 TENM4 CPNE5 GZMAP1 CLTA DUX4L18 PARP16 A1CF ASB13 SLC6A10P CECR2 hsa-mir-490 CASC16 IPO7P2 RHOC CHRNA5 RNU6-1291P FAM63B TSPAN13 GHR SPTSSA RNU6-311P DUX4 FTH1P27 BCLAF1 MIR3118-6 LINC00374 NKD1 ARHGAP39 AKR1CL1 HTR7 KIAA1715 ZNF345 RIPK4 MYL12A CCDC57 C10orf120 TRUB2 CEP170 DNAJC5 RPS15AP3 LINC00440 FRMD5 MAGI1-IT1 MIR99A ULK4 RNU6-1132P PTK2 RNF217 RNU6-1225P DLX6-AS1 LLGL2 RBFOX1 SERPING1 KIR2DL4 STARD13 TMEM120B PCDH7 ASIP CDH10 KL LRRC4C USP17L25 ASUN ABHD2 DENND1A ALCAM MZT1 INTS4L1 PDZRN3 C1orf168 ZNF41 AVEN TMEM117 TPTE2P2 DNMT3B GUCY2C ADAMTS16 OR10T1P TASP1 NIPBL PPP2R5A POTEG FYB SLC17A3 MICU1 EBPL IGHVII-62-1 BDH2 HNRNPA3P14 ZNF114P1 CNN2P7 DUX4L6 RGL1 MIR3198-1 ATG10 MBD5 LRRK2 BACE2 NKAIN3 MIR548AX CPEB1 INO80D PCDHGA1 ZNF728 SLC25A21 DYNC1H1 PALM2 CLSTN2 LSM12 EPHA1-AS1

PWRN3 RNU6-316P PLD1 CD300A VASH1 SLC35A5 PKHD1 MTND2P28 MIR1281 TTLL11 DIDO1 RARB CCDC18 COG3 DIRC1 CBFA2T2 MARK3 DHX15 KRTAP29-1 AGGF1 DUX4L11 PRRG1 LINC00993 ITPRIPL2 ADAMTS2 MYH13 PKIB TCF4 NR2C2 MYH1 MGST1 RBM44 FRYL ZNF432 MMP27 SPG11 VN1R4 RARRES2P4 C6orf183 FUBP1 KIAA0586 AGGF1P3 PCA3 OR7E83P MMP20 FAM126B PLEKHM1P PRG3 MIR4533 GANC KRT43P MLIP PHACTR1 DDX24 ZNF583 OR5H8P ATP1A1 MIR487A LINC01058 EML5 C5orf47 PGM2L1 OR4C9P RNU4-59P STRC BCL2L15 RN7SL646P LIPI OR4H6P YIPF6 DISC1 DZANK1 RN7SL141P ALG6 PPL LRTM1 MAP3K7CL FMN1 ANKRD20A1 RALGPS1 DNM1P51 MIR485 TMEM212 PMS2P11 ANKRD62P1 TRAV6 ZFPM2 SEC63 GC SLC35F1 RPL9P28 TOR1AIP1 ADC PXN SVEP1 TNF C21orf49 LINC00113 USP17L24 PHYKPL VTI1A ASAP1 RNU4-40P PCBP3 BFSP2 LINC00971 FRMPD4 ATP8B4 HPYR1 LMCD1-AS1 COL23A1 LINC00960 TMEM179 LINC00639 DSCR10 FYN FAM3B KCND3 ITGB1 KIR3DP1 PSMC4 RIMBP2 TTC12 CSPP1 IFT43 CCDC169-SOHLH2 XRCC4 RABGEF1 DCLK2 MEMO1 GAB4 PRELID1P2 RNU6-617P LINC00408 EPHA7 OR4A9P FHIT MAP7 PPOX CRCP C7orf49 TEX9 RAD54B MIR1911 NSG2 LRR1 MIR3687 OR8A2P LINC01070 MSANTD3 LINC00842 BARD1 ZNF177 DGCR5 AGGF1P2 MCFD2 IGKV2-36 GRIA1 FAM19A1 POM121L9P ZNF627 RNU2-42P TRABD2B KRT8P15 RN7SL204P RN7SL194P C1QTNF7 SPIDR ARR3 C17orf80 ANKHD1-EIF4EBP3 UBA6-AS1 SNORD115-23 NAV2 MIR183 EFCAB5 STK3 CHN2 ZNF781 VN1R53P CNOT7 FSHR POTEKP NBEAP3 GAS8 LCE2B MARCH3 BMP15 RUNX1T1 SGK2 RNVU1-17 MSR1 SNORD115-40 LINC00380 PSIP1 NOL11 TRPM2 SPRR4 SLC30A7 SMCHD1 MED15P7 LINC00507 NRG1-IT1 S100B NET1 ESR1 GCNT2 ARHGAP12 ZNF767 SLCO1B1 DPY19L3 KSR1P1 SGCZ CILP2 CCDC176 ICT1 EDA2R SEL1L2 TNFRSF19 PLCXD3 MIR889 NAP1L6 DENND2A LUZP2 SIK2 APTX MXRA5 GRM1 SCFD1 PDE4D TRPC4 CNTN5 MTND1P31 RNA5SP519 PRKACB ZNF705A GNG2 RN7SL662P PCDH11X PCBD2 TRANK1 RIMS1 UBE2K CPB1 MIR4798 RRH BCAR3 DRP2 RNU7-176P PDZD7 ENPP2 FHL5 TRIM60P13 RPS27P16 DOCK4 KCNMB3P1 VPS53 SLC5A3 TRIM51 RUFY1 LINC00382 TMPRSS4-AS1 IGHV3-41 OR4K4P ATP6V1E1 RPS3AP41 FRMD4A ZNF965P SNORD115-38 MCTP2 CERS3 LMX1A PIGK SLC25A15P4 PCDHA7 EGFEM1P ABCA12 PLOD2 FIGN PCSK2 TMEM178A NUF2 ABT1 OR4C14P ARID2 CD86 MIR670 MARK2P8 RNA5SP300 NMD3P1 ZNF33BP1 B4GALT4 FAM228B BMS1P16 RNU6-78P LINC01043 KRTAP19-10P CNKSR2 DNM3-IT1 GFRA2 FAM104A HMGB3P20 SNX29 SNURF SAMD5 REXO1L11P RPS23P5 BEND5 ISCA1P3 COL8A1 ATXN3L EPHB1 SSBP2 BMS1P18 FAM85B COLEC12 LPHN3 REEP1 LINC00478 RPH3A LSAMP FAM90A22P MIR548F1 CHMP4C KCNQ1 WFDC9 NR2F2 CTTNBP2 BCL2A1 C2orf27B C14orf164 FHOD3 PSG7 IGHVIII-67-3 LINC01088 PARP8 ZNF215 TEAD4 RNU6-725P EFEMP1 DTX4 RNA5SP186 PIGB SRRM1 RN7SL321P AJAP1 ZNF608 IGSF21 RNU6-250P SLC38A6 SLC9B1P1 GLCCI1 SNX19P2 USP40 LNX1-AS1 RASSF8-AS1 DUSP23 MIPEP HRNR IGKJ4 ABCC9 UCHL3 KLHDC8A IPO8P1 BPIFB4 MRPL42P4 RWDD2B ARHGAP20 INSL6 DOCK1 SERPINA2P NR3C2 MIR134 SNORD115-47 CRADD MTUS2 NCOA5 EMCN RNA5SP478 VASH2 WWTR1-IT1 DIAPH3 OR8U1 TPTE2P1 PTTG1IP ADARB1 GGT1 COL9A1 ZNF664 GPC4 RN7SL568P TMEM178B HAS3 SOD1P3 OR10Z1 PAGE1 ARHGAP32 TTC40 SLC27A6 SLC7A13 TRPM7 CGNL1 FLT1 PPP2R4 REXO1L9P LINC00276 CDRT1 ANKUB1 RN7SKP6 LINC00032 VSTM2B EFNA5 NOL4 LINC00536 MIR4634 GTF2IP3 ISM1-AS1 STARP1 ENPP7P5 CNTLN C1orf173 NEK5 ASL AMY1A PCDHA5 PM20D1 BMS1P10 KLHDC7A CMSS1 PHC1 SLC22A23 LINC01049 DSCAM-AS1 PLCH1 CACNA1C-IT3 PCNXL4 PCDHGB7 C14orf39 ZNF493 TLN2 BPESC1 RNASE13 GLRA1 TRAV33 MIR376A1 STK36 RNU4-24P FAAH2 ZNF717 KLHL1 TRPC5 TEP1 NF1P4 FAM90A20P SRRM1P2 YAF2 WDR95P KIDINS220 KIAA1644 CT49 SMPD4P2 HNRNPUP1 PLCB4 MRM1 F11-AS1 HNRNPA2B1 GADD45A RNF216 NHLRC2 DNM1P50 ZNF430 RCHY1 ZNF793 FTO MGC4294 USP46 DMXL2 STIM2 SNORD23 RNU6-1327P NF1P1 FAM201B SNORD54 RNA5SP492 C8orf46 EIF3A C10ORF68 HS1BP3-IT1 SEC24B FRG1 ADAM28 IGSF11-AS1 TMED8 SDHAF2 ZNF268 ENOX1 KRTAP4-11 RN7SKP60 UNC5C DNM1P28 PRIMA1 ACSBG1 MIR648 ANKRD36C NKAIN1P1 ANKRD26P4 POLR2F KRTAP5-8 CSGALNACT1 IZUMO3 CSMD2 RNU6-1005P PTPN20A KRTAP20-1 SMAD1 ARHGEF15 OR1E3 ZNRF3-IT1 LST3 LINC00523 ATP1A4 RPL21P6 ANKHD1 VPS37A KRTAP12-4 LINC00189 BAZ2B LARS2-AS1 C1QTNF6 POU6F2 TTC8 BRD7 FCF1P10 SNORD6 TENM3 RNU6-280P KRTAP27-1 DUX4L19 SFI1 GNE OR52N5 RBMX2P1 TSPEAR-AS1 HMG20A CEP85L ATF2 ARL6IP5 TUSC3 COPG2 ARFIP1 RNU4-60P PLSCR1 PAPPA-AS1 RNU6-156P PHACTR2 CD163L1 ZNF72P ZNF880 NOX5 C1orf167 EEF1A1P1 RNA5SP283 RBBP8 KCNJ12 OR51AB1P ATP5J SRP54 STARD4 DNAAF2 TAS1R2 MTAP ADORA3 COL6A5 TTI1 SH2D7 NSUN6 HYAL4 BACE2-IT1 SP2 TMEM38B CCDC91 MMP26 GRID2 CALN1 CYTIP CYBB LRP2 UXS1 SPTA1 ZNF483 SEMA6D KCNJ3 ZNF573 PCDHGA8 RALGPS2 DPP9 RGL2 FAM27E4 SNRK PMPCB DHRS4L2 GLDC TTC29 SUZ12 MACC1 OR4C10P CXorf21 ZNF415 IL17RA YTHDF2 BLZF2P ZNF729 MIR548AS LHFP TSPY5P CAMK4 SERPINA9 RN7SL177P TMEM189-UBE2V1 PJA1 IGLVIV-65 RNU6-721P DKFZP761J1410 MZF1 VWC2 GUCY2F ANKRD20A17P IGHVII-40-1 COL16A1 TTC22 KRTAP6-1 CELF2 OR5D15P ATP8A2P1 SMIM2-AS1 TP53I11 CACNA1C-IT1 SHOC2 PDXDC1 NTNG1 HHLA1 ZNF606 ADTRP PDE4B DDX10 MIR5095 IGLV2-8 OR4C2P SNORD116-18 FBXL7 MAPRE2 IL1RN ZNF26 PCDH17 PHF8 TFPI USP17L9P MIR323B ISX RAD51AP1 ABCA9 SGCD SMTN TMEM108 CA3 PQLC1 SEC14L6 CYB5R2 GABRB3 TPTE CKS1BP5 BFSP1 LRRC20 DSCR9 MIR3182 ANKRD20A11P LPPR1 LCP2 RMRPP4 SEZ6L NAP1L4P3 SLC39A8 PET117 IL4R SHROOM4 NAV3 RNU7-144P TM4SF1 IGHV3-29 SMG7 MX1 ZNF564 PAXBP1 PLGRKT ANKRD13A ZCCHC11 COPS8P3 RNU6-288P RNA5SP219 KIAA1211 IQSEC3 TGFBR3 ZNF782 RNU6-772P PNLIPRP3 PPA2 MAP3K7 UPRT AKR1C1 ZNF615 MIPOL1 GTF2I DNAH3 C16orf80 C15orf32 SLC7A1 PTPRM ATXN1 ACTR3BP5 KIRREL3 ADAM17 USP36 CHRNA3 OR6C68 TMEM186 MIR548W AK5 SLC35F2 BNIP3L IGKV1OR-1

HAPLN3 GALNT8 PRKCH PCDHGB6 NRXN3 RFPL4AP7 OR7D1P RMRPP5 WBP11P1 CYP2AC1P OR6N1 RHPN2 ANKRD30A FSBP KRT25 HMGN3P1 C9orf40 ASXL1 KCNH7 NUAK2 GNG5P5 KRTAP15-1 LCE2C NUDT4 SNORA70C RNU1-104P CLDN1 PCTP TSNAX-DISC1 FOXD4L5 WFDC8 IGHV7-56 SPRR2G C14orf64 TMEM211 NSG1 PLEKHG1 RBAK-RBAKDN ANKRD20A8P WDR83OS BCRP1 KCND2 NSUN7 WIPF1 IGF2R C21orf90 PTPRU LY86-AS1 NHSL2 SNTB1 SLC24A3 TGFB1I1 FAM228A SOBP TSGA13 SHOX2 NPY4R RNU6-530P MIR381 EGLN3-AS1 BMS1P15 ZNF207 MPRIP KLF13 RN7SL153P RNASE12 RN7SKP100 NMNAT1P4 IGHVIII-67-2 GRIA4 ANKRD32 CERS6 MARK2P9 VPS26AP1 TMPRSS12 ATP8B5P USP32P1 TPH2 ZNF91 WDYHV1 SNORD115-10 KIAA1598 PATE4 MORF4L1 CNNM2 APC PMP22 FAM108A8P ELK3 ZNF233 FAM111B OLFM3 INADL RRP15 GPR139 LINC00508 COX5BP6 ADNP2 PLEKHA5 OR5AK4P MACROD2 TTLL5 IL18R1 SGK3 BNIP3P7 FAM90A21P INO80 MARCH11 NAA11 TMTC1 TSHR TIMD4 LINC00317 CCDC7 OR4A43P RGS7BP TFF1 FSD1L EPHB2 LPHN2 MTND6P4 PLEKHA6 COL27A1 PHLDB2 BMS1P17 RNU6-1293P TEX11 SCAF4 ESR2 SYT16 PRR9 MTND5P14 RALBP1 IGHV3-47 RNU6-16P ZCWPW2 KAT7 HNRNPA1L2 IFI27L2 ADARB2 MIR376C MYBPC2 ZNF235 OR6K4P ACSL4 CAPZB SATB1 IGHV4-31 ERC1 PPM1D OR10N1P ZNF850 C14orf177 ST20-MTHFS COMT NXPH2 ROS1 MACC1-AS1 PCDHA6 NRG1-IT2 SLC5A4 DGCR10 AGO3 SNX19P1 SLC6A17 STT3B RNA5SP405 ZBED5 MAGEB2 CD1C DUX4L16 CKAP4 SLFN12L CCDC13 MCTP1 RNU1-51P MOB3B SNORA16 PGK1 RNASEH1 MIR656 RPL10P3 PRCP RNF135 NBAS MTND1P17 RN7SKP86 NBN PRTG PANX1 RNA5-8SP6 C12orf42 ADAMTS18 SEPT14 MIR767 CCDC59 EHF LINC00884 RNA5SP20 MAST2 COL22A1 KLRF2 RPS15AP1 RNU6-1320P TC2N SETD2 KCNQ5-IT1 RASSF3 CYCSP6 KRTAP9-3 RNU6-494P SNORD116-19 LGR6 ZBTB34 SYNE2 ZNF826P ENPP7P2 RNU6-954P SQRDL DOCK2 BAI3 TRIM23 DPYSL2 MAGEC3 SDCCAG8 EXOC5 FPR3 MIR381HG RN7SL17P OR4Q1P DSCR8 SLCO1B3 CYLC2 PTPDC1 FLVCR1 PPM1J RAB3C CEP135 NRP1 TRIM22 CDH13 UBE2E3 MDGA2 SAMD3 METTL8 BTNL9 VEPH1 RFC3 FAM129A OOSP1P2 BCAN AKAP7 RNU6-953P OR4C7P TGM2 STAM MIR4535 BASP1 MARCH10 GRM8 ARHGEF7 GRK6 MIR382 PHACTR3 C7orf69 CRLS1 ZNF277 7SK SQSTM1 ANKRD26P1 ZNF879 SAMD12 DACH1 PHKA2 TRDN RNU6-1066P DEFB116 KRTAP4-3 APOL6 KRT39 ZNF397 DAB1 MIR1276 MIR5704 DPRXP4 KRT8P25 LINC00442 DICER1 ZNF148 TEKT4P2 PCDHA4 CHODL-AS1 SNTG1 GGT8P SULT1A4 C5orf38 THOC2 SLC16A12 UBQLN4 TAF1B LINC00898 LINC00906 COL15A1 IGHV3-63 CACHD1 EXOC6B PPP1R26P2 IGKV1OR2-2 EVC2 LINC01019 GREB1 FAM27E3 NUP62CL ADAT2 ARNT TCAM1P BDNF MIR320B2 CHMP1B2P EIF4EBP3 OR51F1 ACBD6 CCDC26 CSF1 REST DNAH6 DSCR4-IT1 REXO1L3P MGAT5 TSPAN8 IGHVII-44-2 MRPS11 C2orf27A MYO5B STOML1 MAEL SDR42E1 LINC01057 RNU6-127P RNU6-469P SNORD109B TMEM233 RNA5SP465 BDKRB2 RNF180 TAS2R41 H3F3AP4 DUX4L5 RNF215 NDST4 PACRGL MIR543 ADORA2A MAGI3 FAM90A23P STIM1 RNA5SP495 TSEN15 MIR521-2 ANKRD30BP1 CCDC122 SYPL2 COL21A1 TUBA3C FAM135B GPATCH2L BACH1-IT3 TSC22D3 KIF16B CASP8 SWT1 NRIP1 IGLJ1 CDH2 MROH7 RNU6-26P CECR7 ARF1 DPYD-IT1 ARID5B FLNC SIPA1L2 CCNG2 PLCH2 RCAN2 LRRC69 CASC18 TMEM261 MTMR8 RPL31P3 DSG2 PPIAP22 CEA TANC1 JAG1 CNN3 MIR105-2 CACNA1B PAPPA SERPINB7 AKNAD1 EVI5 FAM27A IGHVII-65-1 VPS41 IFT80 BMS1P13 LINC00879 AKR1C2 MYBL2 MIR3118-4 RN7SL373P HSPA4L ZNF527 RPL21P11 POU5F1P6 FAM208B HDAC9 DYM METTL15 OR5J7P CUZD1 CPB2 UHMK1 RNF219-AS1 ELOVL7 LINC00351 CHST13 GOLGA1 ANKRD20A18P VDAC2P1 NR3C1 PPIAP6 NEK7 MIR300 GPR133 BRF1 FSTL4 FAM108A10P PFN2 ACTR3BP6 CDCA7L MTOR FAM222B MCM3 SLC19A3 C17orf75 ZNF585B RNU6-690P WT1 OR4C5 CYP3A54P CCDC170 STK38L RN7SKP197 HNRNPA1P40 KSR1 CBLB RORB KIAA1407 CDK6 LARGE VPS39 VAPB CFL1P6 GABRB1 TUFT1 SNAP25 FBLN1 MTFMT LUC7L RAF1 ANO1 ABCD1P5 TPTEP1 ST8SIA1 JAKMIP1 SH3KBP1 PCMTD1P1 RIMS3 GRPEL2P1 SIRPB1 RTFDC1 MIR4290 MIR410 RPL4P2 FHL2 CDC42BPG HIGD1A CADPS LTB LINC01101 OR4A41P NEU3 ZFPM1 BZW1 PIP5K1A GDAP1 OR51H2P RPL8P2 ZRANB1 RNA5SP222 RGS7 TPTE2P5 DISP1 PRB2 MYCT1 CPT1B SCGN KYNU MS4A5 GPN3 RNU6-21P ZFP30 TNMD MTND5P5 RN7SKP101 SPATA6 BEND7 CLVS2 RN7SL609P STXBP5 FAM212B TBXAS1 OSBPL9P4 KXD1 OR2BH1P USP25 RN7SL674P BMS1P14 RN7SKP139 FCRL2 FAM196B SULT1C2P1 H3F3C SPRED1 BMS1P9 HBD KBTBD11 NPR3 SIPA1L3 MROH2B REV1 LIPA GALC RNU6-1269P TMC1 GNG4 CNTNAP4 TTLL11-IT1 MRPS22 TSPAN33 TTC32 LINC00393 CDH26 NOX4 IGKV1-37 CES1P2 LRFN2 FLNB RNU6-1049P ACO1 UBE2Q2P11 WDFY4 SCAI RNU6-157P MEGF9 MIR5190 OR4C12 RNU1-11P SNORA32 PGM5P2 STK33 NCOR1P3 DIRAS2 KRT18P31 POTEA ABCB5 PCDHGA9 NAPEPLD MIR3648 GCNT7 OBSCN SPECC1 FCF1P9 OR4Q2 DPY19L2 GACAT1 FTLP15 C20orf196 NCOR1P2 CYYR1 IGKV2OR2-1 IL1RAP UBXN2B KIR3DL1 NEK2P2 MRPL3P1 FAM118A TAF4 HNRNPA1P58 TRIM9 SETD5-AS1 KY IGHV3-48 WRAP73 CSMD1 SNORA38 PLEKHH2 RPS3AP1 SNORD115-24 SLC24A2 CENPBD1P1 LRRTM1 VAV2 COL25A1 MTND5P11 PPIAP27 FAM90A7P SPRR1A VPS13A SCEL LRRC9 SNX20 GRIA3 NSUN3 RNA5SP488 GOPC RNU6-400P RP2 NTRK3 ZFRP1 OR5E1P LINC00457 MIAT DPRXP5 POLR3C SLC35F3 SGCG FBN1 DCDC2 NANP ZNF621 ANKRD20A12P SEMA4B DYNC1I2 NIPAL2 LONP2 HYDIN RAB31 AGGF1P4 LGALS14 DHRS3 CPPED1 LINC00418 GAB2 TXNDC16 CEP89 VPS13D YRDCP3 DIS3L2 ZNF292 FREM2 ZNF829 TBX15 C21orf91-OT1 BMPER hsa-mir-6723 NOS2P3 SLC7A8 ANKRD31 TMEM41B FAM149B1 MYO5BP3 SPATA7 S100A11 DOT1L EFHC2 ZNF521 PDE1A ZBTB8A RNU7-35P SNORD113-2 SMYD2 TMPRSS2 OR10R2 MS4A6E TMEM194B LINC01036 ONECUT3 ZMAT3 TRAV8-4 IPPKP1 DUXAP10 OR4H12P VIPR2 HMGA2 B3GALTL MIR1185-2 HHAT TMPRSS15 MTATP6P1 ZNF558 MLLT10P2 MX2 CREB5 RPA3-AS1 B3GAT2 FAM227B THSD7B LAMP5 EFCAB4B PSPC1P2 UBE2G2 ACSM5 KIAA0040 HLCS-IT1 RN7SL683P KRT2 CA5AP1 ZNF618 RBKS DNAJC6 CLOCK DSG4

MIR105-1 CPA6 U3 CPNE8 DACH2 DEFA3 PGM2 LINC00521 M1AP AGBL4-IT1 NUS1P2 ZC3H13 ZNF610 WBP1L RXRG DNAH10OS KIR2DS4 H2BFM KDR MIR3118-5 SORCS1 BRD9 PCDHGA2 TBC1D4 OR2M5 OGT DNM3 SYT1 OR4K1 MSL2 MOXD1 FAM13C SYNDIG1 OR5AO1P DPF3 HNRNPA1P68 KCNT2 DHX32 TMEM45B SCAPER GZMH CRYBB2P1 CDK2AP2P3 RNU5A-5P OR4K13 MED4 PTPRS NPHP4 IKZF2 PNPT1P1 DOCK9 LINC00856 IGHV3-32 DUX4L17 PML FAM101A AGBL3 SACS TTLL12 RBCK1 AFF2 MIR650 IGHV3-75 NCKAP5 PLXNA2 ZDHHC15 THAP7-AS1 RN7SL552P FAM27E2 FAM83G SCFD2 PTPRD RNA5-8SP5 RORA CYP4X1 IL2RA TRAPPC12 STXBP5L REXO1L2P SHISA6 FAP NSF ANKRD20A2 SCARA5 OR52U1P TPT1P5 ERICH2 HDX SCPEP1 IGKV2OR22-4 RN7SKP85 METTL9 ATP5O snoMe28S-Am2634 LINC00923 LINC00200 RBAKDN PCDHGB8P RPS20 SEPP1 LINC00885 LOXL2 IGHV1OR15-3 AGGF1P1 LINC01146 ATAD2 ZNF229 RPS10P7 LRRK1 SLC2A12 GRIK1-AS2 RNA5SP221 PIWIL4 MRGPRG ZNF571 PRKG1 UBFD1P1 RASGRP1 CTBP2P1 PLXDC2 ELAVL4 SNORD116-16 CADM3 CDH9 PIP5K1P2 KRTAP9-8 KIR2DL3 FOXRED2 IGHV3-65 IGLV2-18 GLTSCR1 AK4 NDUFAF2 DGUOK-AS1 SNORD116-26 KREMEN1 FMNL3 STXBP6 SNAP23 ANXA8L1 WDR25 CYP4Z1 CHST9 DMBT1 MGA LINC00664 MXI1 SPTLC2 USP17L26 TTC28 MTURN GALK2 NELL1 GRAMD4P5 GYG1P1 ANKRD30B SNX18P15 CEP97 PCDH19 SRIP1 ZNF451 ADORA1 MIR514A1 STX12 VN1R87P MRPL39 OR11H13P MEG9 FAM46A SNORD27 RNU6-368P MCM9 GALNTL6 PTGR1 RNU6-405P KIAA1671 PXDNL ST7-OT4 SAP18 CCDC144CP ASIC2 FIP1L1 RALA DOCK10 SPCS2P4 IGDCC4 RN7SKP126 PRB1 EDDM3A CTXN2 GNPTAB RPS26P30 OR11K2P ABCC10 CACNG3 RARRES2P1 TMEM173 COL2A1 RPA1 CNTNAP3 PPP4R1L PPIAP14 FNDC3A MIR4760 NAP1L4P1 LINC00617 MBD3L1 KHDRBS3 SLC30A10 NECAB1 MYO5BP1 TPK1 MICAL2 SOX8 ATP8A1 KIAA0195 MTHFS ATXN8OS CALCRL PTP4A1P1 RNU1-150P RPS24P12 SLC22A25 EGFL6 BCL2L1 BRSK2 LGALS9 SCARNA21 GBP6 MIR495 DRD5P2 OR5J2 MICAL3 TNNI3K HDAC2 THBS2 RANBP17 TPO IGLV3-19 ETS1 MRPS27 MLST8 SMOC1 TMEM138 ADAT3 C9orf131 TMEM131 RNU6-230P TRIM51CP MIR155HG RNA5SP497 RNASE11 IGHV3-43 OR8K5 MIR4480 BICC1 CRNN DISC1-IT1 HSH2D SCAMP1 SLX4IP SEMA3D KCNK13 BANP MTND2P4 CCDC169 DUX4L4 DDC YME1L1P1 MIR548AL RNU6-614P ZNF569 WFDC11 LYPD5 RN7SKP147 HNRNPA1P53 COX6A1P3 FLNB-AS1 FAT3 OR1AA1P CAB39 ENPP7P1 SNORA25 TMEM161A POTEH-AS1 RAD23BLP OR11G2 WSCD2 SERTM1 PCDHA3 SNORA70 MTMR2 LINC00707 AAGAB KCNH5 YWHAQP9 RRM1 FAM189A2 MFSD12 OR52B4 CTDSPL PDE11A RNU6-576P POLR3H TSPAN9 THUMPD1 SYCP2 MAT1A CLYBL KCNC4 USP32P3 PCCA RAB30 TMEM163 CEP41 MS4A7 CLVS1 GNB5 ATF6 PRKAR1B IL16 LINC00972 FAM189A1 IQCJ ZSWIM7 DEPTOR CCDC88C B4GALT3 IGHV3-60 NVL RNA5SP490 BACH1 ATAT1 TMEM220 PPFIBP2 POTEH EDARADD CENPV EWSR1 CREBRF DTX2P1-UPK3BP1-PMS2P11 RNU6-1239P CTBP2 ZMYND11 MYH8 SNORD113-1 RGS6 SRGAP3 ZNF525 RNF144A-AS1 NCALD PGM5P1 EFCAB2 SNX19P3 CACNA1C-IT2 MIR548X ZNF337 OXSR1 RAD51D MIR369 DYRK1A SARDH CUL2 RNASET2 CCDC146 PSG6 MIR4273 MYRIP FAM221A SLC39A11 RNU4-45P LINC00343 SCNN1A IGKV1OR22-5 KCNE2 ZNF443 DUX4L14 PCP4 OR5V1 RIMS2 KRTAP10-10 TMEM55A IGHVII-28-1 SYT17 RNU6-898P SART3 ACTA2-AS1 FOXP1 MAS1LP1 SLC5A1 SNORA80 C16orf95 DNAJC3-AS1 SNRPD3 STAC RBMX2P3 OR4M1 SCN8A IGHVIII-25-1 FRG1B MIR4300 KCTD16 FDXR RAB27A PNLIPRP1 DEFB122 PARP4P3 KSR2 TMTC4 L3MBTL3 HLA-DQB2 NOVA1-AS1 MROH7-TTC4 IGKV2OR22-3 ARL15 SGOL2 IGHV3OR16-12 SPATA13 ZNF736 CEP152 CLIP1 RNA5SP518 RARRES2P2 RUSC1-AS1 GUSBP6 TUBGCP6 MORN2 5S_rRNA CST2 GAREM PRDM15 PLAC4 OR4N2 BLOC1S6 MTND4P14 NUMA1 RFX4 RPL18AP14 EIF3FP1 ZNF330 ADAM20 LRRC16A SNORD115-45 AGAP1 FREM1 ATP6V1D SAA2 SNORD115-19 LAMA4 PMEPA1 C1orf94 RN7SL659P IGHV4-55 ZZZ3 ZNF112 RN7SL163P MIR548Q UBE3C PDSS2 CTNNA2 ATE1 CCDC92 RPSAP55 CEP44 MYO5BP2 PEX5L TERF1P1 OR8L1P CYP46A1 IGHV1OR15-2 CYB561A3 PARD3B SLC2A9 CHN1 FAM90A15P UCHL1-AS1 PRAMEF12 RNU6-249P HGSNAT RAG2 LCE2A GATAD2B FMNL2 RNU6-540P ANXA4 RNU6-458P IGKV3OR22-2 PARD3 KCNJ15 CEP112 snoU13 MYOM3 CCDC73 ATIC OR8S1 SYNPR-AS1 NPL NRG1 FAM110B CAST SNORD115-34 CES1P1 LGR4 GAB1 SYT9 FANK1 SULT1B1 ZNF845 DGCR2 DHRS4-AS1 LINC00534 VSIG10 RPS20P1 TRDC DLEU1 JPH3 SLC38A7 CYP4F29P GPR39 SLC22A10 RNF128 PXK HSPG2 SLC25A48 CP FAM160A1 SUCLA2 BTF3P10 ATP10B SLC35F4 NPAS3 AMD1 LCE4A ZNF562 SPRR2E DNAJA1P4 PGPEP1 MLTK CCNYL2 MARCH1 FMN2 TUBB1 SEMA3C GRAMD3 WDR93 CACNA1I ARMCX2 SEC22A DPY19L1 RPS12P21 LMBR1 C8orf44 DGKZP1 RNA5SP280 ZPBP KIAA0825 NUSAP1 PCNT BCKDHB PAH OR4K11P USP3 VPS13B LINC00940 DCLRE1C HBG1 ADRBK2 LINC00353 ZNF622 SELO OSBPL5 SNORD115-39 TOP1 TRDV3 RXFP2 EXOC6 SNORD115-25 RN7SKP199 SHISA5 MPST

Table S4. GO associations with Biological Process (GO Profiler) of 426 srRNA genes shown in Fig 1D. Related to Fig. 1E.

GO.ID	Description	padj	*Genes*
		Biological Process	
GO:0048666	neuron development	1.510663E-10	*OPHN1 ARID1B IL1RAPL1 WDPCP CTNNA1 MYO9A NTRK2 MDM2 PTPRG DMD KANK1 LAMA2 RAP1A CNTN4 RUNX1 RAPGEF1 PLS1*

			ROBO1 DOK6 CHODL RNF165 EPHA3 LYN DSCAM EPHA6 NLGN1 CAMK1D COBL RELN APP CCDC88A PAK1 HCN1 FRY PPP3CA RASGRF1 TIAM1 NTM PCDH15 PTPN9 INPP5F PPFIA2 OPCML RBFOX2 ATP8A2 AUTS2 ANKS1A SOS1 CTNND2 PTPN11 ALK DCLK1 TENM2 GLI3 DENND5A TNC NFASC MAGI2 PRKD1 VCL ROBO2 SEMA5A BCL11A DCC
GO:0031175	neuron projection development	2.730268E-09	*OPHN1 ARID1B IL1RAPL1 CTNNA1 MYO9A NTRK2 MDM2 PTPRG DMD KANK1 LAMA2 RAP1A CNTN4 RAPGEF1 PLS1 ROBO1 DOK6 CHODL RNF165 EPHA3 LYN DSCAM EPHA6 NLGN1 CAMK1D COBL RELN APP CCDC88A PAK1 FRY PPP3CA RASGRF1 TIAM1 PCDH15 PTPN9 INPP5F PPFIA2 RBFOX2 ATP8A2 AUTS2 SOS1 CTNND2 PTPN11 ALK DCLK1 GLI3 DENND5A TNC NFASC MAGI2 PRKD1 VCL ROBO2 SEMA5A BCL11A DCC*
GO:0031344	regulation of cell projection organization	6.446895E-08	*IL1RAPL1 WDPCP MYO9A NTRK2 MDM2 PTPRG DMD KANK1 RAP1A RAPGEF1 PLS1 ROBO1 SEPT9 CHODL FXR1 EPHA3 LYN DSCAM NLGN1 CAMK1D COBL RELN CCDC88A PAK1 PPP3CA TIAM1 PTPN9 INPP5F HDAC4 PPFIA2 ATP8A2 AUTS2 CROCC ALK TENM2 DENND5A MAGI2 PRKD1 ROBO2 SEMA5A BCL11A DCC*
GO:0120035	regulation of plasma membrane bounded cell projection organization	1.077281E-07	*IL1RAPL1 WDPCP NTRK2 MDM2 PTPRG DMD KANK1 RAP1A RAPGEF1 PLS1 ROBO1 SEPT9 CHODL FXR1 EPHA3 LYN DSCAM NLGN1 CAMK1D COBL RELN CCDC88A PAK1 PPP3CA TIAM1 PTPN9 INPP5F HDAC4 PPFIA2 ATP8A2 AUTS2 CROCC ALK TENM2 DENND5A MAGI2 PRKD1 ROBO2 SEMA5A BCL11A DCC*
GO:0030182	neuron differentiation	1.616046E-07	*OPHN1 ARID1B IL1RAPL1 WDPCP CTNNA1 MYO9A NTRK2 MDM2 PTPRG DMD KANK1 LAMA2 RAP1A CNTN4 TCF12 RUNX1 ALDH1A2 RAPGEF1 PLS1 ROBO1 DOK6 CHODL RNF165 EPHA3 LYN DSCAM EPHA6 NLGN1 CAMK1D COBL RELN APP CCDC88A PAK1 HCN1 FRY PPP3CA RASGRF1 TIAM1 NTM PCDH15 PTPN9 INPP5F PPFIA2 OPCML RBFOX2 ATP8A2 AUTS2 ANKS1A SOS1 CTNND2 PTPN11 ALK DCLK1 TENM2 GLI3 DENND5A TNC NFASC MAGI2 PRKD1 VCL ROBO2 SEMA5A BCL11A DCC*
GO:0010975	regulation of neuron projection development	3.726668E-07	*IL1RAPL1 NTRK2 MDM2 PTPRG DMD KANK1 RAP1A RAPGEF1 ROBO1 CHODL EPHA3 LYN DSCAM NLGN1 CAMK1D COBL RELN CCDC88A PAK1 PPP3CA TIAM1 PTPN9 INPP5F PPFIA2 ATP8A2 ALK DENND5A MAGI2 PRKD1 ROBO2 SEMA5A BCL11A DCC*
GO:0031346	positive regulation of cell projection organization	3.973052E-07	*IL1RAPL1 NTRK2 DMD RAP1A RAPGEF1 ROBO1 SEPT9 CHODL EPHA3 LYN DSCAM NLGN1 CAMK1D COBL RELN CCDC88A PAK1 TIAM1 HDAC4 ATP8A2 AUTS2 CROCC ALK TENM2 MAGI2 PRKD1 ROBO2 SEMA5A BCL11A*
GO:0048699	generation of neurons	6.618628E-07	*OPHN1 ARID1B IL1RAPL1 WDPCP CTNNA1 MYO9A NTRK2 MDM2 PTPRG DMD KANK1 LAMA2 RAP1A NOS1 CNTN4 TCF12 RUNX1 ALDH1A2 RAPGEF1 PLS1 ROBO1 DOK6 CHODL RNF165 EPHA3 LYN DSCAM EPHA6 NLGN1 CAMK1D COBL CIT RELN APP CCDC88A PAK1 HCN1 FRY PPP3CA RASGRF1 TIAM1 NTM PCDH15 PTPN9 INPP5F PPFIA2 OPCML RBFOX2 ATP8A2 AUTS2 ANKS1A SOS1 CTNND2 PTPN11 ALK DCLK1 TENM2 GLI3 DENND5A TNC NFASC MAGI2 PRKD1 CELSR1 VCL ROBO2 SEMA5A BCL11A DCC*
GO:0007399	nervous system development	9.051198E-07	*OPHN1 LRRTM3 KALRN MALL ARID1B IL1RAPL1 WDPCP CTNNA1 MYO9A NTRK2 SH3TC2 MDM2 PTPRG FBXL17 DMD CHRM3 MYO1D KANK1 CHST8 LAMA2 RAP1A NOS1 CNTN4 TCF12 RUNX1 ALDH1A2 RAPGEF1 PLS1 ROBO1 DOK6 CHODL RNF165 CLSTN1 EPHA3 LYN DSCAM EPHA6 CA10 NLGN1 SHROOM3 CAMK1D PCDH9 COBL CIT RELN APP CCDC88A PAK1 HCN1 FRY PPP3CA RASGRF1 TIAM1 SNTG2 NTM PCDH15 PTPN9 INPP5F HDAC4 PPFIA2 OPCML LINGO2 RBFOX2 IMMP2L DLC1 ATP8A2 AUTS2 WLS ANKS1A SOS1 CTNND2 PTPN11 ALK*

			DCLK1 TENM2 TFAP2D GLI3 DENND5A TNC NFASC PLCB1 BPTF MAGI2 PRKD1 ABAT CELSR1 VCL ROBO2 SEMA5A BCL11A DCC ETV6 DNAH5
GO:0022008	neurogenesis	2.523294E-06	*OPHN1 ARID1B IL1RAPL1 WDPCP CTNNA1 MYO9A NTRK2 SH3TC2 MDM2 PTPRG DMD KANK1 LAMA2 RAP1A NOS1 CNTN4 TCF12 RUNX1 ALDH1A2 RAPGEF1 PLS1 ROBO1 DOK6 CHODL RNF165 EPHA3 LYN DSCAM EPHA6 NLGN1 CAMK1D COBL CIT RELN APP CCDC88A PAK1 HCN1 FRY PPP3CA RASGRF1 TIAM1 NTM PCDH15 PTPN9 INPP5F PPFIA2 OPCML RBFOX2 ATP8A2 AUTS2 ANKS1A SOS1 CTNND2 PTPN11 ALK DCLK1 TENM2 GLI3 DENND5A TNC NFASC MAGI2 PRKD1 CELSR1 VCL ROBO2 SEMA5A BCL11A DCC ETV6*
GO:0048667	cell morphogenesis involved in neuron differentiation	3.764176E-06	*OPHN1 IL1RAPL1 WDPCP NTRK2 LAMA2 CNTN4 PLS1 ROBO1 DOK6 CHODL RNF165 EPHA3 DSCAM EPHA6 NLGN1 COBL RELN APP PAK1 PPP3CA TIAM1 PCDH15 PPFIA2 RBFOX2 ATP8A2 AUTS2 SOS1 CTNND2 PTPN11 DCLK1 GLI3 NFASC VCL ROBO2 SEMA5A BCL11A DCC*
GO:0034330	cell junction organization	1.051547E-05	*ROCK2 OPHN1 LRRTM3 CACNG2 IL1RAPL1 WDPCP CTNNA1 MYO9A NTRK2 RAP1A CORO2B RUNX1 CDH18 RAPGEF1 PKP4 ACTN2 CLSTN1 EPHA3 LRRTM4 DSCAM NLGN1 RELN APP UTRN TIAM1 ERC2 PPFIA2 LINGO2 DLC1 CTNND2 CADM2 PTPRT ABHD17C TNC DLG2 NFASC MAGI2 VCL ROBO2 UNC13C*
GO:0120036	plasma membrane bounded cell projection organization	1.893996E-05	*OPHN1 ARID1B IL1RAPL1 WDPCP CTNNA1 MYO9A NTRK2 MDM2 PTPRG DMD KANK1 LAMA2 RAP1A CNTN4 RAPGEF1 PLS1 ROBO1 SEPT9 DOK6 CHODL ACTN2 RNF165 FXR1 EPHA3 LYN DSCAM EPHA6 NLGN1 CAMK1D COBL RELN APP CCDC88A PAK1 FRY PPP3CA RASGRF1 TIAM1 PCDH15 PTPN9 INPP5F HDAC4 PPFIA2 RBFOX2 ATP8A2 AUTS2 SOS1 CTNND2 CROCC PTPN11 ALK DCLK1 TENM2 GLI3 DENND5A TNC NFASC MAGI2 PRKD1 VCL ROBO2 SEMA5A BCL11A DCC DNAH5*
GO:0030030	cell projection organization	2.124396E-05	*OPHN1 ARID1B IL1RAPL1 WDPCP CTNNA1 MYO9A NTRK2 MDM2 PTPRG DMD KANK1 LAMA2 RAP1A CNTN4 RAPGEF1 PLS1 ROBO1 SEPT9 DOK6 CHODL ACTN2 RNF165 FXR1 EPHA3 LYN DSCAM EPHA6 NLGN1 DNAH9 CAMK1D COBL RELN APP CCDC88A PAK1 FRY PPP3CA RASGRF1 TIAM1 PCDH15 PTPN9 INPP5F HDAC4 PPFIA2 RBFOX2 ATP8A2 AUTS2 SOS1 CTNND2 CROCC PTPN11 ALK DCLK1 TENM2 GLI3 DENND5A TNC NFASC MAGI2 PRKD1 VCL ROBO2 SEMA5A BCL11A DCC DNAH5*
GO:0048468	cell development	2.559540E-05	*ROCK2 OPHN1 RCAN1 MAP2K4 ARID1B IL1RAPL1 WDPCP RPS6KA2 CTNNA1 MYO9A NTRK2 SH3TC2 MDM2 PTPRG DMD KANK1 LAMA2 RAP1A DIAPH2 NOS1 CNTN4 RUNX1 ALDH1A2 RAPGEF1 PLS1 ROBO1 DOK6 CHODL ACTN2 RNF165 EPHA3 LYN DSCAM EPHA6 NLGN1 SHROOM3 CAMK1D COBL RELN APP CCDC88A PAK1 HCN1 FRY PPP3CA RASGRF1 NEBL TIAM1 NTM PCDH15 PTPN9 INPP5F HDAC4 PPFIA2 AKAP6 OPCML RBFOX2 ATP8A2 AUTS2 ANKS1A SOS1 CTNND2 PTPN11 ALK DCLK1 TENM2 SYCP1 GLI3 DENND5A TNC NFASC PLCB1 MAGI2 PRKD1 VCL ROBO2 AKAP13 SEMA5A BCL11A DCC*
GO:0007267	cell-cell signaling	2.744946E-05	*SYN3 CCNE1 OPHN1 DLGAP1 CACNG2 BTBD9 RNF43 TRHDE ARID1B IL1RAPL1 NTRK2 ZNRF3 CHRM3 KANK1 LAMA2 RAP1A SH2D1A NOS1 CNTN4 GRIK4 RUNX1 RAPGEF1 GPR176 PKP4 FCHSD2 PRICKLE2 CLSTN1 LYN NLGN1 CSNK1G3 TCF7L2 RELN APP DKK2 GPC5 PPP3CA RASGRF1 TIAM1 SORCS2 GABRG3 ERC2 WWOX TNRC6B NXN NAMPT CTNNBIP1 PPFIA2 SORCS3 GRM7 GABBR2 WLS CTNND2 PTPN11 PSMB2 GLI3 RNF220 TNC DLG2 PLCB1 MAGI2 PLCL1 EDA ABAT CELSR1 RYR2 UNC13C SEMA5A DCC CASK*
GO:0007155	cell adhesion	2.820753E-05	*EPB41L4B ROCK2 MEGF11 IL1RAPL1 WDPCP CTNNA1 PDZD2 DMD ITGAE KANK1 KIF26B LPP LAMA2 CNTN4 CORO2B RUNX1 CDH18 RAPGEF1 ROBO1 PKP4 ACTN2 CLSTN1 EPHA3 LYN DSCAM NLGN1 PCDH9 RELN APP ADAM12 PAK1 UTRN PPP3CA RIN2 AP3B1 TIAM1*

			NTM PCDH15 PPFIA2 OPCML SSPN DLC1 PLAUR CTNND2 PTPN11 CADM2 TENM2 FAF1 PTPRT GLI3 CTNNA3 TNC DLG2 NFASC MAP2K5 EDA ABAT CELSR1 VCL ROBO2 SEMA5A CASK
GO:0022610	biological adhesion	3.359267E-05	*EPB41L4B ROCK2 MEGF11 IL1RAPL1 WDPCP CTNNA1 PDZD2 DMD ITGAE KANK1 KIF26B LPP LAMA2 CNTN4 CORO2B RUNX1 CDH18 RAPGEF1 ROBO1 PKP4 ACTN2 CLSTN1 EPHA3 LYN DSCAM NLGN1 PCDH9 RELN APP ADAM12 PAK1 UTRN PPP3CA RIN2 AP3B1 TIAM1 NTM PCDH15 PPFIA2 OPCML SSPN DLC1 PLAUR CTNND2 PTPN11 CADM2 TENM2 FAF1 PTPRT GLI3 CTNNA3 TNC DLG2 NFASC MAP2K5 EDA ABAT CELSR1 VCL ROBO2 SEMA5A CASK*
GO:0051128	regulation of cellular component organization	5.138325E-05	*ROCK2 DNAJC15 OPHN1 LRRTM3 BTBD9 IL1RAPL1 WDPCP GCLC RPS6KA2 MYO9A NTRK2 MDM2 PTPRG PDE4DIP DMD RPTOR KANK1 RAP1A TFDP2 NOS1 TBC1D5 CORO2B RUNX1 RAPGEF1 PLS1 ROBO1 SEPT9 FCHSD2 CHODL ACTN2 FXR1 CLSTN1 EPHA3 LYN DSCAM NLGN1 CAMK1D SMG6 COBL CLEC16A PLEKHM2 RELN APP CCDC88A PAK1 PPP3CA SPIRE2 STON1 TIAM1 NSD1 KANK3 PTPN9 GSG1L INPP5F HDAC4 CTNNBIP1 PPFIA2 AKAP6 LINGO2 DLC1 ATP8A2 AUTS2 PLAUR CROCC PTPN11 ALK LDLRAD4 TENM2 PSMG2 FAF1 PTPRT ABHD17C ATF7IP DENND5A MAP2K5 PLCB1 DNMT1 MAGI2 PRKD1 CELSR1 VCL ROBO2 AKAP13 SEMA5A BCL11A DCC*
GO:0023051	regulation of signaling	5.677285E-05	*EPN2 BANK1 SYN3 ROCK2 OPHN1 CAMTA1 DLGAP1 RCAN1 OTUD7A KALRN CACNG2 BTBD9 RNF43 MAP2K4 GCLC STK39 ARHGAP8 CTNNA1 MYO9A NTRK2 CCDC3 SH3TC2 MDM2 ZNRF3 NCOR1 FBXL17 SLC39A10 DMD SPRED2 RPTOR KANK1 LAMA2 RAP1A SH2D1A NOS1 RGS3 CNTN4 GRIK4 RUNX1 RAPGEF1 ROBO1 ACTN2 RNF165 CLSTN1 LYN NLGN1 HELLS TNFRSF10B CSNK1G3 CLEC16A HERC4 TCF7L2 RELN APP CCDC88A PAK1 DKK2 GPC5 PPP3CA WDR59 RASGRF1 OTUD4 DEPDC5 TIAM1 SORCS2 USP18 POR WWOX GSG1L INPP5F SHC2 NXN CTNNBIP1 PPFIA2 AKAP6 SORCS3 NPLOC4 GRM7 DLC1 AUTS2 PLAUR WLS ANKS1A RYR3 SOS1 CTNND2 PTPN11 ALK LDLRAD4 RALGAPA2 FAF1 PSMB2 CYP7B1 PTPRT GLI3 NFAM1 RNF220 DLG2 MGLL MAP2K5 PLCB1 MAGI2 FLT3 PLCL1 EDA PRKD1 ABAT RYR2 ROBO2 AKAP13 UNC13C EYA4 SEMA5A DCC CASK*
GO:0010646	regulation of cell communication	6.323497E-05	*EPN2 BANK1 SYN3 ROCK2 OPHN1 CAMTA1 DLGAP1 RCAN1 OTUD7A KALRN CACNG2 BTBD9 RNF43 MAP2K4 GCLC STK39 ARHGAP8 CTNNA1 MYO9A NTRK2 CCDC3 SH3TC2 MDM2 ZNRF3 NCOR1 FBXL17 SLC39A10 DMD SPRED2 RPTOR KANK1 LAMA2 RAP1A SH2D1A NOS1 RGS3 CNTN4 GRIK4 RUNX1 RAPGEF1 ROBO1 ACTN2 RNF165 CLSTN1 LYN NLGN1 HELLS TNFRSF10B CSNK1G3 CLEC16A HERC4 TCF7L2 RELN APP CCDC88A PAK1 DKK2 GPC5 PPP3CA WDR59 RASGRF1 OTUD4 DEPDC5 TIAM1 SORCS2 USP18 POR WWOX GSG1L INPP5F SHC2 NXN CTNNBIP1 PPFIA2 AKAP6 SORCS3 NPLOC4 GRM7 DLC1 AUTS2 PLAUR WLS ANKS1A SOS1 CTNND2 PTPN11 ALK LDLRAD4 RALGAPA2 FAF1 PSMB2 CYP7B1 PTPRT GLI3 NFAM1 RNF220 DLG2 MGLL MAP2K5 PLCB1 MAGI2 FLT3 PLCL1 EDA PRKD1 ABAT RYR2 ROBO2 AKAP13 UNC13C EYA4 SEMA5A DCC CASK*
GO:0120039	plasma membrane bounded cell projection morphogenesis	8.461055E-05	*OPHN1 IL1RAPL1 MYO9A NTRK2 DMD KANK1 LAMA2 CNTN4 ROBO1 DOK6 CHODL RNF165 EPHA3 DSCAM EPHA6 NLGN1 COBL RELN APP PAK1 PPP3CA TIAM1 PPFIA2 RBFOX2 ATP8A2 AUTS2 SOS1 CTNND2 PTPN11 DCLK1 GLI3 NFASC VCL ROBO2 SEMA5A BCL11A DCC*
GO:0048858	cell projection morphogenesis	9.805225E-05	*OPHN1 IL1RAPL1 MYO9A NTRK2 DMD KANK1 LAMA2 CNTN4 ROBO1 DOK6 CHODL RNF165 EPHA3 DSCAM EPHA6 NLGN1 COBL RELN APP PAK1 PPP3CA TIAM1 PPFIA2 RBFOX2 ATP8A2 AUTS2 SOS1 CTNND2 PTPN11 DCLK1 GLI3 NFASC VCL ROBO2 SEMA5A BCL11A DCC*
GO:0048731	system development	1.132354E-04	*EPN2 ROCK2 OPHN1 LRRTM3 RCAN1 KALRN MEGF11 MAP2K4 MALL ARID1B IL1RAPL1 WDPCP ADAMTS6 RPS6KA2 CTNNA1 MYO9A NTRK2*

			FOXN3 SH3TC2 MDM2 ZNRF3 PTPRG PLAC1 SLC24A4 FBXL17 NHS DMD CHRM3 SPRED2 MYO1D KAZN KANK1 KIF26B CHST8 LAMA2 RAP1A TFDP2 NOS1 CNTN4 TCF12 RUNX1 ALDH1A2 RAPGEF1 PLS1 ROBO1 PKP4 DOK6 CHODL ACTN2 PRICKLE2 RNF165 FXR1 CLSTN1 EPHA3 LYN MYO3A DSCAM EPHA6 CA10 WDR72 NLGN1 HELLS SHROOM3 CAMK1D PCDH9 COBL CIT TCF7L2 RELN APP MAMLD1 CCDC88A ADAM12 PAK1 HCN1 FRY UTRN PPP3CA RASGRF1 RIN2 NEBL AP3B1 CPAMD8 TIAM1 SNTG2 NTM PCDH15 POR WWOX PTPN9 GREB1L TNRC6B INPP5F NXN HDAC4 CTNNBIP1 PPFIA2 AKAP6 OPCML LINGO2 RBFOX2 IMMP2L DLC1 ATP8A2 AUTS2 WLS ANKS1A SOS1 CTNND2 PTPN11 ALK LDLRAD4 DCLK1 TENM2 PSMB2 TFAP2D CYP7B1 PDS5B GLI3 RXFP1 NFAM1 KCNK2 ASXL3 DENND5A TNC NFASC MAP2K5 PLCB1 BPTF DNMT1 MAGI2 FLT3 EDA PRKD1 ABAT CELSR1 RYR2 ACIN1 VCL ROBO2 AKAP13 EYA4 HIVEP3 SEMA5A BCL11A DCC ETV6 DNAH5
GO:0032501	multicellular organismal process	1.367886E-04	*EPB41L4B EPN2 BANK1 ROCK2 OPHN1 CAMTA1 LRRTM3 ZBTB20 DLGAP1 RCAN1 KALRN SNTA1 CACNG2 BTBD9 MEGF11 RNF43 TRHDE MAP2K4 HS3ST5 MALL ARID1B IL1RAPL1 WDPCP GCLC ADAMTS6 STK39 RPS6KA2 CTNNA1 MYO9A NTRK2 FOXN3 SH3TC2 MDM2 ZNRF3 HBE1 PTPRG PLAC1 DIO2 SLC24A4 SYNE1 FBXL17 NHS DMD CHRM3 CALD1 SPRED2 MYO1D KAZN RAP1GDS1 KANK1 KIF26B CHST8 LAMA2 RAP1A TFDP2 DIAPH2 NOS1 CNTN4 TMC2 SLCO3A1 SND1 TCF12 CORO2B RUNX1 ALDH1A2 ESRRG CDH18 RAPGEF1 PLS1 ROBO1 PKP4 USP53 DOK6 CHODL ACTN2 PRICKLE2 RNF165 FXR1 CLSTN1 EPHA3 LYN MYO3A DSCAM EPHA6 CA10 WDR72 NLGN1 HELLS SHROOM3 KCNIP4 CAMK1D PCDH9 COBL CIT HERC4 TCF7L2 RELN APP MAMLD1 CCDC88A ADAM12 PAK1 HCN1 FRY DKK2 UTRN PPP3CA SPIRE2 RASGRF1 RIN2 UBR3 NEBL AP3B1 CPAMD8 TIAM1 PIEZO2 SNTG2 NTM PCDH15 GABRG3 POR WWOX PTPN9 KCNIP1 HUNK GREB1L TNRC6B INPP5F NXN NAMPT HDAC4 CTNNBIP1 POTEF PPFIA2 AKAP6 SORCS3 OPCML LINGO2 NPLOC4 RBFOX2 GRM7 IMMP2L SSPN ELAVL1 DLC1 ABCC1 ATP8A2 SCMH1 LOXHD1 AUTS2 PLAUR WLS ANKS1A RYR3 SOS1 CTNND2 CROCC PTPN11 PHF3 ZNF141 ALK LDLRAD4 DCLK1 TENM2 SYCP1 KCNB2 PSMB2 TFAP2D CYP7B1 SLC44A1 PDS5B GLI3 RXFP1 CTNNA3 NFAM1 KCNK2 ASXL3 DENND5A TNC DLG2 PPP1R12B MGLL NFASC MAP2K5 PLCB1 BPTF DNMT1 MAGI2 NLRP2 FLT3 PLCL1 EDA PRKD1 ABAT CELSR1 RYR2 ACIN1 EYS VCL ROBO2 AKAP13 EYA4 HIVEP3 SEMA5A BCL11A DCC ETV6 DNAH5*
GO:0048812	neuron projection morphogenesis	1.560573E-04	*OPHN1 IL1RAPL1 MYO9A NTRK2 DMD LAMA2 CNTN4 ROBO1 DOK6 CHODL RNF165 EPHA3 DSCAM EPHA6 NLGN1 COBL RELN APP PAK1 PPP3CA TIAM1 PPFIA2 RBFOX2 ATP8A2 AUTS2 SOS1 CTNND2 PTPN11 DCLK1 GLI3 NFASC VCL ROBO2 SEMA5A BCL11A DCC*
GO:0061564	axon development	1.772786E-04	*OPHN1 CTNNA1 NTRK2 LAMA2 CNTN4 ROBO1 DOK6 CHODL RNF165 EPHA3 DSCAM EPHA6 COBL RELN APP PAK1 TIAM1 INPP5F ATP8A2 AUTS2 SOS1 PTPN11 DCLK1 GLI3 TNC NFASC VCL ROBO2 SEMA5A BCL11A DCC*
GO:0032990	cell part morphogenesis	1.941227E-04	*OPHN1 IL1RAPL1 MYO9A NTRK2 DMD KANK1 LAMA2 CNTN4 ROBO1 DOK6 CHODL RNF165 EPHA3 DSCAM EPHA6 NLGN1 COBL RELN APP PAK1 PPP3CA TIAM1 PPFIA2 RBFOX2 ATP8A2 AUTS2 SOS1 CTNND2 PTPN11 DCLK1 GLI3 NFASC VCL ROBO2 SEMA5A BCL11A DCC*
GO:0032989	cellular component morphogenesis	2.015818E-04	*OPHN1 IL1RAPL1 MYO9A NTRK2 DMD KANK1 LAMA2 CNTN4 ROBO1 DOK6 CHODL ACTN2 RNF165 EPHA3 DSCAM EPHA6 NLGN1 COBL RELN APP PAK1 PPP3CA NEBL TIAM1 PPFIA2 RBFOX2 ATP8A2 AUTS2 SOS1 CTNND2 PTPN11 DCLK1 GLI3 NFASC VCL ROBO2 AKAP13 SEMA5A BCL11A DCC*

GO:0000904	cell morphogenesis involved in differentiation	3.891955E-04	*OPHN1 IL1RAPL1 WDPCP NTRK2 KANK1 LAMA2 CNTN4 PLS1 ROBO1 DOK6 CHODL RNF165 EPHA3 DSCAM EPHA6 NLGN1 COBL RELN APP PAK1 PPP3CA TIAM1 PCDH15 PPFIA2 RBFOX2 ATP8A2 AUTS2 SOS1 CTNND2 PTPN11 DCLK1 GLI3 NFASC VCL ROBO2 SEMA5A BCL11A DCC*
GO:0034329	cell junction assembly	6.322353E-04	*ROCK2 OPHN1 LRRTM3 IL1RAPL1 WDPCP CTNNA1 MYO9A NTRK2 RAP1A CORO2B RUNX1 CDH18 RAPGEF1 PKP4 ACTN2 CLSTN1 EPHA3 DSCAM NLGN1 APP LINGO2 DLC1 CTNND2 NFASC MAGI2 VCL ROBO2*
GO:0007275	multicellular organism development	7.408246E-04	*EPN2 ROCK2 OPHN1 LRRTM3 RCAN1 KALRN MEGF11 RNF43 MAP2K4 MALL ARID1B IL1RAPL1 WDPCP ADAMTS6 RPS6KA2 CTNNA1 MYO9A NTRK2 FOXN3 SH3TC2 MDM2 ZNRF3 PTPRG PLAC1 SLC24A4 FBXL17 NHS DMD CHRM3 SPRED2 MYO1D KAZN KANK1 KIF26B CHST8 LAMA2 RAP1A TFDP2 DIAPH2 NOS1 CNTN4 TCF12 RUNX1 ALDH1A2 CDH18 RAPGEF1 PLS1 ROBO1 PKP4 DOK6 CHODL ACTN2 PRICKLE2 RNF165 FXR1 CLSTN1 EPHA3 LYN MYO3A DSCAM EPHA6 CA10 WDR72 NLGN1 HELLS SHROOM3 CAMK1D PCDH9 COBL CIT TCF7L2 RELN APP MAMLD1 CCDC88A ADAM12 PAK1 HCN1 FRY DKK2 UTRN PPP3CA RASGRF1 RIN2 UBR3 NEBL AP3B1 CPAMD8 TIAM1 SNTG2 NTM PCDH15 POR WWOX PTPN9 HUNK GREB1L TNRC6B INPP5F NXN HDAC4 CTNNBIP1 PPFIA2 AKAP6 OPCML LINGO2 RBFOX2 IMMP2L DLC1 ATP8A2 SCMH1 AUTS2 WLS ANKS1A SOS1 CTNND2 PTPN11 PHF3 ZNF141 ALK LDLRAD4 DCLK1 TENM2 PSMB2 TFAP2D CYP7B1 PDS5B GLI3 RXFP1 NFAM1 KCNK2 ASXL3 DENND5A TNC NFASC MAP2K5 PLCB1 BPTF DNMT1 MAGI2 FLT3 EDA PRKD1 ABAT CELSR1 RYR2 ACIN1 VCL ROBO2 AKAP13 EYA4 HIVEP3 SEMA5A BCL11A DCC ETV6 DNAH5*
GO:0007409	axonogenesis	1.021266E-03	*OPHN1 NTRK2 LAMA2 CNTN4 ROBO1 DOK6 CHODL RNF165 EPHA3 DSCAM EPHA6 COBL RELN APP PAK1 TIAM1 ATP8A2 AUTS2 SOS1 PTPN11 DCLK1 GLI3 NFASC VCL ROBO2 SEMA5A BCL11A DCC*
GO:0099537	trans-synaptic signaling	1.072370E-03	*SYN3 OPHN1 DLGAP1 CACNG2 BTBD9 ARID1B IL1RAPL1 NTRK2 CHRM3 LAMA2 RAP1A NOS1 CNTN4 GRIK4 GPR176 FCHSD2 CLSTN1 NLGN1 RELN APP PPP3CA RASGRF1 SORCS2 GABRG3 ERC2 PPFIA2 SORCS3 GRM7 GABBR2 DLG2 PLCB1 PLCL1 ABAT UNC13C DCC CASK*
GO:0008038	neuron recognition	1.091704E-03	*CNTN4 ROBO1 EPHA3 DSCAM APP NTM OPCML ROBO2 SEMA5A*
GO:0051130	positive regulation of cellular component organization	1.233982E-03	*ROCK2 LRRTM3 IL1RAPL1 NTRK2 PDE4DIP DMD RAP1A TFDP2 NOS1 TBC1D5 RAPGEF1 ROBO1 SEPT9 FCHSD2 CHODL ACTN2 CLSTN1 EPHA3 LYN DSCAM NLGN1 CAMK1D COBL CLEC16A PLEKHM2 RELN APP CCDC88A PAK1 PPP3CA SPIRE2 TIAM1 HDAC4 LINGO2 ATP8A2 AUTS2 PLAUR CROCC ALK TENM2 FAF1 ATF7IP PLCB1 DNMT1 MAGI2 PRKD1 ROBO2 SEMA5A BCL11A*
GO:0000902	cell morphogenesis	2.149368E-03	*OPHN1 IL1RAPL1 WDPCP MYO9A NTRK2 DMD KANK1 LAMA2 CNTN4 CDH18 PLS1 ROBO1 DOK6 CHODL RNF165 EPHA3 DSCAM EPHA6 NLGN1 SHROOM3 COBL RELN APP PAK1 FRY PPP3CA AP3B1 TIAM1 PCDH15 PPFIA2 RBFOX2 DLC1 ATP8A2 AUTS2 SOS1 CTNND2 PTPN11 DCLK1 GLI3 NFASC VCL ROBO2 SEMA5A BCL11A DCC*
GO:0010976	positive regulation of neuron projection development	2.278700E-03	*NTRK2 DMD RAP1A RAPGEF1 EPHA3 LYN CAMK1D COBL RELN TIAM1 ATP8A2 ALK MAGI2 PRKD1 BCL11A*
GO:0099536	synaptic signaling	2.284887E-03	*SYN3 OPHN1 DLGAP1 CACNG2 BTBD9 ARID1B IL1RAPL1 NTRK2 CHRM3 LAMA2 RAP1A NOS1 CNTN4 GRIK4 GPR176 FCHSD2 CLSTN1 NLGN1 RELN APP PPP3CA RASGRF1 SORCS2 GABRG3 ERC2 PPFIA2 SORCS3 GRM7 GABBR2 DLG2 PLCB1 PLCL1 ABAT UNC13C DCC CASK*
GO:0009966	regulation of signal transduction	2.712669E-03	*EPN2 BANK1 ROCK2 OPHN1 CAMTA1 DLGAP1 RCAN1 OTUD7A KALRN CACNG2 RNF43 MAP2K4 GCLC STK39 ARHGAP8 CTNNA1 MYO9A NTRK2 CCDC3 SH3TC2 MDM2 ZNRF3 NCOR1 FBXL17 SLC39A10 DMD SPRED2 RPTOR KANK1 RAP1A SH2D1A NOS1 RGS3 RUNX1 RAPGEF1 ROBO1 ACTN2 RNF165 LYN NLGN1 HELLS TNFRSF10B CSNK1G3*

			CLEC16A HERC4 TCF7L2 RELN APP CCDC88A PAK1 DKK2 GPC5 WDR59 RASGRF1 OTUD4 DEPDC5 TIAM1 USP18 POR WWOX GSG1L INPP5F SHC2 NXN CTNNBIP1 AKAP6 NPLOC4 DLC1 AUTS2 PLAUR WLS ANKS1A SOS1 CTNND2 PTPN11 ALK LDLRAD4 RALGAPA2 FAF1 PSMB2 CYP7B1 PTPRT GLI3 NFAM1 RNF220 DLG2 MGLL MAP2K5 PLCB1 MAGI2 FLT3 EDA PRKD1 ABAT ROBO2 AKAP13 EYA4 SEMA5A DCC
GO:0016043	cellular component organization	3.693823E-03	*EPB41L4B EPN2 STX8 ROCK2 DNAJC15 CCNE1 OPHN1 LRRTM3 KIAA0753 CACNG2 BTBD9 ARID1B IL1RAPL1 WDPCP VPS37B GCLC ADAMTS6 RPS6KA2 HFM1 CTNNA1 MYO9A NTRK2 SH3TC2 MDM2 STAG1 PTPRG NCOR1 SYNE1 PDE4DIP DMD EIF3E ITGAE MYO1D GULP1 RAP1GDS1 RPTOR KANK1 LAMA2 RAP1A TFDP2 DIAPH2 NOS1 STAG2 TBC1D5 CNTN4 CORO2B RUNX1 ALDH1A2 CDH18 RAPGEF1 PLS1 ROBO1 PKP4 SEPT9 RBL2 DOK6 FCHSD2 CHODL ACTN2 RNF165 FXR1 CLSTN1 EPHA3 LRRTM4 LYN MTA3 DSCAM EPHA6 WDR72 NLGN1 HELLS DNAH9 SHROOM3 CAMK1D SMG6 COBL MDN1 CIT CLEC16A TCF7L2 PLEKHM2 RELN APP CCDC88A ADAM12 PAK1 HCN1 FRY UTRN PPP3CA SPIRE2 RASGRF1 SCP2 NEBL AP3B1 STON1 TIAM1 PRIM2 CHAF1A NSD1 PRMT8 KANK3 PCDH15 ERC2 PTPN9 GSG1L INPP5F HDAC4 CTNNBIP1 ATP9A PPFIA2 AKAP6 LINGO2 NPLOC4 RBFOX2 IMMP2L ELAVL1 DLC1 ABCC1 ATP8A2 SCMH1 AUTS2 PLAUR RYR3 SOS1 CTNND2 FRMD3 CROCC ASCC3 PTPN11 ALK REEP3 LDLRAD4 CADM2 DCLK1 TENM2 SYCP1 PSMG2 KCNB2 FAF1 SNRPD1 PDS5B SNUPN PTPRT GLI3 RXFP1 ABHD17C ATF7IP DENND5A TNC DLG2 NFASC MAP2K5 PLCB1 BPTF NPEPPS DNMT1 MAGI2 DNAJC2 PRKD1 CELSR1 ACIN1 VCL ROBO2 AKAP13 UNC13C EYA4 SEMA5A BCL11A DCC ETV6 DNAH5*
GO:0050808	synapse organization	4.365431E-03	*OPHN1 LRRTM3 CACNG2 IL1RAPL1 NTRK2 CLSTN1 LRRTM4 DSCAM NLGN1 RELN APP UTRN TIAM1 ERC2 PPFIA2 LINGO2 CTNND2 PTPRT ABHD17C TNC DLG2 NFASC MAGI2 ROBO2 UNC13C*
GO:0016055	Wnt signaling pathway	5.358907E-03	*CCNE1 RNF43 ZNRF3 KANK1 RUNX1 RAPGEF1 PRICKLE2 CSNK1G3 TCF7L2 APP DKK2 GPC5 PPP3CA TIAM1 WWOX TNRC6B NXN CTNNBIP1 WLS CTNND2 PSMB2 GLI3 RNF220 PLCB1 MAGI2 EDA CELSR1 SEMA5A*
GO:0198738	cell-cell signaling by wnt	5.759785E-03	*CCNE1 RNF43 ZNRF3 KANK1 RUNX1 RAPGEF1 PRICKLE2 CSNK1G3 TCF7L2 APP DKK2 GPC5 PPP3CA TIAM1 WWOX TNRC6B NXN CTNNBIP1 WLS CTNND2 PSMB2 GLI3 RNF220 PLCB1 MAGI2 EDA CELSR1 SEMA5A*
GO:0098916	anterograde trans-synaptic signaling	6.148080E-03	*SYN3 OPHN1 DLGAP1 CACNG2 BTBD9 ARID1B NTRK2 CHRM3 LAMA2 RAP1A CNTN4 GRIK4 GPR176 FCHSD2 CLSTN1 NLGN1 RELN APP PPP3CA RASGRF1 SORCS2 GABRG3 ERC2 PPFIA2 SORCS3 GRM7 GABBR2 DLG2 PLCB1 PLCL1 ABAT UNC13C DCC CASK*
GO:0007268	chemical synaptic transmission	6.148080E-03	*SYN3 OPHN1 DLGAP1 CACNG2 BTBD9 ARID1B NTRK2 CHRM3 LAMA2 RAP1A CNTN4 GRIK4 GPR176 FCHSD2 CLSTN1 NLGN1 RELN APP PPP3CA RASGRF1 SORCS2 GABRG3 ERC2 PPFIA2 SORCS3 GRM7 GABBR2 DLG2 PLCB1 PLCL1 ABAT UNC13C DCC CASK*
GO:1904062	regulation of cation transmembrane transport	7.244389E-03	*SNTA1 CACNG2 STK39 DMD NOS1 TMC2 ACTN2 LYN NLGN1 KCNIP4 RELN DPP10 APP UTRN RASGRF1 KCNIP1 GSG1L AKAP6 DLG2 PRKD1 RYR2 NPSR1*
GO:0060341	regulation of cellular localization	7.445081E-03	*STX8 ROCK2 CCNE1 OPHN1 CACNG2 CTNNA1 SH3TC2 MDM2 DMD RAP1A TFDP2 NOS1 PLS1 ACTN2 EPHA3 LYN NLGN1 TCF7L2 DPP10 CCDC88A PAK1 GPC5 SCP2 TIAM1 PTPN9 GSG1L INPP5F AKAP6 CROCC PTPN11 DCLK1 GLI3 ABHD17C NPEPPS MAGI2 PRKD1 RYR2 NPSR1*
GO:0050804	modulation of chemical synaptic transmission	7.952224E-03	*SYN3 OPHN1 DLGAP1 CACNG2 BTBD9 NTRK2 LAMA2 RAP1A CNTN4 GRIK4 CLSTN1 NLGN1 RELN APP PPP3CA RASGRF1 SORCS2 PPFIA2 SORCS3 GRM7 PLCB1 PLCL1 UNC13C DCC CASK*

GO:0099177	regulation of trans-synaptic signaling	8.267872E-03	*SYN3 OPHN1 DLGAP1 CACNG2 BTBD9 NTRK2 LAMA2 RAP1A CNTN4 GRIK4 CLSTN1 NLGN1 RELN APP PPP3CA RASGRF1 SORCS2 PPFIA2 SORCS3 GRM7 PLCB1 PLCL1 UNC13C DCC CASK*
GO:1901888	regulation of cell junction assembly	8.919644E-03	*ROCK2 LRRTM3 IL1RAPL1 WDPCP NTRK2 RAP1A RUNX1 RAPGEF1 CLSTN1 EPHA3 NLGN1 APP LINGO2 DLC1 VCL ROBO2*
GO:1905114	cell surface receptor signaling pathway involved in cell-cell signaling	9.092852E-03	*CCNE1 RNF43 ZNRF3 KANK1 RUNX1 RAPGEF1 PRICKLE2 NLGN1 CSNK1G3 TCF7L2 RELN APP DKK2 GPC5 PPP3CA TIAM1 WWOX TNRC6B NXN CTNNBIP1 WLS CTNND2 PSMB2 GLI3 RNF220 PLCB1 MAGI2 EDA ABAT CELSR1 SEMA5A*
GO:0044093	positive regulation of molecular function	9.543297E-03	*ROCK2 DNAJC15 OPHN1 MOB1B CACNG2 MAP2K4 STK39 ARHGAP8 MYO9A NTRK2 TBC1D22A SLC39A10 DMD EIF3E RAP1GDS1 RPTOR RAP1A NOS1 RGS3 TBC1D5 SLCO3A1 RAPGEF1 ROBO1 PKP4 ACTN2 EPHA3 LYN EPHA6 TNFRSF10B CAMK1D TCF7L2 RELN APP CCDC88A PAK1 PPP3CA RASGRF1 RIN2 DEPDC5 TIAM1 POR SHC2 HDAC4 AKAP6 DLC1 PLAUR SOS1 PTPN11 ALK RALGAPA2 SGSM1 NFAM1 PPP1R12B MAP2K5 PLCB1 MAGI2 NLRP2 FLT3 PLCL1 EDA PRKD1 RYR2 AKAP13 KHDC1*
GO:0003012	muscle system process	2.027280E-02	*ROCK2 SNTA1 MAP2K4 DMD CHRM3 CALD1 RAP1GDS1 NOS1 ACTN2 PAK1 UTRN PPP3CA TIAM1 INPP5F HDAC4 AKAP6 SSPN ATP8A2 KCNB2 CTNNA3 PPP1R12B ABAT RYR2 VCL AKAP13*
GO:0016358	dendrite development	2.424757E-02	*ARID1B IL1RAPL1 DSCAM NLGN1 CAMK1D COBL RELN APP PPP3CA TIAM1 PPFIA2 RBFOX2 CTNND2 ALK DCLK1 BCL11A DCC*
GO:0071840	cellular component organization or biogenesis	2.651434E-02	*EPB41L4B EPN2 STX8 ROCK2 DNAJC15 CCNE1 OPHN1 LRRTM3 KIAA0753 CACNG2 BTBD9 ARID1B IL1RAPL1 WDPCP VPS37B GCLC ADAMTS6 RPS6KA2 HFM1 CTNNA1 MYO9A NTRK2 SH3TC2 MDM2 STAG1 PTPRG NCOR1 SYNE1 PDE4DIP DMD EIF3E ITGAE MYO1D GULP1 RAP1GDS1 RPTOR KANK1 LAMA2 RAP1A TFDP2 DIAPH2 NOS1 STAG2 TBC1D5 CNTN4 CORO2B RUNX1 ALDH1A2 CDH18 RAPGEF1 PLS1 ROBO1 PKP4 SEPT9 RBL2 DOK6 FCHSD2 CHODL ACTN2 RNF165 FXR1 CLSTN1 EPHA3 LRRTM4 LYN MTA3 DSCAM EPHA6 WDR72 NLGN1 HELLS DNAH9 SHROOM3 CAMK1D SMG6 COBL MDN1 CIT CLEC16A TCF7L2 PLEKHM2 RELN APP CCDC88A ADAM12 PAK1 HCN1 FRY UTRN PPP3CA SPIRE2 RASGRF1 SCP2 NEBL AP3B1 STON1 TIAM1 PRIM2 CHAF1A NSD1 PRMT8 KANK3 PCDH15 ERC2 PTPN9 GSG1L INPP5F HDAC4 CTNNBIP1 ATP9A PPFIA2 AKAP6 LINGO2 NPLOC4 RBFOX2 IMMP2L ELAVL1 DLC1 ABCC1 ATP8A2 SCMH1 AUTS2 PLAUR RYR3 SOS1 CTNND2 FRMD3 CROCC ASCC3 PTPN11 ALK REEP3 LDLRAD4 CADM2 DCLK1 TENM2 SYCP1 PSMG2 KCNB2 FAF1 SNRPD1 PDS5B SNUPN PTPRT GLI3 RXFP1 ABHD17C ATF7IP DENND5A TNC DLG2 NFASC MAP2K5 PLCB1 BPTF NPEPPS DNMT1 MAGI2 DNAJC2 PRKD1 CELSR1 ACIN1 VCL ROBO2 AKAP13 UNC13C EYA4 SEMA5A BCL11A DCC ETV6 DNAH5*
GO:0007264	small GTPase mediated signal transduction	3.063129E-02	*ROCK2 OPHN1 KALRN ARHGAP8 MYO9A RASGEF1B KANK1 RAP1A RAPGEF1 ROBO1 LYN RELN CCDC88A RASGRF1 RIN2 KIAA0355 TIAM1 SHC2 DLC1 AUTS2 SOS1 RALGAPA2 DNMT1 PRKD1 CELSR1 AKAP13*
GO:0007411	axon guidance	3.291360E-02	*OPHN1 LAMA2 CNTN4 ROBO1 DOK6 RNF165 EPHA3 DSCAM EPHA6 RELN APP SOS1 PTPN11 GLI3 NFASC ROBO2 SEMA5A DCC*
GO:0048856	anatomical structure development	3.405856E-02	*EPN2 ROCK2 OPHN1 LRRTM3 RCAN1 KALRN MEGF11 RNF43 MAP2K4 MALL ARID1B IL1RAPL1 WDPCP ADAMTS6 RPS6KA2 CTNNA1 MYO9A NTRK2 FOXN3 SH3TC2 MDM2 ZNRF3 PTPRG PLAC1 SLC24A4 SYNE1 FBXL17 NHS DMD CHRM3 SPRED2 MYO1D KAZN KANK1 KIF26B CHST8 LAMA2 RAP1A TFDP2 DIAPH2 NOS1 CNTN4 TCF12 RUNX1 ALDH1A2 CDH18 RAPGEF1 PLS1 ROBO1 PKP4 DOK6 CHODL ACTN2 PRICKLE2 RNF165 FXR1 CLSTN1 EPHA3 LYN MYO3A DSCAM EPHA6 CA10 WDR72 NLGN1 HELLS SHROOM3 CAMK1D PCDH9 COBL CIT TCF7L2 RELN APP MAMLD1 CCDC88A ADAM12 PAK1 HCN1 FRY DKK2*

			UTRN PPP3CA RASGRF1 RIN2 UBR3 NEBL AP3B1 CPAMD8 TIAM1 SNTG2 NTM PCDH15 POR WWOX PTPN9 HUNK GREB1L TNRC6B INPP5F NXN HDAC4 CTNNBIP1 PPFIA2 AKAP6 OPCML LINGO2 RBFOX2 IMMP2L DLC1 ATP8A2 SCMH1 AUTS2 WLS ANKS1A SOS1 CTNND2 PTPN11 PHF3 ZNF141 ALK LDLRAD4 DCLK1 TENM2 SYCP1 PSMB2 TFAP2D CYP7B1 PDS5B GLI3 RXFP1 NFAM1 KCNK2 ASXL3 DENND5A TNC NFASC MAP2K5 PLCB1 BPTF DNMT1 MAGI2 FLT3 EDA PRKD1 ABAT CELSR1 RYR2 ACIN1 EYS VCL ROBO2 AKAP13 EYA4 HIVEP3 SEMA5A BCL11A DCC ETV6 DNAH5
GO:0032502	developmental process	3.446972E-02	*EPN2 ROCK2 OPHN1 WDFY2 LRRTM3 RCAN1 KALRN MEGF11 RNF43 MAP2K4 MALL ARID1B IL1RAPL1 WDPCP GCLC ADAMTS6 RPS6KA2 CTNNA1 MYO9A NTRK2 FOXN3 CCDC3 SH3TC2 MDM2 ZNRF3 PTPRG PLAC1 DIO2 SLC24A4 SYNE1 FBXL17 NHS DMD CHRM3 SPRED2 MYO1D KAZN KANK1 KIF26B CHST8 LAMA2 RAP1A TFDP2 DIAPH2 NOS1 NEK4 CNTN4 SND1 TCF12 RUNX1 ALDH1A2 CDH18 RAPGEF1 PLS1 ROBO1 PKP4 RBL2 DOK6 CHODL ACTN2 PRICKLE2 RNF165 FXR1 CLSTN1 EPHA3 LYN MYO3A DSCAM EPHA6 CA10 WDR72 NLGN1 HELLS SHROOM3 CAMK1D PCDH9 COBL CIT HERC4 TCF7L2 RELN APP MAMLD1 CCDC88A ADAM12 PAK1 HCN1 FRY DKK2 UTRN PPP3CA RASGRF1 RIN2 UBR3 NEBL AP3B1 CPAMD8 TIAM1 SNTG2 NTM PCDH15 POR WWOX PTPN9 HUNK GREB1L TNRC6B INPP5F NXN NAMPT HDAC4 CTNNBIP1 PPFIA2 AKAP6 OPCML LINGO2 RBFOX2 IMMP2L ELAVL1 DLC1 ATP8A2 SCMH1 AUTS2 WLS ANKS1A SOS1 CTNND2 PTPN11 PHF3 ZNF141 ALK LDLRAD4 DCLK1 TENM2 SYCP1 PSMB2 TFAP2D CYP7B1 PDS5B GLI3 RXFP1 NFAM1 KCNK2 ASXL3 DENND5A TNC NFASC MAP2K5 PLCB1 BPTF DNMT1 MAGI2 FLT3 EDA PRKD1 ABAT CELSR1 RYR2 ACIN1 EYS VCL ROBO2 AKAP13 UNC13C EYA4 HIVEP3 SEMA5A BCL11A DCC ETV6 DNAH5*
GO:0097485	neuron projection guidance	3.450587E-02	*OPHN1 LAMA2 CNTN4 ROBO1 DOK6 RNF165 EPHA3 DSCAM EPHA6 RELN APP SOS1 PTPN11 GLI3 NFASC ROBO2 SEMA5A DCC*
GO:0019220	regulation of phosphate metabolic process	4.801776E-02	*BANK1 ROCK2 DNAJC15 CCNE1 CAMTA1 WDFY2 ZBTB20 MOB1B RCAN1 MAP2K4 STK39 NTRK2 NCOR1 SLC39A10 DMD TMEM132D SPRED2 MYO1D RPTOR RAP1A NOS1 RGS3 SLCO3A1 RAPGEF1 ROBO1 RBL2 FXR1 EPHA3 LYN DSCAM EPHA6 TNFRSF10B SMG6 RELN APP CCDC88A PAK1 SCP2 TIAM1 NSD1 INPP5F SHC2 HDAC4 DLC1 PLAUR PTPN11 ALK LDLRAD4 FAF1 PTPRT DLG2 MAP2K5 MAGI2 FLT3 PLCL1 PRKD1 AKAP13*
GO:0051174	regulation of phosphorus metabolic process	4.885474E-02	*BANK1 ROCK2 DNAJC15 CCNE1 CAMTA1 WDFY2 ZBTB20 MOB1B RCAN1 MAP2K4 STK39 NTRK2 NCOR1 SLC39A10 DMD TMEM132D SPRED2 MYO1D RPTOR RAP1A NOS1 RGS3 SLCO3A1 RAPGEF1 ROBO1 RBL2 FXR1 EPHA3 LYN DSCAM EPHA6 TNFRSF10B SMG6 RELN APP CCDC88A PAK1 SCP2 TIAM1 NSD1 INPP5F SHC2 HDAC4 DLC1 PLAUR PTPN11 ALK LDLRAD4 FAF1 PTPRT DLG2 MAP2K5 MAGI2 FLT3 PLCL1 PRKD1 AKAP13*

Table S5. Expression levels (TPM) of srRNA genes in HEK293T cells and the numbers of the corresponding srRNAs. Excel file attached separately. Related to Fig. 4A,B.

Table S6. Accession numbers and the descriptions of the dataset used.

Database	Accession	Description	Comment

GEO	GSM955512/SRR518497	HEK293T small RNA	Raw RNA-Seq reads
Genbank	U13369	H. sapiens rDNA	DNA Sequence
Encode	ENCFF924LOC	HEK293 CTCF	Encode processed profile
Encode	ENCFF716SFD	HEK293T DNAseI	Encode processed profile
Encode	ENCFF717JWL	HEK293 H3K4me1	Encode processed profile
Encode	ENCFF756EHF	HEK293 H3K4me3	Encode processed profile
Encode	ENCFF902RQI	HEK293 H3K9me3	Encode processed profile
Encode	ENCFF631VZK	HEK293 H3K27ac	Encode processed profile
Encode	ENCFF367HGG	HEK293 ZNF263	Encode processed profile
Encode	ENCFF128ERM	HEK293T ZNF384	Encode processed profile
SRA	SRR1910478, SRR1910479	HEK293 DDX21	Raw ChIP-Seq reads
SRA	SRR1001897, SRR1001898, SRR1001900	HEK293 CBP	Raw ChIP-Seq reads
SRA	SRR1001893, SRR1001894, SRR1001900	HEK293 p300	Raw ChIP-Seq reads
SRA	SRR710096, SRR710097	HEK293T RAD21	Raw ChIP-Seq reads
GEO	GSM935534, GSM935533	HEK293 POLR2A	Raw ChIP-Seq reads
SRA	SRR087747, SRR087753	HEK293T RPA116/POLR1B	Raw ChIP-Seq reads
SRA	SRR087746, SRR087753	HEK293T UBF	Raw ChIP-Seq reads
GEO	GSE121413	HEK293T 4C-rDNA	Raw DNA-Seq reads
GEO	GSE130262	HEK293T gene expression	Raw RNA-Seq reads
NCBI RefSeq	ncbiRefSeqCurated hg38	*H. sapiens* hg38 genes	RefSeq genes list
EPD	CAGE/Fantom5	HEK293T	Genome-wide expression
NIH Roadmap Epigenomics	E007 epigenome	H1-derived neuronal progenitor cultured cells, ESC-derived	Chromatine core 15-state model

Supplementary Text

Methylation at srRNA target sites

Encode ENCSR794HFF accession was used to obtain HEK293 methylation track: ENCFF001TMR and ENCFF001TMQ replicates were downloaded, and an intersection genome track was created by bedtools intersectBed tool.

Encode does not contain HEK293 or HEK293T methylation data mapped to the hg38 genome build and so we remapped all srRNA data to hg19 according to the protocol in the Methods section. The mapping produced 2758 mapping regions.

Next, we calculated the direct intersection between HEK293 methylation and srRNA genome tracks (whole-genome targets of srRNAs). The tracks intersected in 1545 regions or 56.02% of the srRNA genome track. We calculated the genome-wide correlations between these tracks by GenomeTrackAnalyzer [1] and StereoGene[2]. The results of both programs (z=2.447 p=0.0144 for GenomeTrackAnalyzer and Mann-Z=2.908 p=0.0037 for StereoGene) imply that srRNA targets and methylation sites correlate significantly. Thus, we can conclude that the srRNAs targets are often methylated.

1. Kravatsky, Y. V., Chechetkin, V. R., Tchurikov, N. A. & Kravatskaya, G. I. Genome-wide study of correlations between genomic features and their relationship with the regulation of gene expression. *DNA Res* **22**, 109-119, doi:10.1093/dnares/dsu044 (2015).
2. Stavrovskaya, E. D. *et al.* StereoGene: rapid estimation of genome-wide correlation of continuous or interval feature data. *Bioinformatics* **33**, 3158-3165, doi:10.1093/bioinformatics/btx379 (2017).
